# KGML-xDTD: a knowledge graph–based machine learning framework for drug treatment prediction and mechanism description

**DOI:** 10.1093/gigascience/giad057

**Published:** 2023-08-21

**Authors:** Chunyu Ma, Zhihan Zhou, Han Liu, David Koslicki

**Affiliations:** Huck Institutes of Life Sciences, Pennsylvania State University, State College, PA 16801, USA; Department of Computer Science, Northwestern University, Evanston, IL 60208, USA; Department of Computer Science, Northwestern University, Evanston, IL 60208, USA; Huck Institutes of Life Sciences, Pennsylvania State University, State College, PA 16801, USA; Department of Computer Science and Engineering, Pennsylvania State University, State College, PA 16801, USA; Department of Biology, Pennsylvania State University, State College, PA 16801, USA

**Keywords:** drug repurposing, reinforcement learning, biomedical knowledge graph

## Abstract

**Background:**

Computational drug repurposing is a cost- and time-efficient approach that aims to identify new therapeutic targets or diseases (indications) of existing drugs/compounds. It is especially critical for emerging and/or orphan diseases due to its cheaper investment and shorter research cycle compared with traditional wet-lab drug discovery approaches. However, the underlying mechanisms of action (MOAs) between repurposed drugs and their target diseases remain largely unknown, which is still a main obstacle for computational drug repurposing methods to be widely adopted in clinical settings.

**Results:**

In this work, we propose KGML-xDTD: a **K**nowledge **G**raph–based **M**achine **L**earning framework for e**x**plainably predicting **D**rugs **T**reating **D**iseases. It is a 2-module framework that not only predicts the treatment probabilities between drugs/compounds and diseases but also biologically explains them via knowledge graph (KG) path-based, testable MOAs. We leverage knowledge-and-publication–based information to extract biologically meaningful “demonstration paths” as the intermediate guidance in the Graph-based Reinforcement Learning (GRL) path-finding process. Comprehensive experiments and case study analyses show that the proposed framework can achieve state-of-the-art performance in both predictions of drug repurposing and recapitulation of human-curated drug MOA paths.

**Conclusions:**

KGML-xDTD is the first model framework that can offer KG path explanations for drug repurposing predictions by leveraging the combination of prediction outcomes and existing biological knowledge and publications. We believe it can effectively reduce “black-box” concerns and increase prediction confidence for drug repurposing based on predicted path-based explanations and further accelerate the process of drug discovery for emerging diseases.

## Introduction

Traditional drug development is a time-consuming process (from initial chemical identification to clinical trials and finally to Food and Drug Administration [FDA] approval) that takes around 10 to 15 years and also comes along with billions-of-dollars investments and high failure rates [1]. Considering the rapid pace of novel disease evolution, it is urgent to find a more efficient and economical drug discovery method. Fortunately, it has been observed that a single drug can often be effective in treating multiple diseases. For example, thalidomide was originally used as an antianxiety medication [2] and was later found to have the anticancer potential for the treatment of cancers [3, 4]. Hence, drug repurposing, also known as the identification of new uses for existing drugs/compounds, might bring us hope to address this urgent need with the advantage of a shorter research cycle, lower development cost, and more preexisting safety tests.

Existing drug repurposing approaches can roughly be categorized into experimental-based approaches (e.g., binding affinity assays [5], phenotypic screening [6]), clinical-based approaches (e.g., off-label drug use analysis [7]), and computational-based approaches (e.g., chemical structure–based [8] and GWAS-based approaches [9]). Compared with the former 2 approaches, the computational approaches are more cost- and time-efficient, particularly when the goal is to prioritize a large number of target drugs/compounds for follow-up experimental investigation. Among all computational drug repurposing methods, the integration of multiple biomedical data sources into a so-called biomedical knowledge graph (BKG) for drug discovery has become popular in recent years [10] due to the increasing availability of curated biomedical databases such as DrugBank [11], ChEMBL [12], and HMDB [13] and the advancement of semantic web techniques [14]. There are 3 types of existing BKGs: database-based BKGs, literature-based BKGs, and mixed BKGs. The database-based BKGs (e.g., *Hetionet* [15], *BioKG* [16], *iBKH* [17]) are constructed by integrating biomedical data and their relations stored in existing biological databases. The literature-based BKGs (e.g., *GNBR* [18]) are built by leveraging Natural Language Processing (NLP) techniques to extract semantic information from a large amount of available biomedical literature and electronic health record (EHR) data, which are mostly disease specific [19–21]. The mixed BKGs (e.g., *CKG* [22], *RTX-KG2* [23]) are generated by combining the knowledge sources from the above 2 methods.

Based on these BKGs, several machine learning methods have been proposed or implemented for drug repurposing prediction by treating it as a link prediction task in the BKGs. For example, Himmelstein et al. [15] used the so-called degree-weighted path count (DWPC) to assess the prevalence of 1,206 metapaths and then classified drug–disease treatment relations by fitting these DWPC features to a logistic regression model. Ioannidis et al. [24] proposed a novel graph neural network model I-RGCN to learn the node and relation embeddings for the Covid-19 drug repurposing task. Zhang et al. [19] recently predicted the possible drugs for Covid-19 with 5 existing popular knowledge graph completion methods (e.g., TransE [25], RotatE [26], DistMult [27], ComplEx [28], and STELP [29]). Although some of these models have shown good performance in drug repurposing prediction on the small-scale BKGs, none have been scaled to massive BKGs with more than millions of nodes and edges and make a comprehensive comparison. More importantly, most of them lack the biological explanatory ability for their predictions, which limits their applicability in clinical research.

Currently, there are few computational models designed for drug repurposing *explanations*. A common and intuitive explanation based on a biomedical knowledge graph for drug repurposing leverages the semantic BKG-based paths between given drug–disease pairs. Sosa et al. [30] applied a graph embedding model UKGE [31], which utilizes the weighted (the frequency of relation appeared in literature) relation edges in a literature-based KG *GNBR* to identify new indications of drugs for rare diseases and then explain the results via the highest-ranking paths based on confidence scores. However, this method is only applicable in the literature-based BKGs with the weighted edge information. Most BKGs using database-based knowledge do not contain such information. Sang et al. [32] proposed GrEDeL that combines the TransE embedding method with a long short-term memory (LSTM) recurrent neural network (RNN) model to predict drug–disease relation. By using the embeddings of BKG paths as model input for predictions, they can provide path-based explanations. However, they claimed that the effectiveness of the approach relies heavily on the NLP tool SemRep, which is reported to have high false positives in named entity recognition [33]. Also, they did not fully evaluate how biologically reasonable their predicted path-based mechanisms of action (MOAs) are.

Besides the existing methods above, we view reinforcement learning (RL) as a promising solution for drug repurposing explanation. RL models solve the decision-making problem, in which an agent learns how to take appropriate actions to maximize cumulative rewards through interactions with the environment. RL has achieved widespread success in various domains, including games, recommendation systems, health care, transportation, and so on [34]. Graph reinforcement learning (GRL), first proposed around in 2017, aims to solve graph mining tasks such as link prediction [35], adversarial attacks [36], and relational reasoning [37]. Unlike its applications in other domains, one of the biggest challenges in GRL is finding an appropriate reward to guide the path searching in specific domains. To address the issue of finding biologically reasonable BKG-based paths for drug repurposing, it is crucial to incorporate biomedical domain knowledge to guide the path-finding process. Liu et al. [38] developed an RL-based model “PoLo” that utilizes the biological meta-paths identified in Himmelstein et al. [15] via the “DWPC” method to supervise path searching for drug repurposing. However, the “PoLo” model does not scale to a massive and complex BKG (e.g., CKG and RTX-KG2) due to its dependence on the “DWPC” method that is reported to be computationally inefficient [39].

In this article, we describe KGML-xDTD: a **K**nowledge **G**raph–based **M**achine **L**earning framework for e**x**plainably predicting **D**rugs **T**reating **D**iseases, which contains 2 modules for both drug repurposing prediction and MOA explanation. We propose to amplify the ability of the RL model in biologically meaningful path searching by utilizing the biologically meaningful “demonstration paths” and pretrained drug repurposing model probability as rewards. We incorporate this idea into the appropriate models (e.g., GraphSAGE [40], random forest, and ADAC RL [41] models) and then make them applicable to the explainable drug repurposing problem at massive data scale and complexity. By comparing with the existing popular drug repurposing models and evaluating the predicted paths with an expert-curated path-based drug MOA database *DrugMechDB* [42, 43], we show that the proposed model framework can achieve state-of-the-art performance in both predictions of drug repurposing and recapitulation of human-curated drug MOA paths provided by DrugMechDB. In further case studies, by comparing the model predictions with the real regulatory networks, we show that the proposed framework effectively identifies biologically reasonable BKG-based MOA paths for real-world applications.

## Materials and Methods

### Datasets

#### Customized biomedical knowledge graph

**Figure 1:**
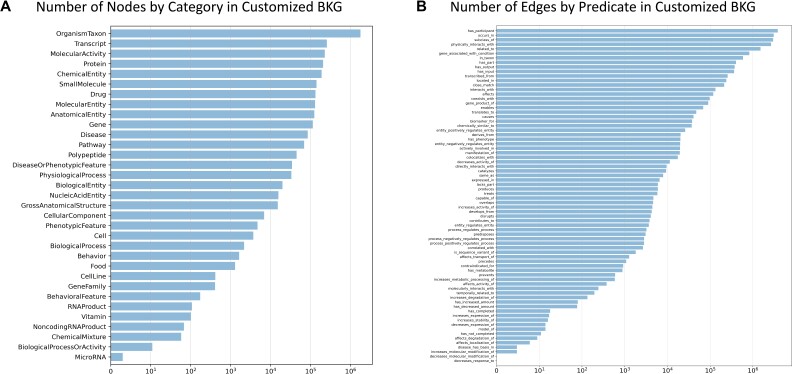
Number of nodes by category (A) and number of edges by predicate (B) in the customized BKG.

To accommodate biomedical-reasonable predictions of drugs’ indications and their mechanisms of action, the ideal biomedical knowledge graph should integrate biomedical knowledge from comprehensive and diverse databases and publications, as well as accurately identify and merge different identifiers representing the same biological entity into one (e.g., “CHEBI:2367” and “CHEMBL455626” are 2 distinct identifiers separately presented in the ChEBI database [44] and ChEMBL database [12] but represent the same compound “abyssinone I”). Thus, we utilize the canonicalized version of the Reasoning Tool X Knowledge Graph 2 (*RTX-KG2c*) [23], one of the largest open-source BKGs that integrates knowledge from extensive human-curated and publication-based databases and has been widely used in the Biomedical Data Translator Project [45, 46]. Compared to other commonly used open-source BKGs mentioned above, *RTX-KG2c* is a biolink model–based standardized [47] and regularly updated BKG that efficiently merges biologically and semantically equivalent nodes and edges via multiple curation steps. The version 2.7.3 of *RTX-KG2c* that we use contains around 6.4 million nodes and 39.3 million edges with knowledge from 70 public biomedical sources, where all biological concepts (e.g., “ibuprofen”) are represented as vertices and all concepts–predicates–concept (e.g., “ibuprofen–increases activity of–GP1BA gene”) are presented as edges. For drug repurposing purposes, we customized *RTX-KG2c* with 4 principles (see more details in Supplementary Section S1): (i) excluding the nodes whose categories are irrelevant to drug repurposing explanation (e.g., “GeographicLocation” and “Device”), (ii) filtering out the low-quality edges based on our criteria, (iii) removing the hierarchically redundant edges, and (iv) excluding all drug–disease edges. After these processing steps, 3,659,165 nodes with 33 distinct categories (Fig. 1A) and 18,291,237 edges with 74 distinct types (Fig. 1B) are left in our customized biomedical knowledge graph, which is used for downstream model training.

#### Data sources for model training

To train the KGML-xDTD framework for drug repurposing prediction and its MOA explanation, we utilize 4 high-quality and NLP-derived training datasets:

- **MyChem Data** [48] is provided by the BioThings API collection [49], which contains up-to-date annotations regarding indication and contraindication for chemicals collected from 11 reliable data resources (summarized in Supplementary Section S2). We use drug–disease pairs with the relation “indication” as true positives while those with “contraindication” as true negatives.
- **SemMedDB Data** [50] is provided by the Semantic MEDLINE Database (SemMedDB), which leverages NLP techniques to extract semantic triples with “treats” and “negatively treats” relations from PubMed abstracts. We use drug–disease pairs with the relation “treats” as true positives and those with “negatively treats” as true negatives.
- **NDF-RT Data** [51] is provided by National Drug File–Reference Terminology from the Veterans Health Administration (VHA), which contains FDA-approved information on drug interaction, indications, and contraindications. We use drug–disease with therapeutics label “indications” as true positives and those with “contraindications” as true negatives.
- **RepoDB Data** [52] is a standard set of successful and failed drug–disease pairs in clinical trials collected by the Blavatnik Institute at Harvard Medical School. We use drug–disease with the status “approved” as true positives and those with “terminated” as true negatives.

We further filter drug–disease pairs from SemMedDB Data due to publication bias and possible NLP mistakes by using both the co-occurrence frequency and the PubMed publication-based normalized Google distance (NGD) [53] defined below:


(1) \begin{equation*} NGD(c1, c2) = \frac{max\lbrace log\mathcal {N}(c1), log\mathcal {N}(c2)\rbrace - log\mathcal {N}(c1, c2)}{logN - min\lbrace log\mathcal {N}(c1), log\mathcal {N}(c2)\rbrace } \end{equation*}

where *c*1 and *c*2 are 2 biological concepts used in the customized BKG, $\mathcal {N}(c1)$ and $\mathcal {N}(c2)$ respectively represent the total number of unique PubMed IDs associated with *c*1 and *c*2, $\mathcal {N}(c1, c2)$ is the total number of unique PubMed IDs shared between *c*1 and *c*2, and *N* is the total number of pairs of Medical Subject Heading (MeSH) terms annotations in PubMed database. Only the SemMedDB drug–disease pairs with at least 10 supporting publications and an NGD score of 0.6 or lower are left for the downstream model training.

These datasets are pooled together and then processed by (i) mapping the raw identifiers of drugs and diseases to the identifiers used in the customized BKG and (ii) removing duplicate drug–disease pairs in both the true-positive set and the true-negative set. Table 1 shows the drug–disease pair count from each data source after data preprocessing.

**Table 1:** Pair count of true-positive (indications) and true-negative (contraindications or no effect) data from 4 data sources after data preprocessing

Source	True positive (treats)	True negative (not treat)
MyChem	3,663	26,795
SemMedDB	8,255	11
NDF-RT	3,421	5,119
RepoDB	2,127	738
Shared	3,971	526
**Total**	21,437	33,189

Note that “shared” means those pairs are from 2 or more data sources.

#### DrugMechDB

DrugMechDB [42, 43], to our best knowledge, is the first human-curated path-based database for explaining the MOA from a drug to a disease in an indication, with 3,593 MOA paths for 3,327 unique drug–disease pairs. These paths are extracted from free-text descriptions from DrugBank, Wikipedia, and other literature sources and then have been curated by subject matter experts and also follow the schema of the Biolink model. Hence, we can match them to nodes used in the RTX-KG2 BKG via the *Node Synonymizer* function [23]. Since the maximum length of predicted MOA paths generated by the KGML-xDTD framework is fixed to 3 in this study due to memory and training time constraints, we consider those 3-hop BKG-based paths as “correct” if all 4 of their nodes show up in the complete DrugMechDB-based MOA paths. Thus, we find 472 unique drug–disease pairs of which each has at least 1 such “correct” matched path in all possible 3-hop paths between drug and disease in the customized BKG. The large reduction in evaluation paths is likely due to incompleteness of the underlying knowledge graphs, imperfect bioentity matching, and possibility of disconnected drug and disease pairs in the customized BKG. However, these paths are used for additional, external validation data only. We use the matched paths as true-positive biologically meaningful paths for the evaluation of the model-predicted paths in the task of MOA prediction (introduced below).

### Model framework

**Figure 2:**
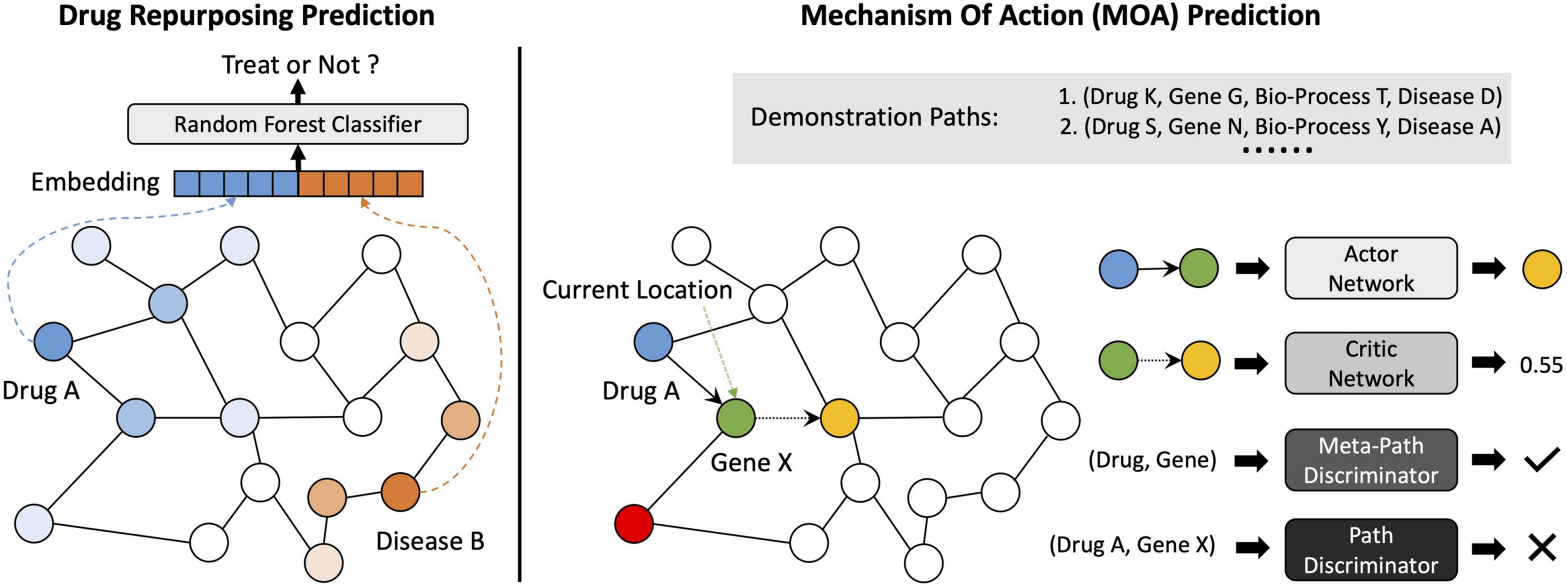
Illustration of the entire KGML-xDTD model framework: DRP module (left) and MOA prediction module (right).

The model framework of KGML-xDTD consists of 2 modules: a drug repurposing prediction (DRP) module that combines the advantages of GraphSAGE [40] and a random forest model, and an MOA prediction module that utilizes an adversarial actor–critic RL model. We show the overview of the entire model framework in Fig. 2. The implementation details of each module in KGML-xDTD framework are presented in Supplementary Section S3.

#### Notations

Let $\mathcal {G} = \lbrace \mathcal {V}, \mathcal {E}\rbrace$ be a directed biomedical knowledge graph, where each node $v \in \mathcal {V}$ represents a biological entity (e.g., a specific drug, disease, gene, or pathway), and each edge $e \in \mathcal {E}$ represents a biomedical relationship (e.g., *interacts-with*; see more in Fig. 1B). We use $\mathcal {V}^{\mathrm{drug}}$ to represent all the drug nodes (the nodes with the categories of “Drug” and “Small Molecule” in the customized BKG) and $\mathcal {V}^{\mathrm{disease}}$ to represent all the disease nodes (the nodes with the categories of “Disease,” “PhenotypicFeature,” “BehavioralFeature,” and “DiseaseOrPhenotypicFeature” in the customized BKG). For each notation, we use bold formatting to represent its embedding (e.g., $\boldsymbol {v}$ represents the embedding of *v* ).

#### DRP module

Drug repurposing aims to identify new indications of existing drugs/compounds. We solve it as a link prediction problem on the graph $\mathcal {G}$. Specifically, given any drug–disease pair (*v_i_*, *v_j_*) where $v_i \in \mathcal {V}^{\mathrm{drug}}$ and $v_j \in \mathcal {V}^{\mathrm{disease}}$, we predict the probability that drug *i* can be used to treat disease *j*. We first use GraphSAGE to calculate the embedding for each node. Ideally, the node embeddings should contain 2 kinds of information: node attributes and node neighborhoods.

To capture the neighborhood information, we optimize GraphSAGE to encourage neighbor nodes to have similar embeddings and nonneighbor nodes to have distinct embeddings. Specifically, we perform random walks for each node to collect its neighborhood information and train the model to maximize a node’s similarity with its neighbor nodes. For a node *u*, the loss is calculated as


(2) \begin{equation*} L_{\mathcal {G}}(\boldsymbol {z}_u) = - \log (\sigma (\boldsymbol {z}_u^{\top }\boldsymbol {z}_v)) - k \cdot \mathbb {E}_{v_n \sim P_n(v)} \log (\sigma (\boldsymbol {z}_u^{\top }\boldsymbol {z}_{v_n})) \end{equation*}

where $\boldsymbol {z}_u, \boldsymbol {z}_v$ are respectively the embeddings of nodes *u, v*, σ is the sigmoid function; *v* is a node that co-occurs with *u* in fixed-length random walks; *P_n_* represents negative sampling distribution; and *k* indicates the number of negative samples (nodes not in *u*’s fixed-length neighborhood).

To capture the node attributes information, we utilize the PubMedBERT model [54], a pretrained language model designed for biomedical texts, to generate a node attribute embedding for each node based on the concatenation of the node’s name and category. We further compress the embeddings to 100 dimensions with principal components analysis (PCA) to reduce memory usage and use them as the initial node feature for GraphSAGE. In this way, the final GraphSAGE embedding of each node should contain the information regarding both graph topology and node attributes. We concatenate the GraphSAGE embeddings of drug–disease pairs and use them as input of a random forest model to classify each drug–disease pair into one of the “not treat,” “treat,” and “unknown” classes. We obtain “treat” and “not treat” drug–disease pairs from 4 data sources (described in “Data sources for model training” section). We generate “unknown” drug–disease pairs through negative sampling [55], that is, replacing the drug or disease identifier in each “treat” drug–disease pair with a random drug or disease identifier to generate a new pair that does not appear in both the “treat” and “not treat” classes. Specifically, for each unique “treat” drug–disease pair, we respectively replace its drug identifier with 1 random drug identifier as well as replace its disease identifier with 1 random disease identifier to make the “unknown” drug–disease pairs.

#### MOA prediction module

When potential indications of a given drug are identified by the drug repurposing prediction module, a natural yet essential question is: can we biologically explain the predictions? We solve this by employing an RL model to predict the BKG-based MOA paths, which are the paths on the knowledge graph from drug nodes to disease nodes. These BKG-based MOA paths can semantically describe an abstract biological process of how a drug treats a disease.

##### Demonstration paths

To encourage the RL agent to terminate the path searching at the expected diseases through a biologically reasonable path, we leverage so-called "demonstration paths", a set of biologically likely paths (e.g., drug1–gene1–protein3–disease1) that explains the underlying reasons for why a drug can treat a disease. We extract 396,705 demonstration paths from the customized BKG using the known drug–target interactions collected from 2 curated biomedical data sources: DrugBank (v5.1) and Molecular Data Provider (v1.2) (see “Data Availability” section), as well as the PubMed publication-based NGD (see Equation 1). We show more details regarding demonstration path extraction in Supplementary Section S4.

##### Adversarial actor–critic reinforcement learning

We formulate the MOA prediction as a path-finding problem and adapt the adversarial actor–critic reinforcement learning model [41] to solve it. Reinforcement learning is defined as a Markov decision process (MDP) that contains:

**States**: Each state *s_t_* at time *t* is defined as *s_t_* = (*v_drug_*, *v_t_*, (*v*_*t* − 1_, *e_t_*), …, (*v*_*t* − *K*_, *e*_*t* − (*K* − 1)_)), where $v_{drug} \in \mathcal {V}^{\mathrm{drug}}$ is a given starting drug node; $v_{t} \in \mathcal {V}$ represents the node where the agent locates at time *t*; and the tuple (*v*_*t* − *K*_, *e*_*t* − (*K* − 1)_) represents the previous *K*th node and (*K* − 1)th predicate. For the initial state *s*_0_, the previous nodes and predicates are substituted by a special dummy node and predicate. We concatenate the embedding of all nodes and predicates of *s_t_* to get the state embedding $\boldsymbol {s}_{t}$, where the node embeddings are node attribute embeddings generated with the PubMedBERT model (see “DRP module” section) and the predicate embeddings employ one-hot vectors.

**Actions**: The action space *A_t_* of each node *v_t_* includes a self-loop action *a_self_* and the actions to reach its outgoing neighbors in the graph $\mathcal {G}$. Due to memory limitation and extremely large out-degree of certain nodes in the knowledge graph, we prune the neighbor actions based on the PageRank scores if a node has more than 3,000 neighbors. Specifically, we let $A_{t}= (a_{self}, a_{1},\dots , a_{k},\dots , a_{n_{v_t}})$, where $n_{v_t}$ is out-degree of node $v_t \in \mathcal {V}$. For each action *a_t_* = (*e_t_*, *v*_*t* + 1_) ∈ *A_t_* taken at time *t*, we concatenate its node and predicate embeddings to obtain action embedding $\boldsymbol {a}_t$. We learn 2 embedding matrices $\boldsymbol{E}^{N_\mathrm{n} \times \mathrm{d}}$ and $\boldsymbol{E}^{N_\mathrm{p} \times \mathrm{d}}$, respectively, for nodes and predicates (note that each subnetwork uses separate embedding matrices), where *d* represents the embedding dimension, *N*_n_ represents the number of nodes in the graph, and *N*_p_ represents the number of predicate categories in the graph.

**Rewards**: During the path-searching process, the agent only receives a terminal reward *R_e, T_* from the environment (i.e., there is no intermediate reward from environment: *R_e, t_* = 0, ∀*t* < *T*). Let *v_T_* be the last node of the path, and $\mathcal {N}_{drug}$ be the known diseases that drug *v_drug_* can treat. The terminal reward *R_e, T_* from environment is calculated with the drug repurposing model via


\begin{equation*} {R_{e,T}=\left\lbrace \begin{array}{ll}1, &\,\, \mbox{if}\ v_T \in \mathcal {N}_{drug}.\\ p_{treat}, &\,\, \mbox{if}\ v_{T} \notin \mathcal {N}_{drug} ; v_{T} \in \mathcal {V}^{\mathrm{disease}}\ \mathrm{and}\ f(v_{drug}, v_{T})\ \mathrm{is\,\,predicted\,\,as\,\,''treat''}.\\ 0, &\,\, \mbox{if}\ v_{T} \notin \mathcal {N}_{drug} ; v_{T} \in \mathcal {V}^{\mathrm{disease}}\ \mathrm{and}\ f(v_{drug}, v_{T})\ \mathrm{is\,\,not\,\,predicted\,\,as\,\,''treat''}.\\ -1, &\,\, \mbox{if}\ v_{T} \notin \mathcal {V}^{\mathrm{disease}}, \end{array} \right.} \end{equation*}

where *p_treat_* is the “treat” class probability predicted by the drug repurposing model *f*.

The adversarial actor–critic RL model consists of 4 subnetworks that share the same model architecture MLP^*i*^ (note that *i* represents the id of each subnetwork described later, such as *a* for *actor network, c* for *critic network*, etc.) but with different parameters:


(3) \begin{equation*} \mathrm{MLP}^{i}(X) = BA(BA(XW^i_{1}+b^i_{1})W^i_{2}+b^i_{2})W^i_{3}+b^i_{3} \end{equation*}

where {${W^i_{1}, W^i_{2}, W^i_{3}, b^i_{1}, b^i_{2}, b^i_{3}}$} are the parameters and biases of linear transformations, and *BA* represents a batch normalization layer followed by an ELU activation function.

**Actor network**: The actor network learns a path-finding policy π_θ_ (note that θ represents all parameters of the actor network) to guide the agent to choose an action *a_t_* from the action space *A_t_* based on the current state *s_t_*:


(4) \begin{equation*} \pi _{\theta }(a_{t}|s_{t}, A_{t}) = \mathrm{softmax}(\boldsymbol {A}_{t} \odot \mathrm{MLP}^{a}(\boldsymbol {s}_t)) \end{equation*}

where $\boldsymbol {A}_{t}$ is the embedding matrix of the action space *A_t_*; ⊙ represents the dot product. Here, π_θ_(*a_t_*|*s_t_*, *A_t_*) represents the probability of choosing action *a_t_* at time *t* from the action space *A_t_* given the state *s_t_*.

**Critic network**: The critic network [56] estimates the expected reward *Q*_ϕ_(*s_t_*, *a_t_*) (note that ϕ represents all parameters of the critic network) if the agent takes the action *a_t_* at the state *s_t_* by


(5) \begin{equation*} Q_{\phi }(s_{t},a_{t}) = \mathrm{MLP}^{c}(\boldsymbol {s}_t) \odot \boldsymbol {a}_{t} \end{equation*}

**Path discriminator network**: Since the RL agent only receives a terminal reward *R_e, T_* from the environment indicating whether it reaches an expected target, to encourage the agent to find biologically reasonable paths and provide intermediate rewards, we further guide it with demonstration paths. This network is essentially a binary classifier that distinguishes whether a path segment (*s_t_*, *a_t_*) is from demonstration paths or generated by the actor network. We treat all the known demonstration path segments $(s_{t}^{D}, a_{t}^{D})$ as positive samples and all actor-generated non-demonstration path segments $(s_{t}^{ND}, a_{t}^{ND})$ as negative samples. The path discriminator network $D_{p}(s,a) = \mathrm{sigmoid}(\mathrm{MLP}^{p}(\boldsymbol {s} \oplus \boldsymbol {a}))$, where $\boldsymbol {s}$ and $\boldsymbol {a}$ are respectively the embeddings of the state *s* and the action *a*; ⊕ represents the concatenation operator and is optimized with


(6) \begin{equation*} L_{p} = - \mathbb {E}_{(s,a) \sim P_{D}}[\log (D_{p}(s,a))] - \mathbb {E}_{(s,a) \sim P_{A}}[\log (1-D_{p}(s,a))]
\end{equation*}

where *P_D_* and *P_A_* respectively represent the demonstration path segment distribution and the actor-generated non-demonstration path segment distribution. Based on the probability *D_p_*(*s_t_*, *a_t_*), the path discriminator–based intermediate reward *R_p, t_* is calculated as


(7) \begin{equation*} R_{p,t} = \log (D_{p}(s_{t},a_{t})) - \log (1-D_{p}(s_{t},a_{t})). \end{equation*}

**Meta-path discriminator network**: Similar to the path discriminator, this network aims to judge whether the meta-path of the actor-generated paths is similar to that of demonstration paths. The meta-path is the path of node categories (e.g., [“Drug”→“Gene”→“BiologicalProcess”→“Disease”]). Similarly, the meta-path discriminator $D_{m}(M) = \mathrm{sigmoid}(\mathrm{MLP}^{m}(\boldsymbol {M}))$, where $\boldsymbol {M}$ is the embedding of the meta-path *M* defined as the concatenation of learned category embeddings of all nodes that appear in the path, is also a binary classifier where the meta-paths of demonstration paths are treated as positive samples while others are negative samples. We optimize it with the following loss:


(8) \begin{equation*} L_{m}= - \mathbb {E}_{M \sim P^M_{D}}[\log (D_{m}(M))] - \mathbb {E}_{M \sim P^M_{A}}[\log (1-D_{m}(M))]
\end{equation*}

where $P^M_{D}$ and $P^M_{A}$ respectively represent the demonstration meta-path distribution and the actor-generated non-demonstration meta-path distribution. The intermediate reward *R_m, t_* generated by the meta-path discriminator is calculated by


(9) \begin{equation*} R_{m,t} = \log (D_{m}(M)) - \log (1-D_{m}(M)). \end{equation*}

The integrated intermediate reward *R_t_* at time *t* is then calculated as


(10) \begin{equation*} R_{t} = {\alpha }_{p}R_{p,t}+{\alpha }_{m}R_{m,t}+(1-{\alpha }_{p}-{\alpha }_{m}){\gamma }^{T-t}R_{e,T} \end{equation*}

where α_*p*_ ∈ [0, 1] and α_*m*_ ∈ [0, 1 − α_*p*_] are hyperparameters, γ is the decay coefficient, and *R_e, T_* is defined in the “Rewards” section above.

To optimize the critic network, we minimize the temporal difference (TD) error [57] with loss:


(11) \begin{equation*} L_{c}= \mathrm{TD}^{2} = [(R_{t} + Q_{\phi }(s_{t+1},a_{t+1})) - Q_{\phi }(s_{t},a_{t})]^{2}. \end{equation*}

Since the goal of the actor network is to achieve the largest expected reward by learning an optimal actor policy, we optimize the actor network by maximizing $J({\theta }) = \mathbb {E}_{a \sim {\pi }_{\theta }}[Q_{\phi }(s_{t},a)]$. We use the REINFORCE algorithm [58] to optimize the parameters. To encourage more diverse exploration in finding paths, we use the entropy of π_θ_ as a regularization term and optimize the actor network with the following stochastic gradient of the loss function *L_a_*:


(12) \begin{equation*} {\nabla }_{\theta }L_{a} = - {\nabla }_{\theta }J({\theta })= - \mathbb {E}_{{\pi }_{\theta }}[{\nabla }_{\theta }\mathrm{TD} \log {\pi }_{\theta }(a_{t}|s_{t})] - {\alpha }{\nabla }_{\theta }\mathrm{entropy}({\pi }_{\theta }) \end{equation*}

where π_θ_ is the action probability distribution based on the actor policy, and α is the entropy weight.

We follow Zhao et al. [41] to train the adversarial actor–critic RL model in a multistage way. First, we initialized the actor network using the behavior cloning method [59] in which the training set of demonstration paths is used to guide the sampling of the agent with mean square error (MSE) loss. Then, in the first *z* epochs, we freeze the parameters of the actor network and the critic network and respectively train the path discriminator network and meta-path discriminator network by minimizing *L_p_* and *L_m_*. After *z* epochs, we unfreeze the actor network and the critic network and optimize them together by minimizing a joint loss *L_joint_* = *L_a_* + *L_c_*.

## Results

### Evaluation settings

#### Data split

The post-processed drug–disease pairs (described in “Data sources for model training” section) are split into training, validation, and test sets where the drug–disease pairs of each unique drug are randomly split according to a ratio of 8/1/1. For example, let’s say drugA has 10 known diseases that it treats (e.g., drugA–disease1, …, drugA–disease10), 8 pairs are randomly split into the training set, 1 pair is to the validation set, and 1 pair to the test set. With this data split method, the model can be exposed to every drug in the training set, which complies with our goal of predicting new indications of known drugs and their potential MOAs based on the MOA of known target diseases.

#### Evaluation metrics

The proposed framework KGML-xDTD is evaluated on 2 types of tasks: ***predicting drug–disease “treat” probability*** (i.e., drug repurposing prediction) as well as ***identifying biologically reasonable MOA paths from all BKG-based path candidates*** (i.e., MOA prediction). These 2 tasks are evaluated based on classification accuracy-based metrics (e.g., accuracy, macro F1 score) and ranking-based metrics (e.g., mean percentile rank, mean reciprocal rank, and proportion of ranks smaller than K) defined as follows:

**Accuracy (ACC)** is the fraction of the model classification is correct, computed as


(13) \begin{equation*} \mbox{ACC} = \frac{\mbox{Number of correct classifications}}{\mbox{Total number of drug-disease pair classifications}}. \end{equation*}

**Macro F1 score (Macro-F1)** is the unweighted mean of all the per-class F1 scores:


(14) \begin{equation*} F1^{c} = 2*\frac{precision^{c} \times recall^{c}}{precision^{c} + recall^{c}} , \mbox{Macro-F1} = \frac{1}{|C|} \sum _{c \in C}{F1^{c}} \end{equation*}

where *C* presents classification classes (e.g., “treat,” “not treat,” and “unknown”).

**Mean percentile rank (MPR)** is the average percentile rank of the 3-hop DrugMechDB-matched BKG-based path (described in “DrugMechDB” section) of true-positive drug–disease pairs:


(15) \begin{equation*} \mbox{MPR} = \frac{1}{|PR|} \sum _{pr \in PR}{pr} \end{equation*}

where *PR* is a list of percentile ranks of DrugMechDB-matched BKG-based paths of true-positive drug–disease pairs (“treat” category).

**Mean reciprocal rank (MRR)** is the average inverse rank of true-positive drug–disease pairs (“treat” category) or their 3-hop DrugMechDB-matched BKG-based paths:


(16) \begin{equation*} \mbox{MRR} = \frac{1}{|R|} \sum _{r \in R}{\frac{1}{r}} \end{equation*}

where *R* is a list of ranks of true-positive drug–disease pairs (for DRP task) or DrugMechDB-matched BKG-based paths (for MOA prediction task).

**Hit@K** is the proportion of ranks not larger than K for true-positive drug–disease pairs (“treat” category) or their 3-hop DrugMechDB-matched BKG-based paths:


(17) \begin{equation*} \mbox{Hit@K} = \frac{1}{|R|} \sum _{r \in R}{|r \le K|} \end{equation*}

where *R* is a list of ranks of true-positive drug–disease pairs (for DRP task) or DrugMechDB-matched BKG-based paths (for MOA prediction task).

#### Drug repurposing prediction evaluation method

We utilize the metrics *ACC* and *Macro-F1* to measure the accuracy of drug repurposing prediction of our KGML-xDTD framework while using ranking-based metrics *MRR* and *Hit@K* to show its capability in reducing false positives (i.e., the false drug–disease pairs ranking higher among possible drug–disease candidates). We use the following 3 methods to generate non-true-positive drug–disease candidates for each true-positive drug–disease pair to calculate the ranks that are employed in the *MRR* and *Hit@K* calculation:

- **Drug rank–based replacement**: For each true positive drug–disease pair, the drug rank–based replacement pairs are generated by replacing the drug entity with each of all 274,676 other drugs in the customized BKG while excluding all known true-positive drug–disease pairs.
- **Disease rank–based replacement**: For each true-positive drug–disease pair, the disease rank–based replacement pairs are generated by replacing the disease entity with each of all 124,638 other diseases in the BKG while excluding all known true-positive drug–disease pairs.
- **Combined replacement:** For each true-positive drug–disease pair, the combined replacement pairs are the combination of all replacement pairs of the above 2 methods. All known true-positive drug–disease pairs are excluded from these replacement pairs.

Due to the massive size of possible drug–disease candidates, some baseline models (e.g., GAT and GraphSAGE+SVM) are not applicable in this setting within a reasonable time (e.g., a week). Thus, we also employ a small subset of drug–disease replacements to calculate the *MRR* and *Hit@K*, allowing for comparison between KGML-xDTD with all baselines. Specifically, we utilize 1,000 random drug–disease pairs from the combined replacement set above: 500 with drug ID replacement and 500 with disease ID replacement. To enhance the robustness of results obtained through this random replacement method, we use this method to generate 10 sets of random drug–disease pairs (each with 1,000 pairs) independently and calculate the mean and standard deviation of the ranking-based metrics outcomes. In addition, since the drug repurposing prediction module of KGML-xDTD framework does 3-class classification while other baselines do 2-class classification, for a fair comparison, we recalculate *ACC* and *Macro-F1* for KGML-xDTD by excluding the “unknown” class.

#### MOA prediction evaluation method

For the evaluation of MOA prediction, we use the DrugMechDB [42] to obtain the expert-verified MOA paths as ground-truth data, and match each biological concept in these verified MOA paths to the biological entities used in the customized BKG, and then generate the BKG-based matched paths of DrugMechDB drug–disease pairs (described in “DrugMechDB” section), which are considered biologically meaningful MOA paths. We first calculate the path scores for all 3-hop KG paths between drug and disease with the path-finding policy learned from adversarial actor–critic RL model using the following equation:


(18) \begin{equation*} \mbox{path score} = \sum _{i=1}^{k}{{\delta }^{i-1} \times \log (P_{i} \times N_{i})} \end{equation*}

where *k* is the number of hops in this path, δ is a decay coefficient (we set it to 0.9 in this study), *P_i_* represents the probability of choosing action *a_i_* in the $i^{th}$ hop following this path based on the trained RL model, and *N_i_* is the number of possible actions in the $i^{th}$ hop.

With the path scores, we obtain the ranks of the DrugMechDB-matched BKG-based paths and calculate their ranking-based metrics (e.g., *MPR, MRR*, and *Hit@K*). For those drug–disease pairs with multiple matched paths, we use the highest ranks of the matched paths as their ranks in the metrics calculation. We compare KGML-xDTD with the baseline models based on these metrics to show the capability of the MOA prediction module of KGML-xDTD in identifying biologically reasonable MOA paths from a massive and complex BKG with a comparably low false positive. In addition, we further perform 2 case studies to evaluate the effectiveness of KGML-xDTD in identifying the biologically reasonable MOA paths.

### Drug repurposing prediction evaluation

For drug repurposing prediction evaluation, we compare the KGML-xDTD model framework against several state-of-the-art (SOTA) KG-based models and variants of KGML-xDTD for drug repurposing prediction based on the method described in “Drug repurposing prediction evaluation method” section.

We use 8 different SOTA KG-based models as baseline models that are commonly used for BKG-based drug repurposing [19, 60]. TransE [25], TransR [61], and RotatE [26] are the translation distance–based models that regard a relation (e.g., “treats”) as a “translation”/”rotation” (e.g., a kind of spatial transformation) from a head entity (e.g., a drug node) to a tail entity (e.g., a disease node). DistMult [27] is a bilinear model that measures the latent semantic similarity of a knowledge graph triple (head entity, relation/predicate, tail entity) with a trilinear dot product. ComplEx [28] and ANALOGY [62] are the extensions of DistMult that consider more complex relations (e.g., asymmetric relations). SimpLE [63] is a tensor factorization–based model to learn the semantic relation of a knowledge graph triple. GAT [64] is a popular graph neural model that leverages the important graph topology structure based on self-attention mechanism for graph-associated tasks (e.g., link prediction). Implementation details of these baselines are presented in Supplementary Section S5.

Besides these SOTA baseline models, we also compare the drug repurposing prediction module in KGML-xDTD with its several variants to show the effectiveness of model components. For example, to show efficacy of the combination of GraphSage and random forest (RF), we use a pure GraphSAGE model for link prediction (GraphSAGE-link), the combination of GraphSage and logistic model (GraphSAGE-logistic), and the combination of GraphSage and support vector machine (SVM) model (GraphSAGE-SVM). To demonstrate the effectiveness of node attribute embeddings (described in “DRP module” section) in improving repurposing prediction, we conduct an ablation experiment that replaces node attribute embeddings (NAEs) with random embeddings (initialized with the Xavier method [65]) as GraphSage initialized embeddings (KGML-xDTD w/o NAE); to support rationality of setting “unknown” class through negative sampling (described in “DRP module” section), we modify the drug repurposing prediction module for 2-class classification (i.e., true positive and true negative) (2-class KGML-xDTD) as a baseline comparison model.

**Figure 3:**
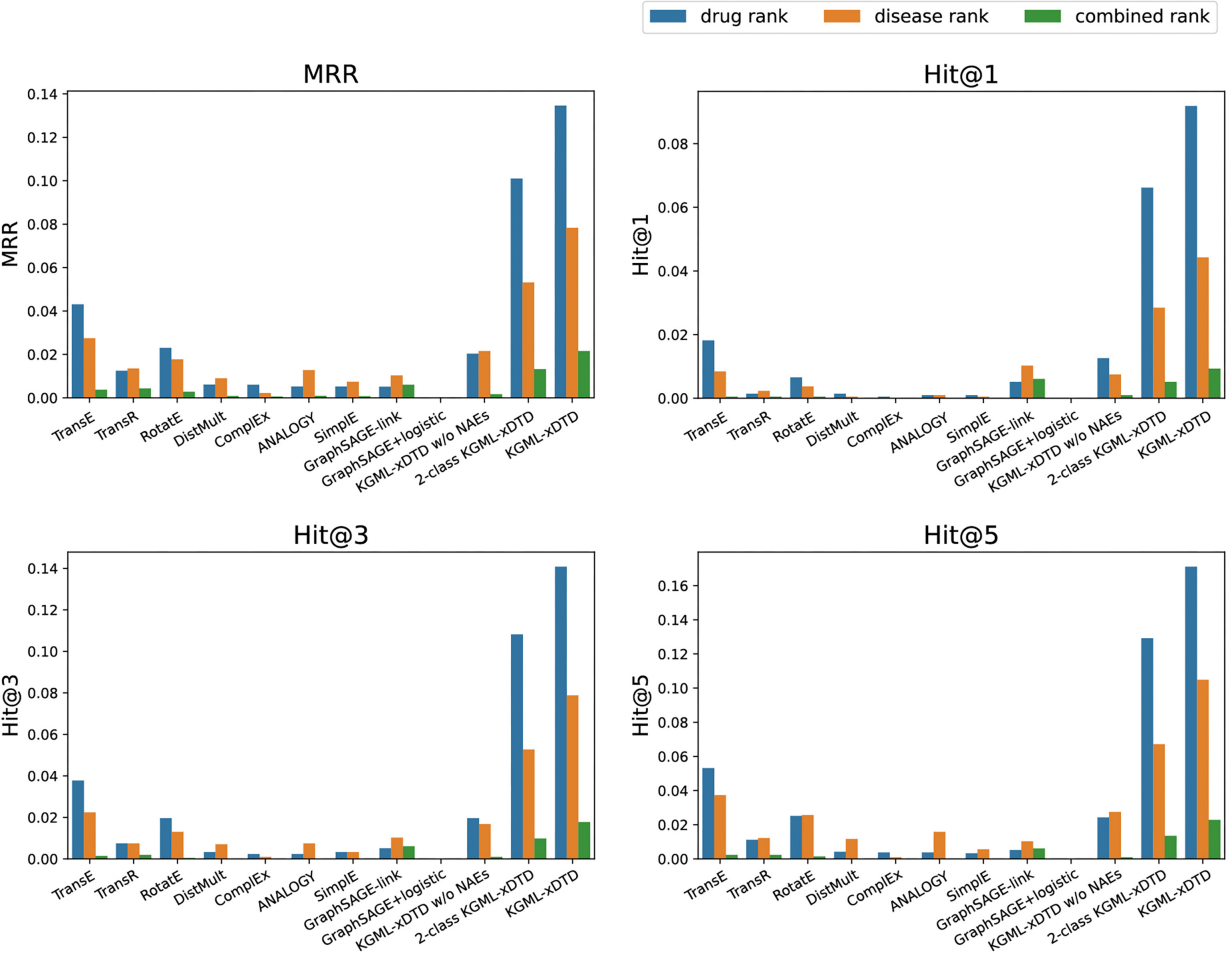
The performance comparison of DRP between KGML-xDTD and different baseline models (GAT and GraphSAGE+SVM are excluded due to computation time constraints) based on test set using 3 “complete” replacement methods (i.e., “drug rank–based replacement,” “disease rank–based replacement,” and “combined replacement” described in “Drug repurposing prediction evaluation method” section) to generate non-true-positive drug–disease candidates for each true-positive drug–disease pair for MRR and Hit@K calculation. “Drug rank”, “disease rank,” and “combined rank” respectively correspond to the methods of “drug rank–based replacement,” “disease rank–based replacement,” and “combined replacement.”

Table 2 shows the performance of the KGML-xDTD model and all other baseline models in the task of drug repurposing prediction based on test set (described in “Data split” section). For the calculation of the *MRR* and *Hit@K* used in this table, we utilize the random subset replacement method described in the “Drug repurposing prediction evaluation method” section. As shown in the table, on the one hand, the KGML-xDTD outperforms most of the baseline models and achieves comparable performance as GAT in classification-based metrics (e.g., accuracy, macro F1 score), indicating its effectiveness in classifying known “treat” and “not treat” drug–disease pairs with both attribute and neighborhood information on the knowledge graph. On the other hand, KGML-xDTD’s exceptional performance in ranking-based metrics shows its superiority over baselines in identifying new indications of existing drugs out of a large number of possible drug–disease pairs with relatively low false positives, which is of great importance for guiding clinical research. Fig. 3 displays the comparison results where we calculate the *MRR* and *Hit@K* with 3 different “complete” replacement methods (described in “Drug repurposing prediction evaluation method” section). Although GAT and GraphSAGE+SVM are excluded in this comparison due to computation time constraints, we can see that the results presented in both Table 2 and Fig. 3 are consistent to demonstrate KGML-xDTD’s ability in reducing false positives. Therefore, excluding the GAT and GraphSAGE+SVM from the comparison with “complete” replacement methods does not affect the conclusion. Besides, by comparing 2-class KGML-xDTD with the vanilla GraphSAGE model (e.g., GraphSAGE-link), we demonstrate the effectiveness of the random forest model over a neural network classifier in this task. The comparison between KGML-xDTD w/o NAE and KGML-xDTD shows that the KGML-xDTD benefits from the use of node attribute embeddings for drug repurposing prediction while the comparison with 2-class KGML-xDTD indicates the effectiveness of using negative sampling to generate “unknown” drug–disease pairs for model training. With the “unknown” drug–disease pairs, the KGML-xDTD model achieves significant improvement in ranking-based metrics, which is essential when applying to real-world drug repurposing because it can reduce the false positives.

**Table 2:** The performance comparison of drug repurposing prediction (DRP) between KGML-xDTD and different baseline models based on test set (described in “Data split” section). The top panel shows the performance of state-of-the-art (SOTA) baseline models; the middle panel shows the performance of variants of the KGML-xDTD model framework; the bottom panel shows the performance of KGML-xDTD model framework

Model	Accuracy	Macro F1 score	MRR	Hit@1	Hit@3	Hit@5
TransE	0.708	0.708	0.301 (±0.005)	0.134 (±0.007)	0.327 (±0.009)	0.482 (±0.007)
TransR	0.858	0.855	0.329 (±0.006)	0.150 (±0.009)	0.378 (±0.008)	0.542 (±0.005)
RotatE	0.704	0.704	0.281 (±0.007)	0.098 (±0.008)	0.314 (±0.007)	0.497 (±0.009)
DistMult	0.555	0.495	0.182 (±0.004)	0.042 (±0.002)	0.157 (±0.010)	0.292 (±0.010)
ComplEx	0.624	0.460	0.138 (±0.004)	0.026 (±0.004)	0.106 (±0.007)	0.205 (±0.008)
ANALOGY	0.594	0.465	0.188 (±0.004)	0.044 (±0.004)	0.165 (±0.009)	0.301 (±0.008)
SimplE	0.599	0.472	0.167 (±0.006)	0.036 (±0.006)	0.140 (±0.008)	0.259 (±0.011)
GAT	**0.936**	**0.934**	0.002 (±0.000)	0.000 (±0.000)	0.000 (±0.000)	0.000 (±0.000)
GraphSAGE-link	0.919	0.915	0.002 (±0.000)	0.000 (±0.000)	0.000 (±0.000)	0.000 (±0.000)
GraphSAGE+logistic	0.791	0.784	0.002 (±0.000)	0.000 (±0.000)	0.000 (±0.000)	0.000 (±0.000)
GraphSAGE+SVM	0.807	0.793	0.002 (±0.000)	0.000 (±0.000)	0.000 (±0.000)	0.000 (±0.000)
KGML-xDTD w/o NAEs	0.909 (0.898*)	0.891 (0.892*)	0.159 (±0.003)	0.035 (±0.002)	0.143 (±0.006)	0.262 (±0.008)
2-class KGML-xDTD	0.929	0.925	0.278 (±0.003)	0.183 (±0.006)	0.321 (±0.003)	0.389 (±0.006)
KGML-xDTD (ours)	0.935 (0.930*)	0.923 (0.926*)	**0.382 (±0.004)**	**0.238 (±0.007)**	**0.425 (±0.006)**	**0.543 (±0.006)**

The values with * inside the parentheses are the adjusted results by excluding the “unknown” category for a fair comparison.

The ranking metrics (e.g., “MRR” and “Hit@K”) are calculated as the mean along with standard deviation based on 10 independent sets of non-true-positive drug–disease candidates generated by the random drug–disease replacement method (i.e., for each true-positive drug–disease pair in test set, we use 1,000 random drug–disease pairs as non-true-positive drug–disease candidates to calculate the rank). See more details in “Drug repurposing prediction evaluation method” section.

The abbreviation “w/o NAEs” in the name of model “KGML-xDTD w/o NAEs” represents without using node attribute embeddings.

### MOA prediction evaluation

For MOA prediction, we evaluate how well the KGML-xDTD can identify the DrugMechDB-matched BKG-based MOA paths (described in “MOA prediction evaluation method” section) from a large number of possible paths in the customized BKG by utilizing ranking-based metrics (e.g., MPR, MRR, and Hit@K) and 2 specific case studies.

There are few machine learning models designed for the task of identifying biologically meaningful paths from biomedical knowledge graphs for explaining drug repurposing. Although the UKGE [30], GrEDeL [32], and Polo [38] models (all mentioned in “Introduction”) were proposed and can be used for this goal, they all have certain constraints and cannot be used as baseline models for comparison. The UKGE model cannot be applied to BKGs without weighted edge information (e.g., frequency of relation appeared in literature). The authors of the GrEDeL model do not provide the code to implement this model. The Polo model cannot be trained within a reasonable time (e.g., within 2 weeks) on a massive and complex BKG (e.g., RTX-KG2) due to its dependence on a computationally inefficient method “DWPC” [39]. Therefore, we choose the MultiHop reinforcement learning model [66] as a baseline model since it uses a similar LSTM model framework as the GrEDel model and allows using a self-defined reward-shaping strategy in its reward function as what we do in the KGML-xDTD model (i.e., we can use the same reward strategy described in “Adversarial actor–critic reinforcement learning” section). Furthermore, we also compare with an ablated version of KGML-xDTD (i.e., KGML-xDTD w/o DP, which does not take advantage of the demonstration paths by setting α_*p*_ and α_*m*_ in Function 9 as 0) as another baseline model to show the importance of proposed demonstration paths.

We compare the MOA prediction performance between the KGML-xDTD model framework and different baseline models in Table 3. Although all the models use the same terminal reward function from the environment (i.e., the drug repurposing prediction module of KGML-xDTD), the MOA prediction module of KGML-xDTD achieves significantly better performance in identifying DrugMechDB-matched BKG-based MOA paths than the other 2 baselines across all ranking-based metrics. Comparison between the KGML-xDTD with and without demonstration paths (i.e., KGML-xDTD w/o DP) further illustrates the great effectiveness of using proposed demonstration paths to guide the path-finding process. Due to the massive searching space and sparse rewards, the RL agent often fails to find biologically reasonable BKG-based MOA paths out of many possible choices, while our model KGML-xDTD, with the intermediate guidance provided by the demonstration path, is able to identify those biologically reasonable choices with a much higher probability. Moreover, comparing KGML-xDTD w/o DP and MultiHop reveals that the actor–critic model structure performs similarly to LSTM. However, incorporating the proposed demonstration paths can significantly enhance the effectiveness of the actor–critic model structure over LSTM for this task.

**Table 3:** The performance comparison of MOA prediction between KGML-xDTD and different baseline models (e.g., MultiHop and KGML-xDTD w/o DP) based on the test set (described in “Data split” section). The metrics in this table are calculated using path scores and all non-DrugMechDB-matched 3-hop paths between drug and disease as “negative” paths for each true-positive drug–disease pair (see more details in “MOA prediction evaluation method” section)

Model	MPR	MRR	Hit@1	Hit@10	Hit@50	Hit@100	Hit@500
MultiHop	61.400%	0.027	0.017	0.042	0.067	0.118	0.345
KGML-xDTD w/o DP	72.965%	0.015	0.008	0.017	0.067	0.160	0.403
KGML-xDTD (ours)	**94.696%**	**0.109**	**0.059**	**0.193**	**0.496**	**0.613**	**0.849**

The abbreviation “w/o DP” in the name of model “KKGML-xDTD w/o DP” represents “without using demonstration paths.”

To further evaluate the performance of KGML-xDTD model framework in identifying biologically relevant MOA paths for drug repurposing, we present 2 different case studies to explore the potential repurposed drugs and their potential mechanism for 2 rare genetic diseases: hemophilia B and Huntington’s disease.

#### Case 1: Hemophilia B

**Figure 4:**
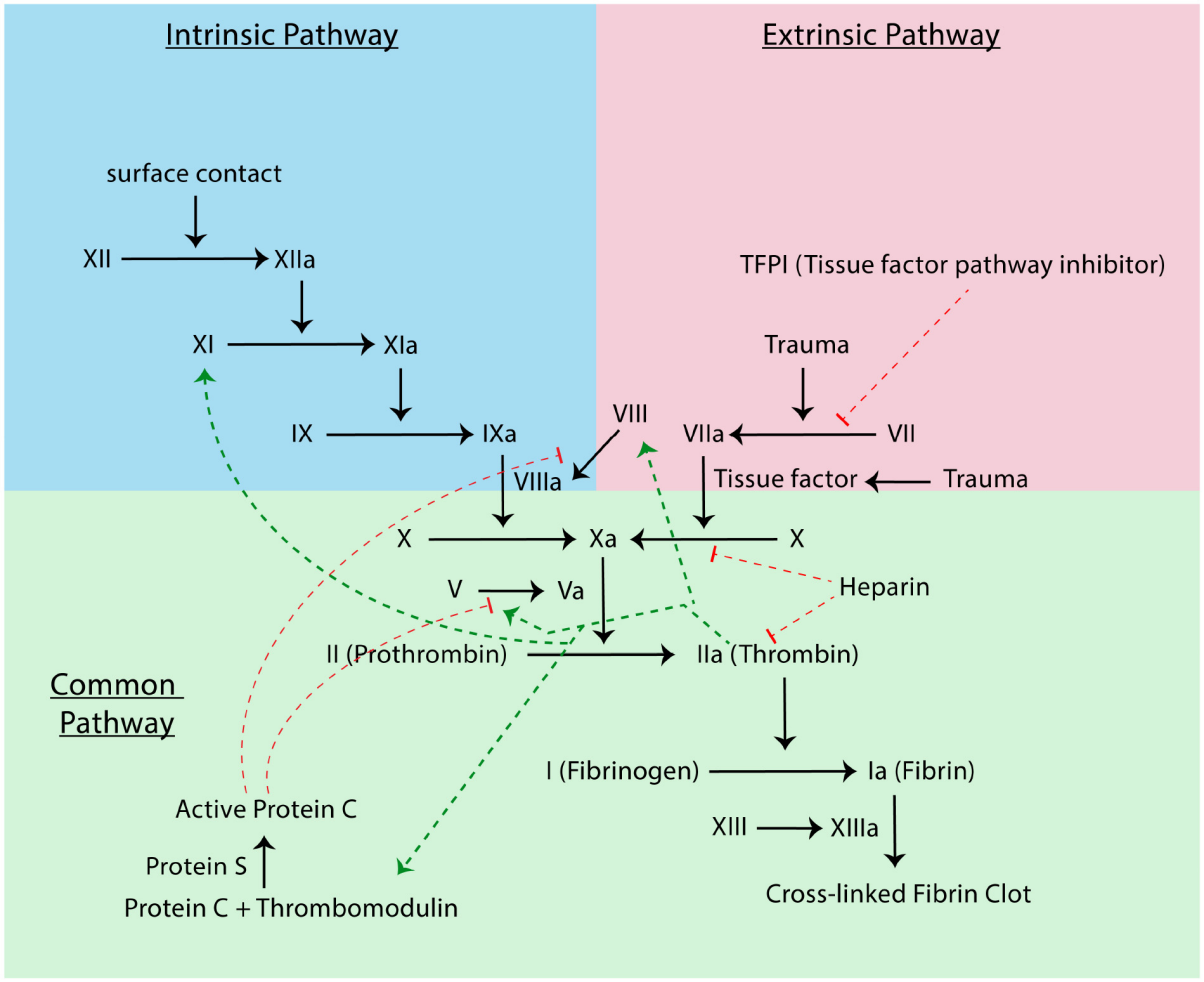
Blood coagulation regulatory network with arrows for molecular reactions (black), positive feedback (green), and negative feedback (red).

Hemophilia B, also known as factor IX deficiency or Christmas disease, is a rare genetic disorder that results in prolonged bleeding in patients. It is caused by mutations in the factor IX (F9) gene, which is located on the X chromosome. Table 4 displays the top 10 drugs/treatments predicted by the KGML-xDTD model framework, including both those that are used in the training set (red bolded) and those that are not. Besides those known drugs/treatments used in the training set, the majority of the remaining 7 drugs/treatments on the list are supported by published research and have the potential to treat hemophilia B. For example, the activated human-derived coagulation factor VII (i.e., factor VIIa) or the recombinant activated factor VII (i.e., rFVIIa) is one of the proteins that can cause blood clots as an important part of the blood coagulation regulatory network (as shown in the Fig. 4). This protein is used as an effective inhibitor in the treatment of patients with hemophilia B [67, 71]. Thrombin is a key enzyme in the maintenance of normal hemostatic function. It has been reported that using thrombin as a therapeutic strategy can help prevent bleeding in patients with hemophilia [72]. The use of recombinant factor IX therapy is a recommended treatment option for individuals with hemophilia B [73]. Some examples of recombinant factor IX products include BeneFIX, Rixubis, Ixinity, Alprolix Idelvion, and Rebinyn. These examples demonstrate the potential capability of KGML-xDTD for drug repurposing in real-world applications.

**Table 4:** Top 10 predicted drugs/treatments for hemophilia B (note that the red bolded drugs are used in the training set)

Drug/Treatment	Prob.	Publications
**Eptacog Alfa (rFVIIa)**	**0.833**	[67, 68]
**Nonacog Alfa (rFIX)**	**0.803**	[69]
**Viral vector**	**0.780**	[70]
Factor VIIa	0.748	[67, 71]
Recombinant FVIIa (rFVIIa)	0.724	[67, 71]
Thrombin	0.709	[72]
Factor IX	0.708	[73]
Epicriptine	0.702	
Hyperbaric oxygen	0.660	
Triamcinolone	0.649	

**Figure 5:**
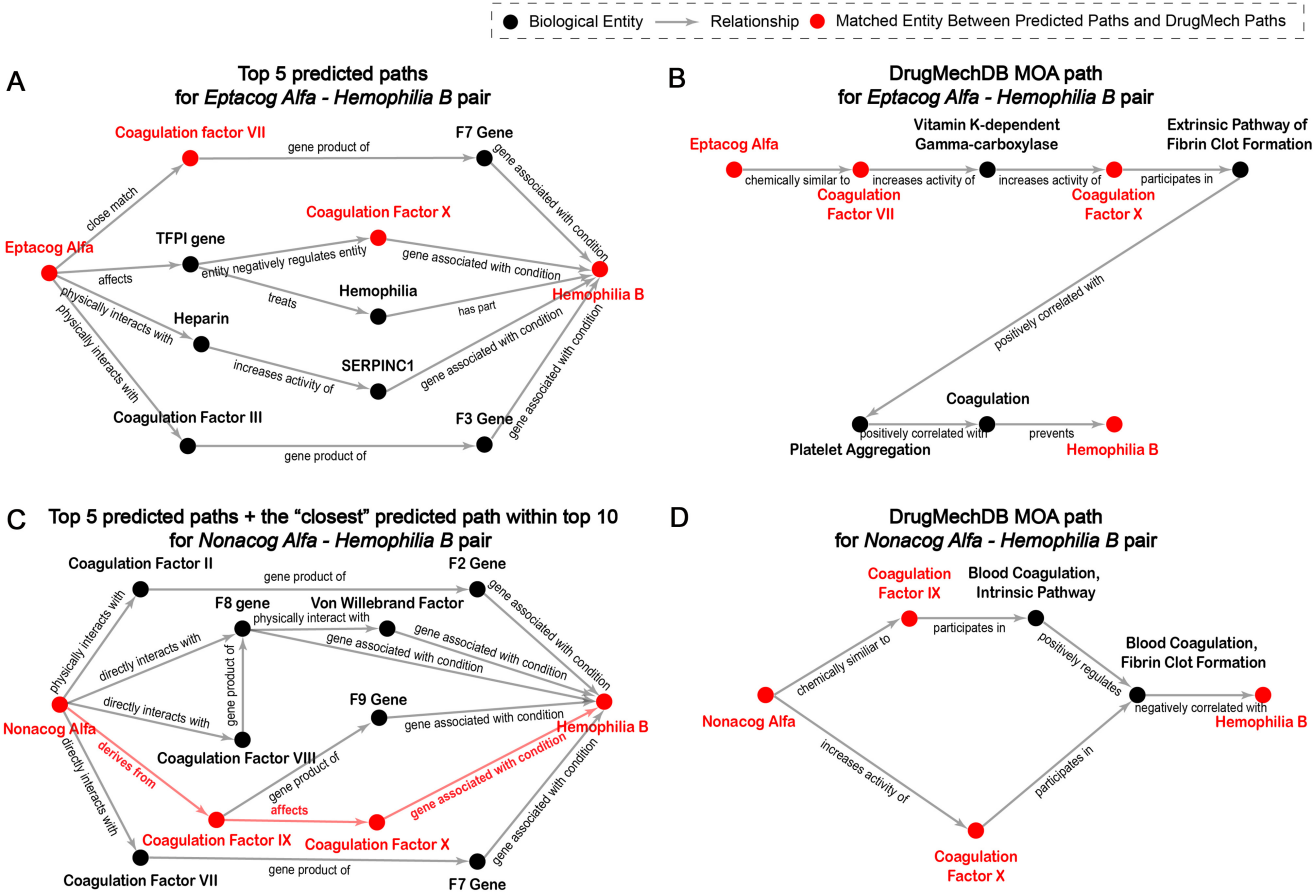
Comparison between the top 5 predicted 3-hop paths (including any available DrugMechDB-matched BKG-based paths in the top 10 predicted paths, highlighted in red) and the curated DrugMechDB-based MOA paths for Eptacog Alfa and Nonacog Alfa. Note that the RTX-KG2 paths and DrugMechDB paths might use different synonyms for the same biological concept. For better visualization and illustration, we utilize consistent entity synonyms between the predicted paths and the curated paths as well as present the predicted paths in a graph structure. (A, C) Graph representation of predicted paths generated by KGML-xDTD respectively for Eptacog Alfa and Nonacog Alfa. (B, D) Human-curated DrugMechDB MOA paths.

To further assess the biological explanations of the predicted 3-hop BKG-based MOA paths for the treatment of hemophilia B, we have used the curated DrugMechDB-based MOA paths, which are not used in the model training process. DrugMechDB contains relevant MOA paths of hemophilia B treatment only for Eptacog Alfa and Nonacog Alfa. We use the KGML-xDTD model to predict the top 10 potential 3-hop BKG-based MOA paths for these 2 drugs and compare them with the curated DugMechDB-based MOA paths in Fig. 5 (for visualization purpose, we only display the top 5 predicted paths along with any available DrugMechDB-matched BKG-based paths in the top 10 predicted paths). The corresponding biological entities between the predicted paths and the curated DrugMechDB-based paths are highlighted in red. Although the predicted paths cannot exactly match the DrugMechDB-based MOA paths due to the limited path length and some missing semantic relationships in the customized biomedical knowledge graph, key biological entities (such as coagulation factor VII, coagulation factor X, and coagulation factor IX) that are important for the treatment of hemophilia B are present in the predicted paths. As shown in Fig. 4, the treatment of hemophilia B involves a complex molecular network of blood coagulation, and many of the coagulation factors (such as factor VII, factor III, factor II, factor VIII, factor IX, and factor X) present in the predicted paths are also part of this molecular network. In Supplementary Section S6, we also utilize the KGML-xDTD model framework to predict the top 10 three-hop BKG-based paths, which can serve as biological explanations of the predicted “treats” relationship between factor VIIa and hemophilia B (shown in Table 4). This particular drug/treatment–disease pair is not included in the training set and thus can be used to indicate how KGML-xDTD’s MOA path predictions can contribute to the explanation of the predicted drug repurposing results. The predicted paths show molecular details akin to those in Fig. 4 for treating hemophilia B. As a result, the predicted paths by KGML-xDTD model framework can help identify key molecules in the real drug action regulatory network, thereby aiding in explaining drug repurposing to some extent.

#### Case 2: Huntington’s disease

**Figure 6:**
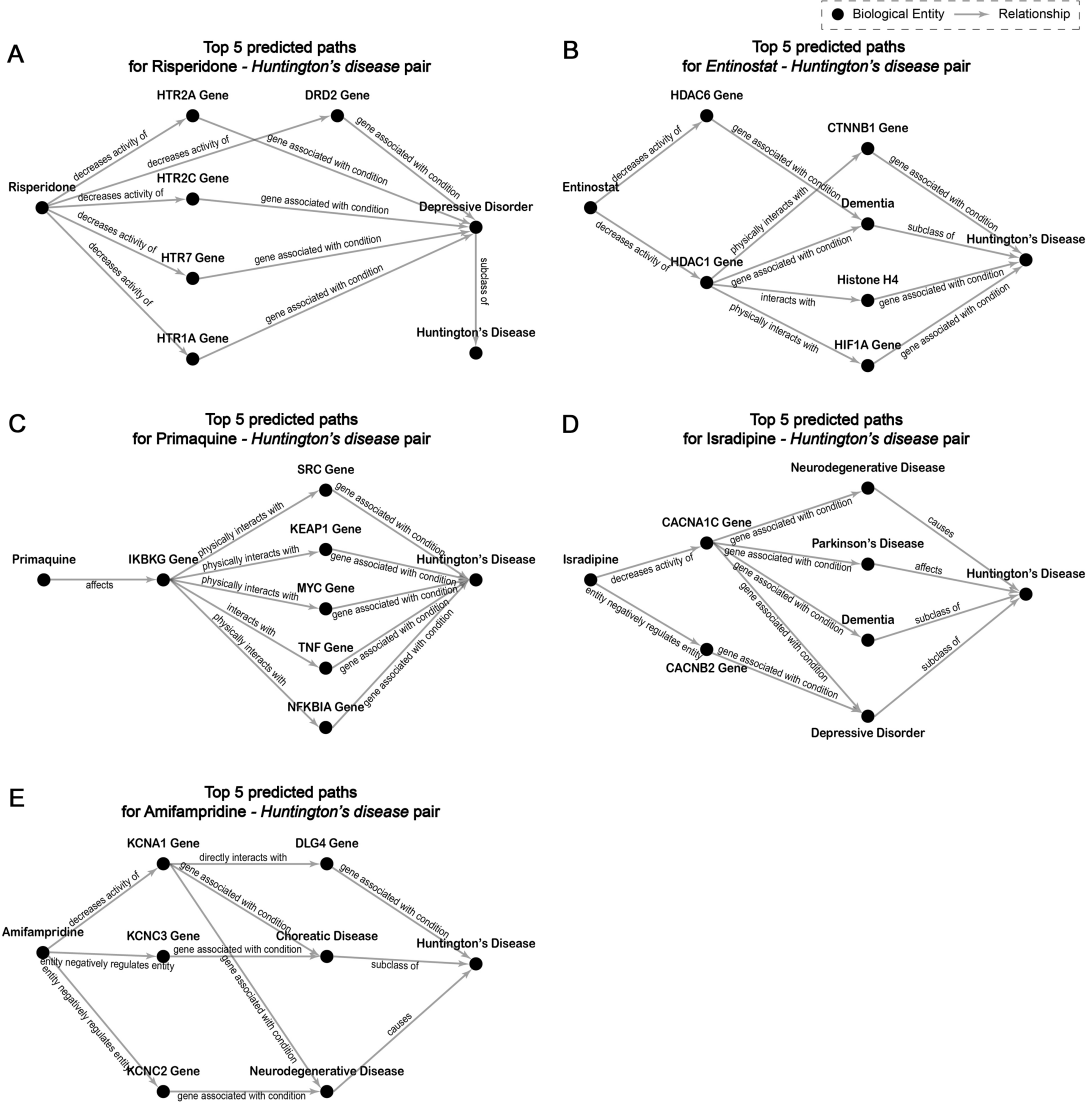
Top 5 predicted 3-hop BKG-based MOA paths (integrated into a graph for better visualization) for top 5 nonchemotherapeutic predicted drugs/treatments that are not included in the training set for Huntington’s disease.

Huntington’s disease (HD) is a rare neurogenetic disorder that typically occurs in midlife with symptoms of depression, uncontrolled movements, and cognitive decline. While there is currently no drug/treatment that can alter the course of HD, some drugs/treatments can be useful for the treatment of its symptoms in abnormal movements (e.g., chorea) and psychiatric phenotypes. We show 10 drugs/treatments with the highest predicted probability by the KGML-xDTD model framework after manual processing in Table 5. This processing involves excluding the chemotherapeutic drugs from the predicted drug candidate list due to their potential risk of cytotoxicity to normal cells (which could lead to false positives for drug repurposing of noncancer diseases [74, 75]), and only presenting the top 5 results in the training set and top 5 from the test or validation set. From this table, it can be observed that many of the top-ranked predicted drugs have been supported by publications as potential treatments for the symptoms of HD. Since there is currently no effective treatment for HD, DrugMechDB does not have a corresponding MOA path for comparison. To analyze the predicted paths by the KGML-xDTD model framework for the predicted nonchemotherapeutic drugs/treatments that are not included in the training set (shown in regular text in Table 5), we present their top 5 predicted paths in Fig. 6. From these predicted paths, we can see that most of them are biologically relevant. For example, Fig. 6A shows that risperidone is predicted to be useful for the treatment of HD by decreasing the activity of the genes associated with the 5-hydroxytryptamine receptor (e.g., HTR1A, HTR2A, HTR2C, HTR7) and dopamine receptor (e.g., DRD2), which have been proven to be involved in the pathogenesis of depressive disorders [76, 77]. The presence of depressive symptoms is a significant characteristic of HD [78]. Entinostat is predicted to have the potential to alleviate the symptoms of HD by inhibiting the functions of histone deacetylase genes such as HDAC1 and HDAC6 (see Fig. 6B), and one of the predicted 3-hop BKG-based MOA paths (“Entinostat” → “decreases activity of” → “HDAC1 gene” → “interacts with” → “Histone H4” → “gene associated with condition” → “Huntington’s disease”) is supported by the previous research [79, 80]. Primaquine is predicted to act on the IKBKG gene to potentially play a therapeutic role in neurodegenerative disease (see Fig. 6C), reported in [81]. According to the predicted BKG-based MOA paths (see Fig. 6D), isradipine may have a potential therapeutic effect for HD by mainly regulating the genes of the calcium voltage-gated channel, including CACNA1C and CACNB2. These genes may be associated with the symptoms of HD, such as depression and dementia [82]. Lastly, amifampridine is predicted to regulate the genes of the potassium voltage-gated channel (see Fig. 6E), which are potentially associated with HD [83]. All these examples indicate that the predicted BKG-based MOA paths can explain the mechanism of repurposed drugs to some extent.

**Table 5:** Top 5 predicted drugs/treatments used in the training set (highlighted in red bold) and the top 5 nonchemotherapeutic predicted drugs/treatments that are not in the training set for Huntington’s disease

Drug/Treatment	Prob.	Publications
**Pimozide**	**0.939**	[84, 85]
**Therapeutic agent**	**0.939**	
**Olanzapine**	**0.938**	[86, 87]
**Riluzole**	**0.935**	[88]
**Antipsychotic agent**	**0.932**	[89]
Risperidone	0.893	[78, 90]
Entinostat	0.888	[79]
Primaquine	0.887	
Isradipine	0.884	[91]
Amifampridine	0.882	

## Drug Class Analysis

**Figure 7:**
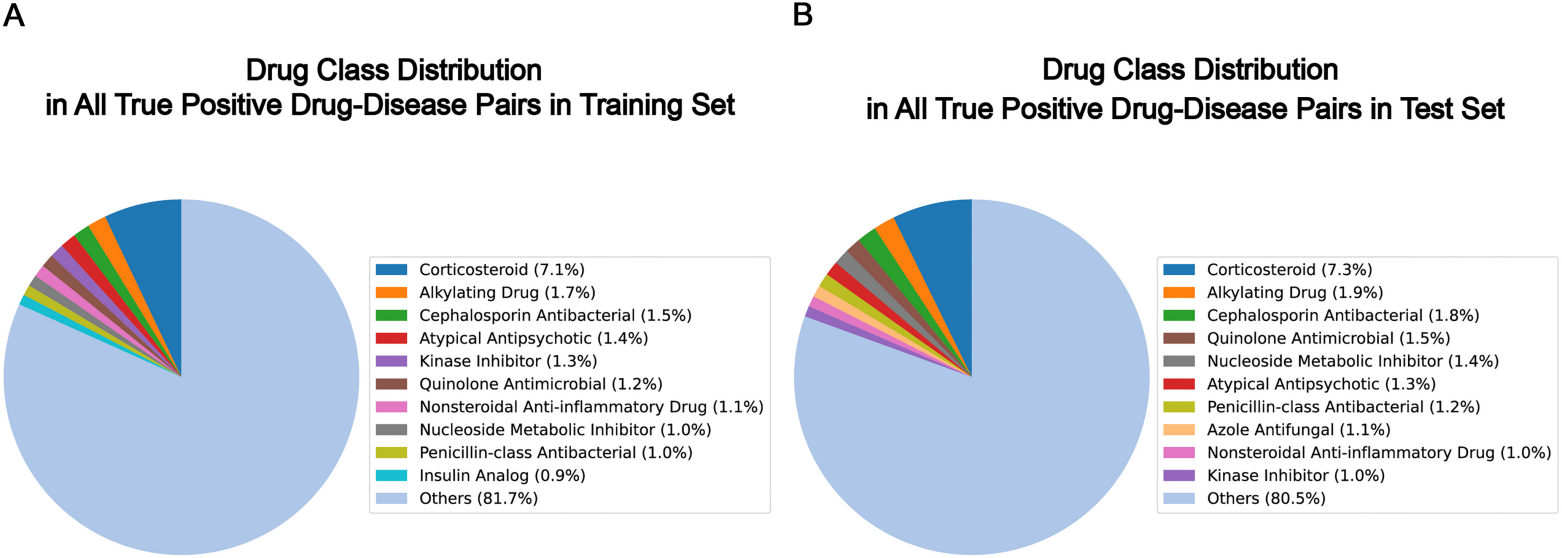
Comparison of the drug class distribution in true-positive drug–disease pairs between the training set and test set. The drug class of each drug/chemical in these pairs is determined based on the FDA “Established Pharmacologic Class” (EPC) accessed via MyChem.info APIs. There are 2,238 drug classes represented in the true-positive drug–disease pairs in the training set and 718 drug classes in the test set. For visualization purposes, we only show the top 10 drug classes in each set and the rest is classified into the “Others” class.

The drug repurposing prediction of the KGML-xDTD model does not leverage any information regarding drug similarity such as drug classes, SMILES, drug side effects, and drug-related gene profiles/sequences, and we find that the distribution of drug classes in true-positive drug–disease pairs is similar between the training and test sets (see Fig. 7). In this section, we examine whether our model can only predict the drugs with the drug classes that it has seen in the training set.

**Figure 8:**
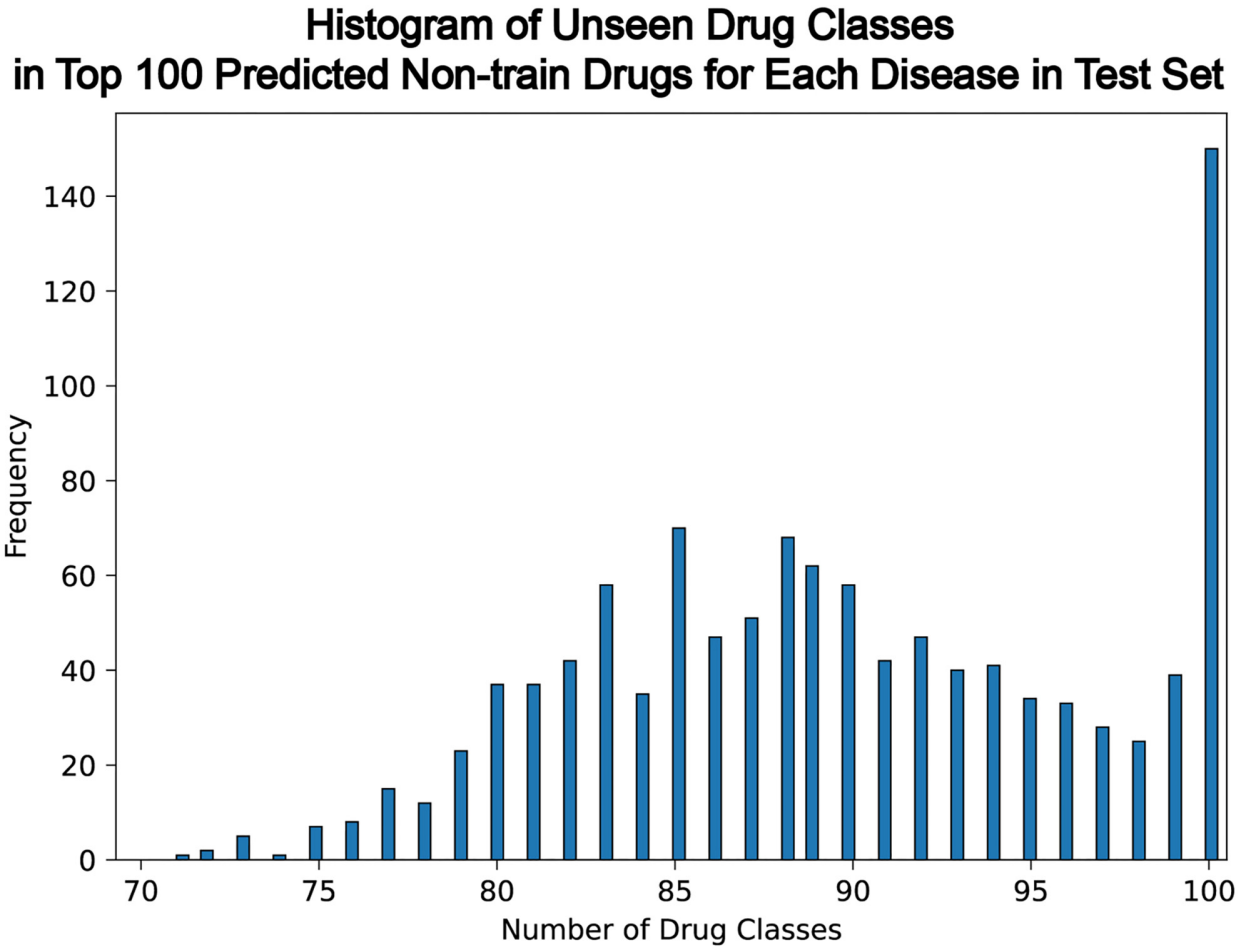
Distribution of unseen drug classes in the top 100 predicted nontrain drugs across the 1,140 diseases in the test set.

To do this, we use the MyChem.info APIs [48] to retrieve the FDA’s “Established Pharmacologic Class” (EPC) information for chemicals/drugs using their synonym identifiers. For the FDA-unapproved chemical/drug without such EPC information, we consider it as as a single class. We first utilize the KGML-xDTD model to predict the top 100 chemicals/drugs for each of the 1,140 diseases in the test set (described in “Data split” section) after excluding the drug–disease pairs presented in the training set. Then we count the number of drug classes among these 100 predicted drugs that are not seen in the training set for each disease. Fig. 8 shows the distribution of unseen drug classes in the top 100 predicted nontrain drugs across the 1,140 diseases in the test set. We can see that each disease has at least 70 different drug classes among the top 100 predicted drugs, indicating that the predictive power of the KGML-xDTD model is derived from the node attribute information and knowledge graph topology structure rather than any drug class information.

## Discussion

In this work, we propose KGML-xDTD, a 2-module, knowledge graph–based machine learning framework that not only predicts the treatment probabilities between drugs/compounds and diseases but also provides biological explanations for these predictions through the predicted paths in a massive biomedical knowledge graph with comprehensive biomedical data sources as potential mechanisms of action. This framework can assist medical researchers in quickly identifying the potential drug/compound–disease pairs that might have a treatment relationship, which can accelerate the process of drug discovery for emerging diseases. Additionally, by leveraging the KG-based MOA paths predicted by the framework, medical professionals (e.g., doctors and licensed medical practitioners) can straightforwardly assess the accuracy of the predictions, which can help to reduce false positives that may be produced by the “black-box” operation of traditional machine learning models.

Although previous research [15, 19, 24] has applied a variety of models to the task of drug repurposing using BKGs, these approaches are implemented in the small-scale BKGs, and many do not scale to larger and more complex graphs as biotechnology advances and the volume of data in biomedical databases increases. In our comparison with state-of-the-art KG-based models for drug repurposing, we find that the KGML-xDTD model had higher accuracy with lower false positives when applied to a massive and complex biomedical knowledge graph *RTX-KG2c*. By evaluating the predicted paths with DrugMechDB and 2 case studies, we show that the model can capture some key biological entities involved in real drug action regulatory networks.

It is widely acknowledged that drug repurposing is one of the most challenging problems in biomedicine, and current artificial intelligence (AI) techniques are still in the early stages of addressing it. Many other AI models, such as those based on chemical structure, drug–target interactions, and drug perturbations of gene expression, are developed for solving this goal. They may offer more accurate predictions but also have limitations in terms of cost and the availability of samples for specific diseases. BKG-based machine learning models, such as the KGML-xDTD model, offer a cost- and time-efficient alternative due to the large volume of biomedical knowledge stored in public databases and publications. The KGML-xDTD model framework is not intended to replace or beat these models but rather provides a complementary approach that leverages emerging knowledge graphs for drug repurposing.

Future work to further enhance the KGML-xDTD model framework might include extending the predicted paths for more specific explanations and considering the negative drug–disease pairs so that the model can explain why certain drugs are harmful to diseases.

## Availability of Source Code and Requirements

Project name: KGML-xDTD

- Project homepage: https://github.com/chunyuma/KGML-xDTD
- Operating system(s): Linux (Ubuntu)
- Resource usage in training step: A Linux (Ubuntu) system with at least 8 CPU cores, 800 GB of VRAM, and a 48 GB GPU card (48 GB Quadro RTX 8000 GPU card used in our training)
- Resource usage in inference step: A Linux (Ubuntu) system with at least 8 CPU cores and 50 GB of VRAM. The GPU card is not necessary, but if used, the GPU card needs at least 24 GB VRAM (48 GB Quadro RTX 8000 GPU card used in our inference)
- Time requirement: Based on our hardware performance and parameter settings (please see the scripts on GitHub), the training step takes approximately 2 weeks while the inference step takes approximately 25.42 seconds for 1 drug–disease pair with 3,320 potential paths. These time estimates may vary depending on the hardware performance, parameter settings, and the number of potential paths of a given drug–disease pair.
- Programming language: Shell Script (Bash) with Python 3.8.12
- Other requirements: Python 3.8.12 with GPU/CPU support (GraphSAGE training needs Python 2.7), neo4j-community 3.5.26, miniconda 4.8.2 (please see more requirements in the yaml files under “envs” folder on Github repository)
- Licenses: MIT license, DrugBank academic license, Apache 2.0 license, UMLS Metathesaurus license, CC-BY 4.0 license
- Research Resource Identifier (#RRID): SCR_023678

## Supplementary Material

giad057_GIGA-D-23-00026_Original_Submission

giad057_GIGA-D-23-00026_Revision_1

giad057_GIGA-D-23-00026_Revision_2

giad057_GIGA-D-23-00026_Revision_3

giad057_Response_to_Reviewer_Comments_Original_Submission

giad057_Response_to_Reviewer_Comments_Revision_1

giad057_Response_to_Reviewer_Comments_Revision_2

giad057_Reviewer_1_Report_Original_Submission Yuansheng Liu -- 3/3/2023 Reviewed

giad057_Reviewer_1_Report_Revision_1 Yuansheng Liu -- 5/18/2023 Reviewed

giad057_Reviewer_2_Report_Original_Submission Michel Dumontier -- 4/2/2023 Reviewed

giad057_Supplemental_Files

## Data Availability

The data sets supporting the results of this article are publicly available in the Zenodo repository [92]. All supporting data and materials are available in the *GigaScience* GigaDB database [93]. **Molecular data provider**: A knowledge-centric data provider for systems chemical biology, as part of the NCATS Biomedical Data Translator (“Translator”). See more in https://github.com/NCATSTranslator/Translator-All/wiki/Molecular-Data-Provider [94].

## Additional Files

**Supplementary Section S1**. Biomedical Knowledge Graph RTX-KG2c Preprocessing.

**Supplementary Section S2**. Summary of Data Resources Used by MyChem Data.

**Supplementary Section S3**. Implementation Details of KGML-xDTD Model Framework.

**Supplementary Section S4**. Implementation Details of Demonstration Path Extraction.

**Supplementary Section S5**. Implementation Details of Baseline Models.

**Supplementary Section S6**. Top 10 KGML-xDTD’s Predicted Paths Serving as Biological Explanations for the Predicted “Treats” Relationship between Factor VIIa and Hemophilia B.

**Supplementary Table S1**. Eleven data resources used by MyChem data.

**Supplementary Table S2**. Hyperparameters used for baseline models.

**Supplementary Fig. S1**. Top 10 predicted 3-hop paths (integrated into a graph for better visualization) generated by the KGML-xDTD model framework serve as biological explanations of the predicted “treats” relationship between factor VIIa and hemophilia B. The path highlighted with red is the one in which all nodes can align with the key molecules in the real drug action regulatory network shown in Fig. 4 in the main text.

## Abbreviations

ACC: accuracy; ADAC: adversarial actor–critic; BKG: biomedical knowledge graph; DWPC: degree-weighted path count; EHR: electronic health record; FDA: Food and Drug Administration; GRL: graph reinforcement learning; GWAS: Genome-wide association studies; KG: knowledge graph; LSTM: long short-term memory; Macro-F1: macro F1 score; MDP: Markov decision process; MeSH: Medical Subject Heading; MOA: mechanism of action; MPR: mean percentile rank; MSE: mean square error; NAE: node attribute embedding; NGD: normalized Google distance; NLP: Natural Language Processing; PCA: principal components analysis; RF: random forest; RL: reinforcement learning; RNN: recurrent neural network; RTX-KG2: Reasoning Tool X Knowledge Graph 2; RTX-KG2c: canonicalized version of the Reasoning Tool X Knowledge Graph 2; SemMedDB: Semantic MEDLINE Database; SMILES: Simplified Molecular Input Line Entry System; SVM: support vector machine; VHA: Veterans Health Administration.
