# Supplementary material for: KGML-xDTD: a knowledge graph–based machine learning framework for drug treatment prediction and mechanism description
Source: Gigascience. 2023 Aug 21;12:giad057. doi: 10.1093/gigascience/giad057 (PMC10441000; doi:10.1093/gigascience/giad057)
Supplement: giad057_GIGA-D-23-00026_Original_Submission [file giad057_giga-d-23-00026_original_submission.pdf]

GigaScience

KGML-xDTD: A Knowledge Graph-based Machine Learning Framework for Drug Treatment Prediction and Mechanism Description

--Manuscript Draft--

**Manuscript Number:**	GIGA-D-23-00026
**Full Title:**	KGML-xDTD: A Knowledge Graph-based Machine Learning Framework for Drug Treatment Prediction and Mechanism Description
**Article Type:**	Research
**Funding Information:**	National Center for Advancing Translational Sciences (OT2-TR003428-01S3) — Dr. David Koslicki<br>National Center for Advancing Translational Sciences (OT2-TR003428-01S2) — Dr. David Koslicki<br>National Center for Advancing Translational Sciences (OT2-TR003428-01) — Dr. David Koslicki<br>U.S. National Library of Medicine (R01LM1372201) — Dr. Han Liu<br>National Science Foundation (CAREER-1841569) — Dr. Han Liu<br>National Science Foundation (TRIPODS-1740735) — Dr. Han Liu
**Abstract:**	Background: Computational drug repurposing is a cost- and time-efficient approach that aims to identify new therapeutic targets or diseases (indications) of existing drugs/compounds. It is especially critical for emerging and/or orphan diseases due to its cheaper investment and shorter research cycle compared with traditional wet-lab drug discovery approaches. However, the underlying mechanisms of action (MOAs) between repurposed drugs and their target diseases remain largely unknown, which is still a main obstacle for computational drug repurposing methods to be widely adopted in clinical settings.<br>Results: In this work, we propose KGML-xDTD: a Knowledge Graph-based Machine Learning framework for explainably predicting Drugs Treating Diseases. It is a two-module framework that not only predicts the treatment probabilities between drugs/compounds and diseases but also biologically explains them via knowledge graph (KG) path-based, testable mechanisms of action (MOAs). We leverage knowledge-and-publication based information to extract biologically meaningful "demonstration paths" as the intermediate guidance in the Graph-based Reinforcement Learning (GRL) path-finding process. Comprehensive experiments and case study analyses show that the proposed framework can achieve state-of-the-art performance in both predictions of drug repurposing and recapitulation of human-curated drug MOA paths.<br>Conclusions: KGML-xDTD is the first model framework that can offer KG-path explanations for drug repurposing predictions by leveraging the combination of prediction outcomes and existing biological knowledge and publications. We believe it can effectively reduce "black-box" concerns and increase prediction confidence for drug repurposing based on predicted path-based explanations, and further accelerate the process of drug discovery for emerging diseases.
**Corresponding Author:**	Chunyu Ma<br>Pennsylvania State University - Main Campus: The Pennsylvania State University - University Park Campus<br>State College, PA UNITED STATES
**Corresponding Author Secondary Information:**	
**Corresponding Author's Institution:**	Pennsylvania State University - Main Campus: The Pennsylvania State University - University Park Campus
**Corresponding Author's Secondary**	

Powered by Editorial Manager® and ProduXion Manager® from Aries Systems Corporation

**Institution:**	
**First Author:**	Chunyu Ma
**First Author Secondary Information:**	
**Order of Authors:**	Chunyu Ma
	Zhihan Zhou
	Han Liu
	David Koslicki
**Order of Authors Secondary Information:**	
**Additional Information:**	
**Question**	**Response**
Are you submitting this manuscript to a special series or article collection?	No
**Experimental design and statistics**<br><br>Full details of the experimental design and statistical methods used should be given in the Methods section, as detailed in our Minimum Standards Reporting Checklist. Information essential to interpreting the data presented should be made available in the figure legends.<br><br>Have you included all the information requested in your manuscript?	Yes
**Resources**<br><br>A description of all resources used, including antibodies, cell lines, animals and software tools, with enough information to allow them to be uniquely identified, should be included in the Methods section. Authors are strongly encouraged to cite Research Resource Identifiers (RRIDs) for antibodies, model organisms and tools, where possible.<br><br>Have you included the information requested as detailed in our Minimum Standards Reporting Checklist?	Yes
**Availability of data and materials**	Yes

All datasets and code on which the conclusions of the paper rely must be either included in your submission or deposited in publicly available repositories (where available and ethically appropriate), referencing such data using a unique identifier in the references and in the "Availability of Data and Materials" section of your manuscript.<br><br>Have you have met the above requirement as detailed in our Minimum Standards Reporting Checklist?	

Powered by Editorial Manager® and ProduXion Manager® from Aries Systems Corporation

Main Text
Click here to access/download;Manuscript;KGML-xDTD_GigaScience_submission.pdf

OXFORD (GIGA)^n SCIENCE

GigaScience, 2017, 1–15

doi: xx.xxxx/xxxx
Manuscript in Preparation
research

RESEARCH

KGML-xDTD: A Knowledge Graph-based Machine Learning Framework for Drug Treatment Prediction and Mechanism Description

Chunyu Ma[1, *], Zhihan Zhou[2], Han Liu[2] and David Koslicki[1,3,4,*]

[1]Huck Institutes of Life Sciences, Pennsylvania State University, State College, PA 16801, USA and [2]Department of Computer Science, Northwestern University, Evanston, IL 60208, USA and [3]Department of Computer Science and Engineering, Pennsylvania State University, State College, PA 16801, USA and [4]Department of Biology, Pennsylvania State University, State College, PA 16801, USA

[*]Correspondence address. Chunyu Ma, E-mail: cqm5886@psu.edu; David Koslicki, E-mail: dmk333@psu.edu

Abstract

Background: Computational drug repurposing is a cost- and time-efficient approach that aims to identify new therapeutic targets or diseases (indications) of existing drugs/compounds. It is especially critical for emerging and/or orphan diseases due to its cheaper investment and shorter research cycle compared with traditional wet-lab drug discovery approaches. However, the underlying mechanisms of action (MOAs) between repurposed drugs and their target diseases remain largely unknown, which is still a main obstacle for computational drug repurposing methods to be widely adopted in clinical settings.
Results: In this work, we propose `KGML-xDTD`: a **K**nowledge **G**raph-based **M**achine **L**earning framework for e**x**plainably predicting **D**rugs **T**reating **D**iseases. It is a two-module framework that not only predicts the treatment probabilities between drugs/compounds and diseases but also biologically explains them via knowledge graph (KG) path-based, testable mechanisms of action (MOAs). We leverage knowledge-and-publication based information to extract biologically meaningful "demonstration paths" as the intermediate guidance in the Graph-based Reinforcement Learning (GRL) path-finding process. Comprehensive experiments and case study analyses show that the proposed framework can achieve state-of-the-art performance in both predictions of drug repurposing and recapitulation of human-curated drug MOA paths.
Conclusions: `KGML-xDTD` is the first model framework that can offer KG-path explanations for drug repurposing predictions by leveraging the combination of prediction outcomes and existing biological knowledge and publications. We believe it can effectively reduce "black-box" concerns and increase prediction confidence for drug repurposing based on predicted path-based explanations, and further accelerate the process of drug discovery for emerging diseases.

Key words: Drug Repurposing, Reinforcement Learning, Biomedical Knowledge Graph

Introduction

Traditional drug development is a time-consuming process (from initial chemical identification to clinical trials and finally to FDA approval) that takes around 10-15 years and also comes along with billions-of-dollars investments and high failure rates [1]. Considering the rapid pace of novel disease evolution, it is urgent to find a more efficient and economical drug discovery method. Fortunately, it has been observed that a single drug can often be effective in treating multiple diseases. For example, thalidomide was originally used as an anti-anxiety medication [2], and was later found to have the anti-cancer potential for the treatment of cancers [3, 4]. Hence, drug repurposing, also known as the identification of new uses for existing drugs/compounds, might bring us hope to address this urgent need with the advantage of a shorter research cycle, lower development cost, and more preexisting safety tests.

Compiled on: January 30, 2023.
Draft manuscript prepared by the author.

Existing drug repurposing approaches can roughly be categorized into experimental-based approaches (e.g., binding affinity assays [5], phenotypic screening [6]), clinical-based approaches (e.g., off-label drug use analysis [7]), and computational-based approaches (e.g., chemical-structure-based [8], and GWAS-based approaches [9]). Compared with the former two approaches, the computational approaches are more cost- and time-efficient, particularly when the goal is to prioritize a large number of target drugs/compounds for follow-up experimental investigation. Among all computational drug repurposing methods, the integration of multiple biomedical data sources into a so-called **biomedical knowledge graph** (BKG) for drug discovery has become popular in recent year [10] due to the increasing availability of curated biomedical databases such as DrugBank [11], ChEMBL [12], HMDB [13] and the advancement of semantic web techniques [14]. There are three types of existing BKGs: database-based BKGs, literature-based BKGs, and mixed BKGs. The database-based BKGs (e.g., *Hetionet* [15], *BioKG* [16], *CBKH* [17]) are constructed by integrating biomedical data and their relations stored in existing biological databases. The literature-based BKGs (e.g., *GNBR* [18]) are built by leveraging Natural Language Processing (NLP) techniques to extract semantic information from a large amount of available biomedical literature and electronic health record (EHR) data, which are mostly disease-specific [19, 20, 21]. The mixed BKGs (e.g., *CKG* [22], *RTX-KG2* [23]) are generated by combining the knowledge sources from the above two methods.

Based on these BKGs, several machine learning methods have been proposed or implemented for drug repurposing prediction by treating it as a link prediction task in the BKGs. For example, Himmelstein et al. [15] used the so-called degree-weighted path count (DWPC) to assess the prevalence of 1,206 metapaths and then classified drug-disease treatment relations by fitting these DWPC features to a logistic regression model. Ioannidis et al. [24] proposed a novel graph neural network model I-RGCN to learn the node and relation embeddings for the Covid-19 drug repurposing task. Zhang et al. [19] recently predicted the possible drugs for Covid-19 with five existing popular knowledge graph completion methods (e.g. TransE [25], RotatE [26], DistMult [27], ComplEx [28], and STELP [29]). Although some of these models have shown good performance in drug repurposing prediction on the small-scale BKGs, none have been scaled to massive BKGs with more than millions of nodes and edges and make a comprehensive comparison. More importantly, most of them lack the biological explanatory ability for their predictions, which limits their applicability in clinical research.

Currently, there are few computational models designed for drug repurposing *explanations*. A common and intuitive explanation based on a biomedical knowledge graph for drug repurposing leverages the semantic BKG-based paths between given drug-disease pairs. Sosa et al. [30] applied a graph embedding model UKGE [31], which utilizes the weighted (the frequency of relation appeared in literature) relation edges in a literature-based KG *GNBR*, to identify new indications of drugs for rare diseases and then explain the results via the highest-ranking paths based on confidence scores. However, this method is only applicable in the literature-based BKGs with the weighted edge information. Most BKGs using database-based knowledge don't contain such information. Sang et al. [32] proposed GrEDeL that combines the TransE embedding method with a Long Short-Term Memory (LSTM) Recurrent Neural Network (RNN) model to predict drug-disease relation. By using the embeddings of BKG-paths as model input for predictions, they can provide path-based explanations. However, they claimed that the effectiveness of the approach relies heavily on the NLP tool SemRep, which is reported to have high false positives in named entity recognition [33]. Also, they didn't fully evaluate how biologically reasonable their predicted path-based mechanisms of action (MOAs) are.

Besides the existing methods above, we view reinforcement learning (RL) as a promising solution for drug repurposing explanation. RL models solve the decision-making problem, in which an agent learns how to take appropriate actions to maximize cumulative rewards through interactions with the environment. RL has achieved widespread success in various domains, including games, recommendation systems, healthcare, transportation, etc [34]. Graph Reinforcement Learning (GRL), first proposed around in 2017, aims to solve graph mining tasks such as link prediction [35], adversarial attacks [36], and relational reasoning [37]. Unlike its applications in other domains, one of the biggest challenges in GRL is finding an appropriate reward to guide the path searching in specific domains. To address the issue of finding biologically reasonable BKG-based paths for drug repurposing, it is crucial to incorporate biomedical domain knowledge to guide the path-finding process. Liu et al. [38] developed an RL-based model "PoLo" that utilizes the biological meta-paths identified in Himmelstein et al. [15] via the "DWPC" method to supervise path searching for drug repurposing. However, the "PoLo" model does not scale to a massive and complex BKG (e.g., CKG and RTX-KG2) due to its dependence on the "DWPC" method that is reported to be computationally inefficient [39].

In this article, we describe `KGML-xDTD`: a **K**nowledge **G**raph-based **M**achine **L**earning framework for e**x**plainably predicting **D**rugs **T**reating **D**iseases, which contains two modules for both drug repurposing prediction and explanation. We propose to amplify the ability of RL model in biologically meaningful path searching by utilizing the biologically meaningful "demonstration paths" and pre-trained drug-repurposing model probability as rewards. We incorporate this idea into the appropriate models (e.g., GraphSAGE [40], Random Forest, and ADAC RL [41] models) and then make them applicable to the explainable drug repurposing problem at massive data scale and complexity. By comparing with the existing popular drug repurposing models and evaluating the predicted paths with an expert-curated path-based drug MOA database *DrugMechDB* [42], we show that the proposed model framework can achieve state-of-the-art performance in both predictions of drug repurposing and recapitulation of human-curated drug MOA paths provided by DrugMechDB. In further case studies, by comparing the model predictions with the real regulatory networks, we show that the proposed framework effectively identifies biologically reasonable BKG-based paths for real-world applications.

Materials and Methods

Datasets

Customized Biomedical Knowledge Graph

To accommodate biomedical-reasonable predictions of drugs' indications and their mechanisms of action, the ideal biomedical knowledge graph should integrate biomedical knowledge from comprehensive and diverse databases and publications, as well as accurately identify and merge different identifiers representing the same biological entity into one (For example, "CHEBI:2367" and "CHEMBL455626" are two distinct identifiers separately presented in ChEBI database [43] and ChEMBL database [12] but represent the same compound "abyssinone I"). Thus, we utilize the canonicalized version of the Reasoning Tool X Knowledge Graph 2 (*RTX-KG2c*) [23], one of the largest open-source biomedical knowledge graph (BKG) that integrates knowledge from extensive human-curated and Publication-based databases, and has been widely used in the Biomedical Data Translator Project [44, 45]. Compared to other commonly used open-source BKGs mentioned above, *RTX-KG2c* is a biolink-model-based [1] standardized [46] and regularly-updated

1 The biolink model [46] is a universal and standardized BKG ontology framework

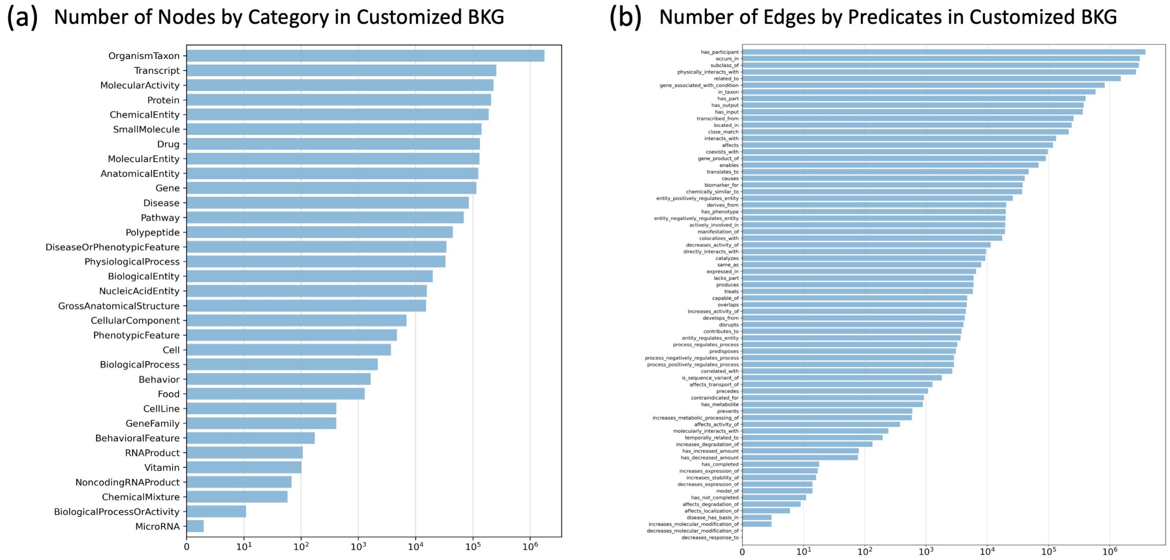

Figure 1. Number of nodes by category (a) and number of edges by predicate (b) in customized Biomedical Knowledge Graph (BKG).

BKG that efficiently merges biologically and semantically equivalent nodes and edges via multiple curation steps. The version 2.7.3 of *RTX-KG2c* that we use contains around 6.4M nodes and 39.3M edges with knowledge from 70 public biomedical sources, where all biological concepts (e.g., "ibuprofen") are represented as vertices and all concept-predicate-concept (e.g., "ibuprofen - increases activity of - GP1BA gene") are presented as edges. For drug repurposing purposes, we customized *RTX-KG2c* with four principles (Please see more details in Supplementary Section S1): 1). excluding the nodes whose categories are irrelevant to drug repurposing explanation (e.g., "GeographicLocation" and "Device"); 2). filtering out the low-quality edges based on our criteria; 3). removing the hierarchically redundant edges; 4). excluding all drug-disease edges. After these processing steps, 3,659,165 nodes with 33 distinct categories (Figure 1 a) and 18,291,237 edges with 74 distinct types (Figure 1 b) are left in our customized biomedical knowledge graph, which is used for downstream model training.

Data Sources for Model Training

To train the `KGML-xDTD` framework for drug repurposing prediction and its explanation, we utilize four human-curated and NLP-derived training datasets:

- **MyChem Data** [47] is provided by the BioThings API collection [48], which contains up-to-date human-curated annotations regarding indication and contraindication for drugs. We use drug-disease pairs with the relation "indication" as true positives while those with "contraindication" as true negatives.
- **SemMedDB Data** [49] is provided by the Semantic MEDLINE Database (SemMedDB), which leverages natural language processing (NLP) techniques to extract semantic triples with "treats" and "negatively treats" relations from PubMed abstracts. We use drug-disease pairs with the relation "treats" as true positives while those with "negatively treats" as true negatives.
- **NDF-RT Data** [50] is provided by National Drug File – Reference Terminology from Veterans Health Administration (VHA) which contains human-curated information on drug interaction, indications, and contraindications. We use drug-disease with therapeutics label "indications" as true positives while those with "contraindications" as true negatives.
- **RepoDB Data** [51] is a standard set of successful and failed drug-disease pairs in clinical trials collected by the Blavatnik Institute at Harvard Medical School. We use drug-disease with the status "approved" as true positives while those with "withdrawn" as true negatives.

Table 1. Pair count of true positive (indications) and true negative (contraindications or no effect) data from four data sources after data preprocessing.

Source	True Positive (Treats)	True Negative (Not Treat)
MyChem	3,663	26,795
SemMedDB	8,255	11
NDF-RT	3,421	5,119
RepoDB	2,127	738
Shared	3,971	526
**Total**	21,437	33,189

Note that 'shared' means those pairs are from two or more data sources.

We further filter drug-disease pairs from SemMedDB Data due to publication bias and possible NLP mistakes by using both the co-occurrence frequency and the PubMed-publication-based Normalized Google Distance (NGD) [52] defined below:

$$NGD(c1, c2) = \frac{max\{log\mathcal{N}(c1), log\mathcal{N}(c2)\} - log\mathcal{N}(c1, c2)}{logN - min\{log\mathcal{N}(c1), log\mathcal{N}(c2)\}} \quad (1)$$

where $c1$ and $c2$ are two biological concepts used in the customized BKG; $\mathcal{N}(c1)$ and $\mathcal{N}(c2)$ respectively represent the total number of unique PubMed IDs associated with $c1$ and $c2$; $\mathcal{N}(c1, c2)$ is the total number of unique PubMed IDs shared between $c1$ and $c2$; N is the total number of pairs of Medical Subject Heading (MeSH) terms annotations in PubMed database. Only the SemMedDB drug-disease pairs with at least 10 supporting publications and an NGD score of 0.6 or lower are left for the downstream model training.

These datasets are pooled together and then processed by 1). mapping the raw identifiers of drugs and diseases to the identifiers used in the customized BKG; 2) removing duplicate drug-disease pairs in both the true positive set and the true negative set. Table 1 shows the drug-disease pair count from each data source after data pre-processing.

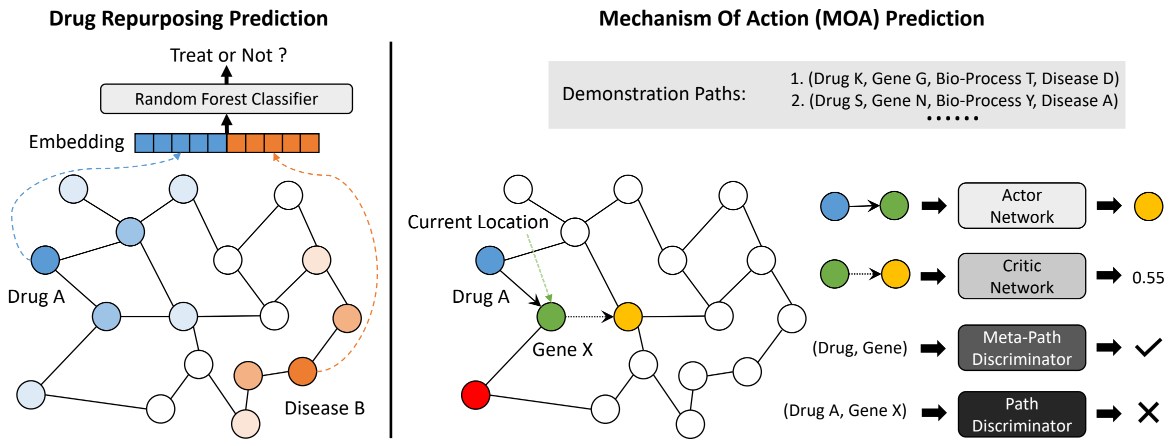

Figure 2. Illustration of entire KGML-xDTD model framework: drug repurposing prediction module (left) and mechanism of action (MOA) prediction module (right).

DrugMechDB

DrugMechDB[2] [53], to our best knowledge, is the first human-curated path-based database for explaining the mechanism of action (MOA) from a drug to a disease in an indication, with 3,593 MOA paths for 3,327 unique drug-disease pairs. These paths are extracted from free-text descriptions from DrugBank, Wikipedia, and other literature sources, and then have been curated by subject matter experts and also follow the schema of Biolink model. Hence, we can match them to nodes and edges used in the RTX-KG2 BKG via the *Node Synonymizer* function [23]. Since the length of predicted MOA paths generated by the KGML-xDTD framework is fixed to 3 in this study, we consider those 3-hop BKG-based paths of which all four nodes show up in the complete DrugMechDB MOA paths are the correct matched paths. Thus, we find 472 unique drug-disease pairs, of which each has at least one such correct matched path in all possible 3-hop paths between drug and disease in the customized BKG. We use these matched paths as true positive biologically meaningful paths for the evaluation of the model-predicted paths.

Model Framework

The model framework of KGML-xDTD consists of two modules: a drug repurposing prediction module that combines the advantages of GraphSAGE [40] and a Random Forest model, and an MOA prediction module that utilizes an adversarial actor-critic reinforcement learning (RL) model. We show the overview of the entire model framework in Figure 2. The implementation details of each module in KGML-xDTD framework are presented in Supplementary Section S2.

Notations

Let $\mathcal{G} = \{\mathcal{V}, \mathcal{E}\}$ be a directed biomedical knowledge graph, where each node $v \in \mathcal{V}$ represents a biological entity (e.g., a specific drug, disease, gene, or pathway, etc.) and each edge $e \in \mathcal{E}$ represents a biomedical relationship (e.g., *interacts-with, see more in Figure 1 b*). We use $\mathcal{V}^{\text{drug}}$ to represent all the drug nodes (the nodes with the categories of "Drug" and "Small Molecule" in the customized BKG) and $\mathcal{V}^{\text{disease}}$ to represent all the disease nodes (the nodes with the categories of "Disease", "PhenotypicFeature", "BehavioralFeature" and "DiseaseOrPhenotypicFeature" in the customized BKG). For each notation, we use bold formatting to represent its embedding (e.g., $\mathbf{v}$ represents the embedding of v).

2 https://sulab.github.io/DrugMechDB

Drug Repurposing Prediction

Drug repurposing aims to identify new indications of existing drugs/compounds. We solve it as a link prediction problem on the graph $\mathcal{G}$. Specifically, given any drug-disease pair (v_i, v_j) where $v_i \in \mathcal{V}^{\text{drug}}$ and $v_j \in \mathcal{V}^{\text{disease}}$, we predict the probability that drug i can be used to treat disease j. We first use GraphSAGE to calculate the embedding for each node. Ideally, the node embeddings should contain two kinds of information: node attributes and node neighborhoods.

To capture the neighborhood information, we optimize GraphSAGE to encourage neighbor nodes to have similar embeddings and non-neighbor nodes to have distinct embeddings. Specifically, we perform random walks for each node to collect its neighborhood information and train the model to maximize a node's similarity with its neighbor nodes. For a node u, the loss is calculated as:

$$L_{\mathcal{G}}(\mathbf{z}_u) = -\log(\sigma(\mathbf{z}_u^\top \mathbf{z}_v)) - k \cdot \mathbb{E}_{v_n \sim P_n(v)} \log(\sigma(\mathbf{z}_u^\top \mathbf{z}_{v_n})) \quad (2)$$

where $\mathbf{z}_u, \mathbf{z}_v$ are respectively the embeddings of nodes u, v, σ is the sigmoid function, v is a node that co-occurs with u in fixed-length random walks, P_n represents negative sampling distribution, and k indicates the number of negative samples (nodes not in u's fixed-length neighborhood).

To capture the node attributes information, we utilize the PubMedBERT model [54], a pre-trained language model designed for biomedical texts, to generate a node attribute embedding for each node based on the concatenation of the node's name and category. We further compress the embeddings to 100 dimensions with Principal Components Analysis (PCA) to reduce memory usage and use them as the initial node feature for GraphSAGE. In this way, the final GraphSAGE embedding of each node should contain the information regarding both graph topology and node attributes. We concatenate the GraphSAGE embeddings of drug-disease pairs and use them as input of a Random Forest model to classify each drug-disease pair into one of the "not treat", "treat", and "unknown" classes. We obtain "treat" and "not treat" drug-disease pairs from four data sources (described in Sec. "Data Sources for Model Training" on page 3). We generate "unknown" drug-disease pairs through negative sampling [55], that is, replacing the drug or disease identifier in each "treat" drug-disease pair with a random drug or disease identifier to generate a new pair that does not appear in both the "treat" and "not treat" classes. Specifically, for each unique "treat" drug-disease pair, we respectively replace its drug identifier with 30 other random drug identifiers as well as replace its disease identifier with 30 other random disease identifiers to make 60 new drug-disease pairs for the "unknown" class.

Mechanism of Action (MOA) Prediction

When potential indications of a given drug are identified by the drug repurposing prediction module, a natural yet essential question

is: can we biologically explain the predictions? We solve this by employing a reinforcement learning (RL) model to predict the BKG-based MOA paths, which are the paths on the knowledge graph from drug nodes to disease nodes. These BKG-based MOA paths can semantically describe an abstract biological process of how a drug treats a disease.

Demonstration paths. To encourage the RL agent to terminate the path searching at the expected diseases through a biologically reasonable path, we leverage so-called "demonstration paths", a set of biologically likely paths (e.g., drug1-gene1-protein3-disease1), that explains the underlying reasons for why a drug can treat a disease. We extract 396,705 demonstration paths from the customized BKG using the known drug-target interactions collected from two curated biomedical data sources: DrugBank (v5.1) and Molecular Data Provider (v1.2)[3], as well as the PubMed-publication-based Normalized Google Distance (NGD) (see Equation 1). We show more details regarding demonstration path extraction in Supplementary Section S3.

Adversarial Actor-critic Reinforcement Learning. We formulate the MOA prediction as a path-finding problem and adapt the Adversarial Actor-Critic Reinforcement Learning model [41] to solve it. Reinforcement learning is defined as a Markov Decision Process (MDP) which contains:

States: Each state s_t at time t is defined as $s_t = \left(v_{drug}, v_t, (v_{t-1}, e_t), \dots, (v_{t-K}, e_{t-(K-1)})\right)$ where $v_{drug} \in \mathcal{V}^{\text{drug}}$ is a given starting drug node; $v_t \in \mathcal{V}$ represents the node where the agent locates at time t; the tuple $\left(v_{t-K}, e_{t-(K-1)}\right)$ represents the previous Kth node and $(K-1)$th predicate. For the initial state s_0, the previous nodes and predicates are substituted by a special dummy node and predicate. We concatenate the embedding of all nodes and predicates of s_t to get the state embedding $\boldsymbol{s}_t$, where the node embeddings are node attribute embeddings generated with the PubMedBERT model (see Sec. "Drug Repurposing Prediction" on page 4) and the predicate embeddings employ one-hot vectors.

Actions: The action space A_t of each node v_t includes a self-loop action a_{self} and the actions to reach its outgoing neighbors in the graph $\mathcal{G}$. Due to memory limitation and extremely large outdegree of certain nodes in the knowledge graph, we prune the neighbor actions based on the PageRank scores if a node has more than 3,000 neighbors. Specifically, we let $A_t = (a_{self}, a_1, \dots, a_k, \dots, a_{n_{v_t}})$ where n_{v_t} is out-degree of node $v_t \in \mathcal{V}$. For each action $a_t = (e_t, v_{t+1}) \in A_t$ taken at time t, we concatenate its node and predicate embeddings to obtain action embedding $\boldsymbol{a}_t$. We learn two embedding matrices $E^{N_\text{n} \times \text{d}}$ and $E^{N_\text{p} \times \text{d}}$ respectively for nodes and predicates[4], where d represents the embedding dimension, N_n represents the number of nodes in graph, and N_p represents the number of predicate categories in graph.

Rewards: During the path searching process, the agent only receives a terminal reward $R_{e,T}$ from environment (that is, there is no intermediate reward from environment: $R_{e,t} = 0, \forall t < T$). Let v_T be the last node of the path, and $\mathcal{N}_{drug}$ be the known diseases that drug v_{drug} can treat. The terminal reward $R_{e,T}$ from environment is calculated with the drug repurposing model via:

$$R_{e,T} = \begin{cases} 1, & \text{if } v_T \in \mathcal{N}_{drug}. \\ p_{treat}, & \text{if } v_T \notin \mathcal{N}_{drug}; v_T \in \mathcal{V}^{\text{disease}} \text{ and } f(v_{drug}, v_T) \text{ is predicted as "treat"}. \\ 0, & \text{if } v_T \notin \mathcal{N}_{drug}; v_T \in \mathcal{V}^{\text{disease}} \text{ and } f(v_{drug}, v_T) \text{ is not predicted as "treat"}. \\ -1, & \text{if } v_T \notin \mathcal{V}^{\text{disease}}. \end{cases}$$

where p_{treat} is the "treat" class probability predicted by the drug repurposing model f.

The Adversarial Actor-critic RL model consists of four sub-networks that share the same model architecture MLP^i (note that i represents the id of each sub-network described later, such as a for *actor network*, c for *critic network*, etc.) but with different parameters:

$$\text{MLP}^i(X) = BA(BA(XW_1^i + b_1^i)W_2^i + b_2^i)W_3^i + b_3^i \tag{3}$$

where $\{W_1^i, W_2^i, W_3^i, b_1^i, b_2^i, b_3^i\}$ are the parameters and biases of linear transformations, BA represents a batch normalization layer followed by an ELU activation function.

Actor network: The actor network learns a path-finding policy π_θ (note that θ represents all parameters of actor network) to guide the agent to choose an action a_t from the action space A_t based on the current state s_t:

$$\pi_\theta(a_t|s_t, A_t) = \text{softmax}(\boldsymbol{A}_t \odot \text{MLP}^a(\boldsymbol{s}_t)) \tag{4}$$

where $\boldsymbol{A}_t$ is the embedding matrix of the action space A_t; $\odot$ represents the dot product. Here, $\pi_\theta(a_t|s_t, A_t)$ represents the probability of choosing action a_t at time t from the action space A_t given the state s_t.

Critic network: The critic network [56] estimates the expected reward $Q_\phi(s_t, a_t)$ (note that ϕ represents all parameters of the critic network) if the agent takes the action a_t at the state s_t by:

$$Q_\phi(s_t, a_t) = \text{MLP}^c(\boldsymbol{s}_t) \odot \boldsymbol{a}_t \tag{5}$$

Path discriminator network: Since the RL agent only receives a terminal reward $R_{e,T}$ from environment indicating whether it reaches an expected target, to encourage the agent to find biologically reasonable paths and provide intermediate rewards, we further guide it with demonstration paths. This network is essentially a binary classifier that distinguishes whether a path segment (s_t, a_t) is from demonstration paths or generated by the actor network. We treat all the known demonstration path segments (s_t^D, a_t^D) as positive samples and all actor-generated non-demonstration path segments (s_t^{ND}, a_t^{ND}) as negative samples. The path discriminator network $D_p(s, a) = \text{sigmoid}(\text{MLP}^p(\boldsymbol{s} \oplus \boldsymbol{a}))$, where $\boldsymbol{s}$ and $\boldsymbol{a}$ are respectively the embeddings of the state s and the action a; $\oplus$ represents the concatenation operator, is optimized with:

$$L_p = -\mathbb{E}_{(s,a)\sim P_D}[\log(D_p(s,a))] - \mathbb{E}_{(s,a)\sim P_A}[\log(1 - D_p(s,a))] \tag{6}$$

where P_D and P_A respectively represent the demonstration path segment distribution and the actor-generated non-demonstration path segment distribution. Based on the probability $D_p(s_t, a_t)$, the path-discriminator-based intermediate reward $R_{p,t}$ is calculated as:

$$R_{p,t} = \log(D_p(s_t, a_t)) - \log(1 - D_p(s_t, a_t)). \tag{7}$$

Meta-Path discriminator network: Similar to the path discriminator, this network aims to judge whether the meta-path of the actor-generated paths is similar to that of demonstration paths. The meta-path is the path of node categories (e.g., ["Drug"→"Gene"→"BiologicalProcess"→"Disease"]). Similarly, the meta-path discriminator $D_m(M) = \text{sigmoid}(\text{MLP}^m(\boldsymbol{M}))$, where $\boldsymbol{M}$ is the embedding of the meta-path M defined as the concatenation of learned category embeddings of all nodes that appear in the path, is also a binary classifier where the meta-paths of demonstration paths are treated as positive samples while others are negative samples. We optimize it with the following loss:

$$L_m = -\mathbb{E}_{M\sim P_D^M}[\log(D_m(M))] - \mathbb{E}_{M\sim P_A^M}[\log(1 - D_m(M))] \tag{8}$$

where P_D^M and P_A^M respectively represent the demonstration meta-path distribution and the actor-generated non-demonstration meta-path distribution. The intermediate reward $R_{m,t}$ generated by the meta-path discriminator is calculated by:

$$R_{m,t} = \log(D_m(M)) - \log(1 - D_m(M)). \tag{9}$$

The integrated intermediate reward R_t at time t is then calculated as:

$$R_t = \alpha_p R_{p,t} + \alpha_m R_{m,t} + (1 - \alpha_p - \alpha_m)\gamma^{T-t} R_{e,T} \tag{10}$$

3 https://github.com/NCATSTranslator/Translator-All/wiki/Molecular-Data-Provider
4 Each sub-network uses separate embedding matrices.

where $\alpha_p \in [0,1]$ and $\alpha_m \in [0, 1-\alpha_p]$ are hyperparameters, γ is the decay coefficient, and $R_{e,T}$ is defined in the "Rewards" section above.

To optimize the critic network, we minimize the Temporal Difference (TD) error [57] with loss:

$$L_c = \mathrm{TD}^2 = [(R_t + Q_\phi(s_{t+1}, a_{t+1})) - Q_\phi(s_t, a_t)]^2. \quad (11)$$

Since the goal of the actor network is to achieve the largest expected reward by learning an optimal actor policy, we optimize the actor network by maximizing $J(\theta) = \mathbb{E}_{a\sim\pi_\theta}[Q_\phi(s_t, a)]$. We use the REINFORCE algorithm [58] to optimize the parameters. To encourage more diverse exploration in finding paths, we use the entropy of π_θ as a regularization term and optimize the actor network with the following stochastic gradient of the loss function L_a:

$$\nabla_\theta L_a = -\nabla_\theta J(\theta) = -\mathbb{E}_{\pi_\theta}[\nabla_\theta \mathrm{TD} \log \pi_\theta(a_t|s_t)] - \alpha\nabla_\theta \mathrm{entropy}(\pi_\theta) \quad (12)$$

where π_θ is the action probability distribution based on the actor policy, and α is the entropy weight.

We follow Zhao et al. [41] to train the Adversarial Actor-critic RL model in a multi-stage way. First, we initialized the actor network using the behavior cloning method [59] in which the training set of demonstration paths is used to guide the sampling of the agent with Mean Square Error (MSE) loss. Then, in the first z epochs, we freeze the parameters of the actor network and the critic network and respectively train the path discriminator network and meta-path discriminator network by minimizing L_p and L_m. After z epochs, we unfreeze the actor network and the critic network and optimize them together by minimizing a joint loss $L_{joint} = L_a + L_c$.

Results

Evaluation Settings

Data Split

The post-processed drug-disease pairs (described in Sec. "Data Sources for Model Training" on page 3) are split into training, validation, and test sets where the drug-disease pairs of each unique drug are randomly split according to a ratio of 8/1/1. For example, let's say drugA has 10 known diseases that it treats (e.g., drugA-disease1, ..., drugA-disease10), 8 pairs are randomly split into the training set, 1 pair is to the validation set, 1 pair to the test set. With this data split method, the model can be exposed to every drug in the training set, which complies with our goal of predicting new indications of known drugs and their potential mechanisms of action (MOAs) based on the MOA of known target diseases.

Evaluation Metrics

The proposed framework `KGML-xDTD` is evaluated on two types of tasks: predicting drug-disease "treat" probability (i.e., drug repurposing prediction) as well as identifying biologically reasonable BKG-based MOA paths from all candidates (i.e., MOA prediction). These two tasks are evaluated based on classification accuracy-based metrics (e.g., accuracy, macro f1 score) and ranking-based metrics (e.g., mean percentile rank, mean reciprocal rank, and proportion of ranks smaller than K) defined as follows:

Accuracy (ACC) is the fraction of the model classification is correct, computed as:

$$\mathrm{ACC} = \frac{\text{Number of correct classifications}}{\text{Total number of drug-disease pair classifications}} \quad (13)$$

Macro F1 score (Macro-F1) is the unweighted mean of all the per-class F1 scores:

$$F1^c = 2 * \frac{precision^c \times recall^c}{precision^c + recall^c} \qquad \text{Macro-F1} = \frac{1}{|C|}\sum_{c\in C} F1^c \quad (14)$$

where C presents classification classes (e.g.,"treat," "not treat," and "unknown").

Mean Percentile Rank (MPR) is the average percentile rank of the 3-hop correct DrugMeshDB-based matched path (described in Sec. "DrugMechDB" on page 4) of true positive drug-disease pairs:

$$\mathrm{MPR} = \frac{1}{|PR|}\sum_{pr\in PR} pr \quad (15)$$

where PR is a list of percentile ranks of correct matched paths of true positive drug-disease pairs ("treat" category).

Mean Reciprocal Rank (MRR) is the average inverse rank of true positive drug-disease pairs ("treat" category) or their 3-hop correct matched paths:

$$\mathrm{MRR} = \frac{1}{|R|}\sum_{r\in R}\frac{1}{r} \quad (16)$$

where R is a list of ranks of true positive drug-disease pairs or their correct matched paths.

Hit@K is the proportion of ranks smaller thank K for true positive drug-disease pairs ("treat" category) or their 3-hop correct matched paths:

$$\mathrm{Hit@K} = \frac{1}{|R|}\sum_{r\in R}|r \le k| \quad (17)$$

where R is a list of ranks of true positive drug-disease pairs or their correct matched paths.

Drug Repurposing Prediction Evaluation Method

We utilize the metrics *ACC* and *Macro-F1* to measure the accuracy of drug repurposing prediction of our `KGML-xDTD` framework while using ranking-based metrics *MRR* and *Hit@K* to show its capability in reducing false positive (i.e., the false drug-disease pairs ranking higher among possible drug-disease candidates). We use the following three methods to generate non-true-positive drug-disease candidates for each true positive drug-disease pair for the *MRR* and *Hit@K* calculation:

- **Drug-rank-based replacement**: For each true positive drug-disease pair, the drug-rank-based replacement pairs are generated by replacing the drug entity with each of all 274,676 other drugs in the customized BKG while excluding all known true positive drug-disease pairs.
- **Disease-rank-based replacement**: For each true positive drug-disease pair, the disease-rank-based replacement pairs are generated by replacing the disease entity with each of all 124,638 other diseases in the BKG while excluding all known true positive drug-disease pairs.
- **Combined Replacement**: For each true positive drug-disease pair, the combined replacement pairs are the combination of all replacement pairs of the above two methods. All known true positive drug-disease pairs are excluded from these replacement pairs.

Due to the massive size of possible drug-disease candidates, some baseline models (e.g., GAT and GraphSAGE+SVM) are not applicable in this setting within a reasonable time (e.g., a week). Thus, we also use a small subset of drug-disease replacement (we use 1000 random drug-disease pairs from the combined replacement set above: 500 with drug id replacement and 500 with disease id replacement) to calculate *MRR* and *Hit@K* in order to compare `KGML-xDTD` with all baselines. In addition, since the drug repurposing prediction module of `KGML-xDTD` framework does 3-class classification while other baselines do 2-class classification, for a fair comparison, we re-calculate *ACC* and *Macro-F1* for `KGML-xDTD` by excluding the "unknown" class.

MOA Prediction Evaluation Method

For the evaluation of MOA prediction, we use the DrugMechDB [42] to obtain the expert-verified MOA paths as ground-truth data and match each biological concept in these verified MOA paths to the biological entities used in the customized BKG, and then generate

Table 2. Comparing evaluation metrics between KGML-xDTD and different baseline models based on test dataset. The top panel shows the performance of state-of-the-art (SOTA) baseline models; the middle panel shows the performance of variants of KGML-xDTD model framework; the bottom panel shows the performance of KGML-xDTD model framework.

Model	Accuracy	Macro F1 score	MRR	Hit@1	Hit@3	Hit@5
TransE	0.707	0.708	0.280	0.117	0.301	0.449
TransR	0.857	0.854	0.301	0.123	0.343	0.518
RotatE	0.704	0.704	0.252	0.075	0.283	0.4484
DistMult	0.555	0.495	0.173	0.041	0.142	0.259
ComplEx	0.624	0.460	0.131	0.020	0.105	0.193
ANALOGY	0.594	0.465	0.180	0.045	0.145	0.272
SimplE	0.599	0.472	0.163	0.038	0.137	0.241
GAT	**0.936**	**0.934**	0.003	0.001	0.001	0.001
GraphSAGE-link	0.919	0.915	0.002	0	0	0
GraphSAGE+logistic	0.791	0.784	0.002	0	0	0
GraphSAGE+SVM	0.807	0.793	0.002	0	0	0
KGML-xDTD w/o NAEs	0.909 (0.898*)	0.891 (0.892*)	0.150	0.029	0.138	0.243
2-class KGML-xDTD	0.929	0.925	0.271	0.177	0.310	0.381
KGML-xDTD (ours)	0.935 (0.930*)	0.923 (0.926*)	**0.356**	**0.206**	**0.407**	**0.522**

1. The values with * inside the parenthesis are the adjusted results by excluding the "unknown" category for a fair comparison.
2. The ranking metrics (e.g., "MRR" and "Hit@K") are calculated with the random drug-disease replacement method (i.e., we use 1,000 random drug-disease pairs to calculate the ranks of true positive drug-disease pairs in test dataset).
3. "NAEs" represents node attribute embeddings.

the correct matched paths (described in Sec. "DrugMechDB" on page 4). we first calculate the path scores for all 3-hop KG paths between drug and disease with the path-finding policy learned from Adversarial Actor-critic Reinforcement Learning (RL) model using equation:

$$\text{path score} = \sum_{i=1}^{k} \delta^{i-1} \times \log(P_i \times N_i) \tag{18}$$

where k is the number of hops in this path; δ is a decay coefficient (we set it to 0.9 in this study); P_i represents the probability of choosing action a_i in the ith hop following this path based on the trained RL model; N_i is the number of possible actions in the ith hop.

With these path scores, we obtain the ranks of the matched BKG-based MOA paths and calculate the ranking-based metrics *MPR*, *MRR*, and *Hit@K*. For those drug-disease pairs with multiple BKG-based MOA paths, we use the highest ranks of their paths. We compare KGML-xDTD with the baseline models based on these metrics to show the capability of MOA prediction module of KGML-xDTD in identifying biologically reasonable BKG-based MOA paths from a massive and complex BKG with comparably low false positive. In addition, we further perform two case studies to evaluate the effectiveness of KGML-xDTD in identifying the biologically reasonable BKG-based paths.

Drug Repurposing Prediction Evaluation

For drug repurposing prediction evaluation, we compare the KGML-xDTD model framework against several state-of-the-art (SOTA) KG-based models and variants of KGML-xDTD for drug repurposing prediction based on the method described in Sec. "Drug Repurposing Prediction Evaluation Method" on page 6.

We use eight different SOTA KG-based models as baseline models that are commonly used for BKG-based drug repurposing [19, 60]. TransE [25], TransR [61], RotatE [26] are the translation-distance-based models that regard a relation (e.g., "treats") as a "translation"/"rotation" (e.g., a kind of spatial transformation) from a head entity (e.g., a drug node) to a tail entity (e.g., a disease node). DistMult [27] is a bilinear model that measures the latent semantic similarity of a knowledge-graph triple (head entity, relation/predicate, tail entity) with a trilinear dot product. ComplEx [28] and ANALOGY [62] are the extensions of DistMult that consider more complex relations (e.g., asymmetric relations). SimpLE [63] is a tensor-factorization-based model to learn the semantic relation of a knowledge-graph triple. GAT [64] is a popular graph neural model that leverages the important graph topology structure based on self-attention mechanism for graph-associated tasks (e.g., link prediction). Implementation details of these baselines are presented in Supplementary Section S4.

Besides these SOTA baseline models, we also compare the drug repurposing prediction module in KGML-xDTD with its several variants to show the effectiveness of model components. For example, to show efficacy of the combination of GraphSage and Random Forest (RF), we use a pure GraphSAGE for link prediction (GraphSAGE-link), the combination of GraphSage and logistic model (GraphSAGE-logistic), and the combination of GraphSage and Support Vector Machine (SVM) model (GraphSAGE-SVM). To demonstrate the effectiveness of node attribute embeddings (described in Sec. "Drug Repurposing Prediction" on page 4) in improving repurposing prediction, we conduct an ablation experiment that replaces node attribute embeddings (NAEs) with random embeddings (initialized with the Xavier method [65]) as GraphSage initialized embeddings (KGML-xDTD w/o NAE); to support rationality of setting "unknown" class through negative sampling (described in Sec. "Drug Repurposing Prediction" on page 4), we modify the drug repurposing prediction module for 2-class classification[5] (2-class KGML-xDTD) as a baseline comparison model.

Table 2 shows the performance of KGML-xDTD model framework and other baseline models in the task of drug repurposing prediction based on the metrics described in Sec. "Evaluation Metrics" on page 6 and test dataset along with 1,000 random drug-disease replacement pairs. As shown in the table, on the one hand, the KGML-xDTD outperforms most of the baseline models and achieves comparable performance as GAT in classification-based metrics (e.g., accuracy, macro f1 score), indicating its effectiveness in classifying known "treat" and "not treat" drug-disease pairs with both attribute and neighborhood information on the knowledge graph. On the other hand, KGML-xDTD's exceptional performance in ranking-based metrics shows its superiority over baselines in identifying new indications of existing drugs out of a large number of possible drug-disease pairs with relatively low false positives (Figure 3 further supports this conclusion with three different "complete" re-

5 only considers true positive and true negative.

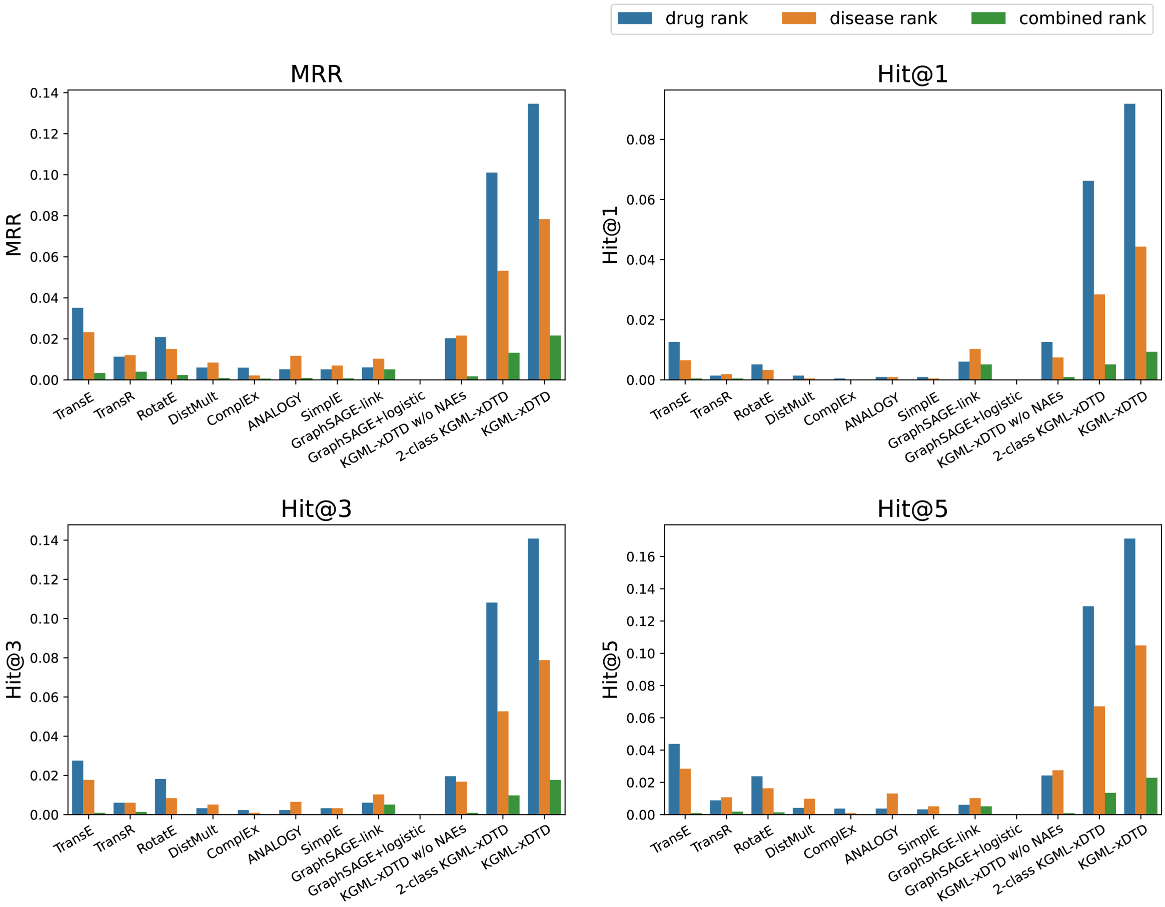

Figure 3. Comparing MRR and Hit@K metrics between KGML-xDTD and other baseline models based on test dataset. The legend "drug rank", "disease rank" and "combined rank" respectively correspond to the methods of "Drug-rank-based replacement", "Disease-rank-based replacement", and "Combined replacement" described in Sec. "Drug repurposing prediction evaluation Method" on page 6.

Table 3. Comparing evaluation results on MOA prediction between KGML-xDTD and different baseline models (e.g., MultiHop model and KGML-xDTD without demonstration paths (KGML-xDTD w/o DP)) based on test dataset.

Model	MPR	MRR	Hit@1	Hit@10	Hit@50	Hit@100	Hit@500
MultiHop	61.400%	0.027	0.017	0.042	0.067	0.118	0.345
KGML-xDTD w/o DP	72.965%	0.015	0.008	0.017	0.067	0.160	0.403
KGML-xDTD (ours)	**94.696%**	**0.109**	**0.059**	**0.193**	**0.496**	**0.613**	**0.849**

placement methods), which is of great importance for guiding clinical research. Besides, by comparing 2-class KGML-xDTD with the vanilla GraphSAGE model (e.g., GraphSAGE-link), we demonstrate the effectiveness of the Random Forest model over a neural network classifier in this task. The comparison between KGML-xDTD w/o NAE and KGML-xDTD shows that the KGML-xDTD benefits from the use of node attribute embeddings for drug repurposing prediction while the comparison with 2-class KGML-xDTD indicates the effectiveness of using negative sampling to generate "unknown" drug-disease pairs for model training. With the "unknown" drug-disease pairs, the KGML-xDTD model achieves significant improvement in ranking-based metrics, which is essential when applying to real-world drug repurposing because it can reduce the false positives.

MOA prediction evaluation

For MOA prediction, we evaluate how well the KGML-xDTD can identify the DrugMechDB-matched BKG-based MOA paths (described in Sec. "MOA Prediction Evaluation Method" on page 6) from a large number of possible paths in the customized BKG by utilizing ranking-based metrics (e.g., MPR, MRR, and Hit@K) and two specific case studies.

There are few machine learning models designed for the task of identifying biologically meaningful paths from biomedical knowledge graphs for explaining drug repurposing. To our best knowledge, although three models (e.g., UKGE [30], GrEDeL [32], Polo [38], all mentioned in Sec. "Introduction" on page 1) were proposed and can be used for this goal, they all have certain constraints and cannot be used as baseline models for comparison. The UKGE model cannot be applied to BKGs without weighted edge information (e.g., frequency of relation appeared in literature). The authors of the GrEDeL model don't provide the code to implement this model. The Polo model cannot be trained within a reasonable time (e.g., within two weeks) on a massive and complex BKG (e.g., RTX-KG2) due to its dependence on a computationally inefficient method "DWPC" [15]. Therefore, we choose the MultiHop reinforcement learning model [66] as a baseline model since it uses the LSTM model framework as the GrEDel model and allows using a self-defined reward shaping strategy in its reward function as what we do in the KGML-xDTD model (i.e., we can use the same reward strategy described in Sec. "Adversarial Actor-critic Reinforcement Learning" on page 5). Furthermore, we also compare with an ablated version of KGML-xDTD (i.e., KGML-xDTD w/o DP which does not take advantage of the demonstration paths by setting α_p and α_m in Function 9 as 0) as another baseline model to show the importance of demonstration paths.

We show the comparison results between the KGML-xDTD model framework and different baseline models for MOA prediction evaluation in Table 3. Although all the models receive the same terminal rewards from the environment, that is given by the drug repurposing prediction module of KGML-xDTD, the MOA prediction module of KGML-xDTD achieves significantly better performance in identifying DrugMechDB-matched BKG-based MOA paths than the other two baselines across all ranking-based metrics. Comparison between the KGML-xDTD with and without demonstration paths (i.e., KGML-xDTD w/o DP) further illustrates the great effectiveness of using demonstration paths to guide the path-finding process. Due to the massive searching space and sparse rewards, the RL agent often fails to find reasonable paths out of many possible choices, while our model KGML-xDTD, with the intermediate guidance provided by the demonstration path, is able to identify the biologically reasonable choices with a much higher probability. Moreover, regardless of the KGML-xDTD with and without demonstration paths, we can see that they both are superior to the MultiHop, which illustrates that the actor-critic model structure might be more effective than LSTM for this task.

To further evaluate the performance of KGML-xDTD model framework in identifying biologically relevant MOA paths for drug repurposing, we present two different case studies to explore the potential repurposed drugs and their potential mechanism for two rare genetic diseases: Hemophilia B and Huntington's disease.

Table 4. Top 10 predicted drugs/treatments for hemophilia B (note that the drugs highlighted in red color are used in the training set).

Drug/Treatment	Prob.	Publications
Eptacog Alfa (rFVIIa)	0.833	[67, 68]
Nonacog Alfa (rFIX)	0.803	[69]
Viral Vector	0.780	[70]
Factor VIIa	0.748	[71, 67]
Recombinant FVIIa (rFVIIa)	0.724	[71, 67]
Thrombin	0.709	[72]
Factor IX	0.708	[73]
Epicriptine	0.702	
Hyperbaric Oxygen	0.660	
Triamcinolone	0.649	

Case 1: Hemophilia B

Hemophilia B, also known as factor IX deficiency or Christmas disease, is a rare genetic disorder that results in prolonged bleeding in patients. It is caused by mutations in the factor IX (F9) gene, which is located on the X chromosome. Table 4 displays the top 10 drugs/treatments predicted by the KGML-xDTD model framework, including both those that are used in the training set (highlighted in red) and those that are not. Besides those known drugs/treatments used in the training set, the majority of the remaining seven drugs/treatments on the list are supported by published research and have the potential to treat hemophilia B. For example, the activated human-derived coagulation factor VII (i.e., Factor VIIa) or the recombinant activated factor VII (i.e., rFVIIa) is one of the proteins that can cause blood clots as an important part of the blood coagulation regulatory network (as shown in the Figure 4). This protein is used as an effective inhibitor in the treatment of patients with hemophilia B [71, 67]. Thrombin is a key enzyme in the maintenance of normal hemostatic function. It has been reported that using thrombin as a therapeutic strategy can help prevent bleeding in patients with hemophilia [72]. The use of recombinant factor IX therapy is a recommended treatment option for individuals with hemophilia B [73]. Some examples of recombinant factor IX products include BeneFIX, Rixubis, Ixinity, Alprolix Idelvion, and Rebinyn.

To further assess the biological explanations of the predicted 3-hop BKG-based MOA paths for the treatment of hemophilia B, we have used the curated DrugMechDB-based MOA paths, which are not used in the model training process. DrugMechDB contains relevant MOA paths of hemophilia B treatment only for Eptacog

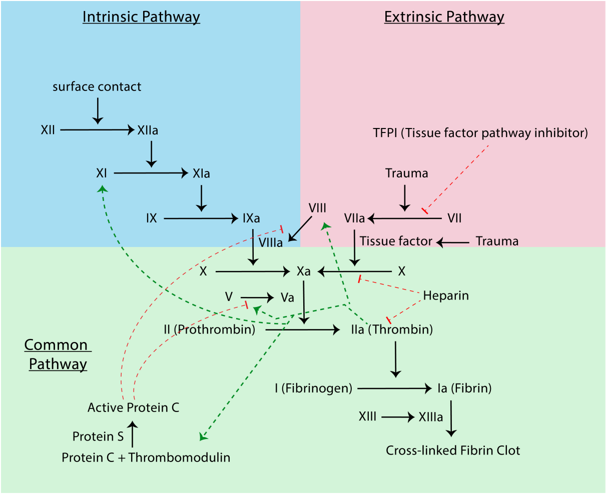

Figure 4. Blood Coagulation Regulatory Network with arrows for molecular reactions (black), positive feedback (green), and negative feedback (red).

Alfa and Nonacog Alfa. We show the comparisons between the subgraphs with the top 10 predicted 3-hop BKG-based paths and the curated DugMechDB-based MOA paths for Eptacog Alfa and Nonacog Alfa in Figure 5. The corresponding biological entities between the predicted paths and the curated DrugMechDB-based paths are highlighted in red color. Although the predicted paths can't exactly match the DrugMechDB-based MOA paths due to the limited path length and some missing semantic relationships in the customized biomedical knowledge graph, key biological entities (such as Coagulation Factor VII, Coagulation Factor X, and Coagulation Factor IX) that are important for the treatment of hemophilia B are present in the subgraphs of the top 10 predicted paths. As shown in Figure 4, the treatment of hemophilia B involves a complex molecular network of blood coagulation, and many of the coagulation factors (such as factor VII, factor VIIa, factor III, factor II, factor VIII, factor IX, and factor X) present in the subgraphs of the top 10 predicted paths are also part of this molecular network. In Supplementary Section S5, we also use Factor VIIa to assess the biological explanations of the predicted 3-hop BKG-based MOA paths for the predicted drugs/treatments that are not in the training set. The subgraph results show similar molecular details as those in Figure 4 for treating hemophilia B. Therefore, the predicted paths by `KGML-xDTD` model framework can help identify key molecules in the real drug action regulatory network, thereby aiding in explaining drug repurposing to some extent.

Case 2: Huntington's disease

Huntington's disease (HD) is a rare neurogenetic disorder that typically occurs in midlife with symptoms of depression, uncontrolled movements, and cognitive decline. While there is currently no drug/treatment that can alter the course of HD, some drugs/treatments can be useful for the treatment of its symptoms in abnormal movements (e.g., chorea) and psychiatric phenotypes. We show ten drugs/treatments with the highest predicted probability by the `KGML-xDTD` model framework after manual processing in Table 5. This processing involves excluding the chemotherapeutic drugs from the predicted drug candidate list due to their potential risk of cytotoxicity to normal cells (which could lead to false positives for drug repurposing of non-cancer diseases [74, 75]), and only presenting the top 5 results in the training set, and top 5 from the test or validation set. From this table, it can be observed that many of the top-ranked predicted drugs have been supported by publications as potential treatments for the symptoms of HD. Since there is currently no effective treatment for HD, DrugMechDB does not have a corresponding MOA path for comparison. To analyze the predicted paths by the `KGML-xDTD` model framework for the predicted non-chemotherapeutic drugs/treatments that are not included in the training set (shown in black in Table 5), we present their top 10 predicted paths (integrated into different subgraphs) in Figure 6. From these predicted paths, we can see that most of them are biologically relevant. For example, the subfigure (a) of Figure 6 shows that Risperidone is predicted to be useful for the treatment of HD by decreasing the activity of the genes associated with the 5-Hydroxytryptamine receptor (e.g., HTR1A, HTR2A, HTR2C, HTR7) and dopamine receptor (e.g., DRD1, DRD2, DRD3) which have been proven to be involved in the pathogenesis of depressive disorders [76, 77]. The presence of depressive symptoms is a significant characteristic of HD [78]. Entinostat is predicted to have the potential to alleviate the symptoms of HD by inhibiting the functions of histone deacetylase genes such as HDAC1, HDAC2, HDAC6 (see subfigure (b) of Figure 6), and one of the predicted 3-hop MOA paths ("Entinostat" → "decreases activity of" → "HDAC1 gene" → "interacts with" → "Histone H4" → "gene associated with condition" → "Huntington's disease") is supported by the previous research [79, 80]. Primaquine is predicted to act on the NQO2 gene and the IKBKG gene to potentially play a therapeutic role in neurodegenerative disease, reported in [81, 82]. According to the predicted MOA paths, Isradipine may have a potential therapeutic effect for HD by mainly regulating the genes of the Calcium Voltage-Gated Channel, including CACNA1S, CACNA1D, CACNA1C, CACNB2, CACNA2D2. These genes may be associated with the symptoms of HD, such as chorea, depression, and dementia [83]. Lastly, Amifampridine is predicted to regulate the genes of the Potassium Voltage-Gated Channel which are potentially associated with HD [84]. All these examples indicate that the predicted BKG-based MOA paths can explain the mechanism of repurposed drugs to some extent.

Table 5. Top 5 predicted drugs/treatments used in the training set (highlighted in red color) and the top 5 non-chemotherapeutic predicted drugs/treatments that are not in the training set for Huntington's Disease.

Drug/Treatment	Prob.	Publications
Pimozide	0.939	[85, 86]
Therapeutic Agent	0.939	
Olanzapine	0.938	[87, 88]
Riluzole	0.935	[89]
Antipsychotic Agent	0.932	[90]
Risperidone	0.893	[78, 91]
Entinostat	0.888	[79]
Primaquine	0.887	
Isradipine	0.884	[92]
Amifampridine	0.882	

Discussion

In this work, we propose `KGML-xDTD`, a two-module, knowledge graph-based machine learning framework that not only predicts the treatment probabilities between drugs/compounds and diseases but also provides biological explanations for these predictions through the predicted paths in a massive biomedical knowledge graph with comprehensive biomedical data sources as potential mechanisms of action. This framework can assist medical researchers in quickly identifying the potential drug/compound-disease pairs that might have a treatment relationship, which can accelerate the process of drug discovery for emerging diseases. Additionally, by leveraging the KG-based paths predicted by the framework, medical professionals (e.g., doctors and licensed medical practitioners) can straightforwardly assess the accuracy of the predictions via the predicted MOAs, which can help to reduce false positives that may be produced by the "black-box" operation of traditional machine learning models.

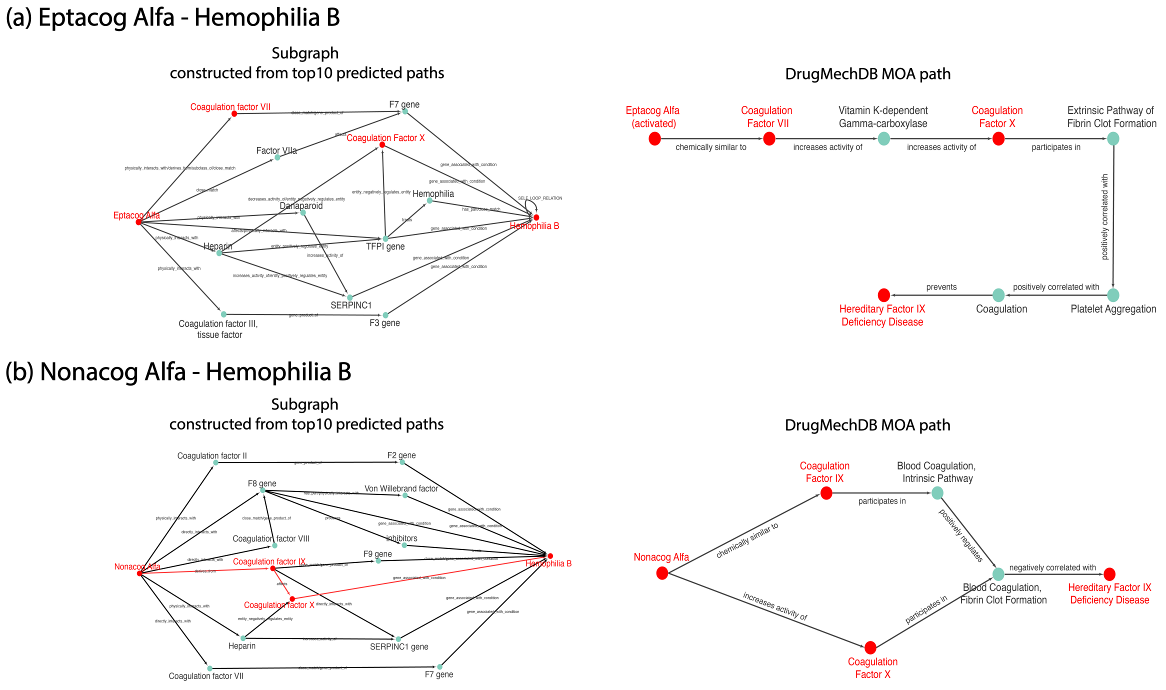

Figure 5. Comparison between the top 10 predicted 3-hop paths (integrated into a subgraph for better visualization) and the curated DugMechDB-based MOA paths for Eptacog Alfa (a) and Nonacog Alfa (b). On the left is a network representation of the top 10 `KGML-xDTD` predicted paths. On the right are network representations of the human-curated DrugMechDB mechanism of action paths. In both, the corresponding biological entities/vertices between the top 10 predicted 3-hop paths and MOA paths are highlighted in red color. The red edges indicate a top-10-`KGML-xDTD` predicted path where all entities show up in the DugMechDB-based MOA paths.

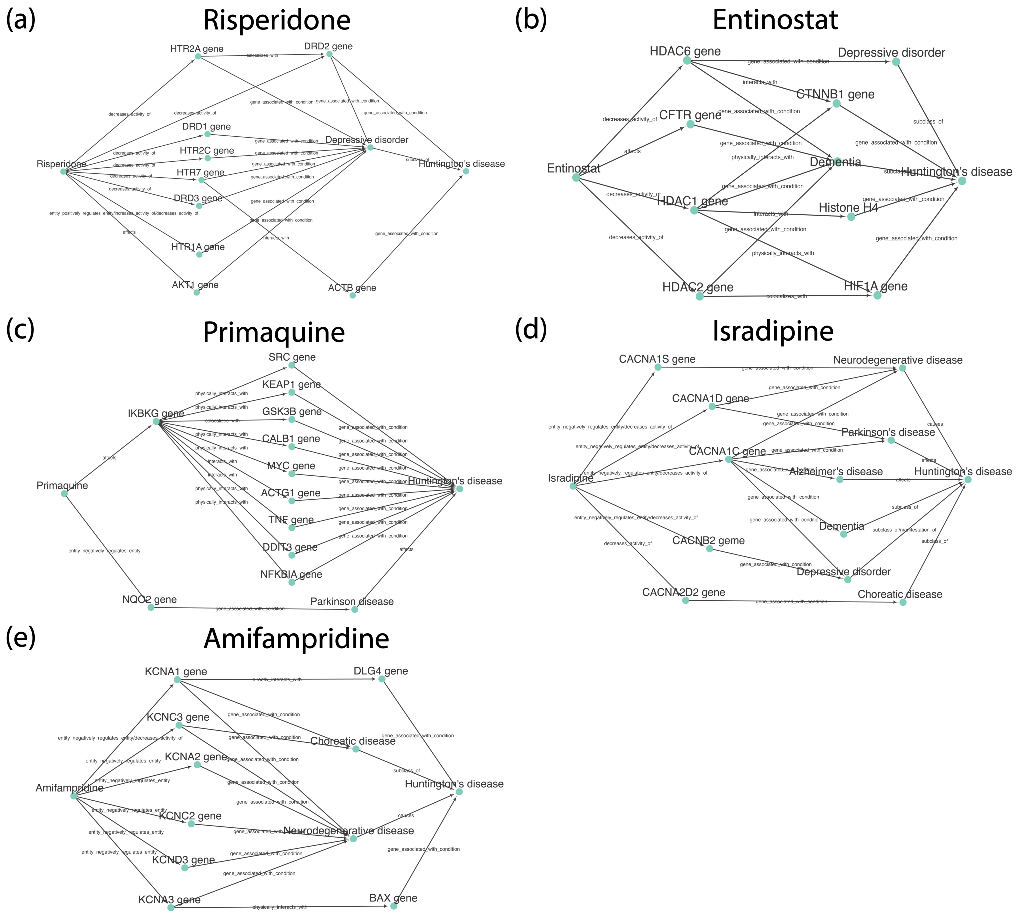

Figure 6. Top 10 predicted 3-hop BKG-based MOA paths (integrated into subgraphs) for top 5 non-chemotherapeutic predicted drugs/treatments that are not included in the training set for Huntington's disease.

Although previous research [15, 19, 24] has applied a variety of models to the task of drug repurposing using biomedical knowledge graphs (BKGs), these approaches are implemented in the small-scale BKGs and many do not scale to larger graphs. As biotechnology advances and the volume of data in biomedical databases increases, BKGs are becoming larger and more complex. In our comparison with state-of-the-art KG-based models for drug repurposing, we find that the `KGML-xDTD` model had higher accuracy with lower false positives when applied to a massive and complex biomedical knowledge graph *RTX-KG2c*. By evaluating the predicted paths with DrugMechDB and two case studies, we show that the model can capture some key biological entities involved in real drug action regulatory networks.

It is widely acknowledged that drug repurposing is one of the most challenging problems in biomedicine, and current AI techniques are still in the early stages of addressing it. Many other AI models, such as those based on chemical structure, drug-target interactions, and drug perturbations of gene expression, are developed for solving this goal. They may offer more accurate predictions but also have limitations in terms of cost and the availability of samples for specific diseases. Biomedical knowledge graph (BKG)-based machine learning models, such as the `KGML-xDTD` model, offer a cost- and time-efficient alternative due to the large volume of biomedical knowledge stored in public databases and publications. The `KGML-xDTD` model framework is not intended to replace or beat these models, but rather provides a complementary approach that leverages emerging knowledge graphs for drug repurposing.

Future work to further enhance the `KGML-xDTD` model framework might include extending the predicted paths for more specific explanations, and considering the negative drug-disease pairs so that the model can explain why certain drugs are harmful to diseases.

Availability of source code and requirements

- **Project name**: KGML-xDTD
- **Project home page**: https://github.com/chunyuma/KGML-xDTD
- **Operating system(s)**: Linux (Ubuntu)
- **Resource usage in training step**: A Linux (Ubuntu) system with at least 8 CPU cores, 800GB of VRAM, and a 48GB GPU card (48GB Quadro RTX 8000 GPU card used in our training).
- **Resource usage in inference step**: A Linux (Ubuntu) system with at least 8 CPU cores and 50GB of VRAM. The GPU card is not necessary but if used, the GPU card needs at least 24GB VRAM (48GB Quadro RTX 8000 GPU card used in our inference).
- **Time Requirement**: Based on our hardware performance and parameter settings (please see the scripts on Github), the training step takes approximately two weeks while the inference step takes approximately 25.42 seconds for one drug-disease pair with 3,320 potential paths. These time estimates may vary depending on the hardware performance, parameter settings, and the number of potential paths of given drug-disease pair.
- **Programming language**: Shell Script (Bash) with Python 3.8.12
- **Other requirements**: Python 3.8.12 with GPU/CPU support (GraphSAGE training needs Python 2.7), neo4j-community 3.5.26, miniconda 4.8.2 (please see more requirements in the yaml files under "envs" folder on Github repository)
- **Licenses**: MIT license, DrugBank academic license, Apache 2.0 license, UMLS Metathesaurus license, CC-BY 4.0 license

Availability of supporting data and materials

The data sets supporting the results of this article are publicly available in the Zenodo repository, DOI:10.5281/zenodo.7582233.

Additional Files

Supplementary Section S1. Biomedical Knowledge Graph RTX-KG2c Pre-processing.
Supplementary Section S2. Implementation Details of `KGML-xDTD` Model Framework.
Supplementary Section S3. Implementation Details of Demonstration Path Extraction.
Supplementary Section S4. Implementation Details of Baseline Models.
Supplementary Section S5. A subgraph with the top 10 predicted 3-hop MOA paths for Factor VIIa – Hemophilia B pair.

Declarations

List of abbreviations

ACC: Accuracy;
ADAC: ADversarial Actor-Critic;
BKG: Biomedical Knowledge Graph;
DWPC: Degree-Weighted Path Count;
EHR: Electronic Health Record;
GRL: Graph Reinforcement Learning;
KG: Knowledge Graph;
LSTM: Long Short-Term Memory;
Macro-F1: Macro F1 score;
MDP: Markov Decision Process;
MeSH: Medical Subject Heading;
MOA: Mechanism of Action; RL: Reinforcement Learning;
MPR: Mean Percentile Rank;
MSE: Mean Square Error;
NAE: Node Attribute Embedding;
NGD: Normalized Google Distance;
NLP: Natural Language Processing;
PCA: Principal Components Analysis;
RF: Random Forest;
RNN: Recurrent Neural Network;
RTX-KG2:the Reasoning Tool X Knowledge Graph 2;
RTX-KG2c:the canonicalized version of the Reasoning Tool X Knowledge Graph 2;
SemMedDB: Semantic MEDLINE Database;
SVM: Support Vector Machine;
VHA: Veterans Health Administration;

Ethical Approval (optional)

Not applicable

Consent for publication

Not applicable

Competing Interests

The authors declare no competing interests.

Funding

Research reported in this publication was supported by the National Center for Advancing Translational Sciences (NCATS) of the National Institutes of Health (NIH) under award numbers: OT2-TR003428-01S3, OT2-TR003428-01S2, OT2-TR003428-01; National Library of Medicine of the NIH under award number

R01LM1372201; the National Science Foundation under award numbers: CAREER-1841569 and TRIPODS-1740735.

Author's Contributions

D.K. and C.M. conceived the project and supervised the study. C.M. processed the raw data and designed model framework, C.M. and Z.Z. wrote code and trained models. C.M, Z.Z. and D.K. drafted the manuscript. All authors read and approved the final manuscript.

Acknowledgements

The authors thank Dr. Jared Roach from Institute for Systems Biology for his assistance in manually evaluating the biological realism of the model-predicted paths in a double-blind manner, and also thank the RTX-KG2 team (Stephen Ramsey, Amy Glen, E. C. Wood, Lili Acevedo) for their guidance in building RTX-KG2 and in resolving any issues that arose.

References

1. Berdigaliyev N, Aljofan M. An overview of drug discovery and development. Future Medicinal Chemistry 2020;12(10):939–947.
2. Miller MT. Thalidomide embryopathy: a model for the study of congenital incomitant horizontal strabismus. Transactions of the American Ophthalmological Society 1991;89:623–74.
3. Verheul HMW, Panigrahy D, Yuan J, D'Amato RJ. Combination oral antiangiogenic therapy with thalidomide and sulindac inhibits tumour growth in rabbits. British Journal of Cancer 1999;79(1):114–118.
4. Singhal S, Mehta J, Desikan R, Ayers D, Roberson P, Eddlemon P, et al. Antitumor Activity of Thalidomide in Refractory Multiple Myeloma. The New England Journal of Medicine 1999;341(21):1565–1571.
5. Kairys V, Baranauskiene L, Kazlauskiene M, Matulis D, Kazlauskas E. Binding affinity in drug design: experimental and computational techniques. Expert Opinion on Drug Discovery 2019;14(8):755–768.
6. Aulner N, Danckaert A, Ihm J, Shum D, Shorte SL. Next-Generation Phenotypic Screening in Early Drug Discovery for Infectious Diseases. Trends in Parasitology 2019;35(7):559–570.
7. Rusz CM, Ősz BE, Jîtcă G, Miklos A, Bătrînu MG, Imre S. Off-Label Medication: From a Simple Concept to Complex Practical Aspects. International Journal of Environmental Research and Public Health 2021;18(19):10447.
8. Swamidass SJ. Mining small-molecule screens to repurpose drugs. Briefings in Bioinformatics 2011;12(4):327–335.
9. Sanseau P, Agarwal P, Barnes MR, Pastinen T, Richards JB, Cardon LR, et al. Use of genome-wide association studies for drug repositioning. Nature Biotechnology 2012;30(4):317–320.
10. Bonner S, Barrett IP, Ye C, Swiers R, Engkvist O, Bender A, et al. A review of biomedical datasets relating to drug discovery: a knowledge graph perspective. Briefings in Bioinformatics 2022 sep;.
11. Wishart DS, Feunang YD, Guo AC, Lo EJ, Marcu A, Grant JR, et al. DrugBank 5.0: a major update to the DrugBank database for 2018. Nucleic Acids Research 2017;46(D1):gkx1037–.
12. Gaulton A, Bellis LJ, Bento AP, Chambers J, Davies M, Hersey A, et al. ChEMBL: a large-scale bioactivity database for drug discovery. Nucleic Acids Research 2012;40(D1):D1100–D1107.
13. Wishart DS, Feunang YD, Marcu A, Guo AC, Liang K, Vázquez-Fresno R, et al. HMDB 4.0: the human metabolome database for 2018. Nucleic Acids Research 2017;46(D1):gkx1089–.
14. Kanza S, Graham Frey J. Semantic Technologies in Drug Discovery. In: Wolkenhauer O, editor. Systems Medicine Oxford: Academic Press; 2021.p. 129–144.
15. Himmelstein DS, Lizee A, Hessler C, Brueggeman L, Chen SL, Hadley D, et al. Systematic integration of biomedical knowledge prioritizes drugs for repurposing. eLife 2017;6:e26726.
16. Walsh B, Mohamed SK, Nováček V. BioKG: A Knowledge Graph for Relational Learning On Biological Data. Proceedings of the 29th ACM International Conference on Information & Knowledge Management 2020;p. 3173–3180.
17. Su C, Hou Y, Guo W, Chaudhry F, Ghahramani G, Zhang H, et al. CBKH: The Cornell Biomedical Knowledge Hub. medRxiv 2021;.
18. Percha B, Altman RB. A global network of biomedical relationships derived from text. Bioinformatics 2018;34(15):2614–2624.
19. Zhang R, Hristovski D, Schutte D, Kastrin A, Fiszman M, Kilicoglu H. Drug repurposing for COVID-19 via knowledge graph completion. Journal of Biomedical Informatics 2021;115:103696.
20. Wang Q, Li M, Wang X, Parulian N, Han G, Ma J, et al. COVID-19 Literature Knowledge Graph Construction and Drug Repurposing Report Generation. Proceedings of the 2021 Conference of the North American Chapter of the Association for Computational Linguistics: Human Language Technologies: Demonstrations 2021;p. 66–77.
21. Li N, Yang Z, Luo L, Wang L, Zhang Y, Lin H, et al. KGHC: a knowledge graph for hepatocellular carcinoma. BMC Medical Informatics and Decision Making 2020;20(Suppl 3):135.
22. Santos A, Colaço AR, Nielsen AB, Niu L, Strauss M, Geyer PE, et al. A knowledge graph to interpret clinical proteomics data. Nature Biotechnology 2022;40(5):692–702.
23. Wood EC, Glen AK, Kvarfordt LG, Womack F, Acevedo L, Yoon TS, et al. RTX-KG2: a system for building a semantically standardized knowledge graph for translational biomedicine. BMC Bioinformatics 2022;23(1):400.
24. Ioannidis VN, Zheng D, Karypis G. Few-shot link prediction via graph neural networks for Covid-19 drug-repurposing. CoRR 2020;abs/2007.10261.
25. Bordes A, Usunier N, Garcia-Duran A, Weston J, Yakhnenko O. Translating Embeddings for Modeling Multi-relational Data. In: Neural Information Processing Systems (NIPS) South Lake Tahoe, United States; 2013. p. 1–9.
26. Sun Z, Deng ZH, Nie JY, Tang J. RotatE: Knowledge Graph Embedding by Relational Rotation in Complex Space. arXiv 2019;.
27. Yang B, Yih Wt, He X, Gao J, Deng L. Embedding Entities and Relations for Learning and Inference in Knowledge Bases. arXiv 2014;.
28. Trouillon T, Welbl J, Riedel S, Gaussier E, Bouchard G. Complex Embeddings for Simple Link Prediction. arXiv 2016;.
29. Wang B, Shen T, Long G, Zhou T, Wang Y, Chang Y. Structure-Augmented Text Representation Learning for Efficient Knowledge Graph Completion. Proceedings of the Web Conference 2021 2021;p. 1737–1748.
30. Sosa DN, Derry A, Guo M, Wei E, Brinton C, Altman RB. A Literature-Based Knowledge Graph Embedding Method for Identifying Drug Repurposing Opportunities in Rare Diseases. Pacific Symposium on Biocomputing Pacific Symposium on Biocomputing 2020;25:463–474.
31. Chen X, Chen M, Shi W, Sun Y, Zaniolo C. Embedding Uncertain Knowledge Graphs. Proceedings of the AAAI Conference on Artificial Intelligence 2019;33:3363–3370.
32. Sang S, Yang Z, Liu X, Wang L, Lin H, Wang J, et al. GrEDeL: A Knowledge Graph Embedding Based Method for Drug Discovery From Biomedical Literatures. IEEE Access 2019;7:8404–8415.
33. Kilicoglu H, Rosemblat G, Fiszman M, Shin D. Broad-coverage biomedical relation extraction with SemRep. BMC Bioinformatics 2020;21(1).

34. Li Y. Reinforcement Learning Applications. arXiv 2019;.
35. Chen L, Cui J, Tang X, Qian Y, Li Y, Zhang Y. RLPath: a knowledge graph link prediction method using reinforcement learning based attentive relation path searching and representation learning. Applied Intelligence 2022;52(4):4715–4726.
36. Sun Y, Wang S, Tang X, Hsieh TY, Honavar V. Adversarial Attacks on Graph Neural Networks via Node Injections: A Hierarchical Reinforcement Learning Approach. Proceedings of The Web Conference 2020 2020;p. 673–683.
37. Zhou X, Wang P, Luo Q, Pan Z. Multi-hop Knowledge Graph Reasoning Based on Hyperbolic Knowledge Graph Embedding and Reinforcement Learning. The 10th International Joint Conference on Knowledge Graphs 2021;p. 1–9.
38. Liu Y, Hildebrandt M, Joblin M, Ringsquandl M, Raissouni R, Tresp V. The Semantic Web. Lecture Notes in Computer Science 2021;.
39. Womack F, McClelland J, Koslicki D. Leveraging Distributed Biomedical Knowledge Sources to Discover Novel Uses for Known Drugs. bioRxiv 2019;p. 765305.
40. Hamilton WL, Ying R, Leskovec J. Inductive Representation Learning on Large Graphs. arXiv 2017;.
41. Zhao K, Wang X, Zhang Y, Zhao L, Liu Z, Xing C, et al. Leveraging Demonstrations for Reinforcement Recommendation Reasoning over Knowledge Graphs. Proceedings of the 43rd International ACM SIGIR Conference on Research and Development in Information Retrieval 2020;p. 239–248.
42. Mayers M, Steinecke D, Su AI, Database of mechanism of action paths for selected drug-disease indications. Zenodo; 2020. https://doi.org/10.5281/zenodo.3708278.
43. Degtyarenko K, Matos Pd, Ennis M, Hastings J, Zbinden M, McNaught A, et al. ChEBI: a database and ontology for chemical entities of biological interest. Nucleic Acids Research 2008;36(Database issue):D344–D350.
44. Consortium TBDT. Toward A Universal Biomedical Data Translator. Clinical and Translational Science 2019;12(2):86–90.
45. Translator Consortium. The Biomedical Data Translator Program: Conception, Culture, and Community. Clinical and Translational Science 2019 Mar;12(2):91–94.
46. Unni DR, Moxon SA, Bada M, Brush M, Bruskiewich R, Caufield JH, et al. Biolink Model: A universal schema for knowledge graphs in clinical, biomedical, and translational science. Clinical and Translational Science 2022;.
47. Xin J, Afrasiabi C, Lelong S, Adesara J, Tsueng G, Su AI, et al. Cross-linking BioThings APIs through JSON-LD to facilitate knowledge exploration. BMC Bioinformatics 2018;19(1):30.
48. Xin J, Mark A, Afrasiabi C, Tsueng G, Juchler M, Gopal N, et al. High-performance web services for querying gene and variant annotation. Genome Biology 2016;17(1):91.
49. Kilicoglu H, Shin D, Fiszman M, Rosemblat G, Rindflesch TC. SemMedDB: a PubMed-scale repository of biomedical semantic predications. Bioinformatics 2012;28(23):3158–3160.
50. Brown SH, Elkin PL, Rosenbloom ST, Husser C, Bauer BA, Lincoln MJ, et al. VA National Drug File Reference Terminology: a cross-institutional content coverage study. Studies in health technology and informatics 2004;107(Pt 1):477–81.
51. Brown AS, Patel CJ. A standard database for drug repositioning. Scientific Data 2017;4(1):170029.
52. Cilibrasi RL, Vitanyi PMB. The Google Similarity Distance. IEEE Transactions on Knowledge and Data Engineering 2007;19(3):370–383.
53. Mayers M, Tu R, Steinecke D, Li TS, Queralt-Rosinach N, Su AI. Design and application of a knowledge network for automatic prioritization of drug mechanisms. Bioinformatics 2022;38(10):btac205.
54. Gu Y, Tinn R, Cheng H, Lucas M, Usuyama N, Liu X, et al. Domain-Specific Language Model Pretraining for Biomedical Natural Language Processing. ACM Transactions on Computing for Healthcare 2022;3(1):1–23.
55. Mikolov T, Sutskever I, Chen K, Corrado G, Dean J, Distributed Representations of Words and Phrases and their Compositionality. arXiv; 2013.
56. Lillicrap TP, Hunt JJ, Pritzel A, Heess N, Erez T, Tassa Y, et al. Continuous control with deep reinforcement learning. arXiv 2015;.
57. Sutton RS. Learning to predict by the methods of temporal differences. Machine Learning 1988;3(1):9–44.
58. Williams RJ. Simple Statistical Gradient-Following Algorithms for Connectionist Reinforcement Learning. Machine Learning 1992;8(3-4):229–256.
59. PomerleauDean A. Efficient training of artificial neural networks for autonomous navigation. Neural Computation 1991;.
60. Hsieh K, Wang Y, Chen L, Zhao Z, Savitz S, Jiang X, et al. Drug repurposing for COVID-19 using graph neural network and harmonizing multiple evidence. arXiv 2020;.
61. Lin Y, Liu Z, Sun M, Liu Y, Zhu X. Learning entity and relation embeddings for knowledge graph completion. In: In Proceedings of AAAI'15; 2015. .
62. Liu H, Wu Y, Yang Y, Analogical Inference for Multi-Relational Embeddings; 2017.
63. Kazemi SM, Poole D, SimplE Embedding for Link Prediction in Knowledge Graphs; 2018.
64. Veličković P, Cucurull G, Casanova A, Romero A, Liò P, Bengio Y. Graph Attention Networks. International Conference on Learning Representations 2018;Accepted as poster.
65. Glorot X, Bengio Y. Understanding the difficulty of training deep feedforward neural networks. In: Teh YW, Titterington DM, editors. AISTATS, vol. 9 of JMLR Proceedings JMLR.org; 2010. p. 249–256.
66. Lin XV, Socher R, Xiong C. Multi-Hop Knowledge Graph Reasoning with Reward Shaping. Proceedings of the 2018 Conference on Empirical Methods in Natural Language Processing 2018;p. 3243–3253.
67. Croom KF, McCormack PL. Recombinant factor VIIa (eptacog alfa): a review of its use in congenital hemophilia with inhibitors, acquired hemophilia, and other congenital bleeding disorders. BioDrugs : clinical immunotherapeutics, biopharmaceuticals and gene therapy 2008;22(2):121–36.
68. Minno GD. Eptacog alfa activated: a recombinant product to treat rare congenital bleeding disorders. Blood Reviews 2015;29:S26–S33.
69. Rendo P, Smith L, Lee HY, Shafer F. Nonacog alfa: an analysis of safety data from six prospective clinical studies in different patient populations with haemophilia B treated with different therapeutic modalities. Blood coagulation & fibrinolysis : an international journal in haemostasis and thrombosis 2015;26(8):912–8.
70. Driessche T, Collen D, Chuah M. Viral Vector-Mediated Gene Therapy for Hemophilia. Current Gene Therapy 2001;1(3):301–315.
71. Roberts HR, Monroe DM, White GC. The use of recombinant factor VIIa in the treatment of bleeding disorders. Blood 2004;104(13):3858–3864.
72. Negrier C, Shima M, Hoffman M. The central role of thrombin in bleeding disorders. Blood Reviews 2019;38:100582.
73. Goodeve AC. Hemophilia B: molecular pathogenesis and mutation analysis. Journal of Thrombosis and Haemostasis 2015;13(7):1184–1195.
74. Sourimant J, Aggarwal M, Plemper RK. Progress and pitfalls of a year of drug repurposing screens against COVID-19. Current Opinion in Virology 2021;49:183–193.
75. Gysi DM, do Valle Í , Zitnik M, Ameli A, Gan X, Varol O, et al. Network medicine framework for identifying drug-repurposing opportunities for COVID-19. Proceedings of the National Academy of Sciences of the United States of America 2021;118(19):e2025581118.
76. Yohn CN, Gergues MM, Samuels BA. The role of 5-HT receptors

in depression. Molecular Brain 2017;10(1):28.
77. Delva NC, Stanwood GD. Dysregulation of brain dopamine systems in major depressive disorder. Experimental Biology and Medicine 2021;246(9):1084–1093.
78. Coppen EM, Roos RAC. Current Pharmacological Approaches to Reduce Chorea in Huntington's Disease. Drugs 2017;77(1):29–46.
79. Shukla S, Tekwani BL. Histone Deacetylases Inhibitors in Neurodegenerative Diseases, Neuroprotection and Neuronal Differentiation. Frontiers in Pharmacology 2020;11:537.
80. Yu IT, Park JY, Kim SH, Lee Js, Kim YS, Son H. Valproic acid promotes neuronal differentiation by induction of proneural factors in association with H4 acetylation. Neuropharmacology 2009;56(2):473–480.
81. Voronin MV, Kadnikov IA, Zainullina LF, Logvinov IO, Verbovaya ER, Antipova TA, et al. Neuroprotective Properties of Quinone Reductase 2 Inhibitor M-11, a 2-Mercaptobenzimidazole Derivative. International Journal of Molecular Sciences 2021;22(23).
82. Singh S, Singh TG. Role of Nuclear Factor Kappa B (NF-KB) Signalling in Neurodegenerative Diseases: An Mechanistic Approach. Current Neuropharmacology 2020;18(10):918–935.
83. Yagami T, Kohma H, Yamamoto Y. L-type voltage-dependent calcium channels as therapeutic targets for neurodegenerative diseases. Current medicinal chemistry 2012;19(28):4816–27.
84. Noh W, Pak S, Choi G, Yang S, Yang S. Transient Potassium Channels: Therapeutic Targets for Brain Disorders. Frontiers in Cellular Neuroscience 2019;13:265.
85. Arena R, Iudice A, Virgili P, Moretti P, Menchetti G. Huntington's disease: clinical effects of a short-term treatment with pimozide. Advances in biochemical psychopharmacology 1980;24:573–5.
86. Videnovic A. Treatment of huntington disease. Current treatment options in neurology 2013;15(4):424–38.
87. Paleacu D, Anca M, Giladi N. Olanzapine in Huntington's disease: Olanzapine in Huntington's disease. Acta Neurologica Scandinavica 2002;105(6):441–444.
88. Squitieri F, Cannella M, Porcellini A, Brusa L, Simonelli M, Ruggieri S. Short-term effects of olanzapine in Huntington disease. Neuropsychiatry, neuropsychology, and behavioral neurology 2001;14(1):69–72.
89. Group HS. Dosage effects of riluzole in Huntington's disease: a multicenter placebo-controlled study. Neurology 2003;61(11):1551–6.
90. Unti E, Mazzucchi S, Palermo G, Bonuccelli U, Ceravolo R. Antipsychotic drugs in Huntington's disease. Expert Review of Neurotherapeutics 2017;17(3):227–237.
91. Duff K, Beglinger LJ, O'Rourke ME, Nopoulos P, Paulson HL, Paulsen JS. Risperidone and the Treatment of Psychiatric, Motor, and Cognitive Symptoms in Huntington's Disease. Annals of Clinical Psychiatry 2008;.
92. Miranda AS, Cardozo PL, Silva FR, Souza JMd, Olmo IG, Cruz JS, et al. Alterations of Calcium Channels in a Mouse Model of Huntington's Disease and Neuroprotection by Blockage of CaV1 Channels. ASN NEURO 2019;11:1759091419856811.

Figure1 in Main Text

Click here to access/download;Figure;main_figure1.jpg

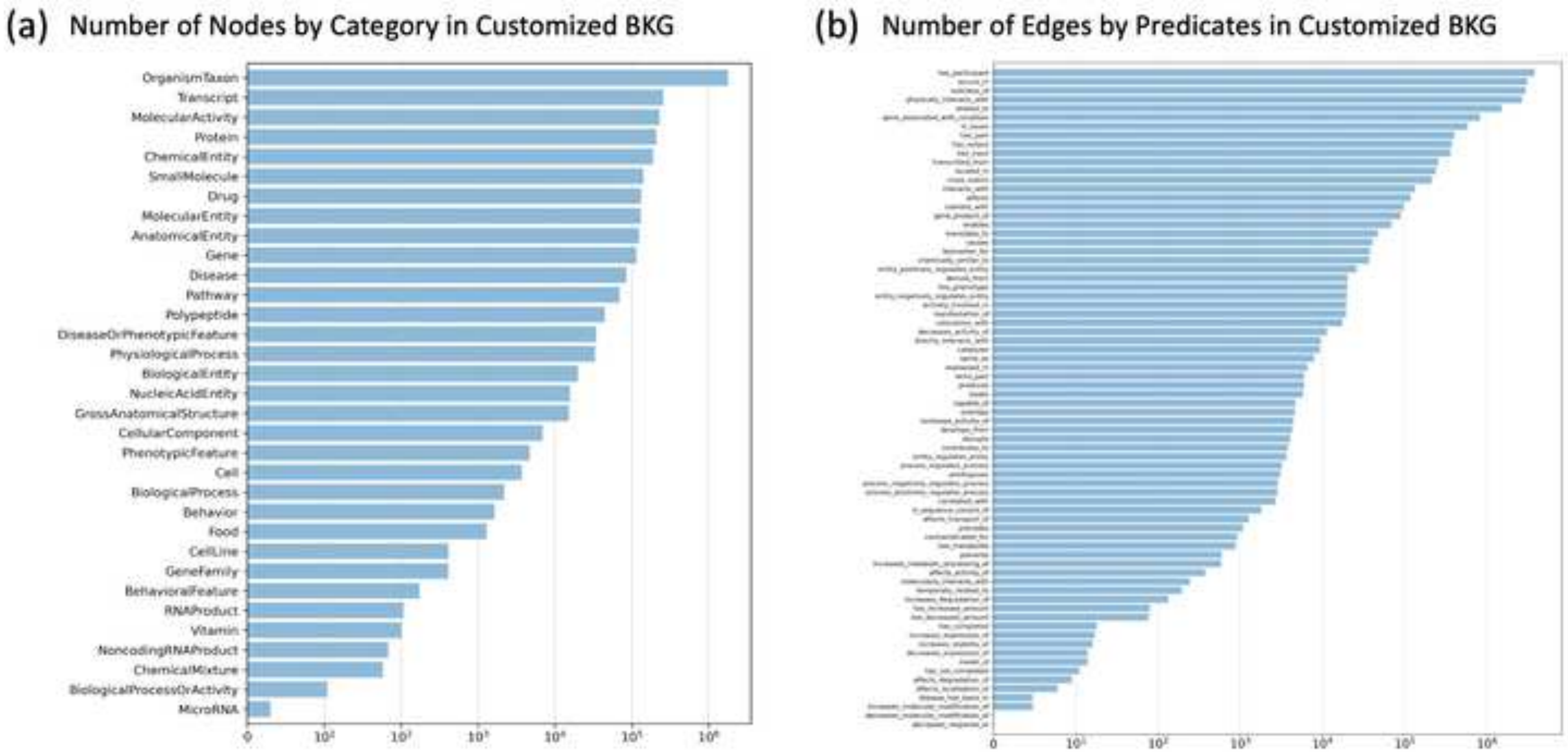

 Click here to access/download;Figure;main_figure2.jpg

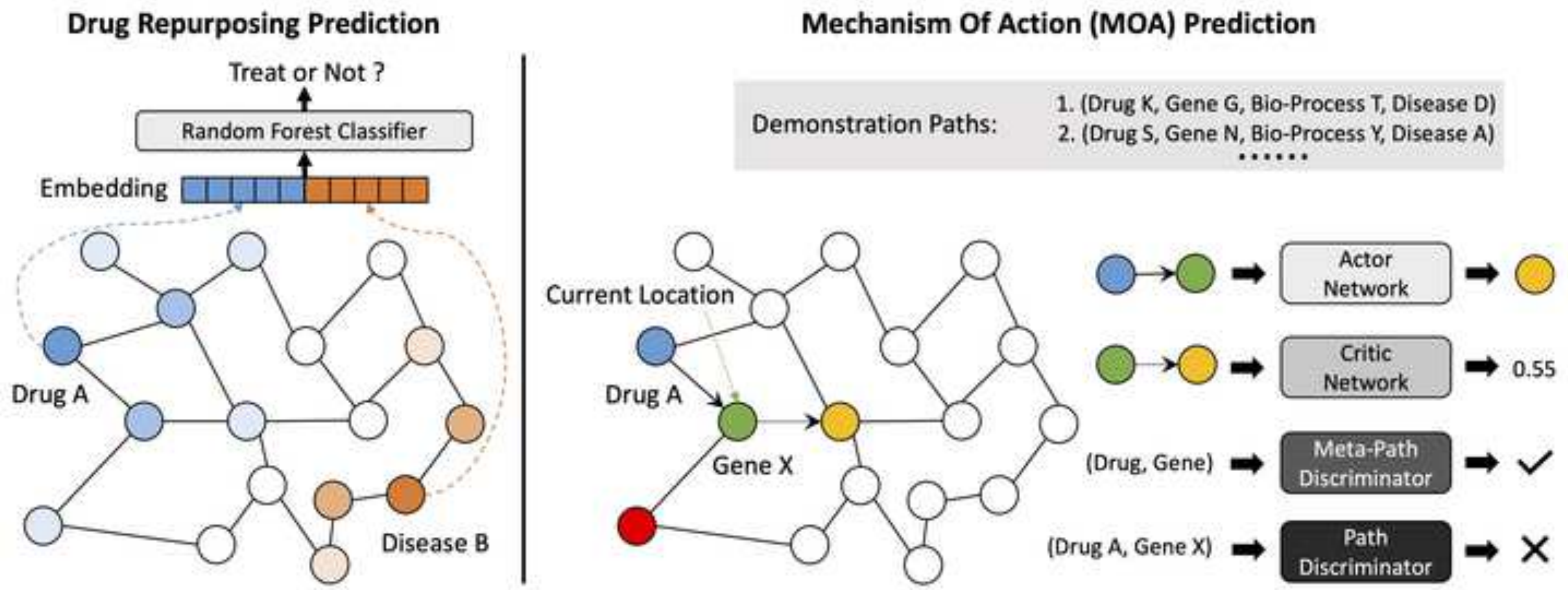

Figure3 in Main Text

Click here to access/download;Figure;main_figure3.jpg

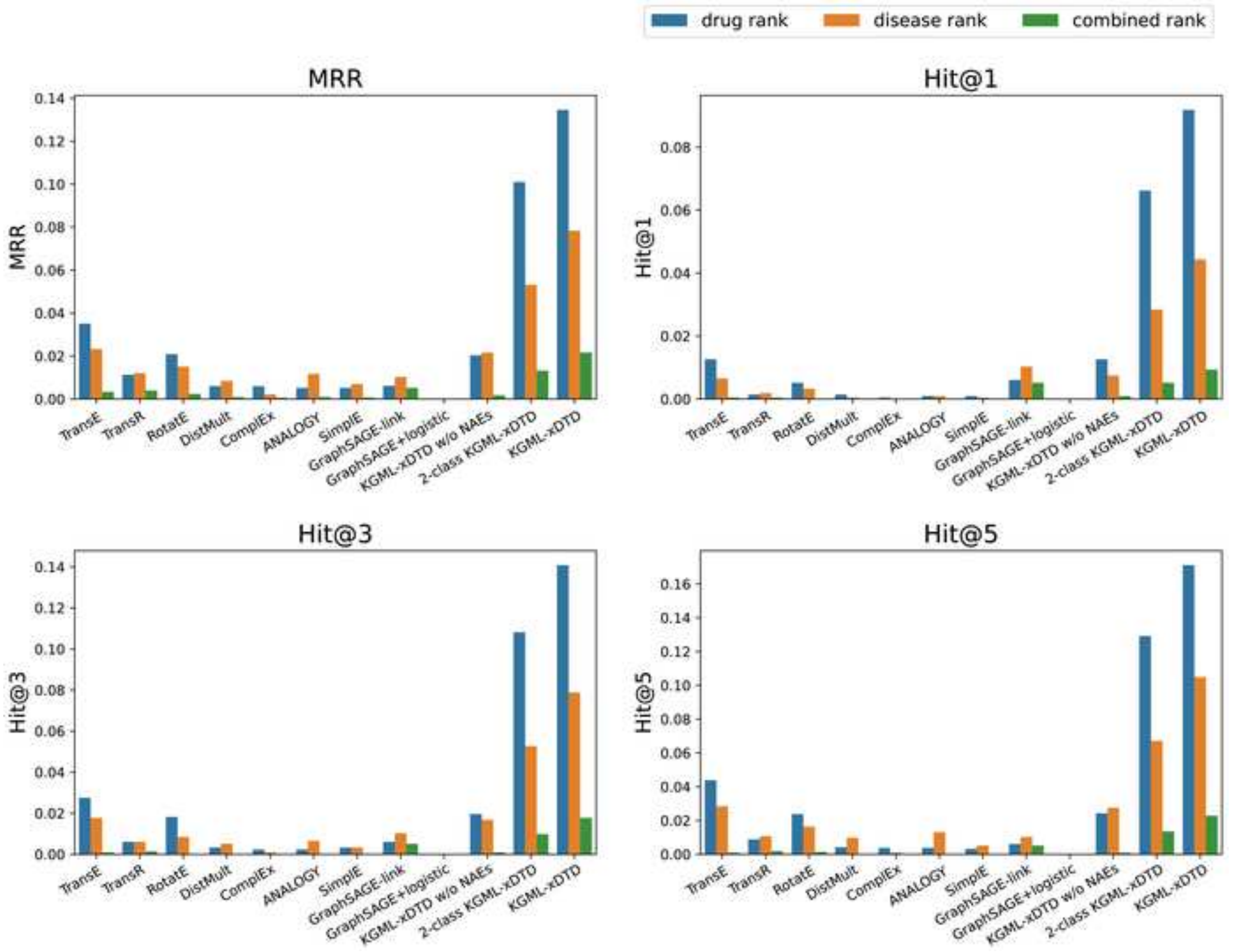

Figure4 in Main Text

Click here to access/download;Figure;main_figure4.jpg

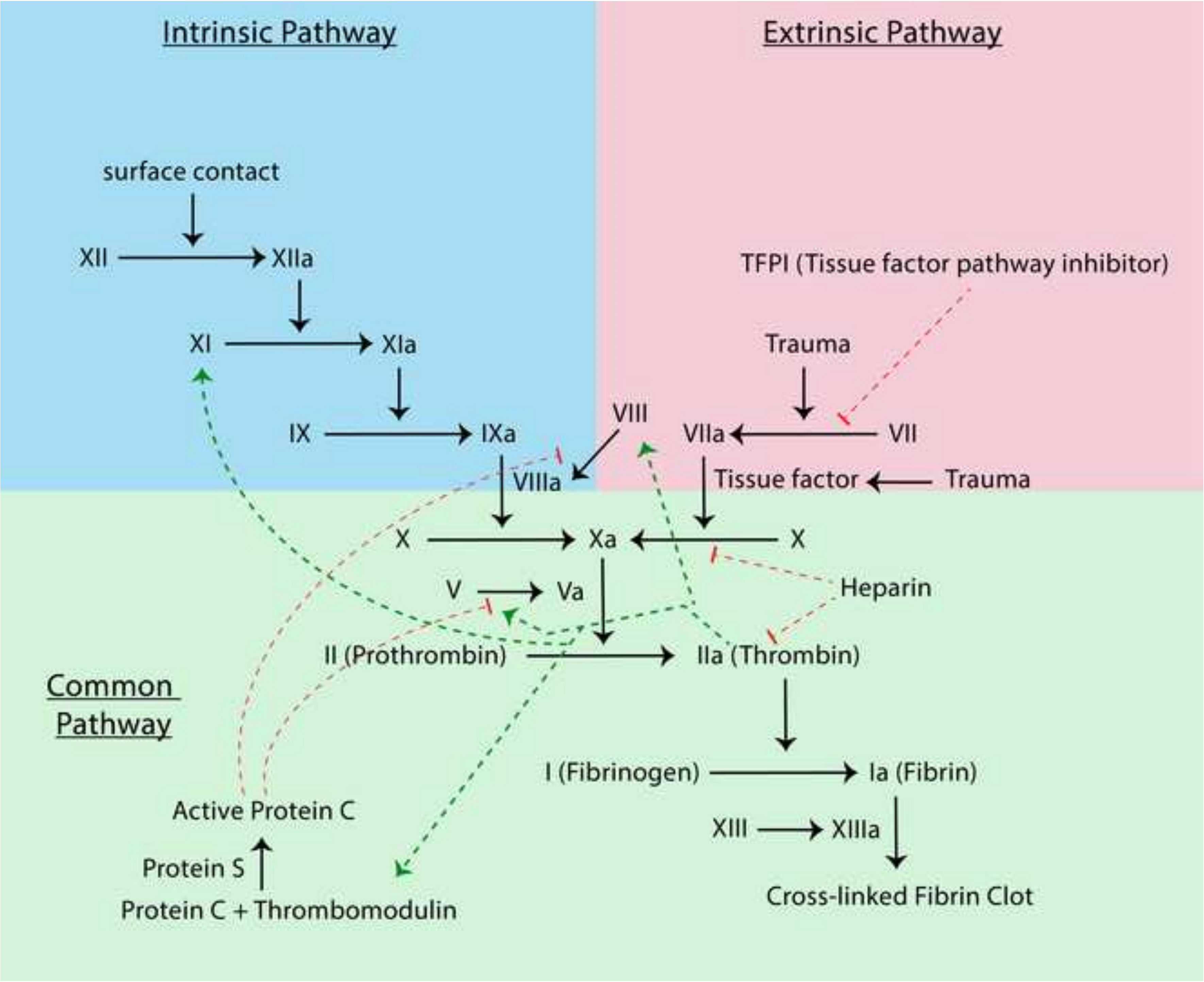

Figure5 in Main Text
Click here to access/download;Figure;main_figure5.jpg

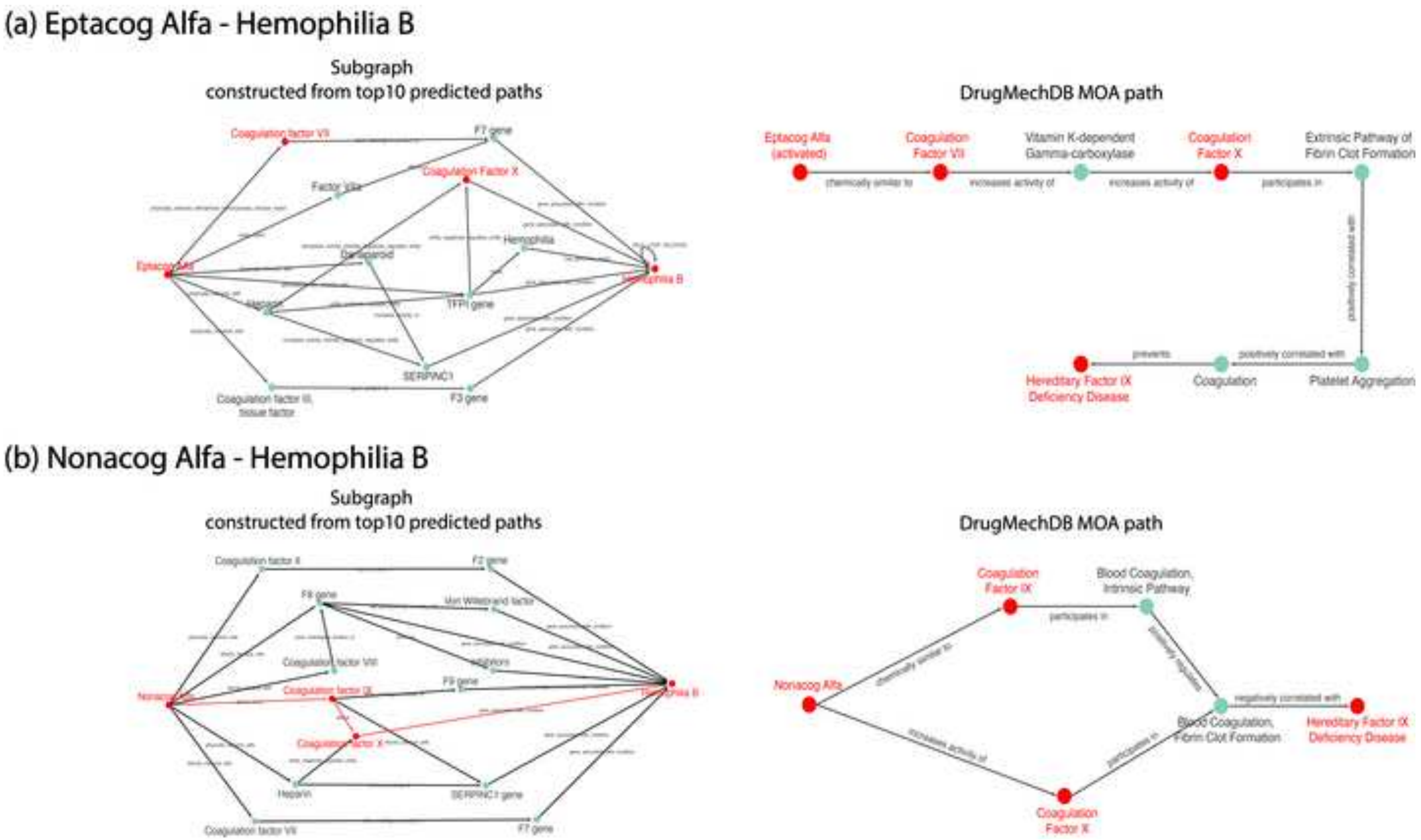

Figure6 in Main Text
Click here to access/download;Figure;main_figure6.jpg

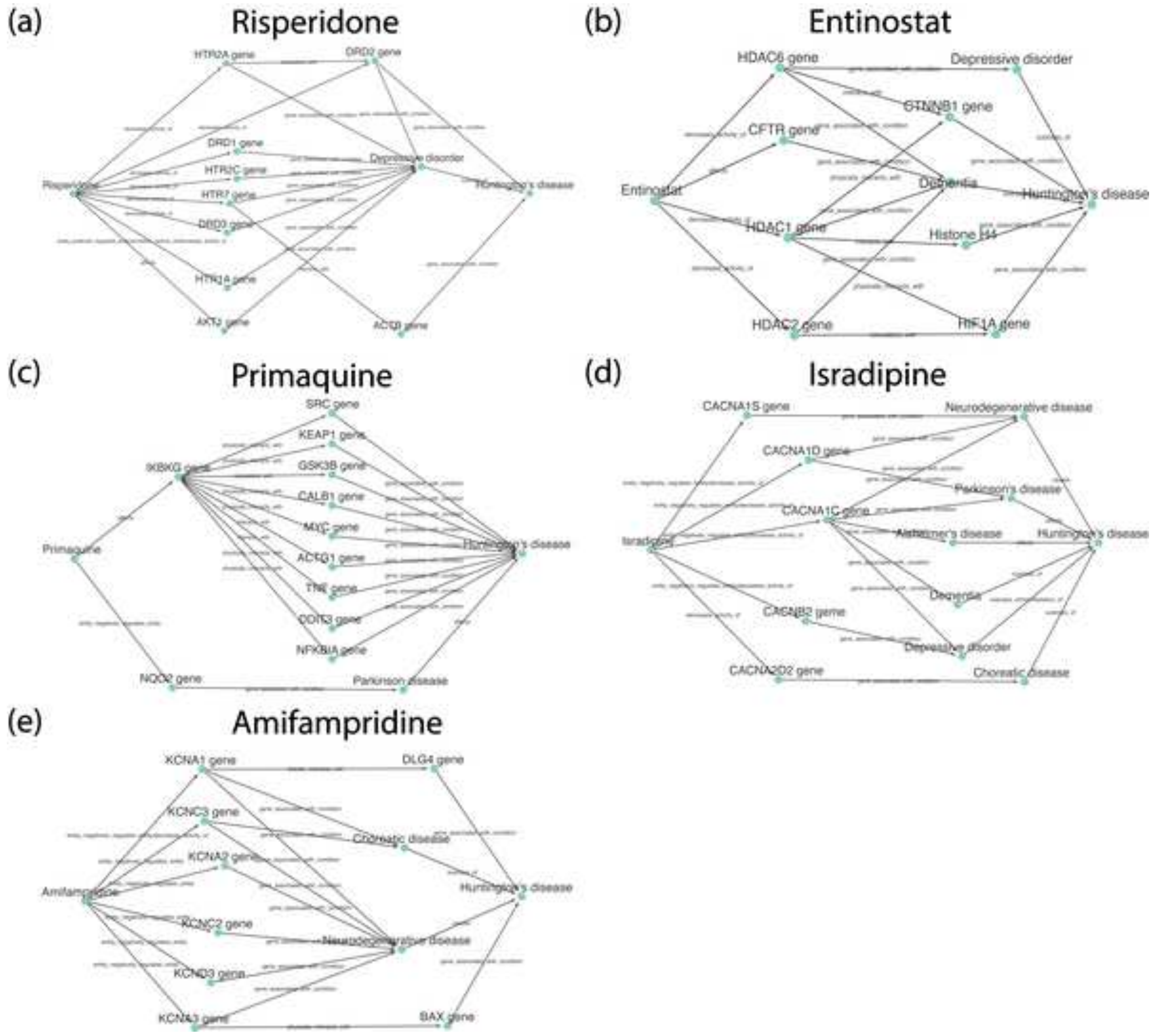

Figure1 in Supplementary Material
Click here to access/download;Figure;suppl_figure1.jpg

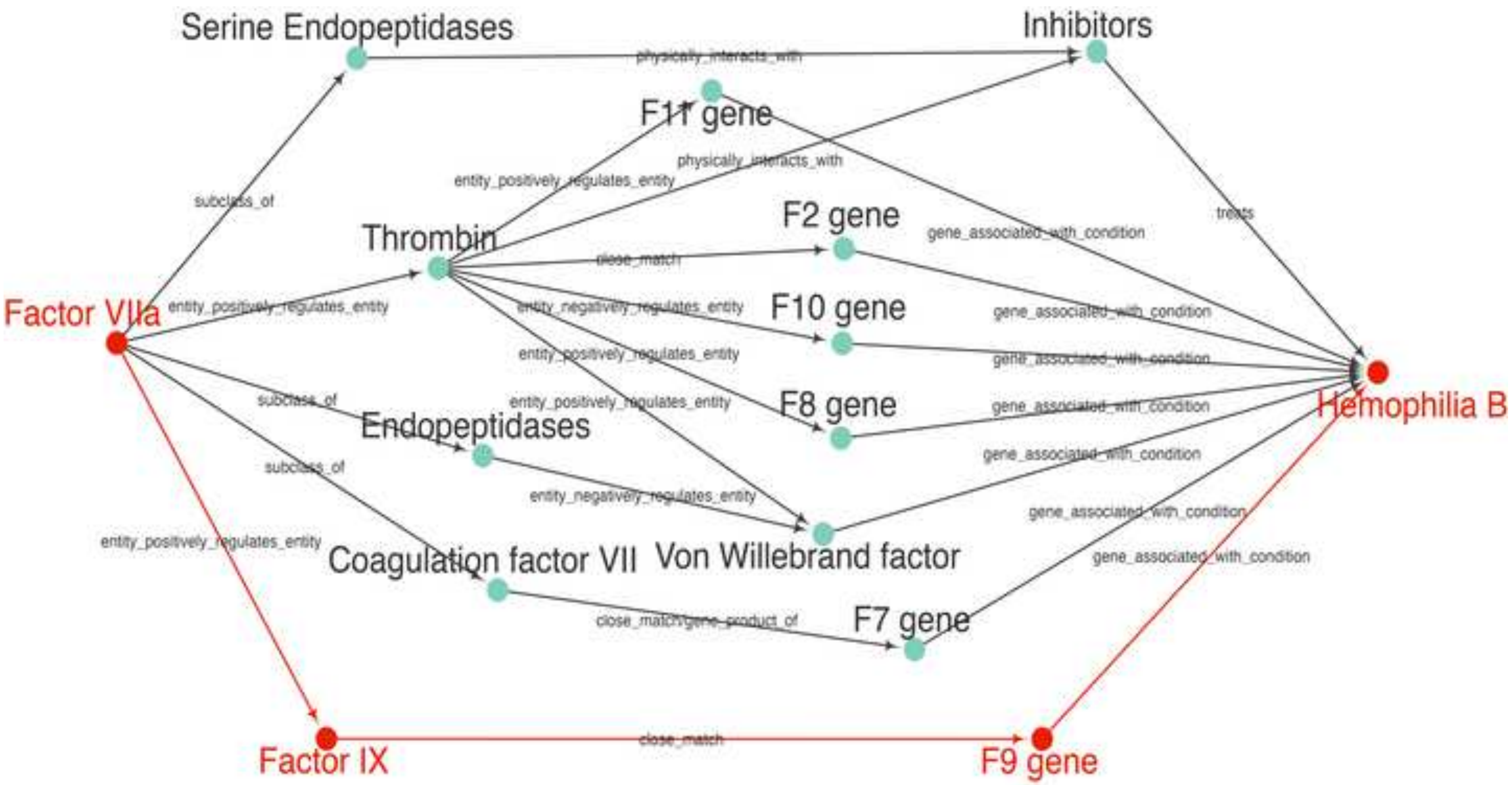

Supplementary Sections

Click here to access/download

Supplementary Material

SI_Supplementary Material.pdf
