# Supplementary material for: KGML-xDTD: a knowledge graph–based machine learning framework for drug treatment prediction and mechanism description
Source: Gigascience. 2023 Aug 21;12:giad057. doi: 10.1093/gigascience/giad057 (PMC10441000; doi:10.1093/gigascience/giad057)
Supplement: giad057_GIGA-D-23-00026_Revision_3 [file giad057_giga-d-23-00026_revision_3.pdf]

GigaScience

KGML-xDTD: A Knowledge Graph-based Machine Learning Framework for Drug Treatment Prediction and Mechanism Description

--Manuscript Draft--

**Manuscript Number:**	GIGA-D-23-00026R3
**Full Title:**	KGML-xDTD: A Knowledge Graph-based Machine Learning Framework for Drug Treatment Prediction and Mechanism Description
**Article Type:**	Research
**Funding Information:**	

Funder	Recipient
National Center for Advancing Translational Sciences (OT2-TR003428-01S3)	Dr. David Koslicki
National Center for Advancing Translational Sciences (OT2-TR003428-01S2)	Dr. David Koslicki
National Center for Advancing Translational Sciences (OT2-TR003428-01)	Dr. David Koslicki
U.S. National Library of Medicine (R01LM01372201)	Dr. Han Liu
National Science Foundation (CAREER-1841569)	Dr. Han Liu

Abstract:

Background: Computational drug repurposing is a cost- and time-efficient approach that aims to identify new therapeutic targets or diseases (indications) of existing drugs/compounds. It is especially critical for emerging and/or orphan diseases due to its cheaper investment and shorter research cycle compared with traditional wet-lab drug discovery approaches. However, the underlying mechanisms of action (MOAs) between repurposed drugs and their target diseases remain largely unknown, which is still a main obstacle for computational drug repurposing methods to be widely adopted in clinical settings.
Results: In this work, we propose KGML-xDTD: a Knowledge Graph-based Machine Learning framework for explainably predicting Drugs Treating Diseases. It is a two-module framework that not only predicts the treatment probabilities between drugs/compounds and diseases but also biologically explains them via knowledge graph (KG) path-based, testable mechanisms of action (MOAs). We leverage knowledge-and-publication based information to extract biologically meaningful "demonstration paths" as the intermediate guidance in the Graph-based Reinforcement Learning (GRL) path-finding process. Comprehensive experiments and case study analyses show that the proposed framework can achieve state-of-the-art performance in both predictions of drug repurposing and recapitulation of human-curated drug MOA paths.
Conclusions: KGML-xDTD is the first model framework that can offer KG-path explanations for drug repurposing predictions by leveraging the combination of prediction outcomes and existing biological knowledge and publications. We believe it can effectively reduce "black-box" concerns and increase prediction confidence for drug repurposing based on predicted path-based explanations, and further accelerate the process of drug discovery for emerging diseases.

**Corresponding Author:**	Chunyu Ma<br>Pennsylvania State University - Main Campus: The Pennsylvania State University - University Park Campus<br>State College, PA UNITED STATES
**Corresponding Author Secondary Information:**	
**Corresponding Author's Institution:**	Pennsylvania State University - Main Campus: The Pennsylvania State University - University Park Campus
**Corresponding Author's Secondary Institution:**	
**First Author:**	Chunyu Ma

Powered by Editorial Manager® and ProduXion Manager® from Aries Systems Corporation

First Author Secondary Information:	
Order of Authors:	Chunyu Ma
	Zhihan Zhou
	Han Liu
	David Koslicki
Order of Authors Secondary Information:	
Response to Reviewers:	We have resolved all minor issues.
Additional Information:	
**Question**	**Response**
Are you submitting this manuscript to a special series or article collection?	No
**Experimental design and statistics**<br><br>Full details of the experimental design and statistical methods used should be given in the Methods section, as detailed in our Minimum Standards Reporting Checklist. Information essential to interpreting the data presented should be made available in the figure legends.<br><br>Have you included all the information requested in your manuscript?	Yes
**Resources**<br><br>A description of all resources used, including antibodies, cell lines, animals and software tools, with enough information to allow them to be uniquely identified, should be included in the Methods section. Authors are strongly encouraged to cite Research Resource Identifiers (RRIDs) for antibodies, model organisms and tools, where possible.<br><br>Have you included the information requested as detailed in our Minimum Standards Reporting Checklist?	Yes
**Availability of data and materials**<br><br>All datasets and code on which the	Yes

Powered by Editorial Manager® and ProduXion Manager® from Aries Systems Corporation

conclusions of the paper rely must be either included in your submission or deposited in publicly available repositories (where available and ethically appropriate), referencing such data using a unique identifier in the references and in the "Availability of Data and Materials" section of your manuscript.<br><br>Have you have met the above requirement as detailed in our Minimum Standards Reporting Checklist?	

Powered by Editorial Manager® and ProduXion Manager® from Aries Systems Corporation

LaTeX - Primary Manuscript File

Click here to access/download;LaTeX - Main Document (TeX file);main.tex

```
This is pdfTeX, Version 3.141592653-2.6-1.40.24 (TeX Live 2022)
(preloaded format=pdflatex 2023.3.8)  3 JUL 2023 03:44
entering extended mode
 restricted \write18 enabled.
 %&-line parsing enabled.
**main.tex
(./main.tex
LaTeX2e <2022-11-01> patch level 1
L3 programming layer <2023-02-22> (./oup-contemporary.cls
Document Class: oup-contemporary 2017/06/28, v1.1
(c:/TeXLive/2022/texmf-dist/tex/latex/base/article.cls
Document Class: article 2022/07/02 v1.4n Standard LaTeX document class
(c:/TeXLive/2022/texmf-dist/tex/latex/base/size10.clo
File: size10.clo 2022/07/02 v1.4n Standard LaTeX file (size option)
)
\c@part=\count185
\c@section=\count186
\c@subsection=\count187
\c@subsubsection=\count188
\c@paragraph=\count189
\c@subparagraph=\count190
\c@figure=\count191
\c@table=\count192
\abovecaptionskip=\skip48
\belowcaptionskip=\skip49
\bibindent=\dimen140
) (c:/TeXLive/2022/texmf-dist/tex/latex/base/inputenc.sty
Package: inputenc 2021/02/14 v1.3d Input encoding file
\inpenc@prehook=\toks16
\inpenc@posthook=\toks17
) (c:/TeXLive/2022/texmf-dist/tex/latex/base/fontenc.sty
Package: fontenc 2021/04/29 v2.0v Standard LaTeX package
) (c:/TeXLive/2022/texmf-dist/tex/generic/iftex/ifpdf.sty
Package: ifpdf 2019/10/25 v3.4 ifpdf legacy package. Use iftex instead.
(c:/TeXLive/2022/texmf-dist/tex/generic/iftex/iftex.sty
Package: iftex 2022/02/03 v1.0f TeX engine tests
)) (c:/TeXLive/2022/texmf-dist/tex/latex/microtype/microtype.sty
Package: microtype 2023/03/13 v3.1a Micro-typographical refinements (RS)
(c:/TeXLive/2022/texmf-dist/tex/latex/graphics/keyval.sty
Package: keyval 2022/05/29 v1.15 key=value parser (DPC)
\KV@toks@=\toks18
) (c:/TeXLive/2022/texmf-dist/tex/latex/etoolbox/etoolbox.sty
Package: etoolbox 2020/10/05 v2.5k e-TeX tools for LaTeX (JAW)
\etb@tempcnta=\count193
)
\MT@toks=\toks19
\MT@tempbox=\box51
\MT@count=\count194
LaTeX Info: Redefining \noprotrusionifhmode on input line 1059.
LaTeX Info: Redefining \leftprotrusion on input line 1060.
\MT@prot@toks=\toks20
LaTeX Info: Redefining \rightprotrusion on input line 1078.
LaTeX Info: Redefining \textls on input line 1368.
\MT@outer@kern=\dimen141
```

```
LaTeX Info: Redefining \textmicrotypecontext on input line 1988.
\MT@listname@count=\count195
(c:/TeXLive/2022/texmf-dist/tex/latex/microtype/microtype-pdftex.def
File: microtype-pdftex.def 2023/03/13 v3.1a Definitions specific to
pdftex (RS)

LaTeX Info: Redefining \lsstyle on input line 902.
LaTeX Info: Redefining \lslig on input line 902.
\MT@outer@space=\skip50
)
Package microtype Info: Loading configuration file microtype.cfg.
(c:/TeXLive/2022/texmf-dist/tex/latex/microtype/microtype.cfg
File: microtype.cfg 2023/03/13 v3.1a microtype main configuration file
(RS)
)) (c:/TeXLive/2022/texmf-dist/tex/latex/euler/euler.sty
Package: euler 1995/03/05 v2.5
Package: `euler' v2.5 <1995/03/05> (FJ and FMi)
LaTeX Font Info:    Redeclaring symbol font `letters' on input line 35.
LaTeX Font Info:    Encoding `OML' has changed to `U' for symbol font
(Font)              `letters' in the math version `normal' on input line
35.
LaTeX Font Info:    Overwriting symbol font `letters' in version `normal'
(Font)                  OML/cmm/m/it --> U/eur/m/n on input line 35.
LaTeX Font Info:    Encoding `OML' has changed to `U' for symbol font
(Font)              `letters' in the math version `bold' on input line
35.
LaTeX Font Info:    Overwriting symbol font `letters' in version `bold'
(Font)                  OML/cmm/b/it --> U/eur/m/n on input line 35.
LaTeX Font Info:    Overwriting symbol font `letters' in version `bold'
(Font)                  U/eur/m/n --> U/eur/b/n on input line 36.
LaTeX Font Info:    Redeclaring math symbol \Gamma on input line 47.
LaTeX Font Info:    Redeclaring math symbol \Delta on input line 48.
LaTeX Font Info:    Redeclaring math symbol \Theta on input line 49.
LaTeX Font Info:    Redeclaring math symbol \Lambda on input line 50.
LaTeX Font Info:    Redeclaring math symbol \Xi on input line 51.
LaTeX Font Info:    Redeclaring math symbol \Pi on input line 52.
LaTeX Font Info:    Redeclaring math symbol \Sigma on input line 53.
LaTeX Font Info:    Redeclaring math symbol \Upsilon on input line 54.
LaTeX Font Info:    Redeclaring math symbol \Phi on input line 55.
LaTeX Font Info:    Redeclaring math symbol \Psi on input line 56.
LaTeX Font Info:    Redeclaring math symbol \Omega on input line 57.
\symEulerFraktur=\mathgroup4
LaTeX Font Info:    Overwriting symbol font `EulerFraktur' in version
`bold'
(Font)                  U/euf/m/n --> U/euf/b/n on input line 63.
LaTeX Info: Redefining \oldstylenums on input line 85.
\symEulerScript=\mathgroup5
LaTeX Font Info:    Overwriting symbol font `EulerScript' in version
`bold'
(Font)                  U/eus/m/n --> U/eus/b/n on input line 93.
LaTeX Font Info:    Redeclaring math symbol \aleph on input line 97.
LaTeX Font Info:    Redeclaring math symbol \Re on input line 98.
LaTeX Font Info:    Redeclaring math symbol \Im on input line 99.
LaTeX Font Info:    Redeclaring math delimiter \vert on input line 101.
```

```
LaTeX Font Info:    Redeclaring math delimiter \backslash on input line
103.
LaTeX Font Info:    Redeclaring math symbol \neg on input line 106.
LaTeX Font Info:    Redeclaring math symbol \wedge on input line 108.
LaTeX Font Info:    Redeclaring math symbol \vee on input line 110.
LaTeX Font Info:    Redeclaring math symbol \setminus on input line 112.
LaTeX Font Info:    Redeclaring math symbol \sim on input line 113.
LaTeX Font Info:    Redeclaring math symbol \mid on input line 114.
LaTeX Font Info:    Redeclaring math delimiter \arrowvert on input line
116.
LaTeX Font Info:    Redeclaring math symbol \mathsection on input line
117.
\symEulerExtension=\mathgroup6
LaTeX Font Info:    Redeclaring math symbol \coprod on input line 125.
LaTeX Font Info:    Redeclaring math symbol \prod on input line 125.
LaTeX Font Info:    Redeclaring math symbol \sum on input line 125.
LaTeX Font Info:    Redeclaring math symbol \intop on input line 130.
LaTeX Font Info:    Redeclaring math symbol \ointop on input line 131.
LaTeX Font Info:    Redeclaring math symbol \braceld on input line 132.
LaTeX Font Info:    Redeclaring math symbol \bracerd on input line 133.
LaTeX Font Info:    Redeclaring math symbol \bracelu on input line 134.
LaTeX Font Info:    Redeclaring math symbol \braceru on input line 135.
LaTeX Font Info:    Redeclaring math symbol \infty on input line 136.
LaTeX Font Info:    Redeclaring math symbol \nearrow on input line 153.
LaTeX Font Info:    Redeclaring math symbol \searrow on input line 154.
LaTeX Font Info:    Redeclaring math symbol \nwarrow on input line 155.
LaTeX Font Info:    Redeclaring math symbol \swarrow on input line 156.
LaTeX Font Info:    Redeclaring math symbol \Leftrightarrow on input line
157.
LaTeX Font Info:    Redeclaring math symbol \Leftarrow on input line 158.
LaTeX Font Info:    Redeclaring math symbol \Rightarrow on input line
159.
LaTeX Font Info:    Redeclaring math symbol \leftrightarrow on input line
160.
LaTeX Font Info:    Redeclaring math symbol \leftarrow on input line 161.
LaTeX Font Info:    Redeclaring math symbol \rightarrow on input line
163.
LaTeX Font Info:    Redeclaring math delimiter \uparrow on input line
166.
LaTeX Font Info:    Redeclaring math delimiter \downarrow on input line
168.
LaTeX Font Info:    Redeclaring math delimiter \updownarrow on input line
170.
LaTeX Font Info:    Redeclaring math delimiter \Uparrow on input line
172.
LaTeX Font Info:    Redeclaring math delimiter \Downarrow on input line
174.
LaTeX Font Info:    Redeclaring math delimiter \Updownarrow on input line
176.
LaTeX Font Info:    Redeclaring math symbol \leftharpoonup on input line
177.
LaTeX Font Info:    Redeclaring math symbol \leftharpoondown on input
line 178.
```

```
LaTeX Font Info:    Redeclaring math symbol \rightharpoonup on input line
179.
LaTeX Font Info:    Redeclaring math symbol \rightharpoondown on input
line 180
.
LaTeX Font Info:    Redeclaring math delimiter \lbrace on input line 182.
LaTeX Font Info:    Redeclaring math delimiter \rbrace on input line 184.
\symcmmigroup=\mathgroup7
LaTeX Font Info:    Overwriting symbol font `cmmigroup' in version `bold'
(Font)                  OML/cmm/m/it --> OML/cmm/b/it on input line 200.
LaTeX Font Info:    Redeclaring math accent \vec on input line 201.
LaTeX Font Info:    Redeclaring math symbol \triangleleft on input line
202.
LaTeX Font Info:    Redeclaring math symbol \triangleright on input line
203.
LaTeX Font Info:    Redeclaring math symbol \star on input line 204.
LaTeX Font Info:    Redeclaring math symbol \lhook on input line 205.
LaTeX Font Info:    Redeclaring math symbol \rhook on input line 206.
LaTeX Font Info:    Redeclaring math symbol \flat on input line 207.
LaTeX Font Info:    Redeclaring math symbol \natural on input line 208.
LaTeX Font Info:    Redeclaring math symbol \sharp on input line 209.
LaTeX Font Info:    Redeclaring math symbol \smile on input line 210.
LaTeX Font Info:    Redeclaring math symbol \frown on input line 211.
LaTeX Font Info:    Redeclaring math accent \grave on input line 245.
LaTeX Font Info:    Redeclaring math accent \acute on input line 246.
LaTeX Font Info:    Redeclaring math accent \tilde on input line 247.
LaTeX Font Info:    Redeclaring math accent \ddot on input line 248.
LaTeX Font Info:    Redeclaring math accent \check on input line 249.
LaTeX Font Info:    Redeclaring math accent \breve on input line 250.
LaTeX Font Info:    Redeclaring math accent \bar on input line 251.
LaTeX Font Info:    Redeclaring math accent \dot on input line 252.
LaTeX Font Info:    Redeclaring math accent \hat on input line 254.
) (c:/TeXLive/2022/texmf-dist/tex/latex/merriweather/merriweather.sty
Package: merriweather 2022/09/20 (Bob Tennent) Supports
Merriweather(Sans) font
s for all LaTeX engines.
(c:/TeXLive/2022/texmf-dist/tex/generic/iftex/ifxetex.sty
Package: ifxetex 2019/10/25 v0.7 ifxetex legacy package. Use iftex
instead.
) (c:/TeXLive/2022/texmf-dist/tex/generic/iftex/ifluatex.sty
Package: ifluatex 2019/10/25 v1.5 ifluatex legacy package. Use iftex
instead.
) (c:/TeXLive/2022/texmf-dist/tex/latex/base/textcomp.sty
Package: textcomp 2020/02/02 v2.0n Standard LaTeX package
) (c:/TeXLive/2022/texmf-dist/tex/latex/xkeyval/xkeyval.sty
Package: xkeyval 2022/06/16 v2.9 package option processing (HA)
(c:/TeXLive/2022/texmf-dist/tex/generic/xkeyval/xkeyval.tex
(c:/TeXLive/2022/te
xmf-dist/tex/generic/xkeyval/xkvutils.tex
\XKV@toks=\toks21
\XKV@tempa@toks=\toks22
)
\XKV@depth=\count196
File: xkeyval.tex 2014/12/03 v2.7a key=value parser (HA)
```

```
)) (c:/TeXLive/2022/texmf-dist/tex/latex/base/fontenc.sty
Package: fontenc 2021/04/29 v2.0v Standard LaTeX package
) (c:/TeXLive/2022/texmf-dist/tex/latex/fontaxes/fontaxes.sty
Package: fontaxes 2020/07/21 v1.0e Font selection axes
LaTeX Info: Redefining \upshape on input line 29.
LaTeX Info: Redefining \itshape on input line 31.
LaTeX Info: Redefining \slshape on input line 33.
LaTeX Info: Redefining \swshape on input line 35.
LaTeX Info: Redefining \scshape on input line 37.
LaTeX Info: Redefining \sscshape on input line 39.
LaTeX Info: Redefining \ulcshape on input line 41.
LaTeX Info: Redefining \textsw on input line 47.
LaTeX Info: Redefining \textssc on input line 48.
LaTeX Info: Redefining \textulc on input line 49.
)) (c:/TeXLive/2022/texmf-dist/tex/latex/mathastext/mathastext.sty
Package: mathastext 2022/11/04 v1.3y Use the text font in math mode (JFB)
\mst@exists@muskip=\muskip16
\mst@forall@muskip=\muskip17
\mst@prime@muskip=\muskip18
\mst@do@nonletters=\toks23
\mst@do@easynonletters=\toks24
\mst@do@az=\toks25
\mst@do@AZ=\toks26
\symmtoperatorfont=\mathgroup8
\symmtletterfont=\mathgroup9
** ! and ?
** punctuation: , . : ; and \colon
LaTeX Info: Redefining \relbar on input line 844.
LaTeX Info: Redefining \rightarrowfill on input line 847.
LaTeX Info: Redefining \leftarrowfill on input line 852.
** + and =
LaTeX Info: Redefining \Relbar on input line 943.
** adding = ; and + to \nfss@catcodes
** parentheses ( ) [ ] and slash /
** alldelims: < > \backslash \setminus | \vert \mid \{ and \}
LaTeX Font Info:    Redeclaring math delimiter \backslash on input line
989.
LaTeX Font Info:    Redeclaring math symbol \setminus on input line 1001.
LaTeX Info: Redefining \models on input line 1010.
** \# \mathdollar \% \&
** \imath and \jmath
LaTeX Font Info:    Overwriting math alphabet `\mathnormalbold' in
version `nor
mal'
(Font)                  T1/Merriwthr-OsF/b/it --> T1/Merriwthr-OsF/b/it
on inpu
t line 2370.
LaTeX Font Info:    Overwriting math alphabet `\mathnormalbold' in
version `bol
d'
(Font)                  T1/Merriwthr-OsF/b/it --> T1/Merriwthr-OsF/b/it
on inpu
t line 2370.
```

```
LaTeX Font Info:    Overwriting symbol font `mtletterfont' in version
`normal'
(Font)                  T1/Merriwthr-OsF/m/it --> T1/Merriwthr-OsF/m/it
on inpu
t line 2370.
LaTeX Font Info:    Overwriting symbol font `mtletterfont' in version
`bold'
(Font)                  T1/Merriwthr-OsF/m/it --> T1/Merriwthr-OsF/b/it
on inpu
t line 2370.
LaTeX Font Info:    Overwriting symbol font `mtoperatorfont' in version
`normal
'
(Font)                  T1/Merriwthr-OsF/m/n --> T1/Merriwthr-OsF/m/n on
input
line 2370.
LaTeX Font Info:    Overwriting symbol font `mtoperatorfont' in version
`bold'
(Font)                  T1/Merriwthr-OsF/m/n --> T1/Merriwthr-OsF/b/n on
input
line 2370.
LaTeX Font Info:    Overwriting math alphabet `\Mathbf' in version
`normal'
(Font)                  T1/Merriwthr-OsF/b/n --> T1/Merriwthr-OsF/b/n on
input
line 2370.
LaTeX Font Info:    Overwriting math alphabet `\Mathbf' in version `bold'
(Font)                  T1/Merriwthr-OsF/b/n --> T1/Merriwthr-OsF/b/n on
input
line 2370.
LaTeX Font Info:    Overwriting math alphabet `\Mathit' in version
`normal'
(Font)                  T1/Merriwthr-OsF/m/it --> T1/Merriwthr-OsF/m/it
on inpu
t line 2370.
LaTeX Font Info:    Overwriting math alphabet `\Mathit' in version `bold'
(Font)                  T1/Merriwthr-OsF/m/it --> T1/Merriwthr-OsF/b/it
on inpu
t line 2370.
LaTeX Font Info:    Overwriting math alphabet `\Mathsf' in version
`normal'
(Font)                  T1/MerriwthrSans-OsF/m/n --> T1/MerriwthrSans-
OsF/m/n o
n input line 2370.
LaTeX Font Info:    Overwriting math alphabet `\Mathsf' in version `bold'
(Font)                  T1/MerriwthrSans-OsF/m/n --> T1/MerriwthrSans-
OsF/b/n o
n input line 2370.
LaTeX Font Info:    Overwriting math alphabet `\Mathtt' in version
`normal'
(Font)                  T1/lmtt/m/n --> T1/lmtt/m/n on input line 2370.
LaTeX Font Info:    Overwriting math alphabet `\Mathtt' in version `bold'
(Font)                  T1/lmtt/m/n --> T1/lmtt/b/n on input line 2370.
** Latin letters in the `normal' (resp. `bold') math versions are now
```

```
** set up to use the fonts T1/Merriwthr-OsF/m(b)/it
** Other characters (digits, ...) and \log-like names will be
** typeset with the n shape.
** \hbar
** minus as endash
** \HUGE has been (re)-defined.
** mathastext has declared larger sizes for subscripts.
** To keep LaTeX defaults, use option `defaultmathsizes'.
) (c:/TeXLive/2022/texmf-dist/tex/latex/relsize/relsize.sty
Package: relsize 2013/03/29 ver 4.1
) (c:/TeXLive/2022/texmf-dist/tex/latex/ragged2e/ragged2e.sty
Package: ragged2e 2023/02/25 v3.4 ragged2e Package
\CenteringLeftskip=\skip51
\RaggedLeftLeftskip=\skip52
\RaggedRightLeftskip=\skip53
\CenteringRightskip=\skip54
\RaggedLeftRightskip=\skip55
\RaggedRightRightskip=\skip56
\CenteringParfillskip=\skip57
\RaggedLeftParfillskip=\skip58
\RaggedRightParfillskip=\skip59
\JustifyingParfillskip=\skip60
\CenteringParindent=\skip61
\RaggedLeftParindent=\skip62
\RaggedRightParindent=\skip63
\JustifyingParindent=\skip64
) (c:/TeXLive/2022/texmf-dist/tex/latex/xcolor/xcolor.sty
Package: xcolor 2022/06/12 v2.14 LaTeX color extensions (UK)
(c:/TeXLive/2022/texmf-dist/tex/latex/graphics-cfg/color.cfg
File: color.cfg 2016/01/02 v1.6 sample color configuration
)
Package xcolor Info: Driver file: pdftex.def on input line 227.
(c:/TeXLive/2022/texmf-dist/tex/latex/graphics-def/pdftex.def
File: pdftex.def 2022/09/22 v1.2b Graphics/color driver for pdftex
) (c:/TeXLive/2022/texmf-dist/tex/latex/graphics/mathcolor.ltx)
Package xcolor Info: Model `cmy' substituted by `cmy0' on input line
1353.
Package xcolor Info: Model `hsb' substituted by `rgb' on input line 1357.
Package xcolor Info: Model `RGB' extended on input line 1369.
Package xcolor Info: Model `HTML' substituted by `rgb' on input line
1371.
Package xcolor Info: Model `Hsb' substituted by `hsb' on input line 1372.
Package xcolor Info: Model `tHsb' substituted by `hsb' on input line
1373.
Package xcolor Info: Model `HSB' substituted by `hsb' on input line 1374.
Package xcolor Info: Model `Gray' substituted by `gray' on input line
1375.
Package xcolor Info: Model `wave' substituted by `hsb' on input line
1376.
) (c:/TeXLive/2022/texmf-dist/tex/latex/colortbl/colortbl.sty
Package: colortbl 2022/06/20 v1.0f Color table columns (DPC)
(c:/TeXLive/2022/texmf-dist/tex/latex/tools/array.sty
Package: array 2022/09/04 v2.5g Tabular extension package (FMi)
\col@sep=\dimen142
```

```
\ar@mcellbox=\box52
\extrarowheight=\dimen143
\NC@list=\toks27
\extratabsurround=\skip65
\backup@length=\skip66
\ar@cellbox=\box53
)
\everycr=\toks28
\minrowclearance=\skip67
\rownum=\count197
) (c:/TeXLive/2022/texmf-dist/tex/latex/graphics/graphicx.sty
Package: graphicx 2021/09/16 v1.2d Enhanced LaTeX Graphics (DPC,SPQR)
(c:/TeXLive/2022/texmf-dist/tex/latex/graphics/graphics.sty
Package: graphics 2022/03/10 v1.4e Standard LaTeX Graphics (DPC,SPQR)
(c:/TeXLive/2022/texmf-dist/tex/latex/graphics/trig.sty
Package: trig 2021/08/11 v1.11 sin cos tan (DPC)
) (c:/TeXLive/2022/texmf-dist/tex/latex/graphics-cfg/graphics.cfg
File: graphics.cfg 2016/06/04 v1.11 sample graphics configuration
)
Package graphics Info: Driver file: pdftex.def on input line 107.
)
\Gin@req@height=\dimen144
\Gin@req@width=\dimen145
) (c:/TeXLive/2022/texmf-dist/tex/latex/xpatch/xpatch.sty
(c:/TeXLive/2022/texm
f-dist/tex/latex/l3kernel/expl3.sty
Package: expl3 2023-02-22 L3 programming layer (loader)
(c:/TeXLive/2022/texmf-dist/tex/latex/l3backend/l3backend-pdftex.def
File: l3backend-pdftex.def 2023-01-16 L3 backend support: PDF output
(pdfTeX)
\l__color_backend_stack_int=\count198
\l__pdf_internal_box=\box54
))
Package: xpatch 2020/03/25 v0.3a Extending etoolbox patching commands
(c:/TeXLive/2022/texmf-dist/tex/latex/l3packages/xparse/xparse.sty
Package: xparse 2023-02-02 L3 Experimental document command parser
)) (c:/TeXLive/2022/texmf-dist/tex/latex/environ/environ.sty
Package: environ 2014/05/04 v0.3 A new way to define environments
(c:/TeXLive/2022/texmf-dist/tex/latex/trimspaces/trimspaces.sty
Package: trimspaces 2009/09/17 v1.1 Trim spaces around a token list
)
\@envbody=\toks29
) (c:/TeXLive/2022/texmf-dist/tex/latex/lastpage/lastpage.sty
Package: lastpage 2023/03/07 v2.0a lastpage: 2.09 or 2e? (HMM)
(c:/TeXLive/2022/texmf-dist/tex/latex/lastpage/lastpage2e.sty
Package: lastpage2e 2023/03/07 v2.0a Decide which 2e lastpage version to
use (H
MM)
(c:/TeXLive/2022/texmf-dist/tex/latex/lastpage/lastpagemodern.sty
Package: lastpagemodern 2023-03-07 v2.0a Refers to last page's name (HMM;
JPG)
)
)) (c:/TeXLive/2022/texmf-dist/tex/latex/graphics/rotating.sty
Package: rotating 2016/08/11 v2.16d rotated objects in LaTeX
```

```
(c:/TeXLive/2022/texmf-dist/tex/latex/base/ifthen.sty
Package: ifthen 2022/04/13 v1.1d Standard LaTeX ifthen package (DPC)
)
\c@r@tfl@t=\count199
\rotFPtop=\skip68
\rotFPbot=\skip69
\rot@float@box=\box55
\rot@mess@toks=\toks30
) (c:/TeXLive/2022/texmf-dist/tex/latex/graphics/lscape.sty
Package: lscape 2020/05/28 v3.02 Landscape Pages (DPC)
) (c:/TeXLive/2022/texmf-dist/tex/latex/tools/afterpage.sty
Package: afterpage 2014/10/28 v1.08 After-Page Package (DPC)
\AP@output=\toks31
\AP@partial=\box56
\AP@footins=\box57
) (c:/TeXLive/2022/texmf-dist/tex/latex/textpos/textpos.sty
Package: textpos 2022/07/23 v1.10.1
Package textpos Info: choosing support for LaTeX3 on input line 60.
\TP@textbox=\box58
\TP@holdbox=\box59
\TPHorizModule=\dimen146
\TPVertModule=\dimen147
\TP@margin=\dimen148
\TP@absmargin=\dimen149
Grid set 16 x 16 = 37.34424pt x 52.81541pt
\TPboxrulesize=\dimen150
\TP@ox=\dimen151
\TP@oy=\dimen152
\TP@tbargs=\toks32
TextBlockOrigin set to 0pt x 0pt
) (c:/TeXLive/2022/texmf-dist/tex/latex/url/url.sty
\Urlmuskip=\muskip19
Package: url 2013/09/16  ver 3.4  Verb mode for urls, etc.
) (c:/TeXLive/2022/texmf-dist/tex/latex/newfloat/newfloat.sty
Package: newfloat 2019/09/02 v1.1l Defining new floating environments
(AR)
Package newfloat Info: `rotating' package detected.
) (c:/TeXLive/2022/texmf-dist/tex/latex/mdframed/mdframed.sty
Package: mdframed 2013/07/01 1.9b: mdframed
(c:/TeXLive/2022/texmf-dist/tex/latex/kvoptions/kvoptions.sty
Package: kvoptions 2022-06-15 v3.15 Key value format for package options
(HO)
(c:/TeXLive/2022/texmf-dist/tex/generic/ltxcmds/ltxcmds.sty
Package: ltxcmds 2020-05-10 v1.25 LaTeX kernel commands for general use
(HO)
) (c:/TeXLive/2022/texmf-dist/tex/latex/kvsetkeys/kvsetkeys.sty
Package: kvsetkeys 2022-10-05 v1.19 Key value parser (HO)
)) (c:/TeXLive/2022/texmf-dist/tex/latex/zref/zref-abspage.sty
Package: zref-abspage 2022-04-07 v2.34 Module abspage for zref (HO)
(c:/TeXLive/2022/texmf-dist/tex/latex/zref/zref-base.sty
Package: zref-base 2022-04-07 v2.34 Module base for zref (HO)
(c:/TeXLive/2022/texmf-dist/tex/generic/infwarerr/infwarerr.sty
Package: infwarerr 2019/12/03 v1.5 Providing info/warning/error messages
(HO)
```

```
) (c:/TeXLive/2022/texmf-dist/tex/generic/kvdefinekeys/kvdefinekeys.sty
Package: kvdefinekeys 2019-12-19 v1.6 Define keys (HO)
) (c:/TeXLive/2022/texmf-dist/tex/generic/pdftexcmds/pdftexcmds.sty
Package: pdftexcmds 2020-06-27 v0.33 Utility functions of pdfTeX for
LuaTeX (HO
)
Package pdftexcmds Info: \pdf@primitive is available.
Package pdftexcmds Info: \pdf@ifprimitive is available.
Package pdftexcmds Info: \pdfdraftmode found.
) (c:/TeXLive/2022/texmf-dist/tex/generic/etexcmds/etexcmds.sty
Package: etexcmds 2019/12/15 v1.7 Avoid name clashes with e-TeX commands
(HO)
) (c:/TeXLive/2022/texmf-dist/tex/latex/auxhook/auxhook.sty
Package: auxhook 2019-12-17 v1.6 Hooks for auxiliary files (HO)
)
Package zref Info: New property list: main on input line 767.
Package zref Info: New property: default on input line 768.
Package zref Info: New property: page on input line 769.
) (c:/TeXLive/2022/texmf-dist/tex/latex/base/atbegshi-ltx.sty
Package: atbegshi-ltx 2021/01/10 v1.0c Emulation of the original atbegshi
package with kernel methods
)
\c@abspage=\count266
Package zref Info: New property: abspage on input line 65.
) (c:/TeXLive/2022/texmf-dist/tex/latex/needspace/needspace.sty
Package: needspace 2010/09/12 v1.3d reserve vertical space
)
\mdf@templength=\skip70
\c@mdf@globalstyle@cnt=\count267
\mdf@skipabove@length=\skip71
\mdf@skipbelow@length=\skip72
\mdf@leftmargin@length=\skip73
\mdf@rightmargin@length=\skip74
\mdf@innerleftmargin@length=\skip75
\mdf@innerrightmargin@length=\skip76
\mdf@innertopmargin@length=\skip77
\mdf@innerbottommargin@length=\skip78
\mdf@splittopskip@length=\skip79
\mdf@splitbottomskip@length=\skip80
\mdf@outermargin@length=\skip81
\mdf@innermargin@length=\skip82
\mdf@linewidth@length=\skip83
\mdf@innerlinewidth@length=\skip84
\mdf@middlelinewidth@length=\skip85
\mdf@outerlinewidth@length=\skip86
\mdf@roundcorner@length=\skip87
\mdf@footenotedistance@length=\skip88
\mdf@userdefinedwidth@length=\skip89
\mdf@needspace@length=\skip90
\mdf@frametitleaboveskip@length=\skip91
\mdf@frametitlebelowskip@length=\skip92
\mdf@frametitlerulewidth@length=\skip93
\mdf@frametitleleftmargin@length=\skip94
\mdf@frametitlerightmargin@length=\skip95
```

```
\mdf@shadowsize@length=\skip96
\mdf@extratopheight@length=\skip97
\mdf@subtitleabovelinewidth@length=\skip98
\mdf@subtitlebelowlinewidth@length=\skip99
\mdf@subtitleaboveskip@length=\skip100
\mdf@subtitlebelowskip@length=\skip101
\mdf@subtitleinneraboveskip@length=\skip102
\mdf@subtitleinnerbelowskip@length=\skip103
\mdf@subsubtitleabovelinewidth@length=\skip104
\mdf@subsubtitlebelowlinewidth@length=\skip105
\mdf@subsubtitleaboveskip@length=\skip106
\mdf@subsubtitlebelowskip@length=\skip107
\mdf@subsubtitleinneraboveskip@length=\skip108
\mdf@subsubtitleinnerbelowskip@length=\skip109
(c:/TeXLive/2022/texmf-dist/tex/latex/mdframed/md-frame-0.mdf
File: md-frame-0.mdf 2013/07/01\ 1.9b: md-frame-0
)
\mdf@frametitlebox=\box60
\mdf@footnotebox=\box61
\mdf@splitbox@one=\box62
\mdf@splitbox@two=\box63
\mdf@splitbox@save=\box64
\mdfsplitboxwidth=\skip110
\mdfsplitboxtotalwidth=\skip111
\mdfsplitboxheight=\skip112
\mdfsplitboxdepth=\skip113
\mdfsplitboxtotalheight=\skip114
\mdfframetitleboxwidth=\skip115
\mdfframetitleboxtotalwidth=\skip116
\mdfframetitleboxheight=\skip117
\mdfframetitleboxdepth=\skip118
\mdfframetitleboxtotalheight=\skip119
\mdffootnoteboxwidth=\skip120
\mdffootnoteboxtotalwidth=\skip121
\mdffootnoteboxheight=\skip122
\mdffootnoteboxdepth=\skip123
\mdffootnoteboxtotalheight=\skip124
\mdftotallinewidth=\skip125
\mdfboundingboxwidth=\skip126
\mdfboundingboxtotalwidth=\skip127
\mdfboundingboxheight=\skip128
\mdfboundingboxdepth=\skip129
\mdfboundingboxtotalheight=\skip130
\mdf@freevspace@length=\skip131
\mdf@horizontalwidthofbox@length=\skip132
\mdf@verticalmarginwhole@length=\skip133
\mdf@horizontalspaceofbox=\skip134
\mdfsubtitleheight=\skip135
\mdfsubsubtitleheight=\skip136
\c@mdfcountframes=\count268

****** mdframed patching \endmdf@trivlist

****** -- success******
```

```
\mdf@envdepth=\count269
\c@mdf@env@i=\count270
\c@mdf@env@ii=\count271
\c@mdf@zref@counter=\count272
Package zref Info: New property: mdf@pagevalue on input line 895.
) (c:/TeXLive/2022/texmf-dist/tex/latex/titlesec/titlesec.sty
Package: titlesec 2021/07/05 v2.14 Sectioning titles
\ttl@box=\box65
\beforetitleunit=\skip137
\aftertitleunit=\skip138
\ttl@plus=\dimen153
\ttl@minus=\dimen154
\ttl@toksa=\toks33
\titlewidth=\dimen155
\titlewidthlast=\dimen156
\titlewidthfirst=\dimen157
) (c:/TeXLive/2022/texmf-dist/tex/latex/koma-script/scrextend.sty
Package: scrextend 2022/10/12 v3.38 KOMA-Script package (extend other
classes w
ith features of KOMA-Script classes)
(c:/TeXLive/2022/texmf-dist/tex/latex/koma-script/scrkbase.sty
Package: scrkbase 2022/10/12 v3.38 KOMA-Script package (KOMA-Script-
dependent b
asics and keyval usage)
(c:/TeXLive/2022/texmf-dist/tex/latex/koma-script/scrbase.sty
Package: scrbase 2022/10/12 v3.38 KOMA-Script package (KOMA-Script-
independent
basics and keyval usage)
(c:/TeXLive/2022/texmf-dist/tex/latex/koma-script/scrlfile.sty
Package: scrlfile 2022/10/12 v3.38 KOMA-Script package (file load hooks)
(c:/TeXLive/2022/texmf-dist/tex/latex/koma-script/scrlfile-hook.sty
Package: scrlfile-hook 2022/10/12 v3.38 KOMA-Script package (using LaTeX
hooks)

(c:/TeXLive/2022/texmf-dist/tex/latex/koma-script/scrlogo.sty
Package: scrlogo 2022/10/12 v3.38 KOMA-Script package (logo)
)))
Applying: [2021/05/01] Usage of raw or classic option list on input line
252.
Already applied: [0000/00/00] Usage of raw or classic option list on
input line
 368.
))
Package scrextend Info: unexpected definition of `\@makefnmark'.
(scrextend)             Trying to patch it on input line 1709.
Package scrextend Info: patch seems to be successfull on input line 1709.
)

LaTeX Font Warning: Font shape `T1/cmr/m/n' in size <7.5> not available
(Font)              size <7> substituted on input line 65.

(c:/TeXLive/2022/texmf-dist/tex/latex/tools/calc.sty
Package: calc 2017/05/25 v4.3 Infix arithmetic (KKT,FJ)
```

```
\calc@Acount=\count273
\calc@Bcount=\count274
\calc@Adimen=\dimen158
\calc@Bdimen=\dimen159
\calc@Askip=\skip139
\calc@Bskip=\skip140
LaTeX Info: Redefining \setlength on input line 80.
LaTeX Info: Redefining \addtolength on input line 81.
\calc@Ccount=\count275
\calc@Cskip=\skip141
) (c:/TeXLive/2022/texmf-dist/tex/latex/geometry/geometry.sty
Package: geometry 2020/01/02 v5.9 Page Geometry
(c:/TeXLive/2022/texmf-dist/tex/generic/iftex/ifvtex.sty
Package: ifvtex 2019/10/25 v1.7 ifvtex legacy package. Use iftex instead.
)
\Gm@cnth=\count276
\Gm@cntv=\count277
\c@Gm@tempcnt=\count278
\Gm@bindingoffset=\dimen160
\Gm@wd@mp=\dimen161
\Gm@odd@mp=\dimen162
\Gm@even@mp=\dimen163
\Gm@layoutwidth=\dimen164
\Gm@layoutheight=\dimen165
\Gm@layouthoffset=\dimen166
\Gm@layoutvoffset=\dimen167
\Gm@dimlist=\toks34
) (c:/TeXLive/2022/texmf-dist/tex/latex/hyperref/hyperref.sty
Package: hyperref 2023-02-07 v7.00v Hypertext links for LaTeX
(c:/TeXLive/2022/texmf-dist/tex/generic/pdfescape/pdfescape.sty
Package: pdfescape 2019/12/09 v1.15 Implements pdfTeX's escape features
(HO)
) (c:/TeXLive/2022/texmf-dist/tex/latex/hycolor/hycolor.sty
Package: hycolor 2020-01-27 v1.10 Color options for hyperref/bookmark
(HO)
) (c:/TeXLive/2022/texmf-dist/tex/latex/letltxmacro/letltxmacro.sty
Package: letltxmacro 2019/12/03 v1.6 Let assignment for LaTeX macros (HO)
) (c:/TeXLive/2022/texmf-dist/tex/latex/hyperref/nameref.sty
Package: nameref 2022-05-17 v2.50 Cross-referencing by name of section
(c:/TeXLive/2022/texmf-dist/tex/latex/refcount/refcount.sty
Package: refcount 2019/12/15 v3.6 Data extraction from label references
(HO)
) (c:/TeXLive/2022/texmf-
dist/tex/generic/gettitlestring/gettitlestring.sty
Package: gettitlestring 2019/12/15 v1.6 Cleanup title references (HO)
)
\c@section@level=\count279
)
\@linkdim=\dimen168
\Hy@linkcounter=\count280
\Hy@pagecounter=\count281
(c:/TeXLive/2022/texmf-dist/tex/latex/hyperref/pd1enc.def
File: pd1enc.def 2023-02-07 v7.00v Hyperref: PDFDocEncoding definition
(HO)
```

```
Now handling font encoding PD1 ...
... no UTF-8 mapping file for font encoding PD1
) (c:/TeXLive/2022/texmf-dist/tex/generic/intcalc/intcalc.sty
Package: intcalc 2019/12/15 v1.3 Expandable calculations with integers
(HO)
)
\Hy@SavedSpaceFactor=\count282
(c:/TeXLive/2022/texmf-dist/tex/latex/hyperref/puenc.def
File: puenc.def 2023-02-07 v7.00v Hyperref: PDF Unicode definition (HO)
Now handling font encoding PU ...
... no UTF-8 mapping file for font encoding PU
)
Package hyperref Info: Option `colorlinks' set `true' on input line 4060.
Package hyperref Info: Hyper figures OFF on input line 4177.
Package hyperref Info: Link nesting OFF on input line 4182.
Package hyperref Info: Hyper index ON on input line 4185.
Package hyperref Info: Plain pages OFF on input line 4192.
Package hyperref Info: Backreferencing OFF on input line 4197.
Package hyperref Info: Implicit mode ON; LaTeX internals redefined.
Package hyperref Info: Bookmarks ON on input line 4425.
\c@Hy@tempcnt=\count283
LaTeX Info: Redefining \url on input line 4763.
\XeTeXLinkMargin=\dimen169
(c:/TeXLive/2022/texmf-dist/tex/generic/bitset/bitset.sty
Package: bitset 2019/12/09 v1.3 Handle bit-vector datatype (HO)
(c:/TeXLive/2022/texmf-dist/tex/generic/bigintcalc/bigintcalc.sty
Package: bigintcalc 2019/12/15 v1.5 Expandable calculations on big
integers (HO
)
))
\Fld@menulength=\count284
\Field@Width=\dimen170
\Fld@charsize=\dimen171
Package hyperref Info: Hyper figures OFF on input line 6042.
Package hyperref Info: Link nesting OFF on input line 6047.
Package hyperref Info: Hyper index ON on input line 6050.
Package hyperref Info: backreferencing OFF on input line 6057.
Package hyperref Info: Link coloring ON on input line 6060.
Package hyperref Info: Link coloring with OCG OFF on input line 6067.
Package hyperref Info: PDF/A mode OFF on input line 6072.
\Hy@abspage=\count285
\c@Item=\count286
\c@Hfootnote=\count287
)
Package hyperref Info: Driver (autodetected): hpdftex.
(c:/TeXLive/2022/texmf-dist/tex/latex/hyperref/hpdftex.def
File: hpdftex.def 2023-02-07 v7.00v Hyperref driver for pdfTeX
(c:/TeXLive/2022/texmf-dist/tex/latex/base/atveryend-ltx.sty
Package: atveryend-ltx 2020/08/19 v1.0a Emulation of the original
atveryend pac
kage
with kernel methods
)
\HyAnn@Count=\count288
```

```
\Fld@listcount=\count289
\c@bookmark@seq@number=\count290
(c:/TeXLive/2022/texmf-dist/tex/latex/rerunfilecheck/rerunfilecheck.sty
Package: rerunfilecheck 2022-07-10 v1.10 Rerun checks for auxiliary files
(HO)
(c:/TeXLive/2022/texmf-dist/tex/generic/uniquecounter/uniquecounter.sty
Package: uniquecounter 2019/12/15 v1.4 Provide unlimited unique counter
(HO)
)
Package uniquecounter Info: New unique counter `rerunfilecheck' on input
line 2
85.
)
\Hy@SectionHShift=\skip142
) (c:/TeXLive/2022/texmf-dist/tex/latex/preprint/authblk.sty
Package: authblk 2001/02/27 1.3 (PWD)
\affilsep=\skip143
\@affilsep=\skip144
\c@Maxaffil=\count291
\c@authors=\count292
\c@affil=\count293
) (c:/TeXLive/2022/texmf-dist/tex/latex/footmisc/footmisc.sty
Package: footmisc 2022/03/08 v6.0d a miscellany of footnote facilities
\FN@temptoken=\toks35
\footnotemargin=\dimen172
\@outputbox@depth=\dimen173
Package footmisc Info: Declaring symbol style bringhurst on input line
695.
Package footmisc Info: Declaring symbol style chicago on input line 703.
Package footmisc Info: Declaring symbol style wiley on input line 712.
Package footmisc Info: Declaring symbol style lamport-robust on input
line 723.

Package footmisc Info: Declaring symbol style lamport* on input line 743.
Package footmisc Info: Declaring symbol style lamport*-robust on input
line 764
.
) (c:/TeXLive/2022/texmf-dist/tex/latex/fancyhdr/fancyhdr.sty
Package: fancyhdr 2022/11/09 v4.1 Extensive control of page headers and
footers

\f@nch@headwidth=\skip145
\f@nch@O@elh=\skip146
\f@nch@O@erh=\skip147
\f@nch@O@olh=\skip148
\f@nch@O@orh=\skip149
\f@nch@O@elf=\skip150
\f@nch@O@erf=\skip151
\f@nch@O@olf=\skip152
\f@nch@O@orf=\skip153
) (c:/TeXLive/2022/texmf-dist/tex/generic/alphalph/alphalph.sty
Package: alphalph 2019/12/09 v2.6 Convert numbers to letters (HO)
)
\c@authorfn=\count294
```

```
(c:/TeXLive/2022/texmf-dist/tex/latex/abstract/abstract.sty
Package: abstract 2009/06/08 v1.2a configurable abstracts
\abstitleskip=\skip154
\absleftindent=\skip155
\absrightindent=\skip156
\absparindent=\skip157
\absparsep=\skip158
)
Package newfloat Info: New float `keypoints' with options
`placement=t!,name=kp
t' on input line 286.
\c@keypoints=\count295
\newfloat@ftype=\count296
Package newfloat Info: float type `keypoints'=8 on input line 286.
(c:/TeXLive/2022/texmf-dist/tex/latex/enumitem/enumitem.sty
Package: enumitem 2019/06/20 v3.9 Customized lists
\labelindent=\skip159
\enit@outerparindent=\dimen174
\enit@toks=\toks36
\enit@inbox=\box66
\enit@count@id=\count297
\enitdp@description=\count298
) (c:/TeXLive/2022/texmf-dist/tex/latex/quoting/quoting.sty
Package: quoting 2014/01/28 v0.1c Consolidated environment for displayed
text
\quo@toppartop=\skip160
) (c:/TeXLive/2022/texmf-dist/tex/latex/sttools/stfloats.sty
Package: stfloats 2017/03/27 v3.3 Improve float mechanism and
baselineskip sett
ings
\@dblbotnum=\count299
\c@dblbotnumber=\count300
) (c:/TeXLive/2022/texmf-dist/tex/latex/booktabs/booktabs.sty
Package: booktabs 2020/01/12 v1.61803398 Publication quality tables
\heavyrulewidth=\dimen175
\lightrulewidth=\dimen176
\cmidrulewidth=\dimen177
\belowrulesep=\dimen178
\belowbottomsep=\dimen179
\aboverulesep=\dimen180
\abovetopsep=\dimen181
\cmidrulesep=\dimen182
\cmidrulekern=\dimen183
\defaultaddspace=\dimen184
\@cmidla=\count301
\@cmidlb=\count302
\@aboverulesep=\dimen185
\@belowrulesep=\dimen186
\@thisruleclass=\count303
\@lastruleclass=\count304
\@thisrulewidth=\dimen187
) (c:/TeXLive/2022/texmf-dist/tex/latex/tools/tabularx.sty
Package: tabularx 2020/01/15 v2.11c `tabularx' package (DPC)
\TX@col@width=\dimen188
```

```
\TX@old@table=\dimen189
\TX@old@col=\dimen190
\TX@target=\dimen191
\TX@delta=\dimen192
\TX@cols=\count305
\TX@ftn=\toks37
)
\enitdp@tablenotes=\count306
(c:/TeXLive/2022/texmf-dist/tex/latex/caption/caption.sty
Package: caption 2022/03/01 v3.6b Customizing captions (AR)
(c:/TeXLive/2022/texmf-dist/tex/latex/caption/caption3.sty
Package: caption3 2022/03/17 v2.3b caption3 kernel (AR)
\caption@tempdima=\dimen193
\captionmargin=\dimen194
\caption@leftmargin=\dimen195
\caption@rightmargin=\dimen196
\caption@width=\dimen197
\caption@indent=\dimen198
\caption@parindent=\dimen199
\caption@hangindent=\dimen256
Package caption Info: Standard document class detected.
)
\c@caption@flags=\count307
\c@continuedfloat=\count308
Package caption Info: hyperref package is loaded.
Package caption Info: rotating package is loaded.
) (c:/TeXLive/2022/texmf-dist/tex/latex/natbib/natbib.sty
Package: natbib 2010/09/13 8.31b (PWD, AO)
\bibhang=\skip161
\bibsep=\skip162
LaTeX Info: Redefining \cite on input line 694.
\c@NAT@ctr=\count309
)) (c:/TeXLive/2022/texmf-dist/tex/latex/siunitx/siunitx.sty
Package: siunitx 2023-03-04 v3.2.2 A comprehensive (SI) units package
\l__siunitx_angle_tmp_dim=\dimen257
\l__siunitx_angle_marker_box=\box67
\l__siunitx_angle_unit_box=\box68
\l__siunitx_compound_count_int=\count310
(c:/TeXLive/2022/texmf-dist/tex/latex/translations/translations.sty
Package: translations 2022/02/05 v1.12 internationalization of LaTeX2e
packages
 (CN)
)
\l__siunitx_number_exponent_fixed_int=\count311
\l__siunitx_number_min_decimal_int=\count312
\l__siunitx_number_min_integer_int=\count313
\l__siunitx_number_round_precision_int=\count314
\l__siunitx_number_lower_threshold_int=\count315
\l__siunitx_number_upper_threshold_int=\count316
\l__siunitx_number_group_first_int=\count317
\l__siunitx_number_group_size_int=\count318
\l__siunitx_number_group_minimum_int=\count319
(c:/TeXLive/2022/texmf-dist/tex/latex/amsmath/amstext.sty
Package: amstext 2021/08/26 v2.01 AMS text
```

```
(c:/TeXLive/2022/texmf-dist/tex/latex/amsmath/amsgen.sty
File: amsgen.sty 1999/11/30 v2.0 generic functions
\@emptytoks=\toks38
\ex@=\dimen258
))
\l__siunitx_table_tmp_box=\box69
\l__siunitx_table_tmp_dim=\dimen259
\l__siunitx_table_column_width_dim=\dimen260
\l__siunitx_table_integer_box=\box70
\l__siunitx_table_decimal_box=\box71
\l__siunitx_table_uncert_box=\box72
\l__siunitx_table_before_box=\box73
\l__siunitx_table_after_box=\box74
\l__siunitx_table_before_dim=\dimen261
\l__siunitx_table_carry_dim=\dimen262
\l__siunitx_unit_tmp_int=\count320
\l__siunitx_unit_position_int=\count321
\l__siunitx_unit_total_int=\count322
) (c:/TeXLive/2022/texmf-dist/tex/latex/tools/bm.sty
Package: bm 2022/01/05 v1.2f Bold Symbol Support (DPC/FMi)
\symboldoperators=\mathgroup10
\symboldletters=\mathgroup11
\symboldsymbols=\mathgroup12
Package bm Info: No bold for \OMX/cmex/m/n, using \pmb.
\symboldEulerFraktur=\mathgroup13
Package bm Info: No bold for \U/euex/m/n, using \pmb.
LaTeX Font Info:    Redeclaring math alphabet \mathbf on input line 149.
) (c:/TeXLive/2022/texmf-dist/tex/latex/pgf/frontendlayer/tikz.sty
(c:/TeXLive/
2022/texmf-dist/tex/latex/pgf/basiclayer/pgf.sty (c:/TeXLive/2022/texmf-
dist/te
x/latex/pgf/utilities/pgfrcs.sty (c:/TeXLive/2022/texmf-
dist/tex/generic/pgf/ut
ilities/pgfutil-common.tex
\pgfutil@everybye=\toks39
\pgfutil@tempdima=\dimen263
\pgfutil@tempdimb=\dimen264
) (c:/TeXLive/2022/texmf-dist/tex/generic/pgf/utilities/pgfutil-latex.def
\pgfutil@abb=\box75
) (c:/TeXLive/2022/texmf-dist/tex/generic/pgf/utilities/pgfrcs.code.tex
(c:/TeX
Live/2022/texmf-dist/tex/generic/pgf/pgf.revision.tex)
Package: pgfrcs 2023-01-15 v3.1.10 (3.1.10)
))
Package: pgf 2023-01-15 v3.1.10 (3.1.10)
(c:/TeXLive/2022/texmf-dist/tex/latex/pgf/basiclayer/pgfcore.sty
(c:/TeXLive/20
22/texmf-dist/tex/latex/pgf/systemlayer/pgfsys.sty
(c:/TeXLive/2022/texmf-dist/
tex/generic/pgf/systemlayer/pgfsys.code.tex
Package: pgfsys 2023-01-15 v3.1.10 (3.1.10)
(c:/TeXLive/2022/texmf-dist/tex/generic/pgf/utilities/pgfkeys.code.tex
\pgfkeys@pathtoks=\toks40
\pgfkeys@temptoks=\toks41
```

```
(c:/TeXLive/2022/texmf-
dist/tex/generic/pgf/utilities/pgfkeyslibraryfiltered.co
de.tex
\pgfkeys@tmptoks=\toks42
))
\pgf@x=\dimen265
\pgf@y=\dimen266
\pgf@xa=\dimen267
\pgf@ya=\dimen268
\pgf@xb=\dimen269
\pgf@yb=\dimen270
\pgf@xc=\dimen271
\pgf@yc=\dimen272
\pgf@xd=\dimen273
\pgf@yd=\dimen274
\w@pgf@writea=\write3
\r@pgf@reada=\read2
\c@pgf@counta=\count323
\c@pgf@countb=\count324
\c@pgf@countc=\count325
\c@pgf@countd=\count326
\t@pgf@toka=\toks43
\t@pgf@tokb=\toks44
\t@pgf@tokc=\toks45
\pgf@sys@id@count=\count327
(c:/TeXLive/2022/texmf-dist/tex/generic/pgf/systemlayer/pgf.cfg
File: pgf.cfg 2023-01-15 v3.1.10 (3.1.10)
)
Driver file for pgf: pgfsys-pdftex.def
(c:/TeXLive/2022/texmf-dist/tex/generic/pgf/systemlayer/pgfsys-pdftex.def
File: pgfsys-pdftex.def 2023-01-15 v3.1.10 (3.1.10)
(c:/TeXLive/2022/texmf-dist/tex/generic/pgf/systemlayer/pgfsys-common-
pdf.def
File: pgfsys-common-pdf.def 2023-01-15 v3.1.10 (3.1.10)
)))
(c:/TeXLive/2022/texmf-
dist/tex/generic/pgf/systemlayer/pgfsyssoftpath.code.tex
File: pgfsyssoftpath.code.tex 2023-01-15 v3.1.10 (3.1.10)
\pgfsyssoftpath@smallbuffer@items=\count328
\pgfsyssoftpath@bigbuffer@items=\count329
)
(c:/TeXLive/2022/texmf-
dist/tex/generic/pgf/systemlayer/pgfsysprotocol.code.tex
File: pgfsysprotocol.code.tex 2023-01-15 v3.1.10 (3.1.10)
)) (c:/TeXLive/2022/texmf-
dist/tex/generic/pgf/basiclayer/pgfcore.code.tex
Package: pgfcore 2023-01-15 v3.1.10 (3.1.10)
(c:/TeXLive/2022/texmf-dist/tex/generic/pgf/math/pgfmath.code.tex
(c:/TeXLive/2
022/texmf-dist/tex/generic/pgf/math/pgfmathutil.code.tex)
(c:/TeXLive/2022/texm
f-dist/tex/generic/pgf/math/pgfmathparser.code.tex
\pgfmath@dimen=\dimen275
```

```
\pgfmath@count=\count330
\pgfmath@box=\box76
\pgfmath@toks=\toks46
\pgfmath@stack@operand=\toks47
\pgfmath@stack@operation=\toks48
) (c:/TeXLive/2022/texmf-
dist/tex/generic/pgf/math/pgfmathfunctions.code.tex)
(c:/TeXLive/2022/texmf-
dist/tex/generic/pgf/math/pgfmathfunctions.basic.code.te
x)
(c:/TeXLive/2022/texmf-
dist/tex/generic/pgf/math/pgfmathfunctions.trigonometric
.code.tex)
(c:/TeXLive/2022/texmf-
dist/tex/generic/pgf/math/pgfmathfunctions.random.code.t
ex)
(c:/TeXLive/2022/texmf-
dist/tex/generic/pgf/math/pgfmathfunctions.comparison.co
de.tex)
(c:/TeXLive/2022/texmf-
dist/tex/generic/pgf/math/pgfmathfunctions.base.code.tex
)
(c:/TeXLive/2022/texmf-
dist/tex/generic/pgf/math/pgfmathfunctions.round.code.te
x)
(c:/TeXLive/2022/texmf-
dist/tex/generic/pgf/math/pgfmathfunctions.misc.code.tex
)
(c:/TeXLive/2022/texmf-
dist/tex/generic/pgf/math/pgfmathfunctions.integerarithm
etics.code.tex) (c:/TeXLive/2022/texmf-
dist/tex/generic/pgf/math/pgfmathcalc.co
de.tex) (c:/TeXLive/2022/texmf-
dist/tex/generic/pgf/math/pgfmathfloat.code.tex
\c@pgfmathroundto@lastzeros=\count331
)) (c:/TeXLive/2022/texmf-dist/tex/generic/pgf/math/pgfint.code.tex)
(c:/TeXLiv
e/2022/texmf-dist/tex/generic/pgf/basiclayer/pgfcorepoints.code.tex
File: pgfcorepoints.code.tex 2023-01-15 v3.1.10 (3.1.10)
\pgf@picminx=\dimen276
\pgf@picmaxx=\dimen277
\pgf@picminy=\dimen278
\pgf@picmaxy=\dimen279
\pgf@pathminx=\dimen280
\pgf@pathmaxx=\dimen281
\pgf@pathminy=\dimen282
\pgf@pathmaxy=\dimen283
\pgf@xx=\dimen284
\pgf@xy=\dimen285
\pgf@yx=\dimen286
\pgf@yy=\dimen287
\pgf@zx=\dimen288
\pgf@zy=\dimen289
)
```

```
(c:/TeXLive/2022/texmf-
dist/tex/generic/pgf/basiclayer/pgfcorepathconstruct.cod
e.tex
File: pgfcorepathconstruct.code.tex 2023-01-15 v3.1.10 (3.1.10)
\pgf@path@lastx=\dimen290
\pgf@path@lasty=\dimen291
)
(c:/TeXLive/2022/texmf-
dist/tex/generic/pgf/basiclayer/pgfcorepathusage.code.te
x
File: pgfcorepathusage.code.tex 2023-01-15 v3.1.10 (3.1.10)
\pgf@shorten@end@additional=\dimen292
\pgf@shorten@start@additional=\dimen293
) (c:/TeXLive/2022/texmf-
dist/tex/generic/pgf/basiclayer/pgfcorescopes.code.tex
File: pgfcorescopes.code.tex 2023-01-15 v3.1.10 (3.1.10)
\pgfpic=\box77
\pgf@hbox=\box78
\pgf@layerbox@main=\box79
\pgf@picture@serial@count=\count332
)
(c:/TeXLive/2022/texmf-
dist/tex/generic/pgf/basiclayer/pgfcoregraphicstate.code
.tex
File: pgfcoregraphicstate.code.tex 2023-01-15 v3.1.10 (3.1.10)
\pgflinewidth=\dimen294
)
(c:/TeXLive/2022/texmf-
dist/tex/generic/pgf/basiclayer/pgfcoretransformations.c
ode.tex
File: pgfcoretransformations.code.tex 2023-01-15 v3.1.10 (3.1.10)
\pgf@pt@x=\dimen295
\pgf@pt@y=\dimen296
\pgf@pt@temp=\dimen297
) (c:/TeXLive/2022/texmf-
dist/tex/generic/pgf/basiclayer/pgfcorequick.code.tex
File: pgfcorequick.code.tex 2023-01-15 v3.1.10 (3.1.10)
) (c:/TeXLive/2022/texmf-
dist/tex/generic/pgf/basiclayer/pgfcoreobjects.code.te
x
File: pgfcoreobjects.code.tex 2023-01-15 v3.1.10 (3.1.10)
)
(c:/TeXLive/2022/texmf-
dist/tex/generic/pgf/basiclayer/pgfcorepathprocessing.co
de.tex
File: pgfcorepathprocessing.code.tex 2023-01-15 v3.1.10 (3.1.10)
) (c:/TeXLive/2022/texmf-
dist/tex/generic/pgf/basiclayer/pgfcorearrows.code.tex
File: pgfcorearrows.code.tex 2023-01-15 v3.1.10 (3.1.10)
\pgfarrowsep=\dimen298
) (c:/TeXLive/2022/texmf-
dist/tex/generic/pgf/basiclayer/pgfcoreshade.code.tex
File: pgfcoreshade.code.tex 2023-01-15 v3.1.10 (3.1.10)
\pgf@max=\dimen299
```

```
\pgf@sys@shading@range@num=\count333
\pgf@shadingcount=\count334
) (c:/TeXLive/2022/texmf-
dist/tex/generic/pgf/basiclayer/pgfcoreimage.code.tex
File: pgfcoreimage.code.tex 2023-01-15 v3.1.10 (3.1.10)
)
(c:/TeXLive/2022/texmf-
dist/tex/generic/pgf/basiclayer/pgfcoreexternal.code.tex
File: pgfcoreexternal.code.tex 2023-01-15 v3.1.10 (3.1.10)
\pgfexternal@startupbox=\box80
) (c:/TeXLive/2022/texmf-
dist/tex/generic/pgf/basiclayer/pgfcorelayers.code.tex
File: pgfcorelayers.code.tex 2023-01-15 v3.1.10 (3.1.10)
)
(c:/TeXLive/2022/texmf-
dist/tex/generic/pgf/basiclayer/pgfcoretransparency.code
.tex
File: pgfcoretransparency.code.tex 2023-01-15 v3.1.10 (3.1.10)
)
(c:/TeXLive/2022/texmf-
dist/tex/generic/pgf/basiclayer/pgfcorepatterns.code.tex
File: pgfcorepatterns.code.tex 2023-01-15 v3.1.10 (3.1.10)
) (c:/TeXLive/2022/texmf-
dist/tex/generic/pgf/basiclayer/pgfcorerdf.code.tex
File: pgfcorerdf.code.tex 2023-01-15 v3.1.10 (3.1.10)
))) (c:/TeXLive/2022/texmf-
dist/tex/generic/pgf/modules/pgfmoduleshapes.code.te
x
File: pgfmoduleshapes.code.tex 2023-01-15 v3.1.10 (3.1.10)
\pgfnodeparttextbox=\box81
) (c:/TeXLive/2022/texmf-
dist/tex/generic/pgf/modules/pgfmoduleplot.code.tex
File: pgfmoduleplot.code.tex 2023-01-15 v3.1.10 (3.1.10)
)
(c:/TeXLive/2022/texmf-dist/tex/latex/pgf/compatibility/pgfcomp-version-
0-65.st
y
Package: pgfcomp-version-0-65 2023-01-15 v3.1.10 (3.1.10)
\pgf@nodesepstart=\dimen300
\pgf@nodesepend=\dimen301
)
(c:/TeXLive/2022/texmf-dist/tex/latex/pgf/compatibility/pgfcomp-version-
1-18.st
y
Package: pgfcomp-version-1-18 2023-01-15 v3.1.10 (3.1.10)
)) (c:/TeXLive/2022/texmf-dist/tex/latex/pgf/utilities/pgffor.sty
(c:/TeXLive/2
022/texmf-dist/tex/latex/pgf/utilities/pgfkeys.sty
(c:/TeXLive/2022/texmf-dist/
tex/generic/pgf/utilities/pgfkeys.code.tex)) (c:/TeXLive/2022/texmf-
dist/tex/la
tex/pgf/math/pgfmath.sty (c:/TeXLive/2022/texmf-
dist/tex/generic/pgf/math/pgfma
```

```
th.code.tex)) (c:/TeXLive/2022/texmf-
dist/tex/generic/pgf/utilities/pgffor.code
.tex
Package: pgffor 2023-01-15 v3.1.10 (3.1.10)
\pgffor@iter=\dimen302
\pgffor@skip=\dimen303
\pgffor@stack=\toks49
\pgffor@toks=\toks50
)) (c:/TeXLive/2022/texmf-
dist/tex/generic/pgf/frontendlayer/tikz/tikz.code.tex
Package: tikz 2023-01-15 v3.1.10 (3.1.10)

(c:/TeXLive/2022/texmf-
dist/tex/generic/pgf/libraries/pgflibraryplothandlers.co
de.tex
File: pgflibraryplothandlers.code.tex 2023-01-15 v3.1.10 (3.1.10)
\pgf@plot@mark@count=\count335
\pgfplotmarksize=\dimen304
)
\tikz@lastx=\dimen305
\tikz@lasty=\dimen306
\tikz@lastxsaved=\dimen307
\tikz@lastysaved=\dimen308
\tikz@lastmovetox=\dimen309
\tikz@lastmovetoy=\dimen310
\tikzleveldistance=\dimen311
\tikzsiblingdistance=\dimen312
\tikz@figbox=\box82
\tikz@figbox@bg=\box83
\tikz@tempbox=\box84
\tikz@tempbox@bg=\box85
\tikztreelevel=\count336
\tikznumberofchildren=\count337
\tikznumberofcurrentchild=\count338
\tikz@fig@count=\count339
(c:/TeXLive/2022/texmf-
dist/tex/generic/pgf/modules/pgfmodulematrix.code.tex
File: pgfmodulematrix.code.tex 2023-01-15 v3.1.10 (3.1.10)
\pgfmatrixcurrentrow=\count340
\pgfmatrixcurrentcolumn=\count341
\pgf@matrix@numberofcolumns=\count342
)
\tikz@expandcount=\count343

(c:/TeXLive/2022/texmf-
dist/tex/generic/pgf/frontendlayer/tikz/libraries/tikzli
brarytopaths.code.tex
File: tikzlibrarytopaths.code.tex 2023-01-15 v3.1.10 (3.1.10)
))) (c:/TeXLive/2022/texmf-dist/tex/latex/amsfonts/amsfonts.sty
Package: amsfonts 2013/01/14 v3.01 Basic AMSFonts support
\symAMSa=\mathgroup14
\symAMSb=\mathgroup15
LaTeX Font Info:    Redeclaring math symbol \hbar on input line 98.
LaTeX Info: Redefining \frak on input line 111.
```

```
) (c:/TeXLive/2022/texmf-dist/tex/latex/orcidlink/orcidlink.sty
Package: orcidlink 2021/06/11 v1.0.4 Linked ORCiD logo macro package

(c:/TeXLive/2022/texmf-
dist/tex/generic/pgf/frontendlayer/tikz/libraries/tikzli
brarysvg.path.code.tex
File: tikzlibrarysvg.path.code.tex 2023-01-15 v3.1.10 (3.1.10)

(c:/TeXLive/2022/texmf-
dist/tex/generic/pgf/libraries/pgflibrarysvg.path.code.t
ex
File: pgflibrarysvg.path.code.tex 2023-01-15 v3.1.10 (3.1.10)
(c:/TeXLive/2022/texmf-
dist/tex/generic/pgf/modules/pgfmoduleparser.code.tex
File: pgfmoduleparser.code.tex 2023-01-15 v3.1.10 (3.1.10)
\pgfparserdef@arg@count=\count344
)
\pgf@lib@svg@last@x=\dimen313
\pgf@lib@svg@last@y=\dimen314
\pgf@lib@svg@last@c@x=\dimen315
\pgf@lib@svg@last@c@y=\dimen316
\pgf@lib@svg@count=\count345
\pgf@lib@svg@max@num=\count346
))
\@curXheight=\skip163
)
Package translations Info: No language package found. I am going to use
`englis
h' as default language. on input line 61.
LaTeX Font Info:    Trying to load font information for T1+Merriwthr-OsF
on inp
ut line 61.
(c:/TeXLive/2022/texmf-dist/tex/latex/merriweather/T1Merriwthr-OsF.fd
File: T1Merriwthr-OsF.fd 2020/08/30 (autoinst) Font definitions for
T1/Merriwth
r-OsF.
)
LaTeX Font Info:    Font shape `T1/Merriwthr-OsF/m/n' will be
(Font)              scaled to size 7.5pt on input line 61.
(./main.aux)
\openout1 = `main.aux'.

LaTeX Font Info:    Checking defaults for OML/cmm/m/it on input line 61.
LaTeX Font Info:    ... okay on input line 61.
LaTeX Font Info:    Checking defaults for OMS/cmsy/m/n on input line 61.
LaTeX Font Info:    ... okay on input line 61.
LaTeX Font Info:    Checking defaults for OT1/cmr/m/n on input line 61.
LaTeX Font Info:    ... okay on input line 61.
LaTeX Font Info:    Checking defaults for T1/cmr/m/n on input line 61.
LaTeX Font Info:    ... okay on input line 61.
LaTeX Font Info:    Checking defaults for TS1/cmr/m/n on input line 61.
LaTeX Font Info:    ... okay on input line 61.
LaTeX Font Info:    Checking defaults for OMX/cmex/m/n on input line 61.
LaTeX Font Info:    ... okay on input line 61.
```

```
LaTeX Font Info:    Checking defaults for U/cmr/m/n on input line 61.
LaTeX Font Info:    ... okay on input line 61.
LaTeX Font Info:    Checking defaults for PD1/pdf/m/n on input line 61.
LaTeX Font Info:    ... okay on input line 61.
LaTeX Font Info:    Checking defaults for PU/pdf/m/n on input line 61.
LaTeX Font Info:    ... okay on input line 61.
LaTeX Info: Redefining \microtypecontext on input line 61.
Package microtype Info: Applying patch `item' on input line 61.
Package microtype Info: Applying patch `toc' on input line 61.
Package microtype Info: Applying patch `eqnum' on input line 61.

Package microtype Warning: Unable to apply patch `footnote' on input line
61.

Package microtype Info: Applying patch `verbatim' on input line 61.
Package microtype Info: Generating PDF output.
Package microtype Info: Character protrusion enabled (level 2).
Package microtype Info: Using default protrusion set `alltext'.
Package microtype Info: Automatic font expansion enabled (level 2),
(microtype)             stretch: 20, shrink: 20, step: 1, non-selected.
Package microtype Info: Using default expansion set `alltext-nott'.
LaTeX Info: Redefining \showhyphens on input line 61.
Package microtype Info: No adjustment of tracking.
Package microtype Info: No adjustment of interword spacing.
Package microtype Info: No adjustment of character kerning.
Package microtype Info: Loading generic protrusion settings for font
family
(microtype)             `Merriwthr-OsF' (encoding: T1).
(microtype)             For optimal results, create family-specific
settings.
(microtype)             See the microtype manual for details.
LaTeX Font Info:    Redeclaring symbol font `operators' on input line 61.
LaTeX Font Info:    Encoding `OT1' has changed to `T1' for symbol font
(Font)              `operators' in the math version `normal' on input
line 61.
LaTeX Font Info:    Overwriting symbol font `operators' in version
`normal'
(Font)                  OT1/cmr/m/n --> T1/Merriwthr-OsF/m/up on input
line 61.

LaTeX Font Info:    Encoding `OT1' has changed to `T1' for symbol font
(Font)              `operators' in the math version `bold' on input line
61.
LaTeX Font Info:    Overwriting symbol font `operators' in version `bold'
(Font)                  OT1/cmr/bx/n --> T1/Merriwthr-OsF/m/up on input
line 61
.
LaTeX Font Info:    Overwriting symbol font `operators' in version `bold'
(Font)                  T1/Merriwthr-OsF/m/up --> T1/Merriwthr-OsF/b/up
on inpu
t line 61.
LaTeX Font Info:    Redeclaring math alphabet \mathbf on input line 61.
LaTeX Font Info:    Redeclaring math alphabet \mathsf on input line 61.
```

```
LaTeX Font Info:    Overwriting math alphabet `\mathsf' in version
`normal'
(Font)                  OT1/cmss/m/n --> T1/MerriwthrSans-OsF/m/up on
input lin
e 61.
LaTeX Font Info:    Overwriting math alphabet `\mathsf' in version `bold'
(Font)                  OT1/cmss/bx/n --> T1/MerriwthrSans-OsF/m/up on
input li
ne 61.
LaTeX Font Info:    Redeclaring math alphabet \mathit on input line 61.
LaTeX Font Info:    Overwriting math alphabet `\mathit' in version
`normal'
(Font)                  OT1/cmr/m/it --> T1/Merriwthr-OsF/m/it on input
line 61
.
LaTeX Font Info:    Overwriting math alphabet `\mathit' in version `bold'
(Font)                  OT1/cmr/bx/it --> T1/Merriwthr-OsF/m/it on input
line 6
1.
LaTeX Font Info:    Redeclaring math alphabet \mathtt on input line 61.
LaTeX Font Info:    Overwriting math alphabet `\mathtt' in version
`normal'
(Font)                  OT1/cmtt/m/n --> T1/lmtt/m/up on input line 61.
LaTeX Font Info:    Overwriting math alphabet `\mathtt' in version `bold'
(Font)                  OT1/cmtt/m/n --> T1/lmtt/m/up on input line 61.
LaTeX Font Info:    Overwriting math alphabet `\mathsf' in version `bold'
(Font)                  T1/MerriwthrSans-OsF/m/up --> T1/MerriwthrSans-
OsF/b/up
 on input line 61.
LaTeX Font Info:    Overwriting math alphabet `\mathit' in version `bold'
(Font)                  T1/Merriwthr-OsF/m/it --> T1/Merriwthr-OsF/b/it
on inpu
t line 61.
\c@mv@tabular=\count347
\c@mv@boldtabular=\count348
(c:/TeXLive/2022/texmf-dist/tex/context/base/mkii/supp-pdf.mkii
[Loading MPS to PDF converter (version 2006.09.02).]
\scratchcounter=\count349
\scratchdimen=\dimen317
\scratchbox=\box86
\nofMPsegments=\count350
\nofMParguments=\count351
\everyMPshowfont=\toks51
\MPscratchCnt=\count352
\MPscratchDim=\dimen318
\MPnumerator=\count353
\makeMPintoPDFobject=\count354
\everyMPtoPDFconversion=\toks52
) (c:/TeXLive/2022/texmf-dist/tex/latex/epstopdf-pkg/epstopdf-base.sty
Package: epstopdf-base 2020-01-24 v2.11 Base part for package epstopdf
Package epstopdf-base Info: Redefining graphics rule for `.eps' on input
line 4
85.
(c:/TeXLive/2022/texmf-dist/tex/latex/latexconfig/epstopdf-sys.cfg
```

```
File: epstopdf-sys.cfg 2010/07/13 v1.3 Configuration of (r)epstopdf for
TeX Liv
e
))
*geometry* driver: auto-detecting
*geometry* detected driver: pdftex
*geometry* verbose mode - [ preamble ] result:
* driver: pdftex
* paper: a4paper
* layout: <same size as paper>
* layoutoffset:(h,v)=(0.0pt,0.0pt)
* modes: includefoot twoside
* h-part:(L,W,R)=(54.64pt, 488.22787pt, 54.64pt)
* v-part:(T,H,B)=(66.0pt, 745.04684pt, 34.0pt)
* \paperwidth=597.50787pt
* \paperheight=845.04684pt
* \textwidth=488.22787pt
* \textheight=715.04684pt
* \oddsidemargin=-17.62999pt
* \evensidemargin=-17.62999pt
* \topmargin=-47.76999pt
* \headheight=17.5pt
* \headsep=24.0pt
* \topskip=10.0pt
* \footskip=30.0pt
* \marginparwidth=48.0pt
* \marginparsep=10.0pt
* \columnsep=18.0pt
* \skip\footins=22.0pt plus 2.0pt
* \hoffset=0.0pt
* \voffset=0.0pt
* \mag=1000
* \@twocolumntrue
* \@twosidetrue
* \@mparswitchtrue
* \@reversemarginfalse
* (1in=72.27pt=25.4mm, 1cm=28.453pt)

Package hyperref Info: Link coloring ON on input line 61.
(./main.out) (./main.out)
\@outlinefile=\write4
\openout4 = `main.out'.

\@gscitedetails=\box87
\@gscitedetailsheight=\skip164
\@gsheadbox=\box88
\@gsheadboxheight=\skip165
LaTeX Font Info:    Font shape `T1/Merriwthr-OsF/b/n' will be
(Font)              scaled to size 6.5pt on input line 61.
LaTeX Font Info:    Calculating math sizes for size <7.5> on input line
61.

LaTeX Font Warning: Font shape `T1/Merriwthr-OsF/m/up' undefined
```

```
(Font)              using `T1/Merriwthr-OsF/m/n' instead on input line
61.

LaTeX Font Info:    Font shape `T1/Merriwthr-OsF/m/up' will be
(Font)              scaled to size 6.24973pt on input line 61.
LaTeX Font Info:    Font shape `T1/Merriwthr-OsF/m/up' will be
(Font)              scaled to size 5.24997pt on input line 61.
LaTeX Font Info:    Trying to load font information for U+eur on input
line 61.

(c:/TeXLive/2022/texmf-dist/tex/latex/amsfonts/ueur.fd
File: ueur.fd 2013/01/14 v3.01 Euler Roman
) (c:/TeXLive/2022/texmf-dist/tex/latex/microtype/mt-eur.cfg
File: mt-eur.cfg 2006/07/31 v1.1 microtype config. file: AMS Euler Roman
(RS)
)

LaTeX Font Warning: Font shape `OMS/cmsy/m/n' in size <7.5> not available
(Font)              size <7> substituted on input line 61.

LaTeX Font Info:    Trying to load font information for U+euf on input
line 61.

(c:/TeXLive/2022/texmf-dist/tex/latex/amsfonts/ueuf.fd
File: ueuf.fd 2013/01/14 v3.01 Euler Fraktur
) (c:/TeXLive/2022/texmf-dist/tex/latex/microtype/mt-euf.cfg
File: mt-euf.cfg 2006/07/03 v1.1 microtype config. file: AMS Euler
Fraktur (RS)

)
LaTeX Font Info:    Trying to load font information for U+eus on input
line 61.

(c:/TeXLive/2022/texmf-dist/tex/latex/amsfonts/ueus.fd
File: ueus.fd 2013/01/14 v3.01 Euler Script
) (c:/TeXLive/2022/texmf-dist/tex/latex/microtype/mt-eus.cfg
File: mt-eus.cfg 2006/07/28 v1.2 microtype config. file: AMS Euler Script
(RS)
)
LaTeX Font Info:    Trying to load font information for U+euex on input
line 61
.
(c:/TeXLive/2022/texmf-dist/tex/latex/amsfonts/ueuex.fd
File: ueuex.fd 2013/01/14 v3.01 Euler extra symbols
)

LaTeX Font Warning: Font shape `OML/cmm/m/it' in size <7.5> not available
(Font)              size <7> substituted on input line 61.

LaTeX Font Info:    Font shape `T1/Merriwthr-OsF/m/n' will be
(Font)              scaled to size 6.24973pt on input line 61.
LaTeX Font Info:    Font shape `T1/Merriwthr-OsF/m/n' will be
(Font)              scaled to size 5.24997pt on input line 61.
LaTeX Font Info:    Font shape `T1/Merriwthr-OsF/m/it' will be
```

```
(Font)              scaled to size 7.5pt on input line 61.
LaTeX Font Info:    Font shape `T1/Merriwthr-OsF/m/it' will be
(Font)              scaled to size 6.24973pt on input line 61.
LaTeX Font Info:    Font shape `T1/Merriwthr-OsF/m/it' will be
(Font)              scaled to size 5.24997pt on input line 61.

LaTeX Font Warning: Font shape `OT1/cmr/bx/n' in size <7.5> not available
(Font)              size <7> substituted on input line 61.

(c:/TeXLive/2022/texmf-dist/tex/latex/microtype/mt-cmr.cfg
File: mt-cmr.cfg 2013/05/19 v2.2 microtype config. file: Computer Modern
Roman
(RS)
)

LaTeX Font Warning: Font shape `OMS/cmsy/b/n' in size <7.5> not available
(Font)              size <7> substituted on input line 61.

LaTeX Font Info:    Trying to load font information for U+msa on input
line 61.

(c:/TeXLive/2022/texmf-dist/tex/latex/amsfonts/umsa.fd
File: umsa.fd 2013/01/14 v3.01 AMS symbols A
) (c:/TeXLive/2022/texmf-dist/tex/latex/microtype/mt-msa.cfg
File: mt-msa.cfg 2006/02/04 v1.1 microtype config. file: AMS symbols (a)
(RS)
)
LaTeX Font Info:    Trying to load font information for U+msb on input
line 61.

(c:/TeXLive/2022/texmf-dist/tex/latex/amsfonts/umsb.fd
File: umsb.fd 2013/01/14 v3.01 AMS symbols B
) (c:/TeXLive/2022/texmf-dist/tex/latex/microtype/mt-msb.cfg
File: mt-msb.cfg 2005/06/01 v1.0 microtype config. file: AMS symbols (b)
(RS)
)
LaTeX Font Info:    Font shape `T1/Merriwthr-OsF/m/n' will be
(Font)              scaled to size 8.0pt on input line 61.
LaTeX Font Info:    Font shape `T1/Merriwthr-OsF/m/it' will be
(Font)              scaled to size 8.0pt on input line 61.
LaTeX Font Info:    Font shape `T1/Merriwthr-OsF/b/it' will be
(Font)              scaled to size 8.0pt on input line 61.
Package caption Info: Begin \AtBeginDocument code.
Package caption Info: End \AtBeginDocument code.

(c:/TeXLive/2022/texmf-dist/tex/latex/translations/translations-basic-
dictionar
y-english.trsl
File: translations-basic-dictionary-english.trsl (english translation
file `tra
nslations-basic-dictionary')
)
Package translations Info: loading dictionary `translations-basic-
dictionary' f
```

```
or `english'. on input line 61.
TextBlockOrigin set to 4pc+6.64pt x 4pc+6pt
<oup.pdf, id=144, 49.18375pt x 48.18pt>
File: oup.pdf Graphic file (type pdf)
<use oup.pdf>
Package pdftex.def Info: oup.pdf  used on input line 78.
(pdftex.def)             Requested size: 59.24683pt x 58.038pt.
<gigascience-logo.pdf, id=145, 99.37125pt x 33.12375pt>
File: gigascience-logo.pdf Graphic file (type pdf)
<use gigascience-logo.pdf>
Package pdftex.def Info: gigascience-logo.pdf  used on input line 78.
(pdftex.def)             Requested size: 126.00902pt x 42.0pt.

Overfull \hbox (54.64pt too wide) in paragraph at lines 78--78
[][]
 []

LaTeX Font Info:    Font shape `T1/Merriwthr-OsF/m/n' will be
(Font)              scaled to size 14.0pt on input line 78.
LaTeX Font Info:    Font shape `T1/Merriwthr-OsF/m/n' will be
(Font)              scaled to size 8.99997pt on input line 78.
LaTeX Font Info:    Calculating math sizes for size <14> on input line
78.
LaTeX Font Info:    Font shape `T1/Merriwthr-OsF/m/up' will be
(Font)              scaled to size 14.0pt on input line 78.
LaTeX Font Info:    Font shape `T1/Merriwthr-OsF/m/up' will be
(Font)              scaled to size 11.66617pt on input line 78.
LaTeX Font Info:    Font shape `T1/Merriwthr-OsF/m/up' will be
(Font)              scaled to size 9.79996pt on input line 78.
LaTeX Font Info:    Font shape `T1/Merriwthr-OsF/m/n' will be
(Font)              scaled to size 11.66617pt on input line 78.
LaTeX Font Info:    Font shape `T1/Merriwthr-OsF/m/n' will be
(Font)              scaled to size 9.79996pt on input line 78.
LaTeX Font Info:    Font shape `T1/Merriwthr-OsF/m/it' will be
(Font)              scaled to size 14.0pt on input line 78.
LaTeX Font Info:    Font shape `T1/Merriwthr-OsF/m/it' will be
(Font)              scaled to size 11.66617pt on input line 78.
LaTeX Font Info:    Font shape `T1/Merriwthr-OsF/m/it' will be
(Font)              scaled to size 9.79996pt on input line 78.
LaTeX Font Info:    Font shape `T1/Merriwthr-OsF/b/n' will be
(Font)              scaled to size 18.0pt on input line 78.
LaTeX Font Info:    Font shape `T1/Merriwthr-OsF/m/n' will be
(Font)              scaled to size 13.0pt on input line 78.
LaTeX Font Info:    Calculating math sizes for size <13> on input line
78.
LaTeX Font Info:    Font shape `T1/Merriwthr-OsF/m/up' will be
(Font)              scaled to size 13.0pt on input line 78.
LaTeX Font Info:    Font shape `T1/Merriwthr-OsF/m/up' will be
(Font)              scaled to size 10.83287pt on input line 78.
LaTeX Font Info:    Font shape `T1/Merriwthr-OsF/m/up' will be
(Font)              scaled to size 9.09996pt on input line 78.

LaTeX Font Warning: Font shape `OMS/cmsy/m/n' in size <13> not available
(Font)              size <12> substituted on input line 78.
```

```
LaTeX Font Warning: Font shape `OML/cmm/m/it' in size <13> not available
(Font)              size <12> substituted on input line 78.

LaTeX Font Info:    Font shape `T1/Merriwthr-OsF/m/n' will be
(Font)              scaled to size 10.83287pt on input line 78.
LaTeX Font Info:    Font shape `T1/Merriwthr-OsF/m/n' will be
(Font)              scaled to size 9.09996pt on input line 78.
LaTeX Font Info:    Font shape `T1/Merriwthr-OsF/m/it' will be
(Font)              scaled to size 13.0pt on input line 78.
LaTeX Font Info:    Font shape `T1/Merriwthr-OsF/m/it' will be
(Font)              scaled to size 10.83287pt on input line 78.
LaTeX Font Info:    Font shape `T1/Merriwthr-OsF/m/it' will be
(Font)              scaled to size 9.09996pt on input line 78.

LaTeX Font Warning: Font shape `OT1/cmr/bx/n' in size <13> not available
(Font)              size <12> substituted on input line 78.


LaTeX Font Warning: Font shape `OMS/cmsy/b/n' in size <13> not available
(Font)              size <12> substituted on input line 78.

LaTeX Font Info:    Trying to load font information for TS1+Merriwthr-OsF
on in
put line 78.
(c:/TeXLive/2022/texmf-dist/tex/latex/merriweather/TS1Merriwthr-OsF.fd
File: TS1Merriwthr-OsF.fd 2020/08/30 (autoinst) Font definitions for
TS1/Merriw
thr-OsF.
)
LaTeX Font Info:    Font shape `TS1/Merriwthr-OsF/m/n' will be
(Font)              scaled to size 10.83287pt on input line 78.
Package microtype Info: Loading generic protrusion settings for font
family
(microtype)             `Merriwthr-OsF' (encoding: TS1).
(microtype)             For optimal results, create family-specific
settings.
(microtype)             See the microtype manual for details.
LaTeX Font Info:    Font shape `T1/Merriwthr-OsF/m/n' will be
(Font)              scaled to size 9.0pt on input line 78.
LaTeX Font Info:    Font shape `T1/Merriwthr-OsF/m/up' will be
(Font)              scaled to size 9.0pt on input line 78.
LaTeX Font Info:    Font shape `T1/Merriwthr-OsF/m/up' will be
(Font)              scaled to size 7.0pt on input line 78.
LaTeX Font Info:    Font shape `T1/Merriwthr-OsF/m/up' will be
(Font)              scaled to size 5.0pt on input line 78.
LaTeX Font Info:    Font shape `T1/Merriwthr-OsF/m/n' will be
(Font)              scaled to size 7.0pt on input line 78.
LaTeX Font Info:    Font shape `T1/Merriwthr-OsF/m/n' will be
(Font)              scaled to size 5.0pt on input line 78.
LaTeX Font Info:    Font shape `T1/Merriwthr-OsF/m/it' will be
(Font)              scaled to size 9.0pt on input line 78.
LaTeX Font Info:    Font shape `T1/Merriwthr-OsF/m/it' will be
```

```
(Font)              scaled to size 7.0pt on input line 78.
LaTeX Font Info:    Font shape `T1/Merriwthr-OsF/m/it' will be
(Font)              scaled to size 5.0pt on input line 78.
LaTeX Font Info:    Font shape `T1/Merriwthr-OsF/m/n' will be
(Font)              scaled to size 6.5pt on input line 78.
LaTeX Font Info:    Calculating math sizes for size <6.5> on input line
78.
LaTeX Font Info:    Font shape `T1/Merriwthr-OsF/m/up' will be
(Font)              scaled to size 6.5pt on input line 78.
LaTeX Font Info:    Font shape `T1/Merriwthr-OsF/m/up' will be
(Font)              scaled to size 5.41643pt on input line 78.
LaTeX Font Info:    Font shape `T1/Merriwthr-OsF/m/up' will be
(Font)              scaled to size 4.54997pt on input line 78.

LaTeX Font Warning: Font shape `OMS/cmsy/m/n' in size <6.5> not available
(Font)              size <6> substituted on input line 78.


LaTeX Font Warning: Font shape `OMS/cmsy/m/n' in size <5.41643> not
available
(Font)              size <5> substituted on input line 78.


LaTeX Font Warning: Font shape `OMS/cmsy/m/n' in size <4.54997> not
available
(Font)              size <5> substituted on input line 78.


LaTeX Font Warning: Font shape `OML/cmm/m/it' in size <6.5> not available
(Font)              size <6> substituted on input line 78.


LaTeX Font Warning: Font shape `OML/cmm/m/it' in size <5.41643> not
available
(Font)              size <5> substituted on input line 78.


LaTeX Font Warning: Font shape `OML/cmm/m/it' in size <4.54997> not
available
(Font)              size <5> substituted on input line 78.

LaTeX Font Info:    Font shape `T1/Merriwthr-OsF/m/n' will be
(Font)              scaled to size 5.41643pt on input line 78.
LaTeX Font Info:    Font shape `T1/Merriwthr-OsF/m/n' will be
(Font)              scaled to size 4.54997pt on input line 78.
LaTeX Font Info:    Font shape `T1/Merriwthr-OsF/m/it' will be
(Font)              scaled to size 6.5pt on input line 78.
LaTeX Font Info:    Font shape `T1/Merriwthr-OsF/m/it' will be
(Font)              scaled to size 5.41643pt on input line 78.
LaTeX Font Info:    Font shape `T1/Merriwthr-OsF/m/it' will be
(Font)              scaled to size 4.54997pt on input line 78.

LaTeX Font Warning: Font shape `OT1/cmr/bx/n' in size <6.5> not available
(Font)              size <6> substituted on input line 78.
```

```
LaTeX Font Warning: Font shape `OT1/cmr/bx/n' in size <5.41643> not
available
(Font)              size <5> substituted on input line 78.


LaTeX Font Warning: Font shape `OT1/cmr/bx/n' in size <4.54997> not
available
(Font)              size <5> substituted on input line 78.


LaTeX Font Warning: Font shape `OMS/cmsy/b/n' in size <6.5> not available
(Font)              size <6> substituted on input line 78.


LaTeX Font Warning: Font shape `OMS/cmsy/b/n' in size <5.41643> not
available
(Font)              size <5> substituted on input line 78.


LaTeX Font Warning: Font shape `OMS/cmsy/b/n' in size <4.54997> not
available
(Font)              size <5> substituted on input line 78.

LaTeX Font Info:    Font shape `TS1/Merriwthr-OsF/m/n' will be
(Font)              scaled to size 5.41643pt on input line 78.

Overfull \hbox (54.64pt too wide) in paragraph at lines 78--78
[][][]
 []

LaTeX Font Info:    Font shape `T1/Merriwthr-OsF/b/n' will be
(Font)              scaled to size 10.0pt on input line 78.
LaTeX Font Info:    Font shape `T1/Merriwthr-OsF/b/n' will be
(Font)              scaled to size 8.0pt on input line 78.
LaTeX Font Info:    Trying to load font information for T1+lmtt on input
line 7
8.
(c:/TeXLive/2022/texmf-dist/tex/latex/lm/t1lmtt.fd
File: t1lmtt.fd 2015/05/01 v1.6.1 Font defs for Latin Modern
)
Package microtype Info: Loading generic protrusion settings for font
family
(microtype)             `lmtt' (encoding: T1).
(microtype)             For optimal results, create family-specific
settings.
(microtype)             See the microtype manual for details.

Overfull \hbox (54.64pt too wide) in paragraph at lines 78--78
[][][]
 []
```

```
Underfull \vbox (badness 10000) has occurred while \output is active []

LaTeX Font Info:    Font shape `T1/Merriwthr-OsF/b/n' will be
(Font)              scaled to size 7.5pt on input line 93.

Underfull \vbox (badness 10000) has occurred while \output is active []

LaTeX Font Info:    Font shape `T1/Merriwthr-OsF/m/n' will be
(Font)              scaled to size 7.8pt on input line 94.
LaTeX Font Info:    Font shape `T1/Merriwthr-OsF/b/n' will be
(Font)              scaled to size 7.8pt on input line 94.
[1{c:/TeXLive/2022/texmf-var/fonts/map/pdftex/updmap/pdftex.map}


 <./oup.pdf> <./gigascience-logo.pdf>]

Package natbib Warning: Author undefined for
citation`Himmelstein_Daniel_Scott_
2017'
(natbib)                on page 2 on input line 95.


Package natbib Warning: Author undefined for
citation`Vassilis_N_Ioannidis_2020
'
(natbib)                on page 2 on input line 95.


Package natbib Warning: Author undefined for citation`Zhang_Rui_2021'
(natbib)                on page 2 on input line 95.


Package natbib Warning: Author undefined for citation`Sosa_Daniel_N_2020'
(natbib)                on page 2 on input line 97.


Package natbib Warning: Author undefined for
citation`Sang_Shengtian_2019'
(natbib)                on page 2 on input line 97.


Package natbib Warning: Author undefined for citation`Liu_Yushan_2021'
(natbib)                on page 2 on input line 99.


Package natbib Warning: Author undefined for
citation`Himmelstein_Daniel_Scott_
2017'
(natbib)                on page 2 on input line 99.

LaTeX Font Info:    Font shape `T1/Merriwthr-OsF/b/n' will be
(Font)              scaled to size 8.5pt on input line 104.
LaTeX Font Info:    Font shape `T1/Merriwthr-OsF/b/sl' in size <7.5> not
availa
```

```
ble
(Font)              Font shape `T1/Merriwthr-OsF/b/it' tried instead on
input l
ine 106.
LaTeX Font Info:    Font shape `T1/Merriwthr-OsF/b/it' will be
(Font)              scaled to size 7.5pt on input line 106.
LaTeX Font Info:    Font shape `T1/Merriwthr-OsF/m/it' will be
(Font)              scaled to size 7.8pt on input line 108.
[2]

! LaTeX Error: File `./Figures/main_figure1' not found.

See the LaTeX manual or LaTeX Companion for explanation.
Type  H <return>  for immediate help.
 ...

l.111 ...h=0.98\textwidth]{./Figures/main_figure1}

I could not locate the file with any of these extensions:
.pdf,.png,.jpg,.mps,.jpeg,.jbig2,.jb2,.PDF,.PNG,.JPG,.JPEG,.JBIG2,.JB2,.e
ps
Try typing  <return>  to proceed.
If that doesn't work, type  X <return>  to quit.

LaTeX Font Info:    Font shape `T1/Merriwthr-OsF/m/n' will be
(Font)              scaled to size 6.0pt on input line 112.
LaTeX Font Info:    Font shape `T1/Merriwthr-OsF/b/n' will be
(Font)              scaled to size 6.0pt on input line 112.
LaTeX Font Info:    Font shape `TS1/Merriwthr-OsF/m/n' will be
(Font)              scaled to size 7.5pt on input line 118.

Underfull \hbox (badness 1142) in paragraph at lines 119--120
\T1/Merriwthr-OsF/m/n/7.5 (+20) cess-ing (NLP) tech-niques to ex-tract
se-man-t
ic triples with
 []

LaTeX Font Info:    Font shape `T1/Merriwthr-OsF/m/up' will be
(Font)              scaled to size 7.5pt on input line 124.

LaTeX Font Warning: Command \footnotesize invalid in math mode on input
line 12
5.

LaTeX Font Info:    Font shape `T1/Merriwthr-OsF/m/n' will be
(Font)              scaled to size 6.25008pt on input line 125.
LaTeX Font Info:    Calculating math sizes for size <6.25008> on input
line 127
.
LaTeX Font Info:    Font shape `T1/Merriwthr-OsF/m/up' will be
(Font)              scaled to size 6.25008pt on input line 127.
LaTeX Font Info:    Font shape `T1/Merriwthr-OsF/m/up' will be
(Font)              scaled to size 5.20816pt on input line 127.
LaTeX Font Info:    Font shape `T1/Merriwthr-OsF/m/up' will be
```

```
(Font)              scaled to size 4.37503pt on input line 127.

LaTeX Font Warning: Font shape `OMS/cmsy/m/n' in size <4.37503> not
available
(Font)              size <5> substituted on input line 127.


LaTeX Font Warning: Font shape `OML/cmm/m/it' in size <4.37503> not
available
(Font)              size <5> substituted on input line 127.

LaTeX Font Info:    Font shape `T1/Merriwthr-OsF/m/n' will be
(Font)              scaled to size 5.20816pt on input line 127.
LaTeX Font Info:    Font shape `T1/Merriwthr-OsF/m/n' will be
(Font)              scaled to size 4.37503pt on input line 127.
LaTeX Font Info:    Font shape `T1/Merriwthr-OsF/m/it' will be
(Font)              scaled to size 6.25008pt on input line 127.
LaTeX Font Info:    Font shape `T1/Merriwthr-OsF/m/it' will be
(Font)              scaled to size 5.20816pt on input line 127.
LaTeX Font Info:    Font shape `T1/Merriwthr-OsF/m/it' will be
(Font)              scaled to size 4.37503pt on input line 127.

LaTeX Font Warning: Font shape `OT1/cmr/bx/n' in size <4.37503> not
available
(Font)              size <5> substituted on input line 127.


LaTeX Font Warning: Font shape `OMS/cmsy/b/n' in size <4.37503> not
available
(Font)              size <5> substituted on input line 127.

LaTeX Font Info:    Calculating math sizes for size <> on input line 127.
LaTeX Font Info:    Font shape `T1/Merriwthr-OsF/m/up' will be
(Font)              scaled to size 1.0pt on input line 127.
LaTeX Font Info:    Font shape `T1/Merriwthr-OsF/m/up' will be
(Font)              scaled to size 0.8333pt on input line 127.
LaTeX Font Info:    Font shape `T1/Merriwthr-OsF/m/up' will be
(Font)              scaled to size 0.7pt on input line 127.

LaTeX Font Warning: Font shape `OMS/cmsy/m/n' in size <> not available
(Font)              size <5> substituted on input line 127.


LaTeX Font Warning: Font shape `OMS/cmsy/m/n' in size <0.8333> not
available
(Font)              size <5> substituted on input line 127.


LaTeX Font Warning: Font shape `OMS/cmsy/m/n' in size <0.7> not available
(Font)              size <5> substituted on input line 127.


LaTeX Font Warning: Font shape `OML/cmm/m/it' in size <> not available
(Font)              size <5> substituted on input line 127.
```

```
LaTeX Font Warning: Font shape `OML/cmm/m/it' in size <0.8333> not
available
(Font)              size <5> substituted on input line 127.


LaTeX Font Warning: Font shape `OML/cmm/m/it' in size <0.7> not available
(Font)              size <5> substituted on input line 127.

LaTeX Font Info:    Font shape `T1/Merriwthr-OsF/m/n' will be
(Font)              scaled to size 1.0pt on input line 127.
LaTeX Font Info:    Font shape `T1/Merriwthr-OsF/m/n' will be
(Font)              scaled to size 0.8333pt on input line 127.
LaTeX Font Info:    Font shape `T1/Merriwthr-OsF/m/n' will be
(Font)              scaled to size 0.7pt on input line 127.
LaTeX Font Info:    Font shape `T1/Merriwthr-OsF/m/it' will be
(Font)              scaled to size 1.0pt on input line 127.
LaTeX Font Info:    Font shape `T1/Merriwthr-OsF/m/it' will be
(Font)              scaled to size 0.8333pt on input line 127.
LaTeX Font Info:    Font shape `T1/Merriwthr-OsF/m/it' will be
(Font)              scaled to size 0.7pt on input line 127.

LaTeX Font Warning: Font shape `OT1/cmr/bx/n' in size <> not available
(Font)              size <5> substituted on input line 127.


LaTeX Font Warning: Font shape `OT1/cmr/bx/n' in size <0.8333> not
available
(Font)              size <5> substituted on input line 127.


LaTeX Font Warning: Font shape `OT1/cmr/bx/n' in size <0.7> not available
(Font)              size <5> substituted on input line 127.


LaTeX Font Warning: Font shape `OMS/cmsy/b/n' in size <> not available
(Font)              size <5> substituted on input line 127.


LaTeX Font Warning: Font shape `OMS/cmsy/b/n' in size <0.8333> not
available
(Font)              size <5> substituted on input line 127.


LaTeX Font Warning: Font shape `OMS/cmsy/b/n' in size <0.7> not available
(Font)              size <5> substituted on input line 127.

LaTeX Font Info:    Font shape `T1/Merriwthr-OsF/b/n' will be
(Font)              scaled to size 7.0pt on input line 134.

Overfull \hbox (4.67296pt too wide) in paragraph at lines 136--149
 [][]
 []
```

```
LaTeX Font Warning: Font shape `T1/Merriwthr-OsF/b/up' undefined
(Font)              using `T1/Merriwthr-OsF/b/n' instead on input line
163.

LaTeX Font Info:    Font shape `T1/Merriwthr-OsF/b/up' will be
(Font)              scaled to size 6.24973pt on input line 163.
LaTeX Font Info:    Font shape `T1/Merriwthr-OsF/b/up' will be
(Font)              scaled to size 5.24997pt on input line 163.

LaTeX Font Warning: Font shape `OML/cmm/b/it' in size <7.5> not available
(Font)              size <7> substituted on input line 163.

LaTeX Font Info:    Font shape `T1/Merriwthr-OsF/b/n' will be
(Font)              scaled to size 6.24973pt on input line 163.
LaTeX Font Info:    Font shape `T1/Merriwthr-OsF/b/n' will be
(Font)              scaled to size 5.24997pt on input line 163.
LaTeX Font Info:    Font shape `T1/Merriwthr-OsF/b/it' will be
(Font)              scaled to size 6.24973pt on input line 163.
LaTeX Font Info:    Font shape `T1/Merriwthr-OsF/b/it' will be
(Font)              scaled to size 5.24997pt on input line 163.
LaTeX Font Info:    Font shape `T1/Merriwthr-OsF/b/up' will be
(Font)              scaled to size 7.5pt on input line 163.

LaTeX Warning: File `./Figures/main_figure2.jpg' not found on input line
167.


! Package pdftex.def Error: File `./Figures/main_figure2.jpg' not found:
using
draft setting.

See the pdftex.def package documentation for explanation.
Type  H <return>  for immediate help.
 ...

l.167 ...98\textwidth]{./Figures/main_figure2.jpg}

Try typing  <return>  to proceed.
If that doesn't work, type  X <return>  to quit.

[3] [4]

LaTeX Font Warning: Command \footnotesize invalid in math mode on input
line 17
8.

LaTeX Font Info:    Font shape `T1/Merriwthr-OsF/b/up' will be
(Font)              scaled to size 6.25008pt on input line 179.
LaTeX Font Info:    Font shape `T1/Merriwthr-OsF/b/up' will be
(Font)              scaled to size 5.20816pt on input line 179.
LaTeX Font Info:    Font shape `T1/Merriwthr-OsF/b/up' will be
(Font)              scaled to size 4.37503pt on input line 179.
```

```
LaTeX Font Warning: Font shape `OML/cmm/b/it' in size <4.37503> not
available
(Font)              size <5> substituted on input line 179.

LaTeX Font Info:    Font shape `T1/Merriwthr-OsF/b/n' will be
(Font)              scaled to size 6.25008pt on input line 179.
LaTeX Font Info:    Font shape `T1/Merriwthr-OsF/b/n' will be
(Font)              scaled to size 5.20816pt on input line 179.
LaTeX Font Info:    Font shape `T1/Merriwthr-OsF/b/n' will be
(Font)              scaled to size 4.37503pt on input line 179.
LaTeX Font Info:    Font shape `T1/Merriwthr-OsF/b/it' will be
(Font)              scaled to size 6.25008pt on input line 179.
LaTeX Font Info:    Font shape `T1/Merriwthr-OsF/b/it' will be
(Font)              scaled to size 5.20816pt on input line 179.
LaTeX Font Info:    Font shape `T1/Merriwthr-OsF/b/it' will be
(Font)              scaled to size 4.37503pt on input line 179.

Underfull \hbox (badness 10000) in paragraph at lines 194--195
[]\T1/Merriwthr-OsF/b/up/7.5 (+20) States\T1/Merriwthr-OsF/m/up/7.5 (+20)
: Eac
h state $\T1/Merriwthr-OsF/m/it/7.5 (+20) s[]$ \T1/Merriwthr-OsF/m/up/7.5
(+20)
 at time $\T1/Merriwthr-OsF/m/it/7.5 (+20) t$ \T1/Merriwthr-OsF/m/up/7.5
(+20)
is de-fined as
 []


Underfull \hbox (badness 10000) in paragraph at lines 194--195
\T1/Merriwthr-OsF/m/it/7.5 (+20) s[] \T1/Merriwthr-OsF/m/up/7.5 (+20) =
[][]\T
1/Merriwthr-OsF/m/it/7.5 (+20) v[]\T1/Merriwthr-OsF/m/up/7.5 (+20) ,
\T1/Merriw
thr-OsF/m/it/7.5 (+20) v[]\T1/Merriwthr-OsF/m/up/7.5 (+20) ,
(\T1/Merriwthr-OsF
/m/it/7.5 (+20) v[]\T1/Merriwthr-OsF/m/up/7.5 (+20) , \T1/Merriwthr-
OsF/m/it/7.
5 (+20) e[]\T1/Merriwthr-OsF/m/up/7.5 (+20) ), [] , (\T1/Merriwthr-
OsF/m/it/7.5
 (+20) v[]\T1/Merriwthr-OsF/m/up/7.5 (+20) , \T1/Merriwthr-OsF/m/it/7.5
(+20) e
[]\T1/Merriwthr-OsF/m/up/7.5 (+20) )[]$ where
 []


Underfull \hbox (badness 1789) in paragraph at lines 194--195
\T1/Merriwthr-OsF/m/up/7.5 (+20) sents the node where the agent lo-cates
at tim
e $\T1/Merriwthr-OsF/m/it/7.5 (+20) t$\T1/Merriwthr-OsF/m/up/7.5 (+20) ;
the tu
-ple
 []
```

```
LaTeX Font Warning: Command \scriptsize invalid in math mode on input
line 200.


LaTeX Font Info:    Font shape `T1/Merriwthr-OsF/m/n' will be
(Font)              scaled to size 5.00003pt on input line 200.
LaTeX Font Info:    Calculating math sizes for size <5.00003> on input
line 203
.
LaTeX Font Info:    Font shape `T1/Merriwthr-OsF/m/up' will be
(Font)              scaled to size 5.00003pt on input line 203.
LaTeX Font Info:    Font shape `T1/Merriwthr-OsF/m/up' will be
(Font)              scaled to size 4.1665pt on input line 203.
LaTeX Font Info:    Font shape `T1/Merriwthr-OsF/m/up' will be
(Font)              scaled to size 3.5pt on input line 203.

LaTeX Font Warning: Font shape `OMS/cmsy/m/n' in size <4.1665> not
available
(Font)              size <5> substituted on input line 203.


LaTeX Font Warning: Font shape `OMS/cmsy/m/n' in size <3.5> not available
(Font)              size <5> substituted on input line 203.


LaTeX Font Warning: Font shape `OML/cmm/m/it' in size <4.1665> not
available
(Font)              size <5> substituted on input line 203.


LaTeX Font Warning: Font shape `OML/cmm/m/it' in size <3.5> not available
(Font)              size <5> substituted on input line 203.

LaTeX Font Info:    Font shape `T1/Merriwthr-OsF/m/n' will be
(Font)              scaled to size 4.1665pt on input line 203.
LaTeX Font Info:    Font shape `T1/Merriwthr-OsF/m/n' will be
(Font)              scaled to size 3.5pt on input line 203.
LaTeX Font Info:    Font shape `T1/Merriwthr-OsF/m/it' will be
(Font)              scaled to size 5.00003pt on input line 203.
LaTeX Font Info:    Font shape `T1/Merriwthr-OsF/m/it' will be
(Font)              scaled to size 4.1665pt on input line 203.
LaTeX Font Info:    Font shape `T1/Merriwthr-OsF/m/it' will be
(Font)              scaled to size 3.5pt on input line 203.

LaTeX Font Warning: Font shape `OT1/cmr/bx/n' in size <4.1665> not
available
(Font)              size <5> substituted on input line 203.


LaTeX Font Warning: Font shape `OT1/cmr/bx/n' in size <3.5> not available
(Font)              size <5> substituted on input line 203.
```

```
LaTeX Font Warning: Font shape `OMS/cmsy/b/n' in size <4.1665> not
available
(Font)              size <5> substituted on input line 203.


LaTeX Font Warning: Font shape `OMS/cmsy/b/n' in size <3.5> not available
(Font)              size <5> substituted on input line 203.


Overfull \hbox (6.1483pt too wide) detected at line 209
\T1/Merriwthr-OsF/m/it/7.5 R[] \T1/Merriwthr-OsF/m/n/7.5 = []
 []


LaTeX Font Warning: Command \footnotesize invalid in math mode on input
line 21
4.


LaTeX Font Warning: Command \footnotesize invalid in math mode on input
line 22
1.


LaTeX Font Warning: Command \footnotesize invalid in math mode on input
line 22
8.

[5]

LaTeX Font Warning: Command \footnotesize invalid in math mode on input
line 23
5.


LaTeX Font Warning: Command \footnotesize invalid in math mode on input
line 24
0.

Package textcomp Info: Symbol \textrightarrow not provided by
(textcomp)             font family Merriwthr-OsF in TS1 encoding.
(textcomp)             Default family used instead on input line 244.

LaTeX Font Warning: Font shape `TS1/cmr/m/n' in size <7.5> not available
(Font)              size <7> substituted on input line 244.

Package textcomp Info: Symbol \textrightarrow not provided by
(textcomp)             font family Merriwthr-OsF in TS1 encoding.
(textcomp)             Default family used instead on input line 244.
Package textcomp Info: Symbol \textrightarrow not provided by
(textcomp)             font family Merriwthr-OsF in TS1 encoding.
(textcomp)             Default family used instead on input line 244.
```

```
LaTeX Font Warning: Command \footnotesize invalid in math mode on input
line 24
6.


LaTeX Font Warning: Command \footnotesize invalid in math mode on input
line 25
2.


LaTeX Font Warning: Command \footnotesize invalid in math mode on input
line 25
8.


LaTeX Font Warning: Command \footnotesize invalid in math mode on input
line 26
5.


LaTeX Font Warning: Command \footnotesize invalid in math mode on input
line 27
1.


Package natbib Warning: Author undefined for citation`Zhao_Kangzhi_2020'
(natbib)                on page 6 on input line 276.


LaTeX Font Warning: Command \footnotesize invalid in math mode on input
line 29
0.


LaTeX Font Warning: Command \footnotesize invalid in math mode on input
line 29
6.


LaTeX Font Warning: Command \footnotesize invalid in math mode on input
line 30
5.


LaTeX Font Warning: Command \footnotesize invalid in math mode on input
line 31
2.


Underfull \hbox (badness 1242) in paragraph at lines 317--318
\T1/Merriwthr-OsF/m/up/7.5 (+20) pos-i-tive drug-disease pairs ("treat"
cat-e-g
ory) or their 3-hop
 []
```

```
LaTeX Font Warning: Command \footnotesize invalid in math mode on input
line 31
9.

[6]

LaTeX Font Warning: Command \footnotesize invalid in math mode on input
line 33
7.


Overfull \hbox (6.06375pt too wide) in paragraph at lines 349--371
 [][]
 []

LaTeX Font Info:    Font shape `T1/Merriwthr-OsF/m/up' will be
(Font)              scaled to size 6.0pt on input line 372.
LaTeX Font Info:    Font shape `T1/Merriwthr-OsF/m/it' will be
(Font)              scaled to size 6.0pt on input line 372.

Underfull \hbox (badness 1412) in paragraph at lines 378--379
\T1/Merriwthr-OsF/m/up/7.5 (+20) For drug re-pur-pos-ing pre-dic-tion
eval-u-a-
tion, we com-pare the
 []


Underfull \hbox (badness 3118) in paragraph at lines 378--379
\T1/lmtt/m/n/7.5 KGML-xDTD \T1/Merriwthr-OsF/m/up/7.5 (+20) model frame-
work ag
ainst sev-eral state-of-the-art
 []


! LaTeX Error: File `./Figures/main_figure3' not found.

See the LaTeX manual or LaTeX Companion for explanation.
Type  H <return>  for immediate help.
 ...

l.386 ...h=0.98\textwidth]{./Figures/main_figure3}

I could not locate the file with any of these extensions:
.pdf,.png,.jpg,.mps,.jpeg,.jbig2,.jb2,.PDF,.PNG,.JPG,.JPEG,.JBIG2,.JB2,.e
ps
Try typing  <return>  to proceed.
If that doesn't work, type  X <return>  to quit.

[7]
Underfull \vbox (badness 10000) has occurred while \output is active []

[8]
```

```
! LaTeX Error: File `./Figures/main_figure4' not found.

See the LaTeX manual or LaTeX Companion for explanation.
Type  H <return>  for immediate help.
 ...

l.452 ...ics[height=6.5cm]{./Figures/main_figure4}

I could not locate the file with any of these extensions:
.pdf,.png,.jpg,.mps,.jpeg,.jbig2,.jb2,.PDF,.PNG,.JPG,.JPEG,.JBIG2,.JB2,.e
ps
Try typing  <return>  to proceed.
If that doesn't work, type  X <return>  to quit.


! LaTeX Error: File `./Figures/main_figure5' not found.

See the LaTeX manual or LaTeX Companion for explanation.
Type  H <return>  for immediate help.
 ...

l.460 ...m, height=10.3cm]{./Figures/main_figure5}

I could not locate the file with any of these extensions:
.pdf,.png,.jpg,.mps,.jpeg,.jbig2,.jb2,.PDF,.PNG,.JPG,.JPEG,.JBIG2,.JB2,.e
ps
Try typing  <return>  to proceed.
If that doesn't work, type  X <return>  to quit.


Underfull \vbox (badness 6641) has occurred while \output is active []

[9]

! LaTeX Error: File `./Figures/main_figure6' not found.

See the LaTeX manual or LaTeX Companion for explanation.
Type  H <return>  for immediate help.
 ...

l.500 ...hics[height=17cm]{./Figures/main_figure6}

I could not locate the file with any of these extensions:
.pdf,.png,.jpg,.mps,.jpeg,.jbig2,.jb2,.PDF,.PNG,.JPG,.JPEG,.JBIG2,.JB2,.e
ps
Try typing  <return>  to proceed.
If that doesn't work, type  X <return>  to quit.


Underfull \vbox (badness 10000) has occurred while \output is active []


! LaTeX Error: File `./Figures/main_figure7' not found.
```

```
See the LaTeX manual or LaTeX Companion for explanation.
Type  H <return>  for immediate help.
 ...

l.511 ...phics[height=6cm]{./Figures/main_figure7}

I could not locate the file with any of these extensions:
.pdf,.png,.jpg,.mps,.jpeg,.jbig2,.jb2,.PDF,.PNG,.JPG,.JPEG,.JBIG2,.JB2,.e
ps
Try typing  <return>  to proceed.
If that doesn't work, type  X <return>  to quit.


! LaTeX Error: File `./Figures/main_figure8' not found.

See the LaTeX manual or LaTeX Companion for explanation.
Type  H <return>  for immediate help.
 ...

l.519 ...phics[height=6cm]{./Figures/main_figure8}

I could not locate the file with any of these extensions:
.pdf,.png,.jpg,.mps,.jpeg,.jbig2,.jb2,.PDF,.PNG,.JPG,.JPEG,.JBIG2,.JB2,.e
ps
Try typing  <return>  to proceed.
If that doesn't work, type  X <return>  to quit.

[10]
Underfull \hbox (badness 10000) in paragraph at lines 580--605

 []


Underfull \hbox (badness 10000) in paragraph at lines 607--608
\T1/Merriwthr-OsF/b/up/7.5 (+20) Molecular Data Provider\T1/Merriwthr-
OsF/m/up/
7.5 (+20) : A knowledge-centric data
 []


Underfull \hbox (badness 1043) in paragraph at lines 607--608
\T1/Merriwthr-OsF/m/up/7.5 (+20) provider for sys-tems chem-i-cal bi-ol-
ogy, as
 part of the NCATS
 []


Underfull \hbox (badness 10000) in paragraph at lines 607--608
\T1/Merriwthr-OsF/m/up/7.5 (+20) Biomed-i-cal Data Trans-la-tor
(^^PTrans-la-to
r^^Q). See more in
 []
```

```
Underfull \hbox (badness 10000) in paragraph at lines 607--608
[][]$\T1/lmtt/m/n/7.5 https : / / github . com / NCATSTranslator /
Translator-[
]All / wiki /
 []

[11]
Underfull \hbox (badness 10000) in paragraph at lines 632--633
[]\T1/Merriwthr-OsF/m/up/7.5 (+20) Berdigaliyev N, Aljo-fan M. An
overview of d
rug dis-
 []


Underfull \hbox (badness 10000) in paragraph at lines 632--633
\T1/Merriwthr-OsF/m/up/7.5 (+20) cov-ery and de-vel-op-ment. Fu-ture
Medic-i-na
l Chem-istry
 []


Underfull \hbox (badness 10000) in paragraph at lines 642--643
\T1/Merriwthr-OsF/m/up/7.5 (+20) Generation Phe-no-typic Screen-ing in
Early Dr
ug Dis-
 []


Underfull \hbox (badness 10000) in paragraph at lines 642--643
\T1/Merriwthr-OsF/m/up/7.5 (+20) cov-ery for In-fec-tious Dis-eases.
Trends in
Par-a-sitol-ogy
 []


Underfull \hbox (badness 10000) in paragraph at lines 666--667
[]\T1/Merriwthr-OsF/m/up/7.5 (+20) Percha B, Alt-man RB. A global net-
work of b
iomed-
 []


Underfull \hbox (badness 10000) in paragraph at lines 666--667
\T1/Merriwthr-OsF/m/up/7.5 (+20) i-cal re-la-tion-ships de-rived from
text. Bio
in-for-mat-ics
 []


Underfull \hbox (badness 3312) in paragraph at lines 686--687
[]\T1/Merriwthr-OsF/m/up/7.5 (+20) Trouillon T, Welbl J, Riedel S,
Gaussier E,
Bouchard G.
```

```
 []


Underfull \hbox (badness 4752) in paragraph at lines 686--687
\T1/Merriwthr-OsF/m/up/7.5 (+20) Com-plex Em-bed-dings for Sim-ple Link
Pre-dic
-tion. 2016.
 []


Underfull \vbox (badness 10000) has occurred while \output is active []

[12]
Underfull \hbox (badness 2469) in paragraph at lines 694--695
\T1/Merriwthr-OsF/m/up/7.5 (+20) DeL: A Knowl-edge Graph Em-bed-ding
Based Meth
od for
 []


Underfull \hbox (badness 10000) in paragraph at lines 698--699
[]\T1/Merriwthr-OsF/m/up/7.5 (+20) Li Y. Re-in-force-ment Learn-ing Ap-
pli-ca-t
ions. 2019.
 []


Underfull \vbox (badness 10000) has occurred while \output is active []


Underfull \hbox (badness 3219) in paragraph at lines 736--737
\T1/Merriwthr-OsF/m/up/7.5 (+20) IEEE Trans-ac-tions on Knowl-edge and
Data En-
gi-neer-ing
 []


Underfull \hbox (badness 4181) in paragraph at lines 748--749
\T1/Merriwthr-OsF/m/up/7.5 (+20) works for Au-tonomous Nav-i-ga-tion.
Neu-ral C
om-pu-ta-tion
 []


Underfull \vbox (badness 10000) has occurred while \output is active []

[13]
Underfull \hbox (badness 3281) in paragraph at lines 770--771
[]\T1/Merriwthr-OsF/m/up/7.5 (+20) Driessche T, Collen D, Chuah M. Vi-ral
Vecto
r-Mediated
 []
```

```
Underfull \hbox (badness 10000) in paragraph at lines 770--771
\T1/Merriwthr-OsF/m/up/7.5 (+20) Gene Ther-apy for Hemophilia. Cur-rent
Gene Th
er-apy
 []


Underfull \hbox (badness 2469) in paragraph at lines 776--777
[]\T1/Merriwthr-OsF/m/up/7.5 (+20) Goodeve AC. Hemophilia B: molec-u-lar
patho-
gen-e-sis and
 []


Underfull \hbox (badness 1028) in paragraph at lines 780--781
\T1/Merriwthr-OsF/m/up/7.5 (+20) et al. Net-work medicine frame-work for
iden-t
i-fy-ing drug-
 []


Underfull \hbox (badness 10000) in paragraph at lines 786--787
[]\T1/Merriwthr-OsF/m/up/7.5 (+20) Coppen EM, Roos RAC. Cur-rent Phar-ma-
co-log
-i-cal Ap-
 []


Underfull \hbox (badness 2418) in paragraph at lines 806--807
\T1/Merriwthr-OsF/m/up/7.5 (+20) ease: a mul-ti-cen-ter placebo-
controlled stud
y. Neu-rol-ogy
 []


Underfull \vbox (badness 10000) has occurred while \output is active []


Underfull \hbox (badness 10000) in paragraph at lines 818--819
[]\T1/Merriwthr-OsF/m/up/7.5 (+20) Molecular Data Provider Team.
[][]$\T1/lmtt/
m/n/7.5 https : / / github .
 []


Underfull \hbox (badness 10000) in paragraph at lines 818--819
\T1/lmtt/m/n/7.5 com / NCATSTranslator / Translator-[]All / wiki /
 []

[14]
enddocument/afterlastpage: lastpage setting LastPage.
(./main.aux)

LaTeX Font Warning: Size substitutions with differences
```

```
(Font)              up to 4.3pt have occurred.


LaTeX Font Warning: Some font shapes were not available, defaults
substituted.

Package rerunfilecheck Info: File `main.out' has not changed.
(rerunfilecheck)             Checksum:
8A2C5834E6B481AA74378840C8B338C9;5829.
 )
Here is how much of TeX's memory you used:
 36099 strings out of 476024
 741670 string characters out of 5794017
 1915382 words of memory out of 5000000
 54847 multiletter control sequences out of 15000+600000
 2644945 words of font info for 918 fonts, out of 8000000 for 9000
 1141 hyphenation exceptions out of 8191
 123i,16n,131p,3640b,1176s stack positions out of
10000i,1000n,20000p,200000b,200000s
{c:/TeXLive/2022/texmf-dist/fonts/enc/dvips/cm-super/cm-super-
ts1.enc}{c:/TeX
Live/2022/texmf-
dist/fonts/enc/dvips/merriweather/merriwthr_posqbl.enc}{c:/TeXL
ive/2022/texmf-dist/fonts/enc/dvips/lm/lm-ec.enc}{c:/TeXLive/2022/texmf-
dist/fo
nts/enc/dvips/merriweather/merriwthr_owzwzj.enc}<c:/TeXLive/2022/texmf-
dist/fon
ts/type1/sorkin/merriweather/Merriwthr-Bold.pfb><c:/TeXLive/2022/texmf-
dist/fon
ts/type1/sorkin/merriweather/Merriwthr-
BoldItalic.pfb><c:/TeXLive/2022/texmf-di
st/fonts/type1/sorkin/merriweather/Merriwthr-
Italic.pfb><c:/TeXLive/2022/texmf-
dist/fonts/type1/sorkin/merriweather/Merriwthr-
Regular.pfb><c:/TeXLive/2022/tex
mf-
dist/fonts/type1/public/amsfonts/cmextra/cmex8.pfb><c:/TeXLive/2022/texmf
-di
st/fonts/type1/public/amsfonts/cm/cmsy5.pfb><c:/TeXLive/2022/texmf-
dist/fonts/t
ype1/public/amsfonts/cm/cmsy6.pfb><c:/TeXLive/2022/texmf-
dist/fonts/type1/publi
c/amsfonts/cm/cmsy7.pfb><c:/TeXLive/2022/texmf-
dist/fonts/type1/public/amsfonts
/euler/euex7.pfb><c:/TeXLive/2022/texmf-
dist/fonts/type1/public/amsfonts/euler/
euex8.pfb><c:/TeXLive/2022/texmf-
dist/fonts/type1/public/amsfonts/euler/eurm5.p
fb><c:/TeXLive/2022/texmf-
dist/fonts/type1/public/amsfonts/euler/eurm7.pfb><c:/
TeXLive/2022/texmf-
dist/fonts/type1/public/amsfonts/euler/eusm5.pfb><c:/TeXLive
/2022/texmf-
dist/fonts/type1/public/amsfonts/euler/eusm7.pfb><c:/TeXLive/2022/t
```

exmf-dist/fonts/type1/public/lm/lmtt8.pfb><c:/TeXLive/2022/texmf-
dist/fonts/typ
e1/public/amsfonts/symbols/msbm7.pfb><c:/TeXLive/2022/texmf-
dist/fonts/type1/pu
blic/cm-super/sfrm0700.pfb>
Output written on main.pdf (14 pages, 501477 bytes).
PDF statistics:
 674 PDF objects out of 1000 (max. 8388607)
 612 compressed objects within 7 object streams
 177 named destinations out of 1000 (max. 500000)
 318759 words of extra memory for PDF output out of 319454 (max.
10000000)

LaTeX - Supplementary Material File
Click here to access/download;LaTeX - Main Document (TeX file);SI_Supplementary Material.tex

```
This is pdfTeX, Version 3.141592653-2.6-1.40.24 (TeX Live 2022)
(preloaded format=pdflatex 2023.3.8)  3 JUL 2023 03:44
entering extended mode
 restricted \write18 enabled.
 %&-line parsing enabled.
**"si_supplementary material.tex"
(./SI_Supplementary Material.tex
LaTeX2e <2022-11-01> patch level 1
L3 programming layer <2023-02-22> (c:/TeXLive/2022/texmf-
dist/tex/latex/base/ar
ticle.cls
Document Class: article 2022/07/02 v1.4n Standard LaTeX document class
(c:/TeXLive/2022/texmf-dist/tex/latex/base/size10.clo
File: size10.clo 2022/07/02 v1.4n Standard LaTeX file (size option)
)
\c@part=\count185
\c@section=\count186
\c@subsection=\count187
\c@subsubsection=\count188
\c@paragraph=\count189
\c@subparagraph=\count190
\c@figure=\count191
\c@table=\count192
\abovecaptionskip=\skip48
\belowcaptionskip=\skip49
\bibindent=\dimen140
) (c:/TeXLive/2022/texmf-dist/tex/latex/geometry/geometry.sty
Package: geometry 2020/01/02 v5.9 Page Geometry
(c:/TeXLive/2022/texmf-dist/tex/latex/graphics/keyval.sty
Package: keyval 2022/05/29 v1.15 key=value parser (DPC)
\KV@toks@=\toks16
) (c:/TeXLive/2022/texmf-dist/tex/generic/iftex/ifvtex.sty
Package: ifvtex 2019/10/25 v1.7 ifvtex legacy package. Use iftex instead.
(c:/TeXLive/2022/texmf-dist/tex/generic/iftex/iftex.sty
Package: iftex 2022/02/03 v1.0f TeX engine tests
))
\Gm@cnth=\count193
\Gm@cntv=\count194
\c@Gm@tempcnt=\count195
\Gm@bindingoffset=\dimen141
\Gm@wd@mp=\dimen142
\Gm@odd@mp=\dimen143
\Gm@even@mp=\dimen144
\Gm@layoutwidth=\dimen145
\Gm@layoutheight=\dimen146
\Gm@layouthoffset=\dimen147
\Gm@layoutvoffset=\dimen148
\Gm@dimlist=\toks17
) (c:/TeXLive/2022/texmf-dist/tex/latex/amsmath/amsmath.sty
Package: amsmath 2022/04/08 v2.17n AMS math features
\@mathmargin=\skip50
For additional information on amsmath, use the `?' option.
(c:/TeXLive/2022/texmf-dist/tex/latex/amsmath/amstext.sty
Package: amstext 2021/08/26 v2.01 AMS text
```

```
(c:/TeXLive/2022/texmf-dist/tex/latex/amsmath/amsgen.sty
File: amsgen.sty 1999/11/30 v2.0 generic functions
\@emptytoks=\toks18
\ex@=\dimen149
)) (c:/TeXLive/2022/texmf-dist/tex/latex/amsmath/amsbsy.sty
Package: amsbsy 1999/11/29 v1.2d Bold Symbols
\pmbraise@=\dimen150
) (c:/TeXLive/2022/texmf-dist/tex/latex/amsmath/amsopn.sty
Package: amsopn 2022/04/08 v2.04 operator names
)
\inf@bad=\count196
LaTeX Info: Redefining \frac on input line 234.
\uproot@=\count197
\leftroot@=\count198
LaTeX Info: Redefining \overline on input line 399.
LaTeX Info: Redefining \colon on input line 410.
\classnum@=\count199
\DOTSCASE@=\count266
LaTeX Info: Redefining \ldots on input line 496.
LaTeX Info: Redefining \dots on input line 499.
LaTeX Info: Redefining \cdots on input line 620.
\Mathstrutbox@=\box51
\strutbox@=\box52
LaTeX Info: Redefining \big on input line 722.
LaTeX Info: Redefining \Big on input line 723.
LaTeX Info: Redefining \bigg on input line 724.
LaTeX Info: Redefining \Bigg on input line 725.
\big@size=\dimen151
LaTeX Font Info:    Redeclaring font encoding OML on input line 743.
LaTeX Font Info:    Redeclaring font encoding OMS on input line 744.
\macc@depth=\count267
LaTeX Info: Redefining \bmod on input line 905.
LaTeX Info: Redefining \pmod on input line 910.
LaTeX Info: Redefining \smash on input line 940.
LaTeX Info: Redefining \relbar on input line 970.
LaTeX Info: Redefining \Relbar on input line 971.
\c@MaxMatrixCols=\count268
\dotsspace@=\muskip16
\c@parentequation=\count269
\dspbrk@lvl=\count270
\tag@help=\toks19
\row@=\count271
\column@=\count272
\maxfields@=\count273
\andhelp@=\toks20
\eqnshift@=\dimen152
\alignsep@=\dimen153
\tagshift@=\dimen154
\tagwidth@=\dimen155
\totwidth@=\dimen156
\lineht@=\dimen157
\@envbody=\toks21
\multlinegap=\skip51
\multlinetaggap=\skip52
```

```
\mathdisplay@stack=\toks22
LaTeX Info: Redefining \[ on input line 2953.
LaTeX Info: Redefining \] on input line 2954.
) (c:/TeXLive/2022/texmf-dist/tex/latex/graphics/graphicx.sty
Package: graphicx 2021/09/16 v1.2d Enhanced LaTeX Graphics (DPC,SPQR)
(c:/TeXLive/2022/texmf-dist/tex/latex/graphics/graphics.sty
Package: graphics 2022/03/10 v1.4e Standard LaTeX Graphics (DPC,SPQR)
(c:/TeXLive/2022/texmf-dist/tex/latex/graphics/trig.sty
Package: trig 2021/08/11 v1.11 sin cos tan (DPC)
) (c:/TeXLive/2022/texmf-dist/tex/latex/graphics-cfg/graphics.cfg
File: graphics.cfg 2016/06/04 v1.11 sample graphics configuration
)
Package graphics Info: Driver file: pdftex.def on input line 107.
(c:/TeXLive/2022/texmf-dist/tex/latex/graphics-def/pdftex.def
File: pdftex.def 2022/09/22 v1.2b Graphics/color driver for pdftex
))
\Gin@req@height=\dimen158
\Gin@req@width=\dimen159
) (c:/TeXLive/2022/texmf-dist/tex/latex/hyperref/hyperref.sty
Package: hyperref 2023-02-07 v7.00v Hypertext links for LaTeX
(c:/TeXLive/2022/texmf-dist/tex/generic/ltxcmds/ltxcmds.sty
Package: ltxcmds 2020-05-10 v1.25 LaTeX kernel commands for general use
(HO)
) (c:/TeXLive/2022/texmf-dist/tex/generic/pdftexcmds/pdftexcmds.sty
Package: pdftexcmds 2020-06-27 v0.33 Utility functions of pdfTeX for
LuaTeX (HO
)
(c:/TeXLive/2022/texmf-dist/tex/generic/infwarerr/infwarerr.sty
Package: infwarerr 2019/12/03 v1.5 Providing info/warning/error messages
(HO)
)
Package pdftexcmds Info: \pdf@primitive is available.
Package pdftexcmds Info: \pdf@ifprimitive is available.
Package pdftexcmds Info: \pdfdraftmode found.
) (c:/TeXLive/2022/texmf-dist/tex/latex/kvsetkeys/kvsetkeys.sty
Package: kvsetkeys 2022-10-05 v1.19 Key value parser (HO)
) (c:/TeXLive/2022/texmf-dist/tex/generic/kvdefinekeys/kvdefinekeys.sty
Package: kvdefinekeys 2019-12-19 v1.6 Define keys (HO)
) (c:/TeXLive/2022/texmf-dist/tex/generic/pdfescape/pdfescape.sty
Package: pdfescape 2019/12/09 v1.15 Implements pdfTeX's escape features
(HO)
) (c:/TeXLive/2022/texmf-dist/tex/latex/hycolor/hycolor.sty
Package: hycolor 2020-01-27 v1.10 Color options for hyperref/bookmark
(HO)
) (c:/TeXLive/2022/texmf-dist/tex/latex/letltxmacro/letltxmacro.sty
Package: letltxmacro 2019/12/03 v1.6 Let assignment for LaTeX macros (HO)
) (c:/TeXLive/2022/texmf-dist/tex/latex/auxhook/auxhook.sty
Package: auxhook 2019-12-17 v1.6 Hooks for auxiliary files (HO)
) (c:/TeXLive/2022/texmf-dist/tex/latex/hyperref/nameref.sty
Package: nameref 2022-05-17 v2.50 Cross-referencing by name of section
(c:/TeXLive/2022/texmf-dist/tex/latex/refcount/refcount.sty
Package: refcount 2019/12/15 v3.6 Data extraction from label references
(HO)
```

```
) (c:/TeXLive/2022/texmf-
dist/tex/generic/gettitlestring/gettitlestring.sty
Package: gettitlestring 2019/12/15 v1.6 Cleanup title references (HO)
(c:/TeXLive/2022/texmf-dist/tex/latex/kvoptions/kvoptions.sty
Package: kvoptions 2022-06-15 v3.15 Key value format for package options
(HO)
))
\c@section@level=\count274
)
\@linkdim=\dimen160
\Hy@linkcounter=\count275
\Hy@pagecounter=\count276
(c:/TeXLive/2022/texmf-dist/tex/latex/hyperref/pd1enc.def
File: pd1enc.def 2023-02-07 v7.00v Hyperref: PDFDocEncoding definition
(HO)
Now handling font encoding PD1 ...
... no UTF-8 mapping file for font encoding PD1
) (c:/TeXLive/2022/texmf-dist/tex/generic/intcalc/intcalc.sty
Package: intcalc 2019/12/15 v1.3 Expandable calculations with integers
(HO)
) (c:/TeXLive/2022/texmf-dist/tex/generic/etexcmds/etexcmds.sty
Package: etexcmds 2019/12/15 v1.7 Avoid name clashes with e-TeX commands
(HO)
)
\Hy@SavedSpaceFactor=\count277
(c:/TeXLive/2022/texmf-dist/tex/latex/hyperref/puenc.def
File: puenc.def 2023-02-07 v7.00v Hyperref: PDF Unicode definition (HO)
Now handling font encoding PU ...
... no UTF-8 mapping file for font encoding PU
)
Package hyperref Info: Option `colorlinks' set `true' on input line 4060.
Package hyperref Info: Hyper figures OFF on input line 4177.
Package hyperref Info: Link nesting OFF on input line 4182.
Package hyperref Info: Hyper index ON on input line 4185.
Package hyperref Info: Plain pages OFF on input line 4192.
Package hyperref Info: Backreferencing OFF on input line 4197.
Package hyperref Info: Implicit mode ON; LaTeX internals redefined.
Package hyperref Info: Bookmarks ON on input line 4425.
\c@Hy@tempcnt=\count278
(c:/TeXLive/2022/texmf-dist/tex/latex/url/url.sty
\Urlmuskip=\muskip17
Package: url 2013/09/16  ver 3.4  Verb mode for urls, etc.
)
LaTeX Info: Redefining \url on input line 4763.
\XeTeXLinkMargin=\dimen161
(c:/TeXLive/2022/texmf-dist/tex/generic/bitset/bitset.sty
Package: bitset 2019/12/09 v1.3 Handle bit-vector datatype (HO)
(c:/TeXLive/2022/texmf-dist/tex/generic/bigintcalc/bigintcalc.sty
Package: bigintcalc 2019/12/15 v1.5 Expandable calculations on big
integers (HO
)
))
\Fld@menulength=\count279
\Field@Width=\dimen162
```

```
\Fld@charsize=\dimen163
Package hyperref Info: Hyper figures OFF on input line 6042.
Package hyperref Info: Link nesting OFF on input line 6047.
Package hyperref Info: Hyper index ON on input line 6050.
Package hyperref Info: backreferencing OFF on input line 6057.
Package hyperref Info: Link coloring ON on input line 6060.
Package hyperref Info: Link coloring with OCG OFF on input line 6067.
Package hyperref Info: PDF/A mode OFF on input line 6072.
(c:/TeXLive/2022/texmf-dist/tex/latex/base/atbegshi-ltx.sty
Package: atbegshi-ltx 2021/01/10 v1.0c Emulation of the original atbegshi
package with kernel methods
)
\Hy@abspage=\count280
\c@Item=\count281
\c@Hfootnote=\count282
)
Package hyperref Info: Driver (autodetected): hpdftex.
(c:/TeXLive/2022/texmf-dist/tex/latex/hyperref/hpdftex.def
File: hpdftex.def 2023-02-07 v7.00v Hyperref driver for pdfTeX
(c:/TeXLive/2022/texmf-dist/tex/latex/base/atveryend-ltx.sty
Package: atveryend-ltx 2020/08/19 v1.0a Emulation of the original
atveryend pac
kage
with kernel methods
)
\Fld@listcount=\count283
\c@bookmark@seq@number=\count284
(c:/TeXLive/2022/texmf-dist/tex/latex/rerunfilecheck/rerunfilecheck.sty
Package: rerunfilecheck 2022-07-10 v1.10 Rerun checks for auxiliary files
(HO)
(c:/TeXLive/2022/texmf-dist/tex/generic/uniquecounter/uniquecounter.sty
Package: uniquecounter 2019/12/15 v1.4 Provide unlimited unique counter
(HO)
)
Package uniquecounter Info: New unique counter `rerunfilecheck' on input
line 2
85.
)
\Hy@SectionHShift=\skip53
) (c:/TeXLive/2022/texmf-dist/tex/latex/preprint/authblk.sty
Package: authblk 2001/02/27 1.3 (PWD)
\affilsep=\skip54
\@affilsep=\skip55
\c@Maxaffil=\count285
\c@authors=\count286
\c@affil=\count287
) (c:/TeXLive/2022/texmf-dist/tex/latex/titlesec/titlesec.sty
Package: titlesec 2021/07/05 v2.14 Sectioning titles
\ttl@box=\box53
\beforetitleunit=\skip56
\aftertitleunit=\skip57
\ttl@plus=\dimen164
\ttl@minus=\dimen165
\ttl@toksa=\toks23
```

```
\titlewidth=\dimen166
\titlewidthlast=\dimen167
\titlewidthfirst=\dimen168
) (c:/TeXLive/2022/texmf-dist/tex/latex/natbib/natbib.sty
Package: natbib 2010/09/13 8.31b (PWD, AO)
\bibhang=\skip58
\bibsep=\skip59
LaTeX Info: Redefining \cite on input line 694.
\c@NAT@ctr=\count288
) (c:/TeXLive/2022/texmf-dist/tex/latex/booktabs/booktabs.sty
Package: booktabs 2020/01/12 v1.61803398 Publication quality tables
\heavyrulewidth=\dimen169
\lightrulewidth=\dimen170
\cmidrulewidth=\dimen171
\belowrulesep=\dimen172
\belowbottomsep=\dimen173
\aboverulesep=\dimen174
\abovetopsep=\dimen175
\cmidrulesep=\dimen176
\cmidrulekern=\dimen177
\defaultaddspace=\dimen178
\@cmidla=\count289
\@cmidlb=\count290
\@aboverulesep=\dimen179
\@belowrulesep=\dimen180
\@thisruleclass=\count291
\@lastruleclass=\count292
\@thisrulewidth=\dimen181
) (c:/TeXLive/2022/texmf-dist/tex/latex/threeparttable/threeparttable.sty
Package: threeparttable 2003/06/13  v 3.0
\@tempboxb=\box54
) (c:/TeXLive/2022/texmf-dist/tex/latex/l3backend/l3backend-pdftex.def
File: l3backend-pdftex.def 2023-01-16 L3 backend support: PDF output
(pdfTeX)
\l__color_backend_stack_int=\count293
\l__pdf_internal_box=\box55
) (./SI_Supplementary Material.aux)
\openout1 = `"SI_Supplementary Material.aux"'.

LaTeX Font Info:    Checking defaults for OML/cmm/m/it on input line 39.
LaTeX Font Info:    ... okay on input line 39.
LaTeX Font Info:    Checking defaults for OMS/cmsy/m/n on input line 39.
LaTeX Font Info:    ... okay on input line 39.
LaTeX Font Info:    Checking defaults for OT1/cmr/m/n on input line 39.
LaTeX Font Info:    ... okay on input line 39.
LaTeX Font Info:    Checking defaults for T1/cmr/m/n on input line 39.
LaTeX Font Info:    ... okay on input line 39.
LaTeX Font Info:    Checking defaults for TS1/cmr/m/n on input line 39.
LaTeX Font Info:    ... okay on input line 39.
LaTeX Font Info:    Checking defaults for OMX/cmex/m/n on input line 39.
LaTeX Font Info:    ... okay on input line 39.
LaTeX Font Info:    Checking defaults for U/cmr/m/n on input line 39.
LaTeX Font Info:    ... okay on input line 39.
LaTeX Font Info:    Checking defaults for PD1/pdf/m/n on input line 39.
```

```
LaTeX Font Info:    ... okay on input line 39.
LaTeX Font Info:    Checking defaults for PU/pdf/m/n on input line 39.
LaTeX Font Info:    ... okay on input line 39.
*geometry* driver: auto-detecting
*geometry* detected driver: pdftex
*geometry* verbose mode - [ preamble ] result:
* driver: pdftex
* paper: <default>
* layout: <same size as paper>
* layoutoffset:(h,v)=(0.0pt,0.0pt)
* modes: 
* h-part:(L,W,R)=(56.9055pt, 500.484pt, 56.9055pt)
* v-part:(T,H,B)=(28.45274pt, 709.61174pt, 56.9055pt)
* \paperwidth=614.295pt
* \paperheight=794.96999pt
* \textwidth=500.484pt
* \textheight=709.61174pt
* \oddsidemargin=-15.36449pt
* \evensidemargin=-15.36449pt
* \topmargin=-80.81725pt
* \headheight=12.0pt
* \headsep=25.0pt
* \topskip=10.0pt
* \footskip=30.0pt
* \marginparwidth=65.0pt
* \marginparsep=11.0pt
* \columnsep=10.0pt
* \skip\footins=9.0pt plus 4.0pt minus 2.0pt
* \hoffset=0.0pt
* \voffset=0.0pt
* \mag=1000
* \@twocolumnfalse
* \@twosidefalse
* \@mparswitchfalse
* \@reversemarginfalse
* (1in=72.27pt=25.4mm, 1cm=28.453pt)

(c:/TeXLive/2022/texmf-dist/tex/context/base/mkii/supp-pdf.mkii
[Loading MPS to PDF converter (version 2006.09.02).]
\scratchcounter=\count294
\scratchdimen=\dimen182
\scratchbox=\box56
\nofMPsegments=\count295
\nofMParguments=\count296
\everyMPshowfont=\toks24
\MPscratchCnt=\count297
\MPscratchDim=\dimen183
\MPnumerator=\count298
\makeMPintoPDFobject=\count299
\everyMPtoPDFconversion=\toks25
) (c:/TeXLive/2022/texmf-dist/tex/latex/epstopdf-pkg/epstopdf-base.sty
Package: epstopdf-base 2020-01-24 v2.11 Base part for package epstopdf
Package epstopdf-base Info: Redefining graphics rule for `.eps' on input
line 4
```

```
85.
(c:/TeXLive/2022/texmf-dist/tex/latex/latexconfig/epstopdf-sys.cfg
File: epstopdf-sys.cfg 2010/07/13 v1.3 Configuration of (r)epstopdf for
TeX Liv
e
)) (c:/TeXLive/2022/texmf-dist/tex/latex/graphics/color.sty
Package: color 2022/01/06 v1.3d Standard LaTeX Color (DPC)
(c:/TeXLive/2022/texmf-dist/tex/latex/graphics-cfg/color.cfg
File: color.cfg 2016/01/02 v1.6 sample color configuration
)
Package color Info: Driver file: pdftex.def on input line 149.
(c:/TeXLive/2022/texmf-dist/tex/latex/graphics/mathcolor.ltx))
Package hyperref Info: Link coloring ON on input line 39.
(./SI_Supplementary Material.out) (./SI_Supplementary Material.out)
\@outlinefile=\write3
\openout3 = `"SI_Supplementary Material.out"'.


Package natbib Warning: Citation `Wood_E_C_2022' on page 1 undefined on
input l
ine 44.


Package natbib Warning: Citation `Kilicoglu_Halil_2012' on page 1
undefined on
input line 47.


Package natbib Warning: Citation `Cong_Qing_2018' on page 1 undefined on
input
line 47.


Package natbib Warning: Citation `Cilibrasi_R_L_2007' on page 1 undefined
on in
put line 50.


Package natbib Warning: Citation `unni2022biolink' on page 1 undefined on
input
 line 52.


Package natbib Warning: Citation `Xin_Jiwen_2018' on page 1 undefined on
input
line 59.

[1

{c:/TeXLive/2022/texmf-var/fonts/map/pdftex/updmap/pdftex.map}]

Package natbib Warning: Citation `Banda_Juan_M_2016' on page 2 undefined
on inp
ut line 70.
```

```
Package natbib Warning: Citation `Degtyarenko_Kirill_2008' on page 2
undefined
on input line 71.


Package natbib Warning: Citation `Gaulton_Anna_2012' on page 2 undefined
on inp
ut line 72.


Package natbib Warning: Citation `Ursu_Oleg_2016' on page 2 undefined on
input
line 73.


Package natbib Warning: Citation `Peryea_Tyler_2020' on page 2 undefined
on inp
ut line 75.


Package natbib Warning: Citation `Thorn_Caroline_F_2013' on page 2
undefined on
 input line 77.


Package natbib Warning: Citation `Kim_Sunghwan_2016' on page 2 undefined
on inp
ut line 78.


Package natbib Warning: Citation `Kuhn_Michael_2015' on page 2 undefined
on inp
ut line 79.


Underfull \hbox (badness 10000) in paragraph at lines 88--89
[][][]\OT1/cmr/m/n/10 https://www.fda.gov/industry/fda-data-standards-
advisory-
board/fdas-global-substance-registration-
 []

LaTeX Font Info:    Font shape `OT1/cmtt/bx/n' in size <12> not available
(Font)              Font shape `OT1/cmtt/m/n' tried instead on input line
94.

Package natbib Warning: Citation `Hamilton_William_L_2017' on page 2
undefined
on input line 97.


Package natbib Warning: Citation `Gu_Yu_2022' on page 2 undefined on
input line
```

```
 97.

LaTeX Font Info:    Trying to load font information for OMS+cmr on input
line 9
7.
(c:/TeXLive/2022/texmf-dist/tex/latex/base/omscmr.fd
File: omscmr.fd 2022/07/10 v2.5l Standard LaTeX font definitions
)
LaTeX Font Info:    Font shape `OMS/cmr/m/n' in size <10> not available
(Font)              Font shape `OMS/cmsy/m/n' tried instead on input line
97.

Package natbib Warning: Citation `Zhao_Kangzhi_2020' on page 2 undefined
on inp
ut line 100.


Package natbib Warning: Citation `Lawrence_Page_1999' on page 2 undefined
on in
put line 100.


Package natbib Warning: Citation `Glorot_Xavier_2010' on page 2 undefined
on in
put line 100.


Package natbib Warning: Citation `Diederik_P_Kingma_2015' on page 2
undefined o
n input line 100.

[2]

LaTeX Warning: `h' float specifier changed to `ht'.


Package natbib Warning: Citation `Lin_Xi_Victoria_2018' on page 3
undefined on
input line 135.

[3]

! LaTeX Error: File `./Figures/suppl_figure1' not found.

See the LaTeX manual or LaTeX Companion for explanation.
Type  H <return>  for immediate help.
 ...

l.144 ...hics[height=9cm]{./Figures/suppl_figure1}

I could not locate the file with any of these extensions:
.pdf,.png,.jpg,.mps,.jpeg,.jbig2,.jb2,.PDF,.PNG,.JPG,.JPEG,.JBIG2,.JB2,.e
ps
Try typing  <return>  to proceed.
```

```
If that doesn't work, type  X <return>  to quit.

[4]
No file "SI_Supplementary Material".bbl.

Package natbib Warning: There were undefined citations.

(./SI_Supplementary Material.aux)
Package rerunfilecheck Info: File `"SI_Supplementary Material".out' has
not cha
nged.
(rerunfilecheck)             Checksum:
022117FEC253165FC3AC7ECE589C936C;550.
 )
Here is how much of TeX's memory you used:
 10367 strings out of 476024
 161816 string characters out of 5794017
 1868382 words of memory out of 5000000
 30647 multiletter control sequences out of 15000+600000
 519811 words of font info for 59 fonts, out of 8000000 for 9000
 1141 hyphenation exceptions out of 8191
 75i,15n,76p,1847b,441s stack positions out of
10000i,1000n,20000p,200000b,200000s
{c:/TeXLive/2022/texmf-dist/fonts/enc/dvips/cm-super/cm-super-
ts1.enc}<c:/TeX
Live/2022/texmf-
dist/fonts/type1/public/amsfonts/cm/cmbx10.pfb><c:/TeXLive/2022
/texmf-
dist/fonts/type1/public/amsfonts/cm/cmbx12.pfb><c:/TeXLive/2022/texmf-di
st/fonts/type1/public/amsfonts/cm/cmmi10.pfb><c:/TeXLive/2022/texmf-
dist/fonts/
type1/public/amsfonts/cm/cmmi7.pfb><c:/TeXLive/2022/texmf-
dist/fonts/type1/publ
ic/amsfonts/cm/cmr10.pfb><c:/TeXLive/2022/texmf-
dist/fonts/type1/public/amsfont
s/cm/cmr12.pfb><c:/TeXLive/2022/texmf-
dist/fonts/type1/public/amsfonts/cm/cmr6.
pfb><c:/TeXLive/2022/texmf-
dist/fonts/type1/public/amsfonts/cm/cmr7.pfb><c:/TeX
Live/2022/texmf-
dist/fonts/type1/public/amsfonts/cm/cmr8.pfb><c:/TeXLive/2022/t
exmf-
dist/fonts/type1/public/amsfonts/cm/cmsy10.pfb><c:/TeXLive/2022/texmf-
dist
/fonts/type1/public/amsfonts/cm/cmti10.pfb><c:/TeXLive/2022/texmf-
dist/fonts/ty
pe1/public/amsfonts/cm/cmtt10.pfb><c:/TeXLive/2022/texmf-
dist/fonts/type1/publi
c/amsfonts/cm/cmtt12.pfb><c:/TeXLive/2022/texmf-
dist/fonts/type1/public/amsfont
s/cm/cmtt8.pfb><c:/TeXLive/2022/texmf-dist/fonts/type1/public/cm-
super/sfrm1000
.pfb>
```

```
Output written on "SI_Supplementary Material.pdf" (4 pages, 212399
bytes).
PDF statistics:
 154 PDF objects out of 1000 (max. 8388607)
 116 compressed objects within 2 object streams
 22 named destinations out of 1000 (max. 500000)
 17 words of extra memory for PDF output out of 10000 (max. 10000000)
```

Manuscript

Click here to access/download;Manuscript;KGML_xDTD_GigaScience_revis

OXFORD

(GIGA)^n SCIENCE

GigaScience, 2017, 1–17

doi: xx.xxxx/xxxx
Manuscript in Preparation
research

RESEARCH

KGML-xDTD: A Knowledge Graph-based Machine Learning Framework for Drug Treatment Prediction and Mechanism Description

Chunyu Ma[1,*], Zhihan Zhou[2], Han Liu[2] and David Koslicki[1,3,4,*]

[1]Huck Institutes of Life Sciences, Pennsylvania State University, State College, PA 16801, USA and [2]Department of Computer Science, Northwestern University, Evanston, IL 60208, USA and [3]Department of Computer Science and Engineering, Pennsylvania State University, State College, PA 16801, USA and [4]Department of Biology, Pennsylvania State University, State College, PA 16801, USA

[*]Correspondence address. Chunyu Ma, E-mail: cqm5886@psu.edu; David Koslicki, E-mail: dmk333@psu.edu

Abstract

Background: Computational drug repurposing is a cost- and time-efficient approach that aims to identify new therapeutic targets or diseases (indications) of existing drugs/compounds. It is especially critical for emerging and/or orphan diseases due to its cheaper investment and shorter research cycle compared with traditional wet-lab drug discovery approaches. However, the underlying mechanisms of action (MOAs) between repurposed drugs and their target diseases remain largely unknown, which is still a main obstacle for computational drug repurposing methods to be widely adopted in clinical settings.
Results: In this work, we propose `KGML-xDTD`: a **K**nowledge **G**raph-based **M**achine **L**earning framework for e**x**plainably predicting **D**rugs **T**reating **D**iseases. It is a two-module framework that not only predicts the treatment probabilities between drugs/compounds and diseases but also biologically explains them via knowledge graph (KG) path-based, testable mechanisms of action (MOAs). We leverage knowledge-and-publication based information to extract biologically meaningful "demonstration paths" as the intermediate guidance in the Graph-based Reinforcement Learning (GRL) path-finding process. Comprehensive experiments and case study analyses show that the proposed framework can achieve state-of-the-art performance in both predictions of drug repurposing and recapitulation of human-curated drug MOA paths.
Conclusions: `KGML-xDTD` is the first model framework that can offer KG-path explanations for drug repurposing predictions by leveraging the combination of prediction outcomes and existing biological knowledge and publications. We believe it can effectively reduce "black-box" concerns and increase prediction confidence for drug repurposing based on predicted path-based explanations, and further accelerate the process of drug discovery for emerging diseases.

Key words: Drug Repurposing, Reinforcement Learning, Biomedical Knowledge Graph

Introduction

Traditional drug development is a time-consuming process (from initial chemical identification to clinical trials and finally to FDA approval) that takes around 10-15 years and also comes along with billions-of-dollars investments and high failure rates [1]. Considering the rapid pace of novel disease evolution, it is urgent to find a more efficient and economical drug discovery method. Fortunately, it has been observed that a single drug can often be effective in treating multiple diseases. For example, thalidomide was originally used as an anti-anxiety medication [2], and was later found to have the anti-cancer potential for the treatment of cancers [3, 4]. Hence, drug repurposing, also known as the identification of new uses for existing drugs/compounds, might bring us hope to address this urgent need with the advantage of a shorter research cycle, lower development cost, and more preexisting safety tests.

Compiled on: July 3, 2023.
Draft manuscript prepared by the author.

Existing drug repurposing approaches can roughly be categorized into experimental-based approaches (e.g., binding affinity assays [5], phenotypic screening [6]), clinical-based approaches (e.g., off-label drug use analysis [7]), and computational-based approaches (e.g., chemical-structure-based [8], and GWAS-based approaches [9]). Compared with the former two approaches, the computational approaches are more cost- and time-efficient, particularly when the goal is to prioritize a large number of target drugs/compounds for follow-up experimental investigation. Among all computational drug repurposing methods, the integration of multiple biomedical data sources into a so-called **biomedical knowledge graph** (BKG) for drug discovery has become popular in recent year [10] due to the increasing availability of curated biomedical databases such as DrugBank [11], ChEMBL [12], HMDB [13] and the advancement of semantic web techniques [14]. There are three types of existing BKGs: database-based BKGs, literature-based BKGs, and mixed BKGs. The database-based BKGs (e.g., *Hetionet* [15], *BioKG* [16], *CBKH* [17]) are constructed by integrating biomedical data and their relations stored in existing biological databases. The literature-based BKGs (e.g., *GNBR* [18]) are built by leveraging Natural Language Processing (NLP) techniques to extract semantic information from a large amount of available biomedical literature and electronic health record (EHR) data, which are mostly disease-specific [19, 20, 21]. The mixed BKGs (e.g., *CKG* [22], *RTX-KG2* [23]) are generated by combining the knowledge sources from the above two methods.

Based on these BKGs, several machine learning methods have been proposed or implemented for drug repurposing prediction by treating it as a link prediction task in the BKGs. For example, **(author?)** [15] used the so-called degree-weighted path count (DWPC) to assess the prevalence of 1,206 metapaths and then classified drug-disease treatment relations by fitting these DWPC features to a logistic regression model. **(author?)** [24] proposed a novel graph neural network model I-RGCN to learn the node and relation embeddings for the Covid-19 drug repurposing task. **(author?)** [19] recently predicted the possible drugs for Covid-19 with five existing popular knowledge graph completion methods (e.g. TransE [25], RotatE [26], DistMult [27], ComplEx [28], and STELP [29]). Although some of these models have shown good performance in drug repurposing prediction on the small-scale BKGs, none have been scaled to massive BKGs with more than millions of nodes and edges and make a comprehensive comparison. More importantly, most of them lack the biological explanatory ability for their predictions, which limits their applicability in clinical research.

Currently, there are few computational models designed for drug repurposing *explanations*. A common and intuitive explanation based on a biomedical knowledge graph for drug repurposing leverages the semantic BKG-based paths between given drug-disease pairs. **(author?)** [30] applied a graph embedding model UKGE [31], which utilizes the weighted (the frequency of relation appeared in literature) relation edges in a literature-based KG *GNBR*, to identify new indications of drugs for rare diseases and then explain the results via the highest-ranking paths based on confidence scores. However, this method is only applicable in the literature-based BKGs with the weighted edge information. Most BKGs using database-based knowledge don't contain such information. **(author?)** [32] proposed GrEDeL that combines the TransE embedding method with a Long Short-Term Memory (LSTM) Recurrent Neural Network (RNN) model to predict drug-disease relation. By using the embeddings of BKG-paths as model input for predictions, they can provide path-based explanations. However, they claimed that the effectiveness of the approach relies heavily on the NLP tool SemRep, which is reported to have high false positives in named entity recognition [33]. Also, they didn't fully evaluate how biologically reasonable their predicted path-based mechanisms of action (MOAs) are.

Besides the existing methods above, we view reinforcement learning (RL) as a promising solution for drug repurposing explanation. RL models solve the decision-making problem, in which an agent learns how to take appropriate actions to maximize cumulative rewards through interactions with the environment. RL has achieved widespread success in various domains, including games, recommendation systems, healthcare, transportation, etc [34]. Graph Reinforcement Learning (GRL), first proposed around in 2017, aims to solve graph mining tasks such as link prediction [35], adversarial attacks [36], and relational reasoning [37]. Unlike its applications in other domains, one of the biggest challenges in GRL is finding an appropriate reward to guide the path searching in specific domains. To address the issue of finding biologically reasonable BKG-based paths for drug repurposing, it is crucial to incorporate biomedical domain knowledge to guide the path-finding process. **(author?)** [38] developed an RL-based model "PoLo" that utilizes the biological meta-paths identified in **(author?)** [15] via the "DWPC" method to supervise path searching for drug repurposing. However, the "PoLo" model does not scale to a massive and complex BKG (e.g., CKG and RTX-KG2) due to its dependence on the "DWPC" method that is reported to be computationally inefficient [39].

In this article, we describe `KGML-xDTD`: a **K**nowledge **G**raph-based **M**achine **L**earning framework for e**x**plainably predicting **D**rugs **T**reating **D**iseases, which contains two modules for both drug repurposing prediction and MOA explanation. We propose to amplify the ability of RL model in biologically meaningful path searching by utilizing the biologically meaningful "demonstration paths" and pre-trained drug-repurposing model probability as rewards. We incorporate this idea into the appropriate models (e.g., GraphSAGE [40], Random Forest, and ADAC RL [41] models) and then make them applicable to the explainable drug repurposing problem at massive data scale and complexity. By comparing with the existing popular drug repurposing models and evaluating the predicted paths with an expert-curated path-based drug MOA database *DrugMechDB* [42, 43], we show that the proposed model framework can achieve state-of-the-art performance in both predictions of drug repurposing and recapitulation of human-curated drug MOA paths provided by DrugMechDB. In further case studies, by comparing the model predictions with the real regulatory networks, we show that the proposed framework effectively identifies biologically reasonable BKG-based MOA paths for real-world applications.

Materials and Methods

Datasets

Customized Biomedical Knowledge Graph

To accommodate biomedical-reasonable predictions of drugs' indications and their mechanisms of action, the ideal biomedical knowledge graph should integrate biomedical knowledge from comprehensive and diverse databases and publications, as well as accurately identify and merge different identifiers representing the same biological entity into one (For example, "CHEBI:2367" and "CHEMBL455626" are two distinct identifiers separately presented in ChEBI database [44] and ChEMBL database [12] but represent the same compound "abyssinone I"). Thus, we utilize the canonicalized version of the Reasoning Tool X Knowledge Graph 2 (*RTX-KG2c*) [23], one of the largest open-source biomedical knowledge graph (BKG) that integrates knowledge from extensive human-curated and Publication-based databases, and has been widely used in the Biomedical Data Translator Project [45, 46]. Compared to other commonly used open-source BKGs mentioned above, *RTX-KG2c* is a biolink-model-based standardized [47] and regularly-updated BKG that efficiently merges biologically and semantically equivalent nodes and edges via multiple curation steps. The version 2.7.3 of *RTX-KG2c* that we use contains around 6.4M nodes and 39.3M edges with knowledge from 70 public biomedical sources, where all biological concepts (e.g., "ibuprofen") are represented as vertices and

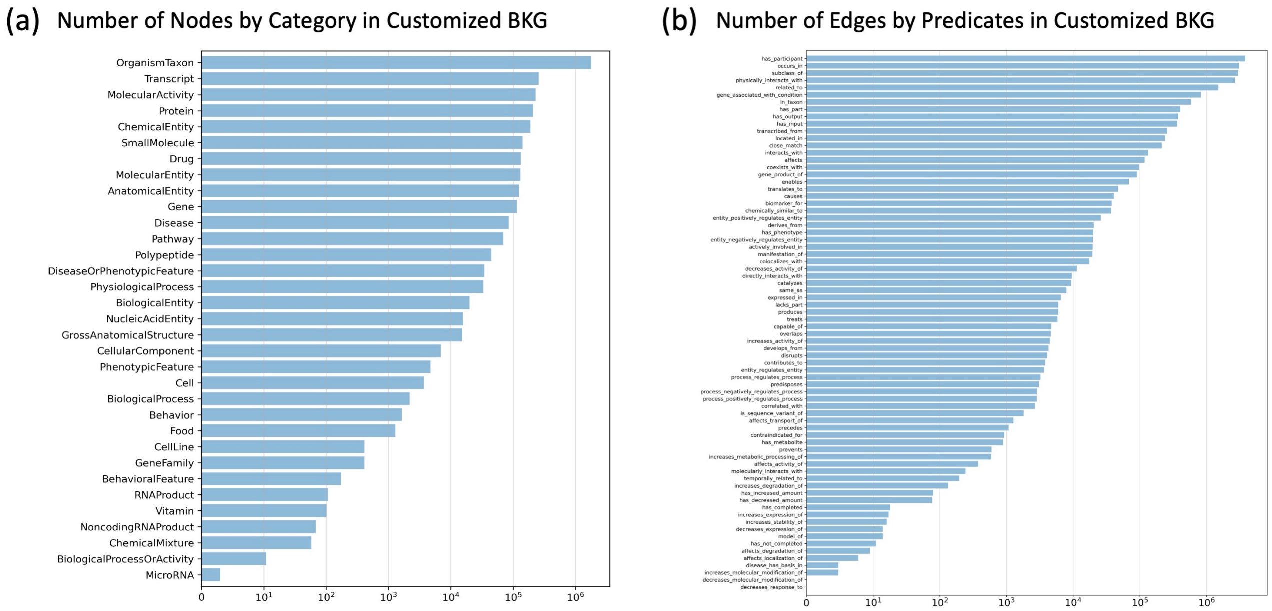

Figure 1. Number of nodes by category (a) and number of edges by predicate (b) in customized Biomedical Knowledge Graph (BKG).

all concept-predicate-concept (e.g., "ibuprofen - increases activity of - GP1BA gene") are presented as edges. For drug repurposing purposes, we customized *RTX-KG2c* with four principles (Please see more details in Supplementary Section S1): 1). excluding the nodes whose categories are irrelevant to drug repurposing explanation (e.g., "GeographicLocation" and "Device"); 2). filtering out the low-quality edges based on our criteria; 3). removing the hierarchically redundant edges; 4). excluding all drug-disease edges. After these processing steps, 3,659,165 nodes with 33 distinct categories (Figure 1 a) and 18,291,237 edges with 74 distinct types (Figure 1 b) are left in our customized biomedical knowledge graph, which is used for downstream model training.

Data Sources for Model Training

To train the `KGML-xDTD` framework for drug repurposing prediction and its MOA explanation, we utilize four high-quality and NLP-derived training datasets:

- **MyChem Data** [48] is provided by the BioThings API collection [49], which contains up-to-date annotations regarding indication and contraindication for chemicals collected from 11 reliable data resources (summarized in Supplementary Section S2). We use drug-disease pairs with the relation "indication" as true positives while those with "contraindication" as true negatives.
- **SemMedDB Data** [50] is provided by the Semantic MEDLINE Database (SemMedDB), which leverages natural language processing (NLP) techniques to extract semantic triples with "treats" and "negatively treats" relations from PubMed abstracts. We use drug-disease pairs with the relation "treats" as true positives while those with "negatively treats" as true negatives.
- **NDF-RT Data** [51] is provided by National Drug File – Reference Terminology from Veterans Health Administration (VHA) which contains FDA-approved information on drug interaction, indications, and contraindications. We use drug-disease with therapeutics label "indications" as true positives while those with "contraindications" as true negatives.
- **RepoDB Data** [52] is a standard set of successful and failed drug-disease pairs in clinical trials collected by the Blavatnik Institute at Harvard Medical School. We use drug-disease with the status "approved" as true positives while those with "terminated" as true negatives.

Table 1. Pair count of true positive (indications) and true negative (contraindications or no effect) data from four data sources after data preprocessing.

Source	True Positive (Treats)	True Negative (Not Treat)
MyChem	3,663	26,795
SemMedDB	8,255	11
NDF-RT	3,421	5,119
RepoDB	2,127	738
Shared	3,971	526
**Total**	21,437	33,189

Note that 'shared' means those pairs are from two or more data sources.

We further filter drug-disease pairs from SemMedDB Data due to publication bias and possible NLP mistakes by using both the co-occurrence frequency and the PubMed-publication-based Normalized Google Distance (NGD) [53] defined below:

$$NGD(c1, c2) = \frac{max\{log\mathcal{N}(c1), log\mathcal{N}(c2)\} - log\mathcal{N}(c1, c2)}{logN - min\{log\mathcal{N}(c1), log\mathcal{N}(c2)\}} \quad (1)$$

where $c1$ and $c2$ are two biological concepts used in the customized BKG; $\mathcal{N}(c1)$ and $\mathcal{N}(c2)$ respectively represent the total number of unique PubMed IDs associated with $c1$ and $c2$; $\mathcal{N}(c1, c2)$ is the total number of unique PubMed IDs shared between $c1$ and $c2$; N is the total number of pairs of Medical Subject Heading (MeSH) terms annotations in PubMed database. Only the SemMedDB drug-disease pairs with at least 10 supporting publications and an NGD score of 0.6 or lower are left for the downstream model training.

These datasets are pooled together and then processed by 1). mapping the raw identifiers of drugs and diseases to the identifiers used in the customized BKG; 2) removing duplicate drug-disease pairs in both the true positive set and the true negative set. Table 1 shows the drug-disease pair count from each data source after data pre-processing.

DrugMechDB

DrugMechDB [42, 43], to our best knowledge, is the first human-curated path-based database for explaining the mechanism of action (MOA) from a drug to a disease in an indication, with 3,593 MOA paths for 3,327 unique drug-disease pairs. These paths are extracted from free-text descriptions from DrugBank, Wikipedia,

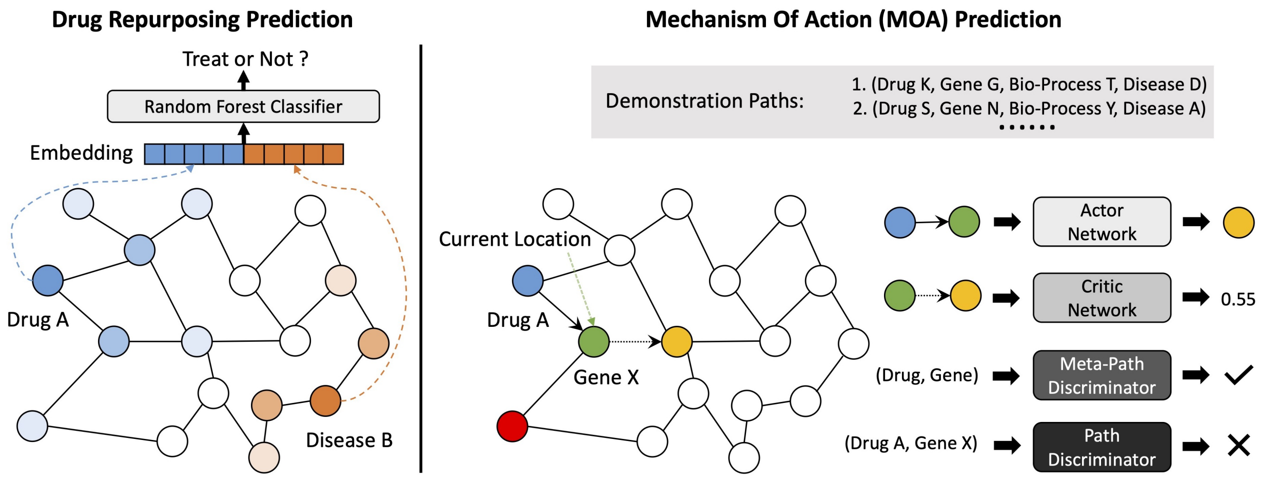

Figure 2. Illustration of entire KGML-xDTD model framework: drug repurposing prediction (DRP) module (left) and mechanism of action (MOA) prediction module (right).

and other literature sources, and then have been curated by subject matter experts and also follow the schema of Biolink model. Hence, we can match them to nodes used in the RTX-KG2 BKG via the *Node Synonymizer* function [23]. Since the maximum length of predicted MOA paths generated by the KGML-xDTD framework is fixed to 3 in this study due to memory and training time constraints, we consider those 3-hop BKG-based paths as "correct" if all four of their nodes show up in the complete DrugMechDB-based MOA paths. Thus, we find 472 unique drug-disease pairs of which each has at least one such "correct" matched path in all possible 3-hop paths between drug and disease in the customized BKG. The large reduction in evaluation paths is likely due to incompleteness of the underlying knowledge graphs, imperfect bioentity matching, and possibility of disconnected drug and disease pairs in the customized BKG. However, these paths are used for additional, external validation data only. We use the matched paths as true positive biologically meaningful paths for the evaluation of the model-predicted paths in the task of Mechanism of Action (MOA) Prediction (introduced below).

Model Framework

The model framework of KGML-xDTD consists of two modules: a drug repurposing prediction (DRP) module that combines the advantages of GraphSAGE [40] and a Random Forest model, and a Mechanism of Action (MOA) prediction module that utilizes an adversarial actor-critic reinforcement learning (RL) model. We show the overview of the entire model framework in Figure 2. The implementation details of each module in KGML-xDTD framework are presented in Supplementary Section S3.

Notations

Let $\mathcal{G} = \{\mathcal{V}, \mathcal{E}\}$ be a directed biomedical knowledge graph, where each node $v \in \mathcal{V}$ represents a biological entity (e.g., a specific drug, disease, gene, or pathway, etc.) and each edge $e \in \mathcal{E}$ represents a biomedical relationship (e.g., *interacts-with, see more in Figure 1 b*). We use $\mathcal{V}^{\text{drug}}$ to represent all the drug nodes (the nodes with the categories of "Drug" and "Small Molecule" in the customized BKG) and $\mathcal{V}^{\text{disease}}$ to represent all the disease nodes (the nodes with the categories of "Disease", "PhenotypicFeature", "BehavioralFeature" and "DiseaseOrPhenotypicFeature" in the customized BKG). For each notation, we use bold formatting to represent its embedding (e.g., $\boldsymbol{v}$ represents the embedding of v).

Drug Repurposing Prediction (DRP) Module

Drug repurposing aims to identify new indications of existing drugs/compounds. We solve it as a link prediction problem on the graph $\mathcal{G}$. Specifically, given any drug-disease pair (v_i, v_j) where $v_i \in \mathcal{V}^{\text{drug}}$ and $v_j \in \mathcal{V}^{\text{disease}}$, we predict the probability that drug i can be used to treat disease j. We first use GraphSAGE to calculate the embedding for each node. Ideally, the node embeddings should contain two kinds of information: node attributes and node neighborhoods.

To capture the neighborhood information, we optimize GraphSAGE to encourage neighbor nodes to have similar embeddings and non-neighbor nodes to have distinct embeddings. Specifically, we perform random walks for each node to collect its neighborhood information and train the model to maximize a node's similarity with its neighbor nodes. For a node u, the loss is calculated as:

$$L_{\mathcal{G}}(z_u) = -\log(\sigma(z_u^\top z_v)) - k \cdot \mathbb{E}_{v_n \sim P_n(v)} \log(\sigma(z_u^\top z_{v_n})) \qquad (2)$$

where z_u, z_v are respectively the embeddings of nodes u, v, σ is the sigmoid function, v is a node that co-occurs with u in fixed-length random walks, P_n represents negative sampling distribution, and k indicates the number of negative samples (nodes not in u's fixed-length neighborhood).

To capture the node attributes information, we utilize the PubMedBERT model [54], a pre-trained language model designed for biomedical texts, to generate a node attribute embedding for each node based on the concatenation of the node's name and category. We further compress the embeddings to 100 dimensions with Principal Components Analysis (PCA) to reduce memory usage and use them as the initial node feature for GraphSAGE. In this way, the final GraphSAGE embedding of each node should contain the information regarding both graph topology and node attributes. We concatenate the GraphSAGE embeddings of drug-disease pairs and use them as input of a Random Forest model to classify each drug-disease pair into one of the "not treat", "treat", and "unknown" classes. We obtain "treat" and "not treat" drug-disease pairs from four data sources (described in Sec. "Data Sources for Model Training" on page 3). We generate "unknown" drug-disease pairs through negative sampling [55], that is, replacing the drug or disease identifier in each "treat" drug-disease pair with a random drug or disease identifier to generate a new pair that does not appear in both the "treat" and "not treat" classes. Specifically, for each unique "treat" drug-disease pair, we respectively replace its drug identifier with one random drug identifier as well as replace its disease identifier with one random disease identifier to make the "unknown" drug-disease pairs.

Mechanism of Action (MOA) Prediction Module

When potential indications of a given drug are identified by the drug repurposing prediction module, a natural yet essential question is: can we biologically explain the predictions? We solve this by employing a reinforcement learning (RL) model to predict the BKG-based MOA paths, which are the paths on the knowledge graph from drug nodes to disease nodes. These BKG-based MOA paths can semantically describe an abstract biological process of how a drug treats a disease.

Demonstration paths. To encourage the RL agent to terminate the path searching at the expected diseases through a biologically reasonable path, we leverage so-called "demonstration paths", a set of biologically likely paths (e.g., drug1-gene1-protein3-disease1), that explains the underlying reasons for why a drug can treat a disease. We extract 396,705 demonstration paths from the customized BKG using the known drug-target interactions collected from two curated biomedical data sources: DrugBank (v5.1) and Molecular Data Provider (v1.2) (see Additional Notes on page 14), as well as the PubMed-publication-based Normalized Google Distance (NGD) (see Equation 1). We show more details regarding demonstration path extraction in Supplementary Section S4.

Adversarial Actor-critic Reinforcement Learning. We formulate the MOA prediction as a path-finding problem and adapt the Adversarial Actor-Critic Reinforcement Learning model [41] to solve it. Reinforcement learning is defined as a Markov Decision Process (MDP) which contains:

States: Each state s_t at time t is defined as $s_t = \left(v_{drug}, v_t, (v_{t-1}, e_t), \ldots, (v_{t-K}, e_{t-(K-1)})\right)$ where $v_{drug} \in \mathcal{V}^{\text{drug}}$ is a given starting drug node; $v_t \in \mathcal{V}$ represents the node where the agent locates at time t; the tuple $\left(v_{t-K}, e_{t-(K-1)}\right)$ represents the previous Kth node and $(K-1)$th predicate. For the initial state s_0, the previous nodes and predicates are substituted by a special dummy node and predicate. We concatenate the embedding of all nodes and predicates of s_t to get the state embedding $\boldsymbol{s}_t$, where the node embeddings are node attribute embeddings generated with the PubMedBERT model (see Sec. "Drug Repurposing Prediction (DRP) Module" on page 4) and the predicate embeddings employ one-hot vectors.

Actions: The action space A_t of each node v_t includes a self-loop action a_{self} and the actions to reach its outgoing neighbors in the graph $\mathcal{G}$. Due to memory limitation and extremely large outdegree of certain nodes in the knowledge graph, we prune the neighbor actions based on the PageRank scores if a node has more than 3,000 neighbors. Specifically, we let $A_t = (a_{self}, a_1, \ldots, a_k, \ldots, a_{n_{v_t}})$ where n_{v_t} is out-degree of node $v_t \in \mathcal{V}$. For each action $a_t = (e_t, v_{t+1}) \in A_t$ taken at time t, we concatenate its node and predicate embeddings to obtain action embedding $\boldsymbol{a}_t$. We learn two embedding matrices $E^{N_\text{n} \times \text{d}}$ and $E^{N_\text{p} \times \text{d}}$ respectively for nodes and predicates (Note that each sub-network uses separate embedding matrices.), where d represents the embedding dimension, N_n represents the number of nodes in graph, and N_p represents the number of predicate categories in graph.

Rewards: During the path searching process, the agent only receives a terminal reward $R_{e,T}$ from environment (that is, there is no intermediate reward from environment: $R_{e,t} = 0, \forall t < T$). Let v_T be the last node of the path, and $\mathcal{N}_{drug}$ be the known diseases that drug v_{drug} can treat. The terminal reward $R_{e,T}$ from environment is calculated with the drug repurposing model via:

$$R_{e,T} = \begin{cases} 1, & \text{if } v_T \in \mathcal{N}_{drug}. \\ p_{treat}, & \text{if } v_T \notin \mathcal{N}_{drug}; v_T \in \mathcal{V}^{\text{disease}} \text{ and } f(v_{drug}, v_T) \text{ is predicted as "treat"}. \\ 0, & \text{if } v_T \notin \mathcal{N}_{drug}; v_T \in \mathcal{V}^{\text{disease}} \text{ and } f(v_{drug}, v_T) \text{ is not predicted as "treat"}. \\ -1, & \text{if } v_T \notin \mathcal{V}^{\text{disease}}. \end{cases}$$

where p_{treat} is the "treat" class probability predicted by the drug repurposing model f.

The Adversarial Actor-critic RL model consists of four sub-networks that share the same model architecture MLP^i (note that i represents the id of each sub-network described later, such as a for *actor network*, c for *critic network*, etc.) but with different parameters:

$$\text{MLP}^i(X) = BA(BA(XW_1^i + b_1^i)W_2^i + b_2^i)W_3^i + b_3^i \tag{3}$$

where $\{W_1^i, W_2^i, W_3^i, b_1^i, b_2^i, b_3^i\}$ are the parameters and biases of linear transformations, BA represents a batch normalization layer followed by an ELU activation function.

Actor network: The actor network learns a path-finding policy π_θ (note that θ represents all parameters of actor network) to guide the agent to choose an action a_t from the action space A_t based on the current state s_t:

$$\pi_\theta(a_t|s_t, A_t) = \text{softmax}(\boldsymbol{A_t} \odot \text{MLP}^a(\boldsymbol{s_t})) \tag{4}$$

where $\boldsymbol{A_t}$ is the embedding matrix of the action space A_t; $\odot$ represents the dot product. Here, $\pi_\theta(a_t|s_t, A_t)$ represents the probability of choosing action a_t at time t from the action space A_t given the state s_t.

Critic network: The critic network [56] estimates the expected reward $Q_\phi(s_t, a_t)$ (note that ϕ represents all parameters of the critic network) if the agent takes the action a_t at the state s_t by:

$$Q_\phi(s_t, a_t) = \text{MLP}^c(\boldsymbol{s_t}) \odot \boldsymbol{a_t} \tag{5}$$

Path discriminator network: Since the RL agent only receives a terminal reward $R_{e,T}$ from environment indicating whether it reaches an expected target, to encourage the agent to find biologically reasonable paths and provide intermediate rewards, we further guide it with demonstration paths. This network is essentially a binary classifier that distinguishes whether a path segment (s_t, a_t) is from demonstration paths or generated by the actor network. We treat all the known demonstration path segments (s_t^D, a_t^D) as positive samples and all actor-generated non-demonstration path segments (s_t^{ND}, a_t^{ND}) as negative samples. The path discriminator network $D_p(s, a) = \text{sigmoid}(\text{MLP}^p(\boldsymbol{s} \oplus \boldsymbol{a}))$, where $\boldsymbol{s}$ and $\boldsymbol{a}$ are respectively the embeddings of the state s and the action a; $\oplus$ represents the concatenation operator, is optimized with:

$$L_p = -\mathbb{E}_{(s,a)\sim P_D}[\log(D_p(s, a))] - \mathbb{E}_{(s,a)\sim P_A}[\log(1 - D_p(s, a))] \tag{6}$$

where P_D and P_A respectively represent the demonstration path segment distribution and the actor-generated non-demonstration path segment distribution. Based on the probability $D_p(s_t, a_t)$, the path-discriminator-based intermediate reward $R_{p,t}$ is calculated as:

$$R_{p,t} = \log(D_p(s_t, a_t)) - \log(1 - D_p(s_t, a_t)). \tag{7}$$

Meta-Path discriminator network: Similar to the path discriminator, this network aims to judge whether the meta-path of the actor-generated paths is similar to that of demonstration paths. The meta-path is the path of node categories (e.g., ["Drug"→"Gene"→"BiologicalProcess"→"Disease"]). Similarly, the meta-path discriminator $D_m(M) = \text{sigmoid}(\text{MLP}^m(\boldsymbol{M}))$, where $\boldsymbol{M}$ is the embedding of the meta-path M defined as the concatenation of learned category embeddings of all nodes that appear in the path, is also a binary classifier where the meta-paths of demonstration paths are treated as positive samples while others are negative samples. We optimize it with the following loss:

$$L_m = -\mathbb{E}_{M\sim P_D^M}[\log(D_m(M))] - \mathbb{E}_{M\sim P_A^M}[\log(1 - D_m(M))] \tag{8}$$

where P_D^M and P_A^M respectively represent the demonstration meta-path distribution and the actor-generated non-demonstration meta-path distribution. The intermediate reward $R_{m,t}$ generated by the meta-path discriminator is calculated by:

$$R_{m,t} = \log(D_m(M)) - \log(1 - D_m(M)). \tag{9}$$

The integrated intermediate reward R_t at time t is then calculated as:

$$R_t = \alpha_p R_{p,t} + \alpha_m R_{m,t} + (1 - \alpha_p - \alpha_m)\gamma^{T-t} R_{e,T} \tag{10}$$

where $\alpha_p \in [0, 1]$ and $\alpha_m \in [0, 1 - \alpha_p]$ are hyperparameters, γ is the decay coefficient, and $R_{e,T}$ is defined in the "Rewards" section above.

To optimize the critic network, we minimize the Temporal Difference (TD) error [57] with loss:

$$L_c = \mathrm{TD}^2 = [(R_t + Q_\phi(s_{t+1}, a_{t+1})) - Q_\phi(s_t, a_t)]^2. \quad (11)$$

Since the goal of the actor network is to achieve the largest expected reward by learning an optimal actor policy, we optimize the actor network by maximizing $J(\theta) = \mathbb{E}_{a \sim \pi_\theta}[Q_\phi(s_t, a)]$. We use the REINFORCE algorithm [58] to optimize the parameters. To encourage more diverse exploration in finding paths, we use the entropy of π_θ as a regularization term and optimize the actor network with the following stochastic gradient of the loss function L_a:

$$\nabla_\theta L_a = -\nabla_\theta J(\theta) = -\mathbb{E}_{\pi_\theta}[\nabla_\theta \mathrm{TD} \log \pi_\theta(a_t|s_t)] - \alpha \nabla_\theta \mathrm{entropy}(\pi_\theta) \quad (12)$$

where π_θ is the action probability distribution based on the actor policy, and α is the entropy weight.

We follow **(author?)** [41] to train the Adversarial Actor-critic RL model in a multi-stage way. First, we initialized the actor network using the behavior cloning method [59] in which the training set of demonstration paths is used to guide the sampling of the agent with Mean Square Error (MSE) loss. Then, in the first z epochs, we freeze the parameters of the actor network and the critic network and respectively train the path discriminator network and meta-path discriminator network by minimizing L_p and L_m. After z epochs, we unfreeze the actor network and the critic network and optimize them together by minimizing a joint loss $L_{joint} = L_a + L_c$.

Results

Evaluation Settings

Data Split

The post-processed drug-disease pairs (described in Sec. "Data Sources for Model Training" on page 3) are split into training, validation, and test sets where the drug-disease pairs of each unique drug are randomly split according to a ratio of 8/1/1. For example, let's say drugA has 10 known diseases that it treats (e.g., drugA-disease1, . . . , drugA-disease10), 8 pairs are randomly split into the training set, 1 pair is to the validation set, 1 pair to the test set. With this data split method, the model can be exposed to every drug in the training set, which complies with our goal of predicting new indications of known drugs and their potential mechanisms of action (MOAs) based on the MOA of known target diseases.

Evaluation Metrics

The proposed framework KGML-xDTD is evaluated on two types of tasks: ***predicting drug-disease "treat" probability*** (i.e., drug repurposing prediction) as well as ***identifying biologically reasonable MOA paths from all BKG-based path candidates*** (i.e., MOA prediction). These two tasks are evaluated based on classification accuracy-based metrics (e.g., accuracy, macro f1 score) and ranking-based metrics (e.g., mean percentile rank, mean reciprocal rank, and proportion of ranks smaller than K) defined as follows:

Accuracy (ACC) is the fraction of the model classification is correct, computed as:

$$\mathrm{ACC} = \frac{\text{Number of correct classifications}}{\text{Total number of drug-disease pair classifications}} \quad (13)$$

Macro F1 score (Macro-F1) is the unweighted mean of all the per-class F1 scores:

$$F1^c = 2 * \frac{precision^c \times recall^c}{precision^c + recall^c} \qquad \text{Macro-F1} = \frac{1}{|C|}\sum_{c \in C} F1^c \quad (14)$$

where C presents classification classes (e.g.,"treat," "not treat," and "unknown").

Mean Percentile Rank (MPR) is the average percentile rank of the 3-hop DrugMechDB-matched BKG-based path (described in Sec. "DrugMechDB" on page 3) of true positive drug-disease pairs:

$$\mathrm{MPR} = \frac{1}{|PR|}\sum_{pr \in PR} pr \quad (15)$$

where PR is a list of percentile ranks of DrugMechDB-matched BKG-based paths of true positive drug-disease pairs ("treat" category).

Mean Reciprocal Rank (MRR) is the average inverse rank of true positive drug-disease pairs ("treat" category) or their 3-hop DrugMechDB-matched BKG-based paths:

$$\mathrm{MRR} = \frac{1}{|R|}\sum_{r \in R} \frac{1}{r} \quad (16)$$

where R is a list of ranks of true positive drug-disease pairs (for DRP task) or DrugMechDB-matched BKG-based paths (for MOA prediction task).

Hit@K is the proportion of ranks not larger than K for true positive drug-disease pairs ("treat" category) or their 3-hop DrugMechDB-matched BKG-based paths:

$$\mathrm{Hit@K} = \frac{1}{|R|}\sum_{r \in R} |r \le k| \quad (17)$$

where R is a list of ranks of true positive drug-disease pairs (for DRP task) or DrugMechDB-matched BKG-based paths (for MOA prediction task).

Drug Repurposing Prediction Evaluation Method

We utilize the metrics *ACC* and *Macro-F1* to measure the accuracy of drug repurposing prediction of our KGML-xDTD framework while using ranking-based metrics *MRR* and *Hit@K* to show its capability in reducing false positive (i.e., the false drug-disease pairs ranking higher among possible drug-disease candidates). We use the following three methods to generate non-true-positive drug-disease candidates for each true positive drug-disease pair to calculate the ranks that are employed in the *MRR* and *Hit@K* calculation:

- **Drug-rank-based replacement**: For each true positive drug-disease pair, the drug-rank-based replacement pairs are generated by replacing the drug entity with each of all 274,676 other drugs in the customized BKG while excluding all known true positive drug-disease pairs.
- **Disease-rank-based replacement**: For each true positive drug-disease pair, the disease-rank-based replacement pairs are generated by replacing the disease entity with each of all 124,638 other diseases in the BKG while excluding all known true positive drug-disease pairs.
- **Combined Replacement**: For each true positive drug-disease pair, the combined replacement pairs are the combination of all replacement pairs of the above two methods. All known true positive drug-disease pairs are excluded from these replacement pairs.

Due to the massive size of possible drug-disease candidates, some baseline models (e.g., GAT and GraphSAGE+SVM) are not applicable in this setting within a reasonable time (e.g., a week). Thus, we also employ a small subset of drug-disease replacements to calculate the *MRR* and *Hit@K*, allowing for comparison between KGML-xDTD with all baselines. Specifically, we utilize 1,000 random drug-disease pairs from the combined replacement set above: 500 with drug ID replacement and 500 with disease ID replacement. To enhance the robustness of results obtained through this random replacement method, we use this method to generate 10 sets of random drug-disease pairs (each with 1,000 pairs) independently and calculate the mean and standard deviation of the ranking-based metrics outcomes. In addition, since the drug repurposing prediction module of KGML-xDTD framework does 3-class classification while other baselines do 2-class classification, for a fair comparison, we re-calculate *ACC* and *Macro-F1* for KGML-xDTD by excluding the "unknown" class.

Table 2. The performance comparison of Drug Repurposing Prediction (DRP) between `KGML-xDTD` and different baseline models based on test set (described in Sec. "Data Split" on page 6). The top panel shows the performance of state-of-the-art (SOTA) baseline models; the middle panel shows the performance of variants of `KGML-xDTD` model framework; the bottom panel shows the performance of `KGML-xDTD` model framework.

Model	Accuracy	Macro F1 score	MRR	Hit@1	Hit@3	Hit@5
TransE	0.708	0.708	0.301 (+/-0.005)	0.134 (+/-0.007)	0.327 (+/-0.009)	0.482 (+/-0.007)
TransR	0.858	0.855	0.329 (+/-0.006)	0.150 (+/-0.009)	0.378 (+/-0.008)	0.542 (+/-0.005)
RotatE	0.704	0.704	0.281 (+/-0.007)	0.098 (+/-0.008)	0.314 (+/-0.007)	0.497 (+/-0.009)
DistMult	0.555	0.495	0.182 (+/-0.004)	0.042 (+/-0.002)	0.157 (+/-0.010)	0.292 (+/-0.010)
ComplEx	0.624	0.460	0.138 (+/-0.004)	0.026 (+/-0.004)	0.106 (+/-0.007)	0.205 (+/-0.008)
ANALOGY	0.594	0.465	0.188 (+/-0.004)	0.044 (+/-0.004)	0.165 (+/-0.009)	0.301 (+/-0.008)
SimplE	0.599	0.472	0.167 (+/-0.006)	0.036 (+/-0.006)	0.140 (+/-0.008)	0.259 (+/-0.011)
GAT	**0.936**	**0.934**	0.002 (+/-0.000)	0.000 (+/-0.000)	0.000 (+/-0.000)	0.000 (+/-0.000)
GraphSAGE-link	0.919	0.915	0.002 (+/-0.000)	0.000 (+/-0.000)	0.000 (+/-0.000)	0.000 (+/-0.000)
GraphSAGE+logistic	0.791	0.784	0.002 (+/-0.000)	0.000 (+/-0.000)	0.000 (+/-0.000)	0.000 (+/-0.000)
GraphSAGE+SVM	0.807	0.793	0.002 (+/-0.000)	0.000 (+/-0.000)	0.000 (+/-0.000)	0.000 (+/-0.000)
KGML-xDTD w/o NAEs	0.909 (0.898*)	0.891 (0.892*)	0.159 (+/-0.003)	0.035 (+/-0.002)	0.143 (+/-0.006)	0.262 (+/-0.008)
2-class KGML-xDTD	0.929	0.925	0.278 (+/-0.003)	0.183 (+/-0.006)	0.321 (+/-0.003)	0.389 (+/-0.006)
KGML-xDTD (ours)	0.935 (0.930*)	0.923 (0.926*)	**0.382 (+/-0.004)**	**0.238 (+/-0.007)**	**0.425 (+/-0.006)**	**0.543 (+/-0.006)**

1. The values with * inside the parenthesis are the adjusted results by excluding the "unknown" category for a fair comparison.
2. The ranking metrics (e.g., "MRR" and "Hit@K") are calculated as the mean along with standard deviation based on 10 independent sets of non-true-positive drug-disease candidates generated by the random drug-disease replacement method (i.e., for each true positive drug-disease pair in test set, we use 1,000 random drug-disease pairs as non-true-positive drug-disease candidates to calculate the rank). See more details in Sec. "Drug Repurposing Prediction Evaluation Method" on page 6.
3. The abbreviation "w/o NAEs" in the name of model "KGML-xDTD w/o NAEs" represents without using node attribute embeddings.

MOA Prediction Evaluation Method

For the evaluation of MOA prediction, we use the DrugMechDB [42] to obtain the expert-verified MOA paths as ground-truth data and match each biological concept in these verified MOA paths to the biological entities used in the customized BKG, and then generate the BKG-based matched paths of DrugMechDB drug-disease pairs (described in Sec. "DrugMechDB" on page 3), which are considered as biologically meaningful MOA paths. We first calculate the path scores for all 3-hop KG paths between drug and disease with the path-finding policy learned from Adversarial Actor-critic Reinforcement Learning (RL) model using equation:

$$\text{path score} = \sum_{i=1}^{k} \delta^{i-1} \times \log(P_i \times N_i) \tag{18}$$

where k is the number of hops in this path; δ is a decay coefficient (we set it to 0.9 in this study); P_i represents the probability of choosing action a_i in the ith hop following this path based on the trained RL model; N_i is the number of possible actions in the ith hop.

With the path scores, we obtain the ranks of the DrugMechDB-matched BKG-based paths and calculate their ranking-based metrics (e.g., *MPR*, *MRR*, and *Hit@K*). For those drug-disease pairs with multiple matched paths, we use the highest ranks of the matched paths as their ranks in the metrics calculation. We compare `KGML-xDTD` with the baseline models based on these metrics to show the capability of MOA prediction module of `KGML-xDTD` in identifying biologically reasonable MOA paths from a massive and complex BKG with comparably low false positive. In addition, we further perform two case studies to evaluate the effectiveness of `KGML-xDTD` in identifying the biologically reasonable MOA paths.

Drug Repurposing Prediction Evaluation

For drug repurposing prediction evaluation, we compare the `KGML-xDTD` model framework against several state-of-the-art (SOTA) KG-based models and variants of `KGML-xDTD` for drug repurposing prediction based on the method described in Sec. "Drug Repurposing Prediction Evaluation Method" on page 6.

We use eight different SOTA KG-based models as baseline models that are commonly used for BKG-based drug repurposing [19, 60]. `TransE` [25], `TransR` [61], `RotatE` [26] are the translation-distance-based models that regard a relation (e.g., "treats") as a "translation"/"rotation" (e.g., a kind of spatial transformation) from a head entity (e.g., a drug node) to a tail entity (e.g., a disease node). `DistMult` [27] is a bilinear model that measures the latent semantic similarity of a knowledge-graph triple (head entity, relation/predicate, tail entity) with a trilinear dot product. `ComplEx` [28] and `ANALOGY` [62] are the extensions of `DistMult` that consider more complex relations (e.g., asymmetric relations). `SimpLE` [63] is a tensor-factorization-based model to learn the semantic relation of a knowledge-graph triple. `GAT` [64] is a popular graph neural model that leverages the important graph topology structure based on self-attention mechanism for graph-associated tasks (e.g., link prediction). Implementation details of these baselines are presented in Supplementary Section S5.

Besides these SOTA baseline models, we also compare the drug repurposing prediction module in `KGML-xDTD` with its several variants to show the effectiveness of model components. For example, to show efficacy of the combination of GraphSage and Random Forest (RF), we use a pure GraphSAGE model for link prediction (`GraphSAGE-link`), the combination of GraphSage and logistic model (`GraphSAGE-logistic`), and the combination of GraphSage and Support Vector Machine (SVM) model (`GraphSAGE-SVM`). To demonstrate the effectiveness of node attribute embeddings (described in Sec. "Drug Repurposing Prediction (DRP) Module" on page 4) in improving repurposing prediction, we conduct an ablation experiment that replaces node attribute embeddings (NAEs) with random embeddings (initialized with the Xavier method [65]) as GraphSage initialized embeddings (`KGML-xDTD w/o NAE`); to support rationality of setting "unknown" class through negative sampling (described in Sec. "Drug Repurposing Prediction (DRP) Module" on page 4), we modify the drug repurposing prediction module for 2-class classification (i.e., true positive and true negative) (`2-class KGML-xDTD`) as a baseline comparison model.

Table 2 shows the performance of `KGML-xDTD` model and all other baseline models in the task of drug repurposing prediction based on test set (described in Sec. "Data Split" on page 6). For the cacualtion of the *MRR* and *Hit@K* used in this table, we utilize the random subset replacement method described in Sec. "Drug Repurposing Prediction Evaluation Method" on page 6. As shown in the table, on the one hand, the `KGML-xDTD` outperforms most of the baseline models and achieves comparable performance as `GAT` in classification-based metrics (e.g., accuracy, macro f1 score), indi-

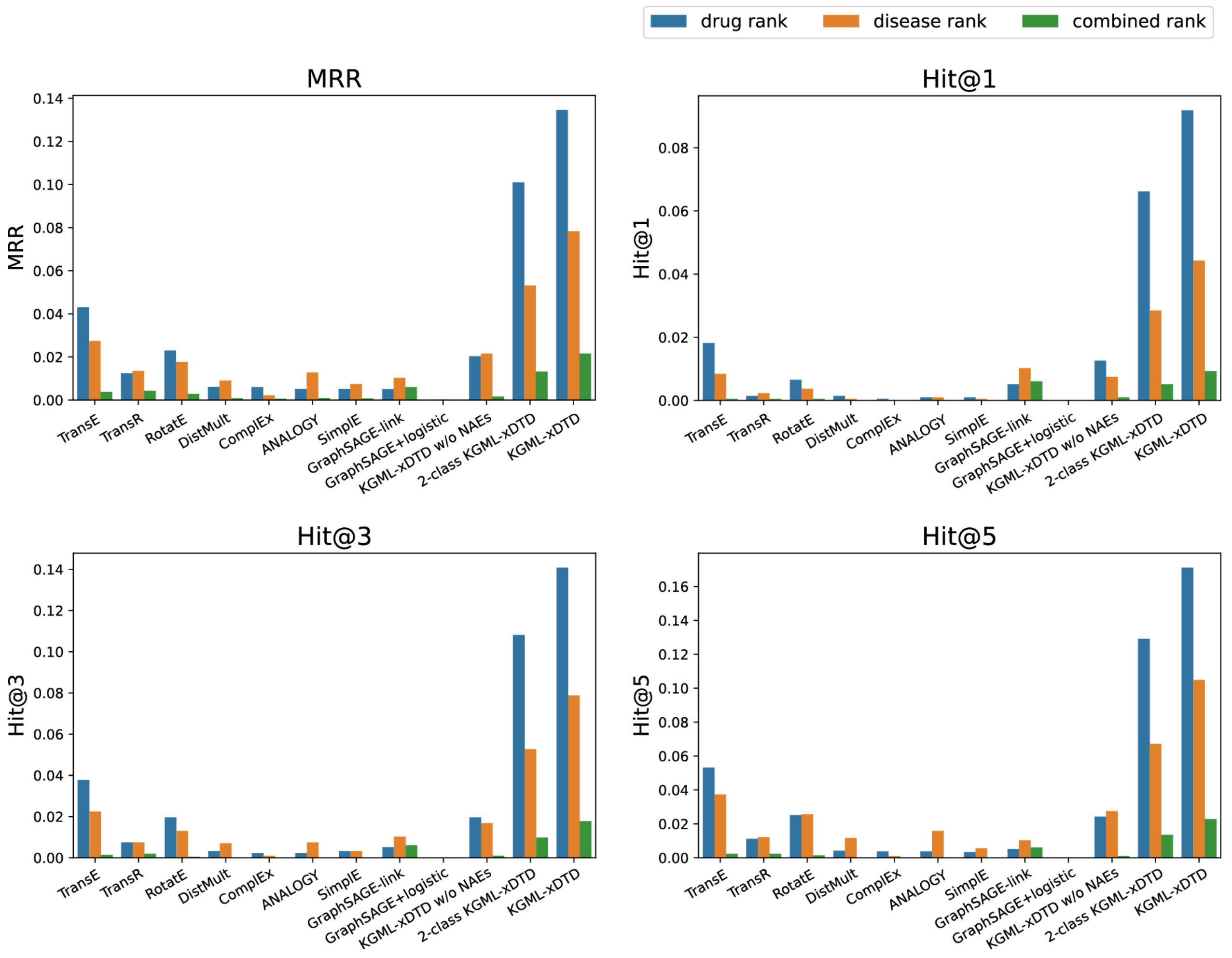

Figure 3. The performance comparison of Drug Repurposing Prediction (DRP) between `KGML-xDTD` and different baseline models (`GAT` and `GraphSAGE+SVM` are excluded due to computation time constraints) based on test set using three "complete" replacement methods (i.e., Drug-rank-based replacement", "Disease-rank-based replacement", and "Combined replacement" described in Sec. "Drug Repurposing Prediction Evaluation Method" on page 6) to generate non-true-positive drug-disease candidates for each true positive drug-disease pair for MRR and Hit@K calculation. The legend "drug rank", "disease rank" and "combined rank" respectively correspond to the methods of "Drug-rank-based replacement", "Disease-rank-based replacement", and "Combined replacement".

cating its effectiveness in classifying known "treat" and "not treat" drug-disease pairs with both attribute and neighborhood information on the knowledge graph. On the other hand, KGML-xDTD's exceptional performance in ranking-based metrics shows its superiority over baselines in identifying new indications of existing drugs out of a large number of possible drug-disease pairs with relatively low false positives, which is of great importance for guiding clinical research. Figure 3 displays the comparison results where we calculate the *MRR* and *Hit@K* with three different "complete" replacement methods (described in Sec. "Drug Repurposing Prediction Evaluation Method" on page 6). Although GAT and GraphSAGE+SVM are excluded in this comparison due to computation time constraints, we can see that the results presented in both Table 2 and Figure 3 are consistent to demonstrate KGML-xDTD's ability in reducing false positive. Therefore, excluding the GAT and GraphSAGE+SVM from the comparison with "complete" replacement methods does not affect the conclusion. Besides, by comparing 2-class KGML-xDTD with the vanilla GraphSAGE model (e.g., GraphSAGE-link), we demonstrate the effectiveness of the Random Forest model over a neural network classifier in this task. The comparison between KGML-xDTD w/o NAE and KGML-xDTD shows that the KGML-xDTD benefits from the use of node attribute embeddings for drug repurposing prediction while the comparison with 2-class KGML-xDTD indicates the effectiveness of using negative sampling to generate "unknown" drug-disease pairs for model training. With the "unknown" drug-disease pairs, the KGML-xDTD model achieves significant improvement in ranking-based metrics, which is essential when applying to real-world drug repurposing because it can reduce the false positives.

MOA prediction evaluation

For MOA prediction, we evaluate how well the KGML-xDTD can identify the DrugMechDB-matched BKG-based MOA paths (described in Sec. "MOA Prediction Evaluation Method" on page 7) from a large number of possible paths in the customized BKG by utilizing ranking-based metrics (e.g., MPR, MRR, and Hit@K) and two specific case studies.

There are few machine learning models designed for the task of identifying biologically meaningful paths from biomedical knowledge graphs for explaining drug repurposing. Although the UKGE [30], GrEDeL [32], Polo [38] models (all mentioned in Sec. "Introduction" on page 1) were proposed and can be used for this goal, they all have certain constraints and cannot be used as baseline models for comparison. The UKGE model cannot be applied to BKGs without weighted edge information (e.g., frequency of relation appeared in literature). The authors of the GrEDeL model don't provide the code to implement this model. The Polo model cannot be trained within a reasonable time (e.g., within two weeks) on a massive and complex BKG (e.g., RTX-KG2) due to its dependence on a computationally inefficient method "DWPC" [39]. Therefore, we choose the MultiHop reinforcement learning model [66] as a baseline model since it uses a similar LSTM model framework as the GrEDel model and allows using a self-defined reward shaping strategy in its reward function as what we do in the KGML-xDTD model (i.e., we can use the same reward strategy described in Sec. "Adversarial Actor-critic Reinforcement Learning" on page 5). Furthermore, we also compare with an ablated version of KGML-xDTD (i.e., KGML-xDTD w/o DP which does not take advantage of the demonstration paths by setting α_p and α_m in Function 9 as 0) as another baseline model to show the importance of proposed demonstration paths.

We compare the MOA prediction performance between the KGML-xDTD model framework and different baseline models in Table 3. Although all the models use the same terminal reward function from the environment, that is the drug repurposing prediction module of KGML-xDTD, the MOA prediction module of KGML-xDTD achieves significantly better performance in identifying DrugMechDB-matched BKG-based MOA paths than the other two baselines across all ranking-based metrics. Comparison between the KGML-xDTD with and without demonstration paths (i.e., KGML-xDTD w/o DP) further illustrates the great effectiveness of using proposed demonstration paths to guide the path-finding process. Due to the massive searching space and sparse rewards, the RL agent often fails to find biologically reasonable BKG-based MOA paths out of many possible choices, while our model KGML-xDTD, with the intermediate guidance provided by the demonstration path, is able to identify those biologically reasonable choices with a much higher probability. Moreover, comparing KGML-xDTD w/o DP and MultiHop reveals that the actor-critic model structure performs similarly to LSTM. However, incorporating the proposed demonstration paths can significantly enhance the effectiveness of the actor-critic model structure over LSTM for this task.

To further evaluate the performance of KGML-xDTD model framework in identifying biologically relevant MOA paths for drug repurposing, we present two different case studies to explore the potential repurposed drugs and their potential mechanism for two rare genetic diseases: Hemophilia B and Huntington's disease.

Case 1: Hemophilia B

Hemophilia B, also known as factor IX deficiency or Christmas disease, is a rare genetic disorder that results in prolonged bleeding in patients. It is caused by mutations in the factor IX (F9) gene, which is located on the X chromosome. Table 4 displays the top 10 drugs/treatments predicted by the KGML-xDTD model framework, including both those that are used in the training set (highlighted in red) and those that are not. Besides those known drugs/treatments used in the training set, the majority of the remaining seven drugs/treatments on the list are supported by published research and have the potential to treat hemophilia B. For example, the activated human-derived coagulation factor VII (i.e., Factor VIIa) or the recombinant activated factor VII (i.e., rFVIIa) is one of the proteins that can cause blood clots as an important part of the blood coagulation regulatory network (as shown in the Figure 4). This protein is used as an effective inhibitor in the treatment of patients with hemophilia B [71, 67]. Thrombin is a key enzyme in the maintenance of normal hemostatic function. It has been reported that using thrombin as a therapeutic strategy can help prevent bleeding in patients with hemophilia [72]. The use of recombinant factor IX therapy is a recommended treatment option for individuals with hemophilia B [73]. Some examples of recombinant factor IX products include BeneFIX, Rixubis, Ixinity, Alprolix Idelvion, and Rebinyn. These examples demonstrate the potential capability of KGML-xDTD for drug repurposing in real-world applications.

To further assess the biological explanations of the predicted 3-hop BKG-based MOA paths for the treatment of hemophilia B, we have used the curated DrugMechDB-based MOA paths, which are not used in the model training process. DrugMechDB contains relevant MOA paths of hemophilia B treatment only for Eptacog Alfa and Nonacog Alfa. We use the KGML-xDTD model to predict the top 10 potential 3-hop BKG-based MOA paths for these two drugs and compare them with the curated DugMechDB-based MOA paths in Figure 5 (For visualization purpose, we only display the top 5 predicted paths along with any available DrugMechDB-matched BKG-based paths in the top 10 predicted paths). The corresponding biological entities between the predicted paths and the curated DrugMechDB-based paths are highlighted in red color. Although the predicted paths can't exactly match the DrugMechDB-based MOA paths due to the limited path length and some missing semantic relationships in the customized biomedical knowledge graph, key biological entities (such as Coagulation Factor VII, Coagulation Factor X, and Coagulation Factor IX) that are important for the treatment of hemophilia B are present in the predicted paths. As shown in Figure 4, the treatment of hemophilia B involves a complex molecular network of blood coagulation, and many of the coagulation factors (such as factor VII, factor III, factor II, factor VIII,

Table 3. The performance comparison of Mechanism of Action (MOA) Prediction between `KGML-xDTD` and different baseline models (e.g., `MultiHop` and `KGML-xDTD w/o DP`) based on the test set (described in Sec. "Data Split" on page 6). The metrics in this table are calculated using path scores and all non-DrugMechDB-matched 3-hop paths between drug and disease as "negative" paths for each true positive drug-disease pair (see more details in Sec. "MOA Prediction Evaluation Method" on page 7).

Model	MPR	MRR	Hit@1	Hit@10	Hit@50	Hit@100	Hit@500
MultiHop	61.400%	0.027	0.017	0.042	0.067	0.118	0.345
KGML-xDTD w/o DP	72.965%	0.015	0.008	0.017	0.067	0.160	0.403
KGML-xDTD (ours)	**94.696%**	**0.109**	**0.059**	**0.193**	**0.496**	**0.613**	**0.849**

1. The abbreviation "w/o DP" in the name of model "KKGML-xDTD w/o DP" represents "without using demonstration paths".

Table 4. Top 10 predicted drugs/treatments for hemophilia B (note that the drugs highlighted in red color are used in the training set).

Drug/Treatment	Prob.	Publications
Eptacog Alfa (rFVIIa)	0.833	[67, 68]
Nonacog Alfa (rFIX)	0.803	[69]
Viral Vector	0.780	[70]
Factor VIIa	0.748	[71, 67]
Recombinant FVIIa (rFVIIa)	0.724	[71, 67]
Thrombin	0.709	[72]
Factor IX	0.708	[73]
Epicriptine	0.702	
Hyperbaric Oxygen	0.660	
Triamcinolone	0.649	

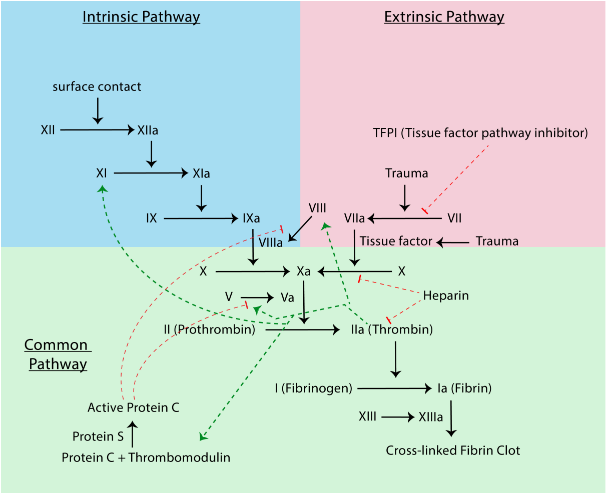

Figure 4. Blood Coagulation Regulatory Network with arrows for molecular reactions (black), positive feedback (green), and negative feedback (red).

factor IX, and factor X) present in the predicted paths are also part of this molecular network. In Supplementary Section S6, we also utilize the `KGML-xDTD` model framework to predict the top 10 3-hop BKG-based paths, which can serve as biological explanations of the predicted "treats" relationship between Factor VIIa and Hemophilia B (shown in Table 4). This particular drug/treatment - disease pair is not included in the training set and thus can be used to indicate how `KGML-xDTD`'s MOA path predictions can contribute to the explanation of the predicted drug repurposing results. The predicted paths show molecular details akin to those in Figure 4 for treating hemophilia B. As a result, the predicted paths by `KGML-xDTD` model framework can help identify key molecules in the real drug action regulatory network, thereby aiding in explaining drug repurposing to some extent.

Case 2: Huntington's disease

Huntington's disease (HD) is a rare neurogenetic disorder that typically occurs in midlife with symptoms of depression, uncontrolled movements, and cognitive decline. While there is currently no drug/treatment that can alter the course of HD, some drugs/treatments can be useful for the treatment of its symptoms in abnormal movements (e.g., chorea) and psychiatric phenotypes. We show ten drugs/treatments with the highest predicted probability by the `KGML-xDTD` model framework after manual processing in Table 5. This processing involves excluding the chemotherapeutic drugs from the predicted drug candidate list due to their potential risk of cytotoxicity to normal cells (which could lead to false positives for drug repurposing of non-cancer diseases [74, 75]), and only presenting the top 5 results in the training set, and top 5 from the test or validation set. From this table, it can be observed that many of the top-ranked predicted drugs have been supported by publications as potential treatments for the symptoms of HD. Since there is currently no effective treatment for HD, DrugMechDB does not have a corresponding MOA path for comparison. To analyze the predicted paths by the `KGML-xDTD` model framework for the predicted non-chemotherapeutic drugs/treatments that are not included in the training set (shown in black in Table 5), we present their top 5 predicted paths in Figure 6. From these predicted paths, we can see that most of them are biologically relevant. For example, the subfigure (a) of Figure 6 shows that Risperidone is predicted to be useful for the treatment of HD by decreasing the activity of the genes associated with the 5-Hydroxytryptamine receptor (e.g., HTR1A, HTR2A, HTR2C, HTR7) and dopamine receptor (e.g., DRD2) which have been proven to be involved in the pathogenesis of depressive disorders [76, 77]. The presence of depressive symptoms is a significant characteristic of HD [78]. Entinostat is predicted to have the potential to alleviate the symptoms of HD by inhibiting the functions of histone deacetylase genes such as HDAC1, HDAC6 (see subfigure (b) of Figure 6), and one of the predicted 3-hop BKG-based MOA paths ("Entinostat" → "decreases activity of" → "HDAC1 gene" → "interacts with" → "Histone H4" → "gene associated with condition" → "Huntington's disease") is supported by the previous research [79, 80]. Primaquine is predicted to act on the IKBKG gene to potentially play a therapeutic role in neurodegenerative disease (see subfigure (c) of Figure 6), reported in [81]. According to the predicted BKG-based MOA paths (see subfigure (d) of Figure 6), Isradipine may have a potential therapeutic effect for HD by mainly regulating the genes of the Calcium Voltage-Gated Channel, including CACNA1C, CACNB2. These genes may be associated with the symptoms of HD, such as depression, and dementia [82]. Lastly, Amifampridine is predicted to regulate the genes of the Potassium Voltage-Gated Channel such as (see subfigure (e) of Figure 6) which are potentially associated with HD [83]. All these examples indicate that the predicted BKG-based MOA paths can explain the mechanism of repurposed drugs to some extent.

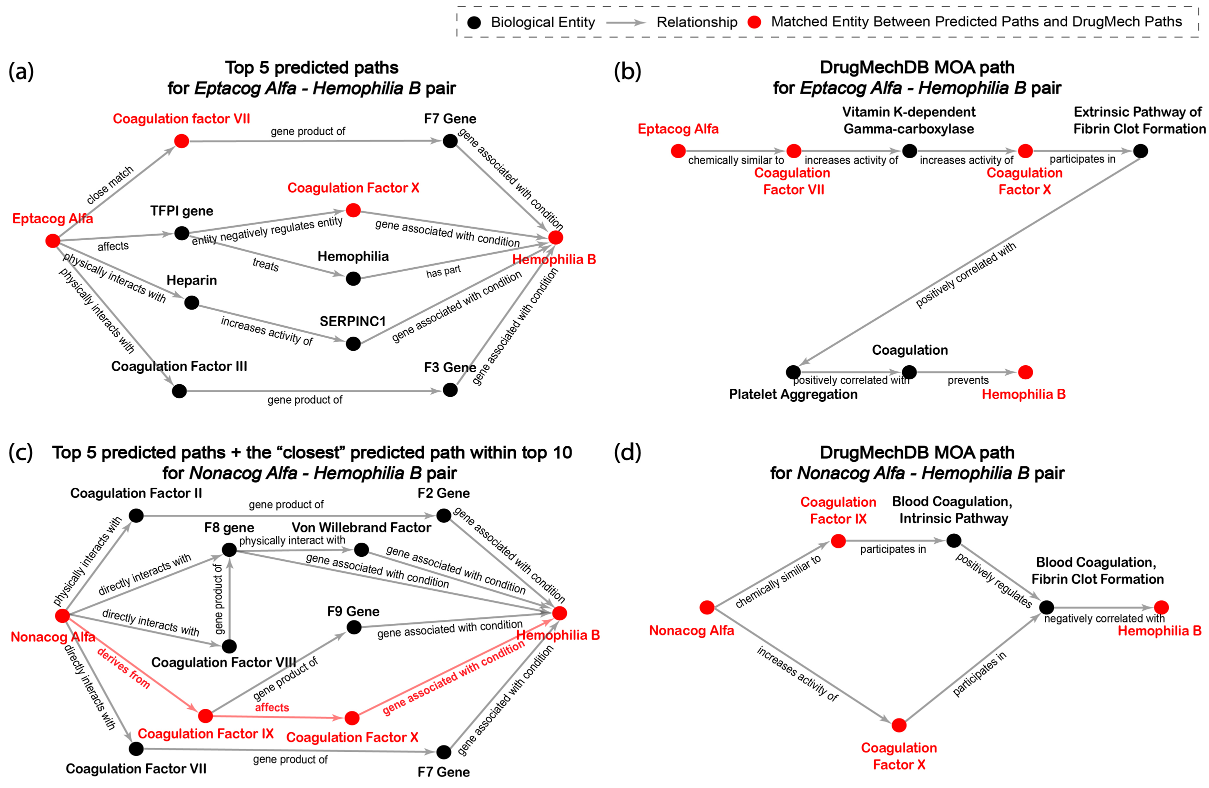

Figure 5. Comparison between the top 5 predicted 3-hop paths (including any available DrugMechDB-matched BKG-based paths in the top 10 predicted paths, highlighted in red) and the curated DugMechDB-based MOA paths for Eptacog Alfa and Nonacog Alfa. Note that the RTX-KG2 paths and DrugMechDB paths might use different synonyms for the same biological concept. For better visualization and illustration, we utilize consistent entity synonyms between the predicted paths and the curated paths as well as present the predicted paths in a graph structure. The subfigures (a) and (c) are the graph representation of predicted paths generated by `KGML-xDTD` respectively for Eptacog Alfa and Nonacog Alfa while the subfigures (b) and (d) are the human-curated DrugMechDB mechanism of action (MOA) paths.

Table 5. Top 5 predicted drugs/treatments used in the training set (highlighted in red color) and the top 5 non-chemotherapeutic predicted drugs/treatments that are not in the training set for Huntington's Disease.

Drug/Treatment	Prob.	Publications
Pimozide	0.939	[84, 85]
Therapeutic Agent	0.939	
Olanzapine	0.938	[86, 87]
Riluzole	0.935	[88]
Antipsychotic Agent	0.932	[89]
Risperidone	0.893	[78, 90]
Entinostat	0.888	[79]
Primaquine	0.887	
Isradipine	0.884	[91]
Amifampridine	0.882	

Drug Class Analysis

The Drug Repurposing Prediction of the `KGML-xDTD` model does not leverage any information regarding drug similarity such as drug classes, SMILES, drug side effects, drug-related gene profiles/sequences, and we find that the distribution of drug classes in true positive drug-disease pairs is similar between the training and test sets (see Figure 7). In this section, we examine whether our model can only predict the drugs with the drug classes that it has seen in the training set.

To do this, we use the MyChem.info APIs [48] to retrieve the FDA's "Established Pharmacologic Class" (EPC) information for chemicals/drugs using their synonym identifiers. For the FDA-unapproved chemical/drug without such EPC information, we consider it as as a single class. We first utilize the `KGML-xDTD` model to predict the top 100 chemicals/drugs for each of the 1,140 diseases in the test set (described in Sec. "Data Split" on page 6) after excluding the drug-disease pairs presented in the training set. Then we count the number of drug classes among these 100 predicted drugs that are not seen in the training set for each disease. Figure 8) shows the distribution of unseen drug classes in top 100 predicted non-train drugs across the 1,140 diseases in test set . We can see that each disease has at least 70 different drug classes among the top 100 predicted drugs, indicating that the predictive power of the `KGML-xDTD` model is derived from the node attribute information and knowledge graph topology structure rather than any drug class information.

Discussion

In this work, we propose `KGML-xDTD`, a two-module, knowledge graph-based machine learning framework that not only predicts the treatment probabilities between drugs/compounds and diseases but also provides biological explanations for these predictions through the predicted paths in a massive biomedical knowledge graph with comprehensive biomedical data sources as potential mechanisms of action. This framework can assist medical researchers in quickly identifying the potential drug/compound-disease pairs that might have a treatment relationship, which can accelerate the process of drug discovery for emerging diseases. Additionally, by leveraging the KG-based MOA paths predicted by the framework, medical professionals (e.g., doctors and licensed medical practitioners) can straightforwardly assess the accuracy of the predictions, which can help to reduce false positives that may be produced by the "black-box" operation of traditional machine learning models.

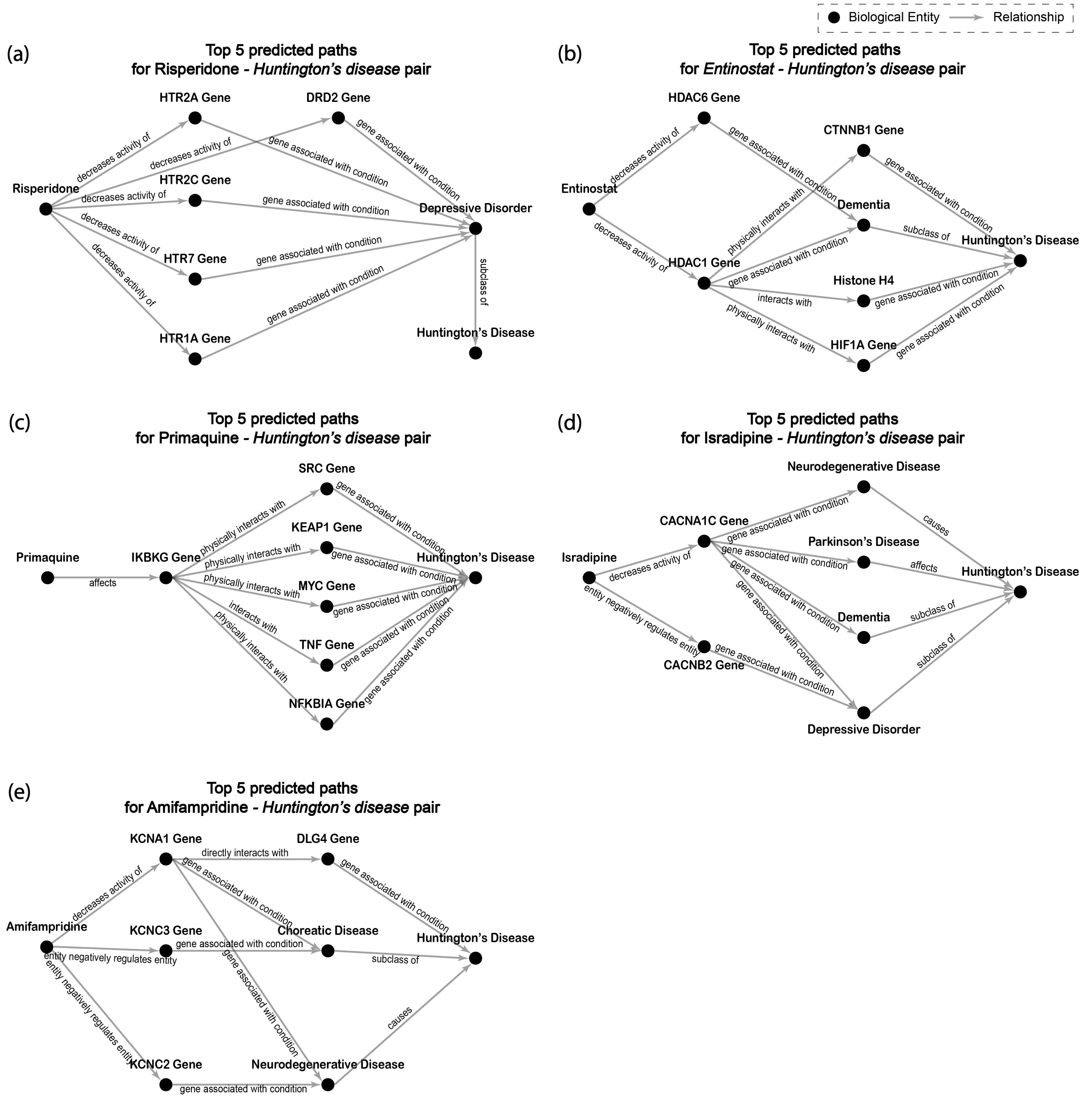

Figure 6. Top 5 predicted 3-hop BKG-based MOA paths (integrated into a graph for better visualization) for top 5 non-chemotherapeutic predicted drugs/treatments that are not included in the training set for Huntington's disease.

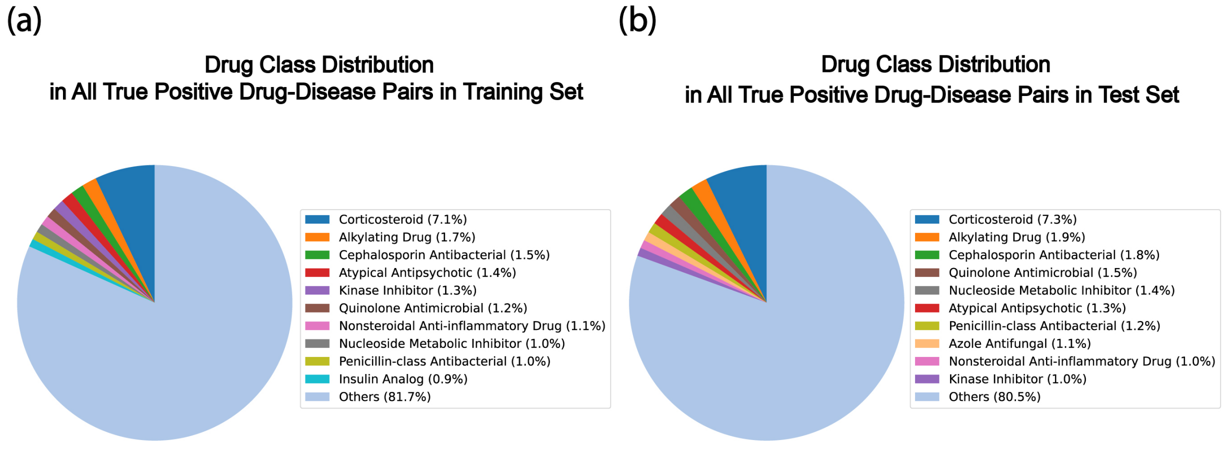

Figure 7. Comparison of the drug class distribution in true positive drug-disease pairs between the training set and test set. The Drug class of each drug/chemical in these pairs are determined based on the FDA "Established Pharmacologic Class" (EPC) accessed via MyChem.info APIs. There are 2,238 drug classes represented in the true positive drug-disease pairs in the training set while 718 drug classes in the test set. For visualization purpose, we only show the top 10 drug classes in each set and the rest is classified into the "Others" class.

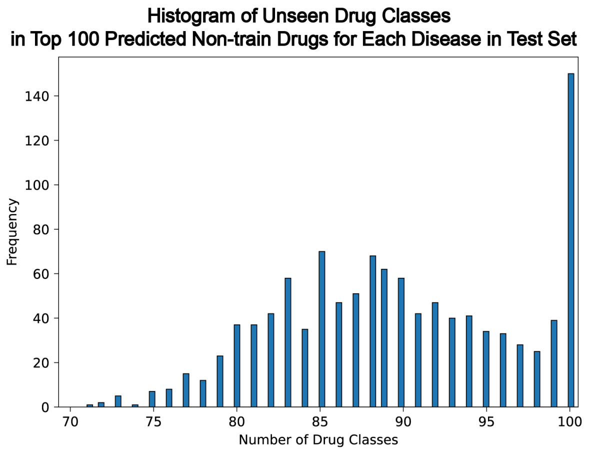

Figure 8. Distribution of unseen drug classes in the top 100 predicted non-train drugs across the 1,140 diseases in the test set.

Although previous research [15, 19, 24] has applied a variety of models to the task of drug repurposing using biomedical knowledge graphs (BKGs), these approaches are implemented in the small-scale BKGs and many do not scale to larger and more complex graphs as biotechnology advances and the volume of data in biomedical databases increases. In our comparison with state-of-the-art KG-based models for drug repurposing, we find that the `KGML-xDTD` model had higher accuracy with lower false positives when applied to a massive and complex biomedical knowledge graph *RTX-KG2c*. By evaluating the predicted paths with DrugMechDB and two case studies, we show that the model can capture some key biological entities involved in real drug action regulatory networks.

It is widely acknowledged that drug repurposing is one of the most challenging problems in biomedicine, and current AI techniques are still in the early stages of addressing it. Many other AI models, such as those based on chemical structure, drug-target interactions, and drug perturbations of gene expression, are developed for solving this goal. They may offer more accurate predictions but also have limitations in terms of cost and the availability of samples for specific diseases. Biomedical knowledge graph (BKG)-based machine learning models, such as the `KGML-xDTD` model, offer a cost- and time-efficient alternative due to the large volume of biomedical knowledge stored in public databases and publications.

The `KGML-xDTD` model framework is not intended to replace or beat these models, but rather provides a complementary approach that leverages emerging knowledge graphs for drug repurposing.

Future work to further enhance the `KGML-xDTD` model framework might include extending the predicted paths for more specific explanations, and considering the negative drug-disease pairs so that the model can explain why certain drugs are harmful to diseases.

Availability of source code and requirements

- **Project name**: KGML-xDTD
- **Project home page**: https://github.com/chunyuma/KGML-xDTD
- **Operating system(s)**: Linux (Ubuntu)
- **Resource usage in training step**: A Linux (Ubuntu) system with at least 8 CPU cores, 800GB of VRAM, and a 48GB GPU card (48GB Quadro RTX 8000 GPU card used in our training).
- **Resource usage in inference step**: A Linux (Ubuntu) system with at least 8 CPU cores and 50GB of VRAM. The GPU card is not necessary but if used, the GPU card needs at least 24GB VRAM (48GB Quadro RTX 8000 GPU card used in our inference).
- **Time Requirement**: Based on our hardware performance and parameter settings (please see the scripts on Github), the training step takes approximately two weeks while the inference step takes approximately 25.42 seconds for one drug-disease pair with 3,320 potential paths. These time estimates may vary depending on the hardware performance, parameter settings, and the number of potential paths of given drug-disease pair.
- **Programming language**: Shell Script (Bash) with Python 3.8.12
- **Other requirements**: Python 3.8.12 with GPU/CPU support (GraphSAGE training needs Python 2.7), neo4j-community 3.5.26, miniconda 4.8.2 (please see more requirements in the yaml files under "envs" folder on Github repository)
- **Licenses**: MIT license, DrugBank academic license, Apache 2.0 license, UMLS Metathesaurus license, CC-BY 4.0 license
- **Research Resource Identifier (#RRID)**: SCR_023678

Data Availability

The data sets supporting the results of this article are publicly available in the Zenodo repository [92]. All supporting data and materials are available in the GigaScience GigaDB database [93].

Additional Files

Supplementary Section S1. Biomedical Knowledge Graph RTX-KG2c Pre-processing.
Supplementary Section S2. Summary of Data Resources Used by MyChem Data.
Supplementary Section S3. Implementation Details of `KGML-xDTD` Model Framework.
Supplementary Section S4. Implementation Details of Demonstration Path Extraction.
Supplementary Section S5. Implementation Details of Baseline Models.
Supplementary Section S6. Top 10 KGML-xDTD's Predicted Paths Serving as Biological Explanations for the Predicted "Treats" Relationship between Factor VIIa and Hemophilia B.

Declarations

List of abbreviations

ACC: Accuracy;
ADAC: ADversarial Actor-Critic;
BKG: Biomedical Knowledge Graph;
DWPC: Degree-Weighted Path Count;
EHR: Electronic Health Record;
GRL: Graph Reinforcement Learning;
KG: Knowledge Graph;
LSTM: Long Short-Term Memory;
Macro-F1: Macro F1 score;
MDP: Markov Decision Process;
MeSH: Medical Subject Heading;
MOA: Mechanism of Action; RL: Reinforcement Learning;
MPR: Mean Percentile Rank;
MSE: Mean Square Error;
NAE: Node Attribute Embedding;
NGD: Normalized Google Distance;
NLP: Natural Language Processing;
PCA: Principal Components Analysis;
RF: Random Forest;
RNN: Recurrent Neural Network;
RTX-KG2:the Reasoning Tool X Knowledge Graph 2;
RTX-KG2c:the canonicalized version of the Reasoning Tool X Knowledge Graph 2;
SemMedDB: Semantic MEDLINE Database;
SVM: Support Vector Machine;
VHA: Veterans Health Administration;

Additional Notes

Molecular Data Provider: A knowledge-centric data provider for systems chemical biology, as part of the NCATS Biomedical Data Translator ("Translator"). See more in `https://github.com/NCATSTranslator/Translator-All/wiki/Molecular-Data-Provider`.[94]

Ethical Approval (optional)

Not applicable

Consent for publication

Not applicable

Competing Interests

The authors declare no competing interests.

Funding

Research reported in this publication was supported by the National Center for Advancing Translational Sciences (NCATS) of the National Institutes of Health (NIH) under award numbers: OT2-TR003428-01S3, OT2-TR003428-01S2, OT2-TR003428-01; National Library of Medicine of the NIH under award number R01LM01372201; the National Science Foundation under award numbers: CAREER-1841569.

Author's Contributions

D.K. and C.M. conceived the project and supervised the study. C.M. processed the raw data and designed model framework, C.M. and Z.Z. wrote code and trained models. C.M, Z.Z. and D.K. drafted the manuscript. All authors read and approved the final manuscript.

Acknowledgements

The authors thank Dr. Jared Roach from Institute for Systems Biology for his assistance in manually evaluating the biological realism of the model-predicted paths in a double-blind manner, and also thank the RTX-KG2 team (Stephen Ramsey, Amy Glen, E. C. Wood, Lili Acevedo) for their guidance in building RTX-KG2 and in resolving any issues that arose.

References

1. Berdigaliyev N, Aljofan M. An overview of drug discovery and development. Future Medicinal Chemistry 2020;12(10):939–947.
2. Miller MT. Thalidomide embryopathy: a model for the study of congenital incomitant horizontal strabismus. Transactions of the American Ophthalmological Society 1991;89:623–74.
3. Verheul HMW, Panigrahy D, Yuan J, D'Amato RJ. Combination oral antiangiogenic therapy with thalidomide and sulindac inhibits tumour growth in rabbits. British Journal of Cancer 1999;79(1):114–118.
4. Singhal S, Mehta J, Desikan R, Ayers D, Roberson P, Eddlemon P, et al. Antitumor Activity of Thalidomide in Refractory Multiple Myeloma. The New England Journal of Medicine 1999;341(21):1565–1571.
5. Kairys V, Baranauskiene L, Kazlauskiene M, Matulis D, Kazlauskas E. Binding affinity in drug design: experimental and computational techniques. Expert Opinion on Drug Discovery 2019;14(8):755–768.
6. Aulner N, Danckaert A, Ihm J, Shum D, Shorte SL. Next-Generation Phenotypic Screening in Early Drug Discovery for Infectious Diseases. Trends in Parasitology 2019;35(7):559–570
7. Rusz CM, Ősz BE, Jîtcă G, Miklos A, Bătrînu MG, Imre S. Off-Label Medication: From a Simple Concept to Complex Practical Aspects. International Journal of Environmental Research and Public Health 2021;18(19):10447.
8. Swamidass SJ. Mining small-molecule screens to repurpose drugs. Briefings in Bioinformatics 2011;12(4):327–335.
9. Sanseau P, Agarwal P, Barnes MR, Pastinen T, Richards JB, Cardon LR, et al. Use of genome-wide association studies for drug repositioning. Nature Biotechnology 2012;30(4):317–320.
10. Bonner S, Barrett IP, Ye C, Swiers R, Engkvist O, Bender A, et al. A review of biomedical datasets relating to drug discovery:

a knowledge graph perspective. Briefings in Bioinformatics 2022 09;23(6):bbac404.
11. Wishart DS, Feunang YD, Guo AC, Lo EJ, Marcu A, Grant JR, et al. DrugBank 5.0: a major update to the DrugBank database for 2018. Nucleic Acids Research 2017;46(D1):gkx1037–.
12. Gaulton A, Bellis LJ, Bento AP, Chambers J, Davies M, Hersey A, et al. ChEMBL: a large-scale bioactivity database for drug discovery. Nucleic Acids Research 2012;40(D1):D1100–D1107.
13. Wishart DS, Feunang YD, Marcu A, Guo AC, Liang K, Vázquez-Fresno R, et al. HMDB 4.0: the human metabolome database for 2018. Nucleic Acids Research 2017;46(D1):gkx1089–.
14. Kanza S, Graham Frey J. Semantic Technologies in Drug Discovery. In: Wolkenhauer O, editor. Systems Medicine Oxford: Academic Press; 2021.p. 129–144.
15. Himmelstein DS, Lizee A, Hessler C, Brueggeman L, Chen SL, Hadley D, et al. Systematic integration of biomedical knowledge prioritizes drugs for repurposing. eLife 2017;6:e26726.
16. Walsh B, Mohamed SK, Nováček V. BioKG: A Knowledge Graph for Relational Learning On Biological Data. Proceedings of the 29th ACM International Conference on Information & Knowledge Management 2020;p. 3173–3180.
17. Su C, Hou Y, Guo W, Chaudhry F, Ghahramani G, Zhang H, et al. CBKH: The Cornell Biomedical Knowledge Hub. medRxiv 2021;.
18. Percha B, Altman RB. A global network of biomedical relationships derived from text. Bioinformatics 2018;34(15):2614–2624.
19. Zhang R, Hristovski D, Schutte D, Kastrin A, Fiszman M, Kilicoglu H. Drug repurposing for COVID-19 via knowledge graph completion. Journal of Biomedical Informatics 2021;115:103696.
20. Wang Q, Li M, Wang X, Parulian N, Han G, Ma J, et al. COVID-19 Literature Knowledge Graph Construction and Drug Repurposing Report Generation. Proceedings of the 2021 Conference of the North American Chapter of the Association for Computational Linguistics: Human Language Technologies: Demonstrations 2021;p. 66–77.
21. Li N, Yang Z, Luo L, Wang L, Zhang Y, Lin H, et al. KGHC: a knowledge graph for hepatocellular carcinoma. BMC Medical Informatics and Decision Making 2020;20(Suppl 3):135.
22. Santos A, Colaço AR, Nielsen AB, Niu L, Strauss M, Geyer PE, et al. A knowledge graph to interpret clinical proteomics data. Nature Biotechnology 2022;40(5):692–702.
23. Wood EC, Glen AK, Kvarfordt LG, Womack F, Acevedo L, Yoon TS, et al. RTX-KG2: a system for building a semantically standardized knowledge graph for translational biomedicine. BMC Bioinformatics 2022;23(1):400.
24. Ioannidis VN, Zheng D, Karypis G. Few-shot link prediction via graph neural networks for Covid-19 drug-repurposing. CoRR 2020;abs/2007.10261.
25. Bordes A, Usunier N, Garcia-Duran A, Weston J, Yakhnenko O. Translating Embeddings for Modeling Multi-relational Data. In: Neural Information Processing Systems (NIPS) South Lake Tahoe, United States; 2013. p. 1–9.
26. Sun Z, Deng ZH, Nie JY, Tang J. RotatE: Knowledge Graph Embedding by Relational Rotation in Complex Space. 2019. arXiv:1902.10197.
27. Yang B, Yih Wt, He X, Gao J, Deng L. Embedding Entities and Relations for Learning and Inference in Knowledge Bases. 2014. arXiv:1412.6575.
28. Trouillon T, Welbl J, Riedel S, Gaussier E, Bouchard G. Complex Embeddings for Simple Link Prediction. 2016. arXiv:1606.06357.
29. Wang B, Shen T, Long G, Zhou T, Wang Y, Chang Y. Structure-Augmented Text Representation Learning for Efficient Knowledge Graph Completion. Proceedings of the Web Conference 2021 2021;p. 1737–1748.
30. Sosa DN, Derry A, Guo M, Wei E, Brinton C, Altman RB. A Literature-Based Knowledge Graph Embedding Method for Identifying Drug Repurposing Opportunities in Rare Diseases Pacific Symposium on Biocomputing Pacific Symposium on Biocomputing 2020;25:463–474.
31. Chen X, Chen M, Shi W, Sun Y, Zaniolo C. Embedding Uncertain Knowledge Graphs. Proceedings of the AAAI Conference on Artificial Intelligence 2019;33:3363–3370.
32. Sang S, Yang Z, Liu X, Wang L, Lin H, Wang J, et al. GrEDeL: A Knowledge Graph Embedding Based Method for Drug Discovery From Biomedical Literatures. IEEE Access 2019;7:8404–8415.
33. Kilicoglu H, Rosemblat G, Fiszman M, Shin D. Broad-coverage biomedical relation extraction with SemRep. BMC Bioinformatics 2020;21(1).
34. Li Y. Reinforcement Learning Applications. 2019. arXiv:1908.06973.
35. Chen L, Cui J, Tang X, Qian Y, Li Y, Zhang Y. RLPath: a knowledge graph link prediction method using reinforcement learning based attentive relation path searching and representation learning. Applied Intelligence 2022;52(4):4715–4726.
36. Sun Y, Wang S, Tang X, Hsieh TY, Honavar V. Adversarial Attacks on Graph Neural Networks via Node Injections: A Hierarchical Reinforcement Learning Approach. Proceedings of The Web Conference 2020 2020;p. 673–683.
37. Zhou X, Wang P, Luo Q, Pan Z. Multi-hop Knowledge Graph Reasoning Based on Hyperbolic Knowledge Graph Embedding and Reinforcement Learning. The 10th International Joint Conference on Knowledge Graphs 2021;p. 1–9.
38. Liu Y, Hildebrandt M, Joblin M, Ringsquandl M, Raissouni R, Tresp V. Neural Multi-hop Reasoning with Logical Rules on Biomedical Knowledge Graphs. In: Verborgh R, Hose K, Paulheim H, Champin PA, Maleshkova M, Corcho O, et al., editors. The Semantic Web Cham: Springer International Publishing; 2021. p. 375–391.
39. Womack F, McClelland J, Koslicki D. Leveraging Distributed Biomedical Knowledge Sources to Discover Novel Uses for Known Drugs. bioRxiv 2019;p. 765305.
40. Hamilton WL, Ying R, Leskovec J. Inductive Representation Learning on Large Graphs. 2017. arXiv:1706.02216.
41. Zhao K, Wang X, Zhang Y, Zhao L, Liu Z, Xing C, et al. Leveraging Demonstrations for Reinforcement Recommendation Reasoning over Knowledge Graphs. Proceedings of the 43rd International ACM SIGIR Conference on Research and Development in Information Retrieval 2020;p. 239–248.
42. Mayers M, Steinecke D, Su AI, Database of mechanism of action paths for selected drug-disease indications. Zenodo; 2020. https://doi.org/10.5281/zenodo.3708278.
43. Mayers M, Tu R, Steinecke D, Li TS, Queralt-Rosinach N, Su AI. Design and application of a knowledge network for automatic prioritization of drug mechanisms. Bioinformatics 2022;38(10):btac205.
44. Degtyarenko K, Matos Pd, Ennis M, Hastings J, Zbinden M, McNaught A, et al. ChEBI: a database and ontology for chemical entities of biological interest. Nucleic Acids Research 2008;36(Database issue):D344–D350.
45. Consortium TBDT. Toward A Universal Biomedical Data Translator. Clinical and Translational Science 2019;12(2):86–90.
46. Translator Consortium. The Biomedical Data Translator Program: Conception, Culture, and Community. Clinical and Translational Science 2019 Mar;12(2):91–94.
47. Unni DR, Moxon SA, Bada M, Brush M, Bruskiewich R, Caufield JH, et al. Biolink Model: A universal schema for knowledge graphs in clinical, biomedical, and translational science. Clinical and Translational Science 2022;.
48. Xin J, Afrasiabi C, Lelong S, Adesara J, Tsueng G, Su AI, et al. Cross-linking BioThings APIs through JSON-LD to facilitate knowledge exploration. BMC Bioinformatics 2018;19(1):30.
49. Xin J, Mark A, Afrasiabi C, Tsueng G, Juchler M, Gopal N, et al. High-performance web services for querying gene and variant

annotation. Genome Biology 2016;17(1):91.
50. Kilicoglu H, Shin D, Fiszman M, Rosemblat G, Rindflesch TC. SemMedDB: a PubMed-scale repository of biomedical semantic predications. Bioinformatics 2012;28(23):3158–3160
51. Brown SH, Elkin PL, Rosenbloom ST, Husser C, Bauer BA, Lincoln MJ, et al. VA National Drug File Reference Terminology: a cross-institutional content coverage study. Studies in health technology and informatics 2004;107(Pt 1):477–81.
52. Brown AS, Patel CJ. A standard database for drug repositioning. Scientific Data 2017;4(1):170029.
53. Cilibrasi RL, Vitanyi PMB. The Google Similarity Distance. IEEE Transactions on Knowledge and Data Engineering 2007;19(3):370–383.
54. Gu Y, Tinn R, Cheng H, Lucas M, Usuyama N, Liu X, et al. Domain-Specific Language Model Pretraining for Biomedical Natural Language Processing. ACM Transactions on Computing for Healthcare 2022;3(1):1–23.
55. Mikolov T, Sutskever I, Chen K, Corrado G, Dean J. Distributed Representations of Words and Phrases and their Compositionality. Advances in neural information processing systems 2013;26.
56. Lillicrap TP, Hunt JJ, Pritzel A, Heess N, Erez T, Tassa Y, et al. Continuous control with deep reinforcement learning. 2015. arXiv:1509.02971.
57. Sutton RS. Learning to predict by the methods of temporal differences. Machine Learning 1988;3(1):9–44.
58. Williams RJ. Simple Statistical Gradient-Following Algorithms for Connectionist Reinforcement Learning. Machine Learning 1992;8(3-4):229–256.
59. Pomerleau DA. Efficient Training of Artificial Neural Networks for Autonomous Navigation. Neural Computation 1991;3(1):88–97.
60. Hsieh K, Wang Y, Chen L, Zhao Z, Savitz S, Jiang X, et al. Drug repurposing for COVID-19 using graph neural network and harmonizing multiple evidence. Scientific reports 2021;11(1):23179
61. Lin Y, Liu Z, Sun M, Liu Y, Zhu X. Learning entity and relation embeddings for knowledge graph completion. Proceedings of the AAAI conference on artificial intelligence 2015;29(1). https://doi.org/10.1609/aaai.v29i1.9491.
62. Liu H, Wu Y, Yang Y. Analogical inference for multi-relational embeddings. In: International conference on machine learning; 2017. p. 2168–2178.
63. Kazemi SM, Poole D. SimplE Embedding for Link Prediction in Knowledge Graphs. Advances in Neural Information Processing Systems 2018;31:4284-4295.
64. Veličković P, Cucurull G, Casanova A, Romero A, Liò P, Bengio Y, Graph Attention Networks. 2017. arXiv:1710.10903.
65. Glorot X, Bengio Y. Understanding the difficulty of training deep feedforward neural networks. In: Teh YW, Titterington DM, editors. AISTATS, vol. 9 of JMLR Proceedings JMLR.org; 2010. p. 249–256.
66. Lin XV, Socher R, Xiong C. Multi-Hop Knowledge Graph Reasoning with Reward Shaping. Proceedings of the 2018 Conference on Empirical Methods in Natural Language Processing 2018;p. 3243–3253.
67. Croom KF, McCormack PL. Recombinant factor VIIa (eptacog alfa): a review of its use in congenital hemophilia with inhibitors, acquired hemophilia, and other congenital bleeding disorders. BioDrugs : clinical immunotherapeutics, biopharmaceuticals and gene therapy 2008;22(2):121–36.
68. Minno GD. Eptacog alfa activated: a recombinant product to treat rare congenital bleeding disorders. Blood Reviews 2015;29:S26–S33.
69. Rendo P, Smith L, Lee HY, Shafer F. Nonacog alfa: an analysis of safety data from six prospective clinical studies in different patient populations with haemophilia B treated with different therapeutic modalities. Blood coagulation & fibrinolysis : an international journal in haemostasis and thrombosis 2015;26(8):912–8.
70. Driessche T, Collen D, Chuah M. Viral Vector-Mediated Gene Therapy for Hemophilia. Current Gene Therapy 2001;1(3):301–315.
71. Roberts HR, Monroe DM, White GC. The use of recombinant factor VIIa in the treatment of bleeding disorders. Blood 2004;104(13):3858–3864.
72. Negrier C, Shima M, Hoffman M. The central role of thrombin in bleeding disorders. Blood Reviews 2019;38:100582.
73. Goodeve AC. Hemophilia B: molecular pathogenesis and mutation analysis. Journal of Thrombosis and Haemostasis 2015;13(7):1184–1195.
74. Sourimant J, Aggarwal M, Plemper RK. Progress and pitfalls of a year of drug repurposing screens against COVID-19. Current Opinion in Virology 2021;49:183–193.
75. Gysi DM, do Valle Í , Zitnik M, Ameli A, Gan X, Varol O, et al. Network medicine framework for identifying drug-repurposing opportunities for COVID-19. Proceedings of the National Academy of Sciences of the United States of America 2021;118(19):e2025581118.
76. Yohn CN, Gergues MM, Samuels BA. The role of 5-HT receptors in depression. Molecular Brain 2017;10(1):28.
77. Delva NC, Stanwood GD. Dysregulation of brain dopamine systems in major depressive disorder. Experimental Biology and Medicine 2021;246(9):1084–1093.
78. Coppen EM, Roos RAC. Current Pharmacological Approaches to Reduce Chorea in Huntington's Disease. Drugs 2017;77(1):29–46.
79. Shukla S, Tekwani BL. Histone Deacetylases Inhibitors in Neurodegenerative Diseases, Neuroprotection and Neuronal Differentiation. Frontiers in Pharmacology 2020;11:537.
80. Yu IT, Park JY, Kim SH, Lee Js, Kim YS, Son H. Valproic acid promotes neuronal differentiation by induction of proneural factors in association with H4 acetylation. Neuropharmacology 2009;56(2):473–480.
81. Singh S, Singh TG. Role of Nuclear Factor Kappa B (NF-KB) Signalling in Neurodegenerative Diseases: An Mechanistic Approach. Current Neuropharmacology 2020;18(10):918–935.
82. Yagami T, Kohma H, Yamamoto Y. L-type voltage-dependent calcium channels as therapeutic targets for neurodegenerative diseases. Current medicinal chemistry 2012;19(28):4816–27.
83. Noh W, Pak S, Choi G, Yang S, Yang S. Transient Potassium Channels: Therapeutic Targets for Brain Disorders. Frontiers in Cellular Neuroscience 2019;13:265.
84. Arena R, Iudice A, Virgili P, Moretti P, Menchetti G. Huntington's disease: clinical effects of a short-term treatment with pimozide. Advances in biochemical psychopharmacology 1980;24:573–5.
85. Videnovic A. Treatment of huntington disease. Current treatment options in neurology 2013;15(4):424–38.
86. Paleacu D, Anca M, Giladi N. Olanzapine in Huntington's disease: Olanzapine in Huntington's disease. Acta Neurologica Scandinavica 2002;105(6):441–444.
87. Squitieri F, Cannella M, Porcellini A, Brusa L, Simonelli M, Ruggieri S. Short-term effects of olanzapine in Huntington disease. Neuropsychiatry, neuropsychology, and behavioralneurology 2001;14(1):69–72.
88. Group HS. Dosage effects of riluzole in Huntington's disease: a multicenter placebo-controlled study. Neurology 2003;61(11):1551–6.
89. Unti E, Mazzucchi S, Palermo G, Bonuccelli U, Ceravolo R. Antipsychotic drugs in Huntington's disease. Expert Review of Neurotherapeutics 2017;17(3):227–237.
90. Duff K, Beglinger LJ, O'Rourke ME, Nopoulos P, Paulson HL, Paulsen JS. Risperidone and the Treatment of Psychiatric, Motor, and Cognitive Symptoms in Huntington's Disease. Annals of Clinical Psychiatry 2008;20(1):1–3.
91. Miranda AS, Cardozo PL, Silva FR, Souza JMd, Olmo IG, Cruz

JS, et al. Alterations of Calcium Channels in a Mouse Model of Huntington's Disease and Neuroprotection by Blockage of CaV1 Channels. ASN NEURO 2019;11:1759091419856811.
92. Ma C, Zhou Z, Liu H, Koslicki D. Relevant Datasets and Software Used for Paper "KGML-xDTD: A Knowledge Graph-based Machine Learning Framework for Drug Treatment Prediction and Mechanism Description (1.0.0) [Data set]. Zenodo 2023; https://doi.org/10.5281/zenodo.7582233.
93. Ma C, Zhou Z, Liu H, Koslicki D. Supporting data for KGML-xDTD: A Knowledge Graph-based Machine Learning Framework for Drug Treatment Prediction and Mechanism Description. GigaScience Database 2023; http://dx.doi.org/10.5524/102404.
94. Molecular Data Provider Team. https://github.com/NCATSTranslator/Translator-All/wiki/Molecular-Data-Provider. Accessed 09 November 2021.

Click here to access/download;Figure;suppl_figure1.jpg

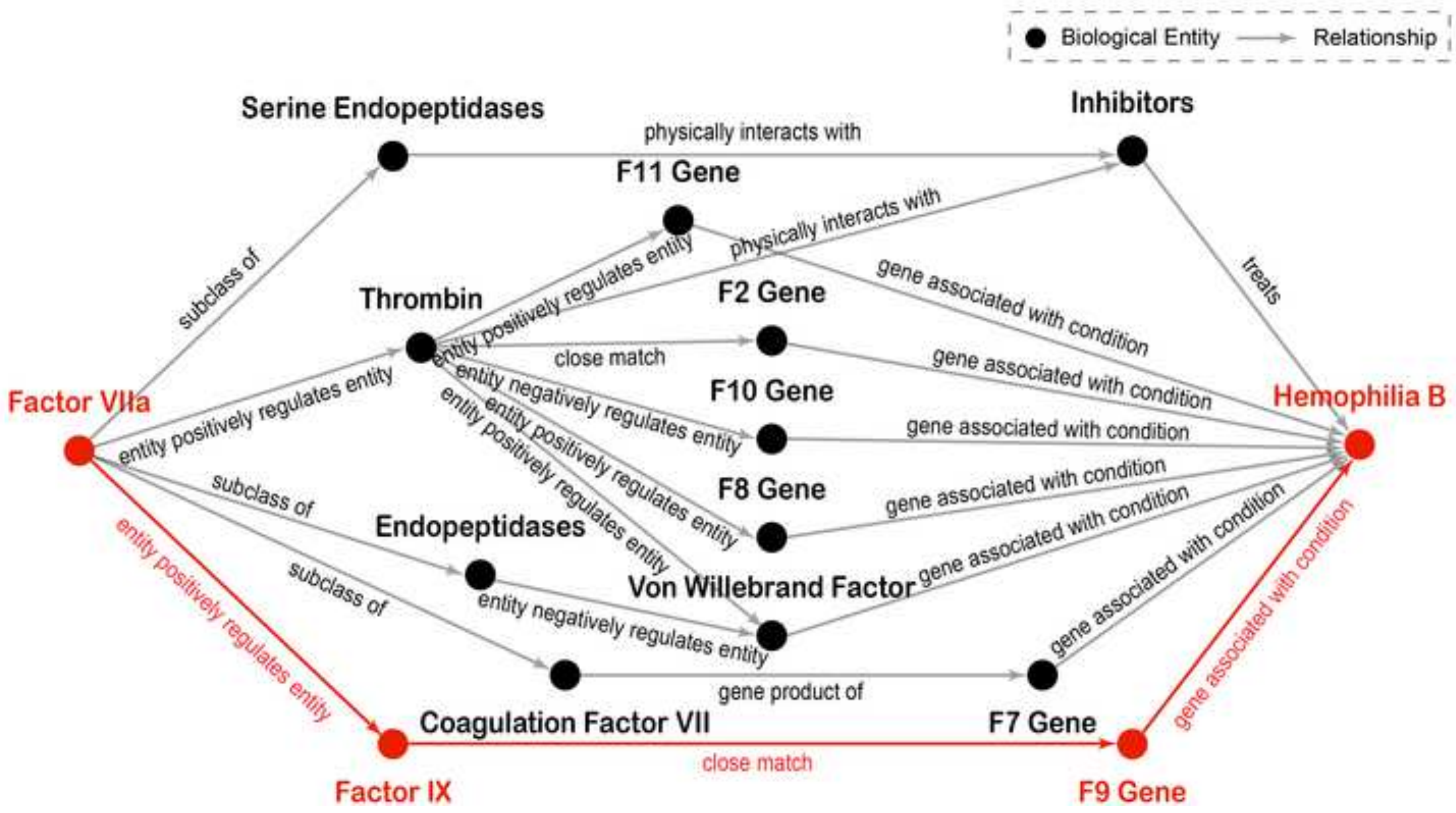

 Click here to access/download;Figure;main_figure2.jpg

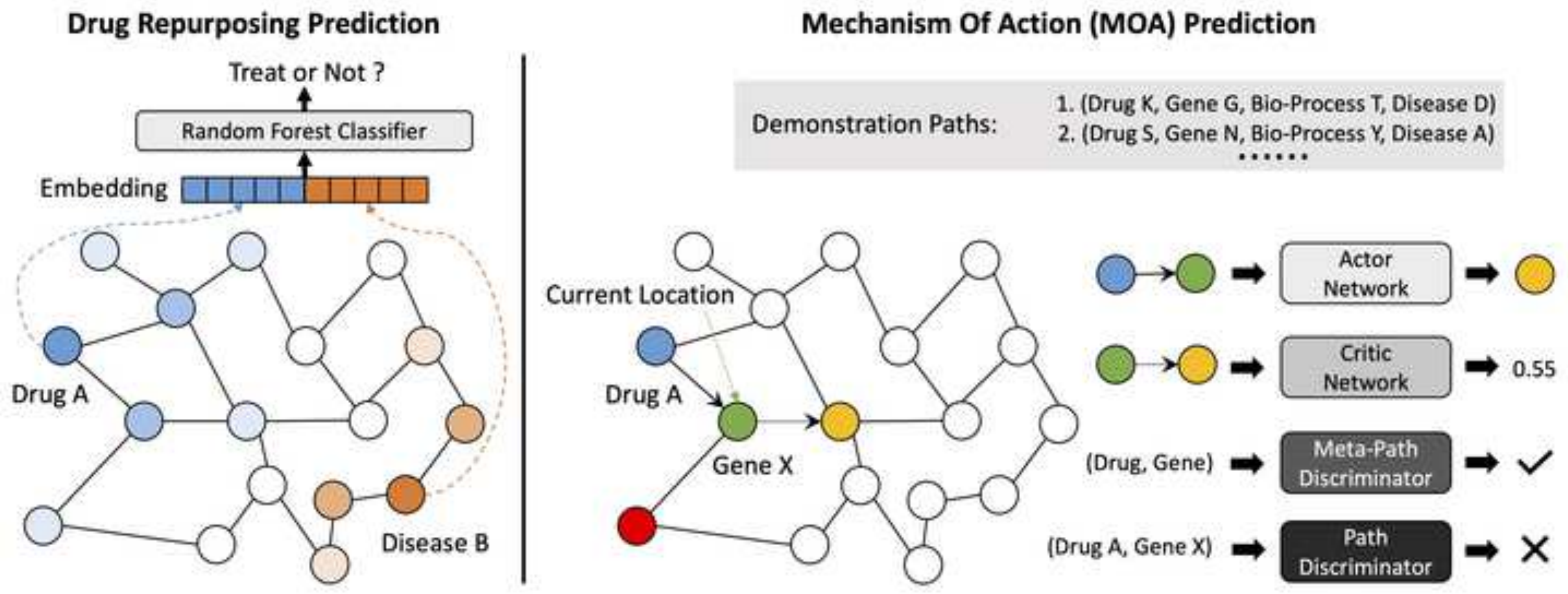

Figure3 in Main Text

Click here to access/download;Figure;main_figure3.jpg

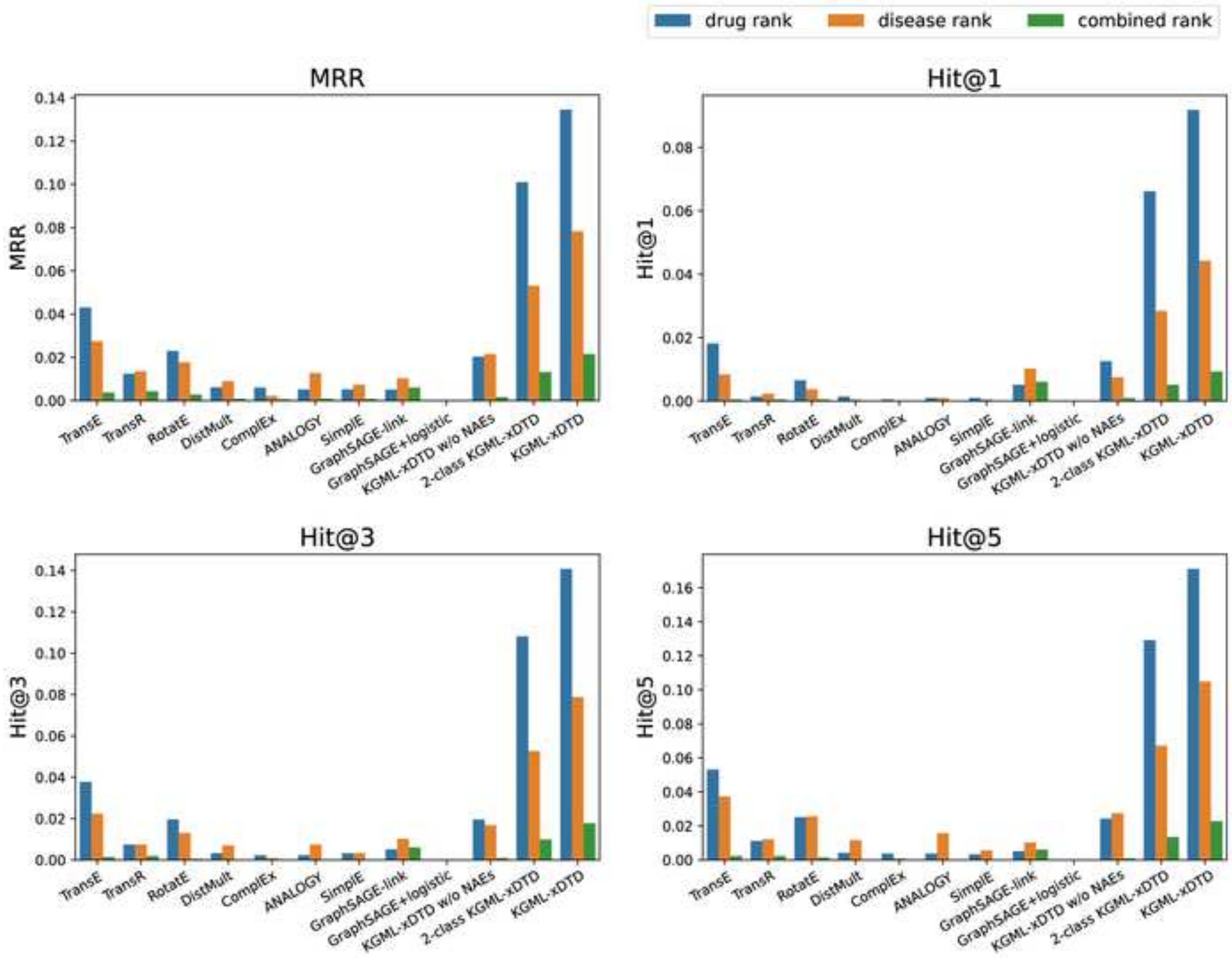

 Click here to access/download;Figure;main_figure5.jpg

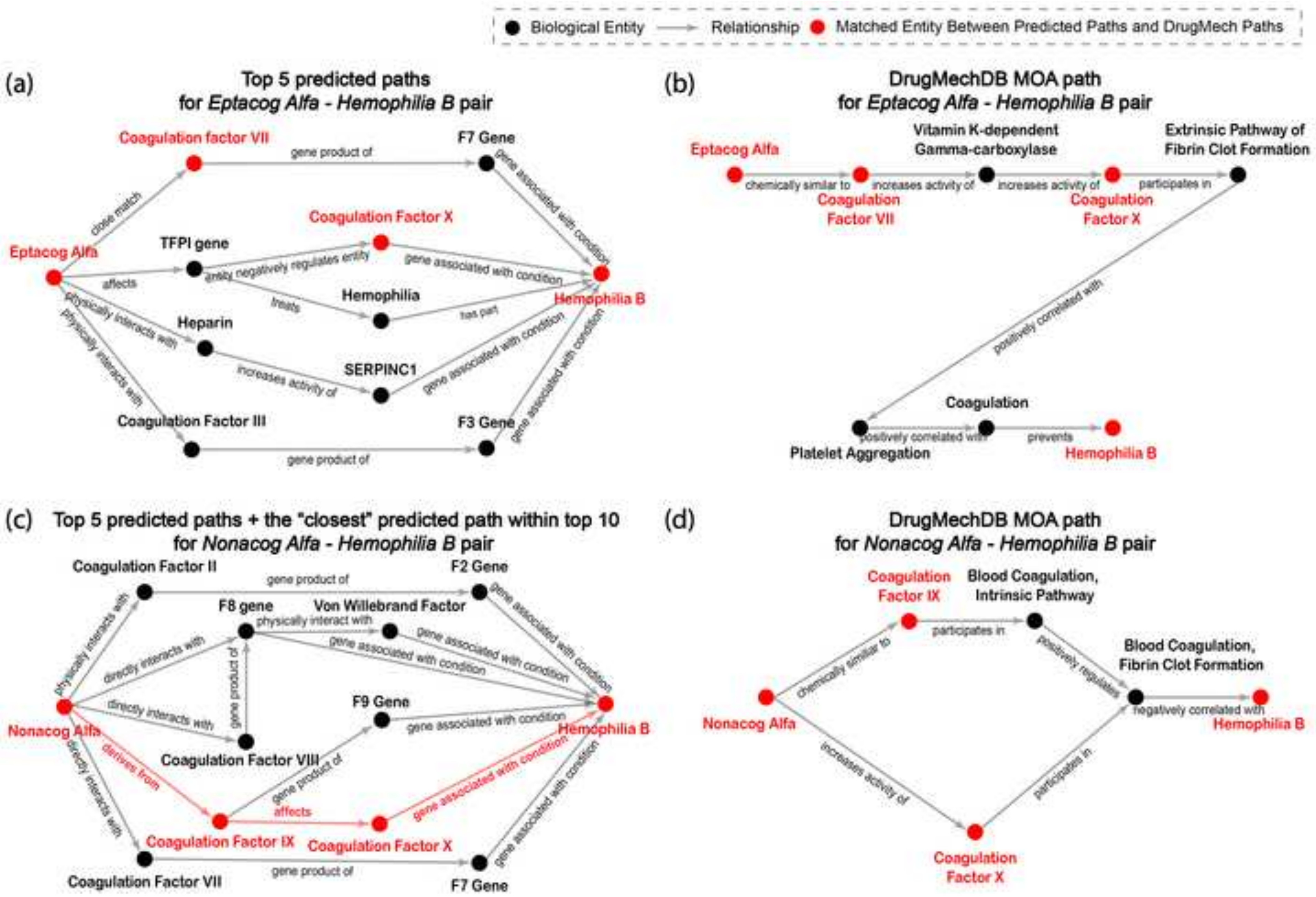

Click here to access/download;Figure;main_figure6.jpg

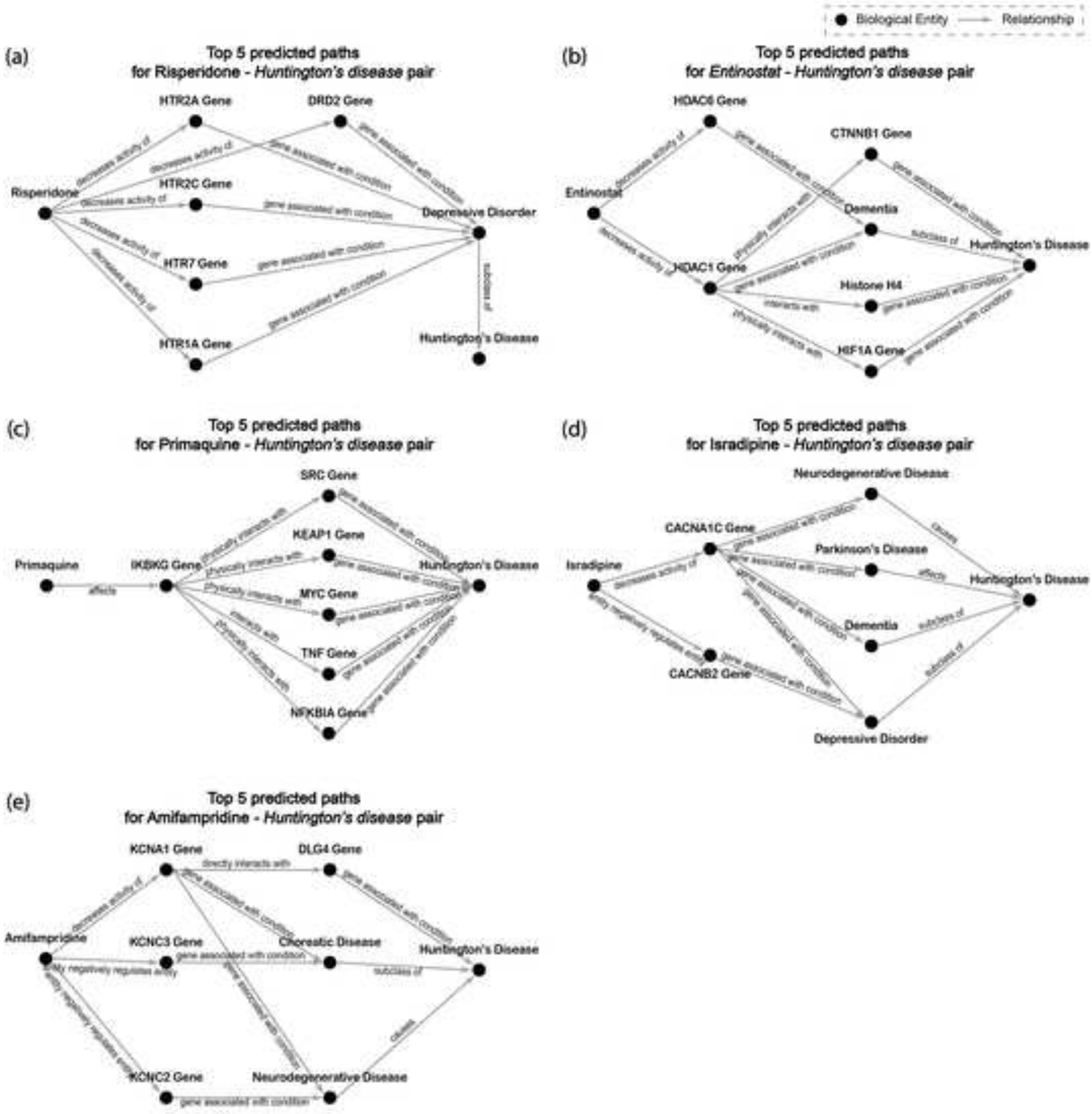

Click here to access/download;Figure;main_figure7.jpg

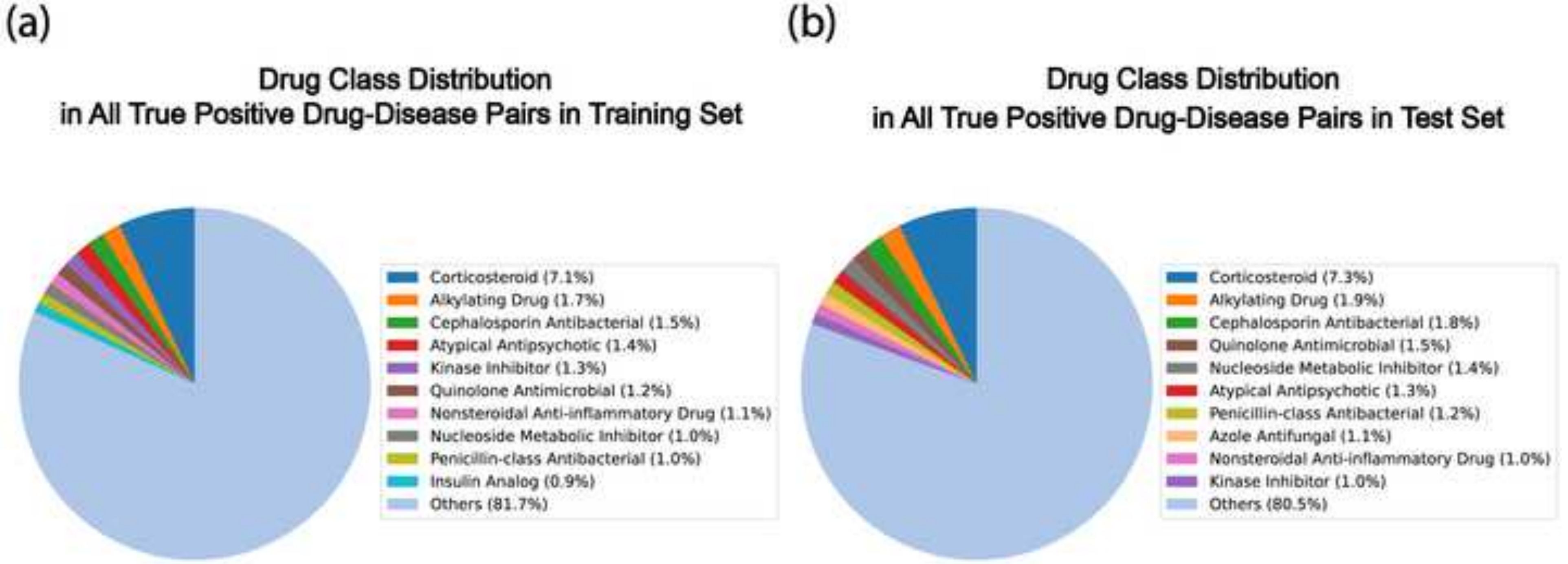

Figure8 in Main Text

Click here to access/download;Figure;main_figure8.jpg

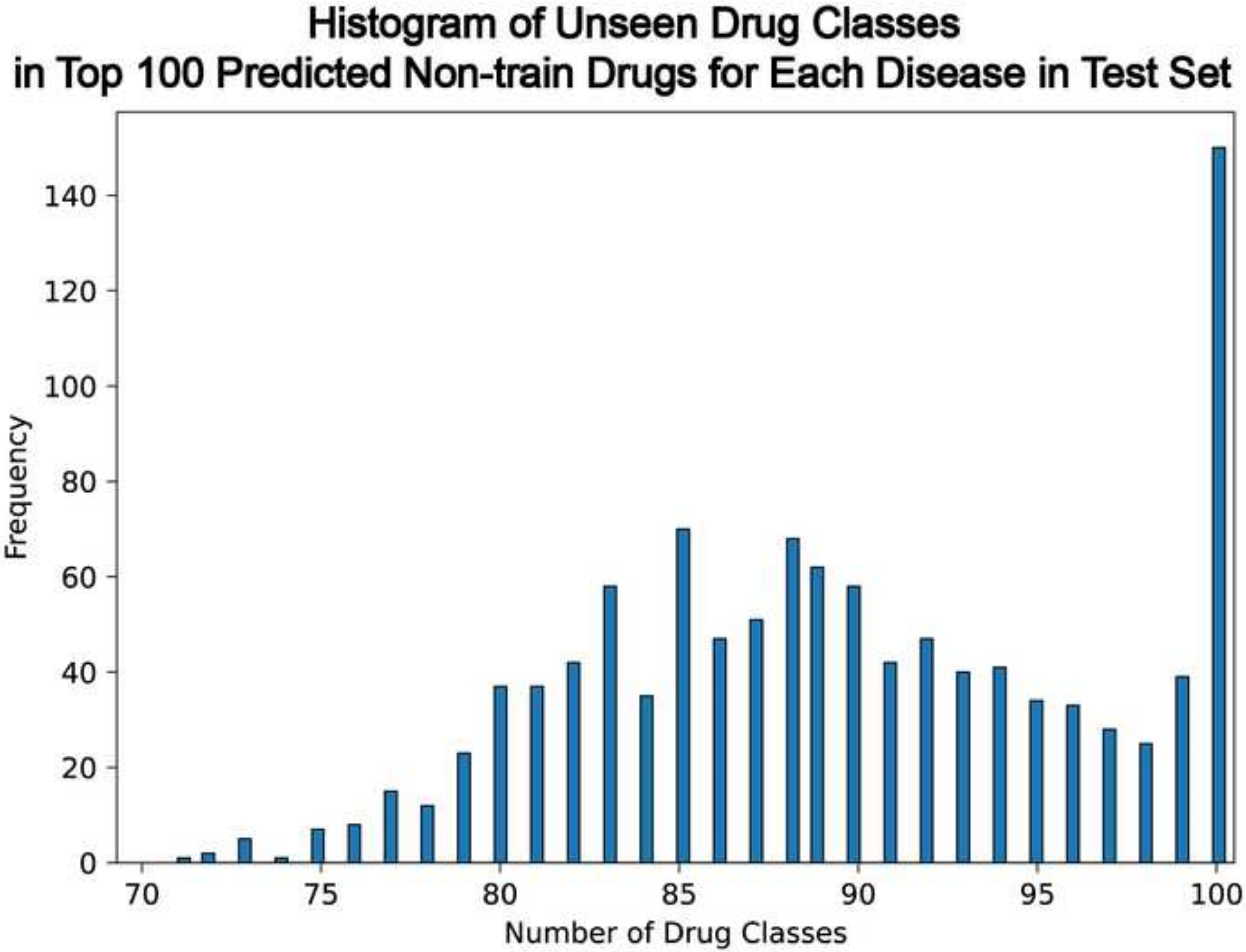

Click here to access/download
Supplementary Material
KGML_xDTD_GigaScience_supplementary_revision2.pdf
