# Supplementary material for: KGML-xDTD: a knowledge graph–based machine learning framework for drug treatment prediction and mechanism description
Source: Gigascience. 2023 Aug 21;12:giad057. doi: 10.1093/gigascience/giad057 (PMC10441000; doi:10.1093/gigascience/giad057)
Supplement: giad057_Supplemental_Files [file giad057_supplemental_files.zip › KGML_xDTD_GigaScience_supplementary_revision2.pdf]

Supplemental Information

KGML-xDTD: A Knowledge Graph-based Machine Learning Framework for Drug Treatment Prediction and Mechanism Description

Chunyu Ma[1,*], Zhihan Zhou[2], Han Liu[2], and David Koslicki[1,3,4,*]

[1] *Huck Institutes of Life Sciences, Pennsylvania State University, State College, PA 16801, USA*
[2] *Department of Computer Science, Northwestern University, Evanston, IL 60208, USA*
[3] *Department of Computer Science and Engineering, Pennsylvania State University, State College, PA 16801, USA*
[4] *Department of Biology, Pennsylvania State University, State College, PA 16801, USA*

[*] *Correspondence address: Chunyu Ma, E-mail:cqm5886@psu.edu; David Koslicki, E-mail: dmk333@psu.edu*

Section S1. Biomedical Knowledge Graph RTX-KG2c Pre-processing

The dataset of *RTX-KG2c* (v2.7.3) (Wood et al., 2022) is accessed via https://github.com/RTXteam/RTX-KG2. We pre-process the raw data of *RTX-KG2c* by the following four principles:

1. Since we are mainly interested in the categories relevant to drug mechanisms of action (MOAs), we exclude the nodes with categories that are not expected to be useful for drug repurposing explanation (e.g., "GeographicaLocation", "Device", "InformationResource").

2. One of data sources used in the *RTX-KG2c* is the Semantic MEDLINE Database (SemMedDB) (Kilicoglu et al., 2012), one of the most widely used NLP-derived biomedical knowledge sources, that has been found recently (Cong et al., 2018) to contain some inconsistent relations due to the immature NLP techniques even though it contains many latest-found relations (e.g., the relations with Covid-19). To improve the quality of SemMedDB-based edges, we filter out some edges based on the following criteria:

   - Each remaining SemMedDB-based edge must be supported by at least 10 publications.
   - The PubMed-publication-based NGD score (Cilibrasi and Vitanyi, 2007) (defined in Equation 1 in the main text) of the two end nodes should be higher than 0.6.

3. The *RTX-KG2c* is a multigraph that allows multiple edges to connect between two nodes. These edges present different relations (a.k.a. predicates) between the nodes, and follow the predicate hierarchy [1] used in the Biolink model (Unni et al., 2022). As a result, the *RTX-KG2c* contains some hierarchically redundant edges between two nodes. To simplify the process of path finding for the downstream MOA predictions, we only reserve the "leaf" predicates (i.e., the most specific predicates) in the Biolink semantic relation hierarchy if there are hierarchically associated edges between two nodes. For example, if there are edges between two nodes representing the predicates "affected by", "entity regulated by entity" and "entity positively regulated by entity", we would remove the "affected by" and "entity regulated by entity" edges because they are more general (i.e., "ancestor") predicates in the hierarchy compared to "entity positively regulated by entity". Removing those more general predicates does not affect the interpretability of the paths because the "leaf" predicates contain more precise semantic information.

4. To avoid the training information leakage, we exclude all existing edges that directly connect the potential drug nodes (nodes with categories "Drug" or "SmallMolecule") to potential disease nodes (nodes with categories "Disease", "PhenotypicFeature", "BehavioralFeature" or "DiseaseOrPhenotypicFeature") in the *RTX-KG2c*.

After applying the pre-processing steps to *RTX-KG2c* (v2.7.3) described above, the post-processed/customized biomedical knowledge graph (BKG) consists of 3,659,165 nodes with 33 distinct categories, and 18,291,237 edges with 74 distinct types.

[1] Visualization of hierarchical predicates in the Biolink Model v2.1.0 `http://tree-viz-biolink.herokuapp.com/predicates/2.1.0`

Section S2. Summary of Data Resources used by MyChem Data

In Table 1, we summarize 11 data resources from which the MyChem Data (Xin et al., 2018) collected the up-to-date chemical annotation data. The table includes information such as source name (e.g., source), version, number of chemicals (e.g., # of chemicals), publication or link to original resource (e.g., publication/link), and quality of data (e.g., quality). For more detailed information about each specific data resource, please refer to its respective publication or original resource.

Table 1: 11 Data Resources Used by MyChem Data.

Source [a]	Version [a]	# of chemicals [a]	Publication/Link	Quality
AEOLUS		3,044	(Banda et al., 2016)	human curated
ChEBI	rel218	162,676	(Degtyarenko et al., 2008)	human curated
ChEMBL	ChEMBL_31	2,331,591	(Gaulton et al., 2012)	human curated
DrugCentral		5,399	(Ursu et al., 2016)	human curated
FDA Orphan Drug Designations	See Note [b]	3,661	See Note [c]	FDA-approved
ginas		58,468	(Peryea et al., 2020)	human curated
NDC	2023-02-10	47,596	See Note [d]	FDA-approved
PharmGKB	2023-02-05	3,532	(Thorn et al., 2013)	human curated
PubChem	2023-02-02	113,609,246	(Kim et al., 2016)	NIH-approved
SIDER	2015-10-21	1,507	(Kuhn et al., 2015)	human curated
UNII	2023-01-19	137,795	See Note [e]	FDA-approved

[a] The information of these columns is from https://docs.mychem.info/en/latest/doc/data.html.
[b] dc05824f916d1099dbb74145656308230290cff7f62b233fe9fb51fbca955a34.
[c] https://www.accessdata.fda.gov/scripts/opdlisting/oopd/.
[d] https://www.fda.gov/drugs/drug-approvals-and-databases/national-drug-code-directory.
[e] https://www.fda.gov/industry/fda-data-standards-advisory-board/fdas-global-substance-registration-system.

Section S3. Implementation Details of `KGML-xDTD` Model Framework

0.1 Drug Repurposing Prediction (DRP) Module

In the drug repurposing prediction module, we utilize the source code [2] provided by Hamilton et al. (2017) to train unsupervised GraphSAGE embeddings with its "big" mean-based aggregator and two hidden layers of dimensions [256, 256]. For the random walk setting, we performed 10 walks each with length of 100. As for other parameters, the number of epochs is set to 10, the neighbor sampling size of each layer is 96, the learning rate is 0.001, the batch size is 256, and the maximum number of iterations per epoch is 10,000. Instead of using the default identity embeddings as the initial features, we use node attribute embeddings generated by the pre-trained PubMedBert model (Gu et al., 2022) with concatenation of the node's name and category, and then reduce their dimensions to 100 using Principal Component Analysis (PCA). The final output output embedding vector for each node has a dimension of 512. With these GraphSAGE embedding vectors, we concatenate the embedding vectors of each drug-disease pair in the training set as input features and use the *RandomForestClassifier* function of scikit-learn (v1.0) python package to train a Random Forest model. We run a grid search using the *GridSearchCV* function to determine the optimal parameter set for the Random Forest model from a range of depths {5, 10, 15, 20, 25, 30, 35} and number of trees {500, 1000, 1500, 2000}. The best parameter set for the Random Forest model uses the maximum depth $max\_depth = 35$ and the number of trees $n\_estimators = 2000$.

0.2 Mechanism of Action (MOA) Prediction Module

In the mechanism of action prediction module, we design a reinforcement learning model following the model framework of Zhao et al. (2020) for drug repurposing purpose (see Sec. "Mechanism of Action (MOA) Prediction" in the main text). We set the state history length $K = 2$ and the maximum length of path $T = 3$. In order to make the customized biomedical knowledge graph (described in Section S1 on page 1 above) enable training on a 48GB Quadro RTX 8000 GPU, we prune the action space of each node to a maximum size of 3,000 based on the PageRank score (Page et al., 1999) (calculated by the *pagerank* function of NetworkX (v2.7.1) python package). We set the dimensions of all lookup matrices used within actor, critic, meta-path discriminator, and path discriminator

[2] `https://github.com/williamleif/GraphSAGE`

networks to 100. The dimensions of hidden layers of the actor network and critic network are both set to 512. We set the dimensions of hidden layers of the path discriminator with [512, 512] and used the dimension set of [512, 256] for the hidden layers of the meta-path discriminator. We use Xavier initialization (Glorot and Bengio, 2010) for the embeddings of all lookup matrices and the network layers. The weight of the path discriminator reward α_p is set to 0.006 while the meta-factor of the path discriminator reward $a_m = 0.012$. We respectively assign 0.99 and 0.005 to the decaying coefficient γ of $R_{e,T}$ and the weight α of entropy term. We optimize all networks using the Adam optimization algorithm (Kingma and Ba, 2015) with a learning rate of 0.0005. The mini-batch size is set to 32 with a path rollout of 35. The dropout rates of all subnetworks are set to 0.3 and the action dropout rate is set to 0.5.

Section S4. Implementation Details of Demonstration Path Extraction

The demonstration paths are a set of multi-hop BKG-based paths that can be used to guide the agent in the reinforcement learning model of `KGML-xDTD` model framework to find biologically reasonable BKG-based MOA paths. It can be formulated as $P^k = \{p^k_{s,t} | v_s \in \mathcal{V}^{\text{drug}}; v_t \in \mathcal{V}^{\text{disease}}\}$ where $p^k_{s,t}$ is a multi-hop demonstration path with maximum path length k starting from a drug node v_s and ending at a disease node v_t. Given a potential drug node and a potential disease node (defined in Section S1 on page 1 above), the number of paths in the customized biomedical knowledge graph between them grows exponentially as the value of k increases. Therefore, we set $k = 3$ to guarantee that the agent can find biologically meaningful MOA paths in a reasonable amount of time. To extract reasonable demonstration paths from the customized biomedical knowledge graph, we use the known drug-target interactions collected from two curated biomedical data sources (e.g., DrugBank (v5.1) and Molecular Data Provider (v1.2)[3]), as well as the PubMed-publication-based Normalized Google Distance (NGD) (see Equation 1 in the main text). A demonstration path extracted from the customized biomedical knowledge graph must satisfy the following two requirements:

1. The edge connecting the drug node to the first intermediate node in a demonstration path must be supported by DrugBank or Molecular Data Provider as a known drug-target interaction, and must also have a Normalized Google Distance (NGD) score of 0.6 or lower.

2. The edge connecting the second intermediate node to the disease node in a demonstration path should have a Normalized Google Distance (NGD) score of 0.6 or lower.

Only the true positive pairs (see Table 1 in the main text) collected from four human-curated and NLP-derived training datasets (described in Sec. "Data Sources for Model Training" in the main text) are used to extract demonstration paths. We also filter out any true positive pairs that are not reachable from a drug node to its corresponding disease node within maximum of 3 hops in the customized biomedical knowledge graph. Out of these 21,437 true positive pairs, 8,495 are able to find at least one demonstration path that meet the requirement. We finally find 396,705 demonstration paths for 8,495 true positive drug-disease pairs which are used for reinforcement learning training.

Section S5. Implementation Details of Baseline Models

We use the OpenKE library [4] to implement the models `TransE`, `TransR`, `RotatE`, `DistMult`, `ComplEx`, `ANALOGY`, and `SimplE` with their default parameter settings (we adjusted the hyperparameters for some models to ensure we can finish the training on GPUs for a reasonable time). Table 2 shows the detailed hyperparameter setting of these baseline model. For other baseline models, we use the PyTorch Geometric [5] framework to implement the `GAT` and `GraphSAGE-link` model and use the same GraphSAGE embeddings (mentioned above) with scikit-learn (v1.0) python package to implement the `GraphSAGE+logistic`, `GraphSAGE+SVM` and `2-class GraphSAGE+RF` models with the grid-search-based optimal parameter settings.

For the implementation of the `MultiHop` model, we use the source code[6] provided by Lin et al. (2018) and modify its reward function by using our defined reward shaping strategy (described in Sec. "Adversarial Actor-critic Reinforcement Learning" in the main text). We set all its parameters the same as the reinforcement learning model in the `KGML-xDTD` model framework if they are available otherwise we use the default parameters.

[3] https://github.com/NCATSTranslator/Translator-All/wiki/Molecular-Data-Provider
[4] https://github.com/thunlp/OpenKE
[5] https://github.com/pyg-team/pytorch_geometric
[6] https://github.com/salesforce/MultiHopKG

Table 2: Hyperparameters Used for Baseline Models.

Model	Hidden Dim.	Num. Epochs	Batch Size	Learning Rate	Optimizer
TransE	100	10000	1000	1	SGD
TransR	50	2000	1000	1	SGD
RotatE	30	2000	1000	2e-5	Adam
DistMult	100	10000	1000	0.5	Adagrad
ComplEx	50	2000	500	0.5	Adagrad
ANALOGY	20	2000	500	0.5	Adagrad
SimpLE	100	2000	500	0.5	Adagrad

Section S6. Top 10 KGML-xDTD's Predicted Paths Serving as Biological Explanations for the Predicted "Treats" Relationship between Factor VIIa and Hemophilia B

We utilize the KGML-xDTD model framework to predict the top 10 3-hop BKG-based paths, serving as biological explanations of the predicted "treats" relationship between Factor VIIa and Hemophilia B (shown in Table 4 for case study 1 in the main text). This particular drug/treatment - disease pair is not used in the training set and thus can be used to indicate how KGML-xDTD's MOA path predictions can contribute to the explanation of the predicted drug repurposing results. The predicted paths show similar molecular details as those in Figure 4 in the main text for treating hemophilia B. The path that we highlighted in red is the one in which all nodes can align with the key molecules in the real drug action regulatory network shown in Figure 4.

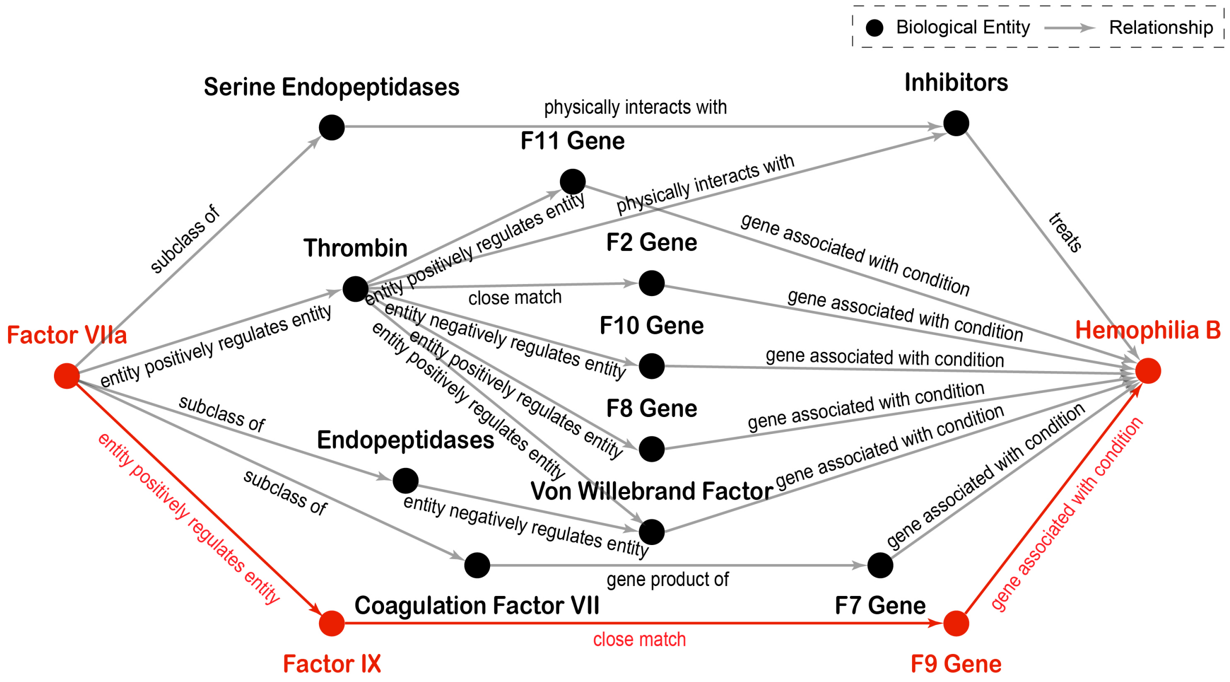

Figure 1: Top 10 predicted 3-hop paths (integrated into a graph for better visualization) generated by the KGML-xDTD model framework serve as biological explanations of the predicted "treats" relationship between Factor VIIa and Hemophilia B. The path highlighted with red is the one in which all nodes can align with the key molecules in the real drug action regulatory network shown in Figure 4 in the main text.

References

Wood EC, Glen AK, Kvarfordt LG, Womack F, Acevedo L, Yoon TS, et al. RTX-KG2: a system for building a semantically standardized knowledge graph for translational biomedicine. BMC Bioinformatics 2022;23(1):400.

Kilicoglu H, Shin D, Fiszman M, Rosemblat G, Rindflesch TC. SemMedDB: a PubMed-scale repository of biomedical semantic predications. Bioinformatics 2012;28(23):3158–3160.

Cong Q, Feng Z, Li F, Zhang L, Rao G, Tao C. Constructing Biomedical Knowledge Graph Based on SemMedDB and Linked Open Data. In: 2018 IEEE International Conference on Bioinformatics and Biomedicine (BIBM); 2018. p. 1628–1631.

Cilibrasi RL, Vitanyi PMB. The Google Similarity Distance. IEEE Transactions on Knowledge and Data Engineering 2007;19(3):370–383.

Unni DR, Moxon SA, Bada M, Brush M, Bruskiewich R, Caufield JH, et al. Biolink Model: A universal schema for knowledge graphs in clinical, biomedical, and translational science. Clinical and Translational Science 2022;.

Xin J, Afrasiabi C, Lelong S, Adesara J, Tsueng G, Su AI, et al. Cross-linking BioThings APIs through JSON-LD to facilitate knowledge exploration. BMC Bioinformatics 2018;19(1):30.

Banda JM, Evans L, Vanguri RS, Tatonetti NP, Ryan PB, Shah NH. A curated and standardized adverse drug event resource to accelerate drug safety research. Scientific Data 2016;3(1):160026.

Degtyarenko K, Matos Pd, Ennis M, Hastings J, Zbinden M, McNaught A, et al. ChEBI: a database and ontology for chemical entities of biological interest. Nucleic Acids Research 2008;36(Database issue):D344–D350.

Gaulton A, Bellis LJ, Bento AP, Chambers J, Davies M, Hersey A, et al. ChEMBL: a large-scale bioactivity database for drug discovery. Nucleic Acids Research 2012;40(D1):D1100–D1107.

Ursu O, Holmes J, Knockel J, Bologa CG, Yang JJ, Mathias SL, et al. DrugCentral: online drug compendium. Nucleic Acids Research 2016 10;45(D1):D932–D939. https://doi.org/10.1093/nar/gkw993.

Peryea T, Southall N, Miller M, Katzel D, Anderson N, Neyra J, et al. Global Substance Registration System: consistent scientific descriptions for substances related to health. Nucleic Acids Research 2020 11;49(D1):D1179–D1185. https://doi.org/10.1093/nar/gkaa962.

Thorn CF, Klein TE, Altman RB. PharmGKB: the Pharmacogenomics Knowledge Base. Methods in molecular biology (Clifton, NJ) 2013;1015:311–20.

Kim S, Thiessen PA, Bolton EE, Chen J, Fu G, Gindulyte A, et al. PubChem Substance and Compound databases. Nucleic Acids Research 2016;44(Database issue):D1202–D1213.

Kuhn M, Letunic I, Jensen LJ, Bork P. The SIDER database of drugs and side effects. Nucleic Acids Research 2015 10;44(D1):D1075–D1079. https://doi.org/10.1093/nar/gkv1075.

Hamilton WL, Ying R, Leskovec J. Inductive Representation Learning on Large Graphs. arXiv 2017;.

Gu Y, Tinn R, Cheng H, Lucas M, Usuyama N, Liu X, et al. Domain-Specific Language Model Pretraining for Biomedical Natural Language Processing. ACM Transactions on Computing for Healthcare 2022;3(1):1–23.

Zhao K, Wang X, Zhang Y, Zhao L, Liu Z, Xing C, et al. Leveraging Demonstrations for Reinforcement Recommendation Reasoning over Knowledge Graphs. Proceedings of the 43rd International ACM SIGIR Conference on Research and Development in Information Retrieval 2020;p. 239–248.

Page L, Brin S, Motwani R, Winograd T. The PageRank Citation Ranking: Bringing Order to the Web. Stanford InfoLab; 1999.

Glorot X, Bengio Y. Understanding the difficulty of training deep feedforward neural networks. In: Teh YW, Titterington DM, editors. AISTATS, vol. 9 of JMLR Proceedings JMLR.org; 2010. p. 249–256.

Kingma DP, Ba J. Adam: A Method for Stochastic Optimization. CoRR 2015;abs/1412.6980.

Lin XV, Socher R, Xiong C. Multi-Hop Knowledge Graph Reasoning with Reward Shaping. Proceedings of the 2018 Conference on Empirical Methods in Natural Language Processing 2018;p. 3243–3253.
