# Supplementary figures and images for: KGML-xDTD: a knowledge graph–based machine learning framework for drug treatment prediction and mechanism description
Source: Gigascience. 2023 Aug 21;12:giad057. doi: 10.1093/gigascience/giad057 (PMC10441000; doi:10.1093/gigascience/giad057)

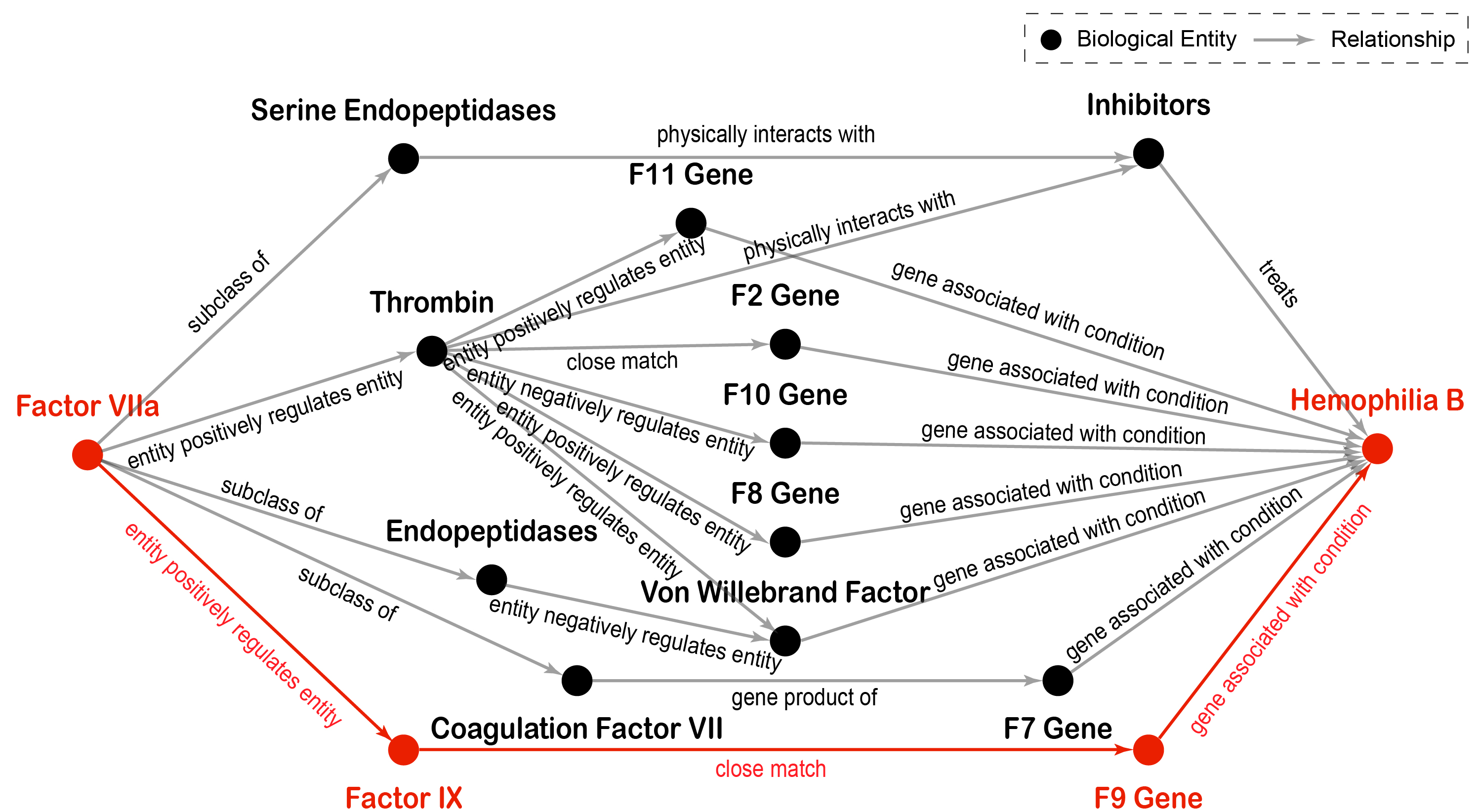

Supplement: giad057_Supplemental_Files [file giad057_supplemental_files.zip › suppl_figure1.jpg]
