# Supplementary material for: KGML-xDTD: a knowledge graph–based machine learning framework for drug treatment prediction and mechanism description
Source: Gigascience. 2023 Aug 21;12:giad057. doi: 10.1093/gigascience/giad057 (PMC10441000; doi:10.1093/gigascience/giad057)
Supplement: giad057_Response_to_Reviewer_Comments_Original_Submission [file giad057_response_to_reviewer_comments_original_submission.pdf]

Author's Response To Reviewer Comments

Close

We'd like to thank the reviewers and editor for their valuable comments and suggestions which have been incorporated in the revised version. We've also corrected some typos and revised certain sentences to improve clarity in the revised version. We've updated the data and code on Github and Zenodo for the new experiment in the revised version. Please see our responses to individual comments below.

Reviewer1:
1) It is mentioned on page 6 that the data are classified into three categories, while other data are classified into two categories. How did you exclude the "unknown" category and adjusted result?

Thank you for this and the following comments. To adjust the three-classification accuracy and macro F1 score to binary classification, for the three-class predicted probabilities of each drug-disease pair, we first excluded the "unknown" probability and then used the class with the highest probability among the remaining two classes as the predicted class for calculating two-category accuracy and macro F1 score.

For instance, suppose a drug-disease pair has three probabilities: 0.1, 0.5, 0.4 (respectively corresponding to "not treat," "treat," and "unknown" classes). In that case, we exclude 0.4 and predict this drug-disease pair as "treat" as its "treat" probability 0.5 is higher than 0.1.

2) Drug Repurposing Prediction model and Mechanism of Action Prediction model seems to be two separate training model. I can not find evidence of multitasking training from the content. If the model is trained separately, which model is the evaluation metrics according to? If training together, the model section should be written more clearly.

Yes, the Drug Repurposing Prediction (DRP) model and the Mechanism of Action Prediction (MOA) model are two models and trained separately. However, the training process of the MOA model needs results from the trained DRP model, that is, we used the predicted "treat" probability of the DRP model as reward for the MOA training. We separately provided the details of these two models in Section "Model Framework". In this section, there are two subsections "Drug Repurposing Prediction (DRP) Module" and "Mechanism of Action (MOA) Prediction Module" that separately and clearly describe these two models in the revised version of paper.

Sorry for the confusion about the evaluation metrics. For the DRP model, we evaluate its performance using six metrics (e.g., Accuray, Macro F1 score, MRR, Hit@1, Hit@3, and Hit@5). For the MOA model, the evaluation of its performance uses four metrics (e.g., MRR, Hit@1, Hit@3, and Hit@5) along with the comparison between the model-predicted paths and DrugMechDB-based MOA paths in two case studies. We describe how to calculate these metrics in the subsection "Evaluation Metrics". Although both models use MRR, Hit@1, Hit@3, and Hit@5, the underlying data is not the same. The DRP model uses these metrics based on rank of true positive drug-disease pairs while for MOA it is based on DrugMechDB-matched BKG-based 3-hop paths (described in sub-section "DrugMechDB" on page 4 in the revised version).

3) The introduction part only mentioned about Drug Repurposing Prediction Model, but it didn't describe existing Mechanism Of Action Prediction model.

In the introduction we describe some existing models that could be used for drug repurposing explanations via BKG-based paths on the paragraphs 4 and 5 of the introduction, such as UKGE, GrEDel, PoLo. However, as we mentioned there, these models are not primarily designed for finding biological meaning paths for drug repurposing in a large and complex biomedical knowledge graph (e.g., RTX-KG2). In addition, in Section "MOA prediction evaluation" on page 9, we explained why they cannot be scaled to RTX-KG2 for

comparison with our model. The reasons include the need to have edge weight information (e.g., UKGE), a lack of source code (e.g., GrEDel), and expensive computational time (e.g., PoLo). Nonetheless, we attempted to utilize a model with a similar model structure to GrEDel for comparison, such as MultiHop which also uses LSTM layers for path reasoning.

4) Baseline seems to be Drug Repurposing Prediction SOTA model. But the best performance of the work is about Mechanism Of Action Prediction.

Our framework contains two modules: the DRP model and the MOA model. We separately compare these models with other existing available baseline models for the same goals: Drug Repurposing Prediction and Mechanism Of Action prediction, and characterize performance of both models against appropriate baselines. Table 2 and Figure 3 shows the comparison results between the DRP model and its baseline models. In these comparison results, we show that the DRP model outperforms most of the baseline models and achieves comparable performance as GAT based on Accuracy and Macro F1 score while having superiority over baselines in relatively low false positives. In Table 3, we show the MOA model can identify the biologically reasonable choices with a much higher ranking compared with two baseline models.

To better clarify these two kinds of comparisons, we've modified the captions of the tables and figure mentioned above.

5) The data set appears to selectively chose drug-disease pairs with intermediate paths. But if the drug or disease in the network do not connect, that how dose Drug Repurposing Prediction model perform?

Our KGML-xDTD model framework can achieve two goals namely Drug Repurposing Prediction and Mechanism Of Action prediction, for drug-disease pairs via the DRP model and the MOA model. The DRP model leverages the node attribute information and the graph topology to extract features for each node in the biomedical knowledge graph (BKG) for drug repurposing prediction. Therefore, it does not require a drug-disease pair to have connections in the graph, highlighting the power of using the GNN model for knowledge graph completion. In contrast, for Mechanism of Action prediction, since we utilize the BKG-based paths as mechanism of action for drug repurposing, we indeed need a drug-disease pair to have connections. Otherwise, the model can't explain its mechanism based on the knowledge graph. This is to be expected, since the MOA prediction is predicated on the existence of intermediate nodes and edges to describe the predicted mechanism.
Therefore, for the results in Table 2 that are used for evaluating the performance of DRP model, they are based on the drug-disease pairs in the test set in which some with connection and some without connection (up to 3 hops) in the graph. We don't selectively choose only drug-disease pairs with intermediate paths.

In our test set, we have 2,145 true positive drug-disease pairs (in which 1,950 are connected and 195 are not connected up to 3 hops) and 3,319 true negative drug-disease pairs (2,793 are connected and 526 are not connected up to 3 hops). To answer your question, we did an additional experiment based on those "disconnected" drug-disease pairs (195 true positive and 526 true negative) to report the KGML-xDTD model's performance on the DRP task compared with other baselines below:

**Model**	**Accuracy**	**Macro F1 score**	**MRR**	**Hit@1**	**Hit@3**	**Hit@5**
TransE	0.940	0.922	0.182 (+/- 0.006)	0.074 (+/- 0.011)	0.164 (+/- 0.015)	0.244 (+/- 0.017)
TransR	0.908	0.886	0.314 (+/- 0.014)	0.165 (+/- 0.015)	0.347 (+/- 0.031)	0.479 (+/- 0.037)
RotatE	0.904	0.877	0.230 (+/- 0.009)	0.088 (+/- 0.013)	0.245 (+/- 0.025)	0.376 (+/- 0.029)
DistMult	0.728	0.426	0.195 (+/- 0.009)	0.061 (+/- 0.010)	0.177 (+/- 0.021)	0.328 (+/- 0.036)
ComplEx	0.731	0.427	0.092 (+/- 0.007)	0.018 (+/- 0.008)	0.052 (+/- 0.010)	0.115 (+/- 0.013)
ANALOGY	0.730	0.422	0.183 (+/- 0.012)	0.044 (+/- 0.012)	0.167 (+/- 0.019)	0.321 (+/- 0.034)
SimplE	0.728	0.421	0.165 (+/- 0.012)	0.037 (+/- 0.012)	0.154 (+/- 0.024)	0.284 (+/- 0.023)
GAT	0.967	0.958	0.005(+/-0.002)	0.001 (+/-0.002)	0.001 (+/-0.002)	0.002 (+/-0.002)

GraphSAGE-link	0.950	0.936	0.002 (+/- 0.000)	0.000 (+/- 0.000)	0.000 (+/- 0.000)	0.001 (+/- 0.002)
GraphSAGE+logistic	0.852	0.820	0.010 (+/- 0.003)	0.005 (+/- 0.004)	0.006 (+/- 0.004)	0.010 (+/- 0.002)
GraphSAGE+SVM	0.864	0.828	0.007 (+/- 0.002)	0.002 (+/- 0.002)	0.005 (+/- 0.003)	0.008 (+/- 0.003)
KGML-xDTD w/o NAEs	0.927 (0.949*)	0.859 (0.933*)	0.181 (+/- 0.012)	0.083 (+/- 0.012)	0.169 (+/- 0.022)	0.258 (+/- 0.018)
2-class KGML-xDTD	0.971	0.962	0.388 (+/- 0.014)	0.310 (+/- 0.020)	0.419 (+/- 0.017)	0.476 (+/- 0.018)
KGML-xDTD (ours)	0.951 **(0.971*)**	0.908 **(0.963*)**	**0.416 (+/- 0.009)**	**0.302 (+/- 0.015)**	**0.444 (+/- 0.015)**	**0.535 (+/- 0.015)**

Based on the results (please put this table above in a markdown editor for better visualization), our model still outperforms other baseline models even when the drugs and diseases under consideration are not connected within 3 hops.

Reviewer2:
1) The baseline models (GAT & GraphSAGE+SVM) only use a small subset of drug-disease replacements. The authors indicate that the smaller subset is necessary owing to time performance constraints. However, there is no discussion as to the possible impact the reduced subset any aspects in relation to their method.

Thank you for this and the following comments. For the comparison between our KGML-xDTD model with GAT and GraphSAGE+SVM models, and even other baselines for Drug Repurposing prediction goal, we used six different metrics (e.g., Accuracy, Macro F1 score, MRR, Hit@1, Hit@3 and Hit@5) described in Section "Evaluation Metrics" on page 6. The small (1,000 pairs) random subset of drug-disease replacement is only used for the calculation of MRR, Hit@1, Hit@3 and Hit@5. In order to improve the robustness and generalizability of the small subset-based result, we ran this small subset experiment 10 times (each with different 1,000 random subset) and showed the average and standard deviation of MRR, Hit@1, Hit@3 and Hit@5 on Table 2 in the revised version; showing little variance over the 10 iterations of 1,000. Based on the experiment results, their (GAT & GraphSAGE+SVM) performances are still worse than our model's, which is consistent with the comparison with "complete" replacement methods in Figure 3. Therefore, in Section "Drug Repurposing Prediction Evaluation", we've emphasized excluding these two models in the comparison with "complete" replacement does not affect our conclusion that the KGML-xDTD is superior to other baseline models in identifying new indications of existing drugs with relatively low false positives.

2) The approach only evaluate 3-hop KG paths, which is 1/7 of what is available in DrugMechDB. What is the quality/performance impact of choosing longer paths? Wouldn't the the number of biologically reasonable paths to explain a predict be substantially reduced? I worry that this is cherry picking the dataset to show good performance for the only case (3-hop) that it is capable of (While critizing other methods as not being performant)

The reason why the matched 3-hop KG-based paths are only approximately 1/7 of DrugMechDB paths is due to the incompleteness of RTX-KG2 and the imperfect concept matching between DrugMechDB and KG2. Although the RTX-KG2 is one of the largest open-source biomedical knowledge graphs, it still cannot cover all biomedical knowledge and relations in biomedicine and entity identification between DrugMechDB and RTX-KG2 is imperfect (given they were developed independently). The incompleteness is common in all knowledge graphs that is why we need to use machine learning models for their completion. In addition, please note that the DrugMechDB paths are derived from free-text descriptions found on DrugBank, Wikipedia as well as within literature, and are then normalized via the biolink model and curated by human experts. However, the KG paths are constructed based on the existing triplets from multiple data sources which are more abstract and compressed. Thus, some edges and concepts in the DrugMechDB paths may not exist in KGs. This is also observed in the paper https://doi.org/10.1093/bioinformatics/btac205.

In sub-section “DrugMechDB” on page 2, we require that the 3-hop KG2-based paths that are considered to match the DrugMechDB paths are those with all nodes showing up in the complete DrugMechDB paths. Considering three factors: the incompleteness of RTX-KG2, the imperfect concept matching, and the drug-disease pairs should be connected, only 1/7 drug-diseases satisfy these requirements. Therefore, we didn't cherry pick the dataset: the 1/7 was merely the fraction of 3-hop paths that passed the criteria (all 4 nodes in the 3 hop path matching between the BKG and DrugMechDB). We have now clarified this at the end of the “DrugMechDB” section.

Regarding your concern about the quality/performance of choosing longer paths, although we used 3-hop paths in our experiments for practical reasons, this does not mean that the predicted paths are fundamentally limited to only those with a length of 3 (as any path with up to length 3 can be returned, due to the self loops): we simply picked 3 due to GPU memory constraints and training time. Furthermore, it is important to note that our model's good performance is mainly attributed to using biologically meaningful "demonstration paths" during training rather than any path-length restrictions. That is evidenced by the comparison between KGML-xDTD and its variant KGML-xDTD w/o DP (which does not utilize demonstration paths) in Table 3.

3) The authors use RepoDB as one of their sources, and specifically use the "withdrawn" set as true negatives. However, most withdrawn tags are linked to reasons other than safety or efficacy of the clinical trial. As such it is not clear that this set is a good true negative set.

Sorry for this typo. We used the “terminated” set instead of the “withdrawn” set in our training data. The “terminated”indicates that the trial has been stopped due to the safety or efficacy of the clinical trial and will not be resumed, so I think it should be reasonable to be used as a good true negative set. We’ve corrected this typo in the revised version.

4) The authors use MyChem as a resource for drug indications/contraindications. However, MyChem is not an original source - it aggregates other resources. The authors should properly identify the source of "human curated annotations".

Thanks for this suggestion. For MyChem data, we’ve summarized the original resources that this dataset used and labeled their reliability in the supplementary section S2. Upon reviewing these data resources, we can confirm that they are reliable as they have either been human curated or approved by FDA/NIH. However, some sources approved by FDA/NIH are not explicitly labeled as "human curated". Therefore, we have modified our manuscript to claim that these sources are reliable annotations rather than “human curated annotations”.

5) I commend the authors for their evaluation, which uses a number of different train/test strategies and against different methods. However, as far as i can see the train/test strategy does not adequately remove similar true drugs-disease pairs from the training/test set. That is to say there are many drugs that are approved for very similar conditions, and therefore it becomes somewhat trivial to predict these (this problem is highlighted in the 2011 PREDICT paper by assaf gottlieb). More work should be done here to report an accuracy based on more stringent evaluation criteria.

Thanks for this question. Regarding the similarity of drug/chemical signatures, such as SMILES, drug side effects, drug-related gene profiles/sequences, mentioned in the 2011 PREDICT paper, unlike the logistic regression used in that paper, KGML-xDTD model does not leverage such information for prediction. Only information utilized by our model are the node attribute (e.g. node name and category) and graph topology structure of RTX-KG2 knowledge graph (KG). In the KG reprocessing (please refer to the “customized biomedical knowledge graph” section), we have removed all existing drug-disease edges in the graph, that is to say the model can only use other potential graph connections (e.g., similar neighborhood) between drugs and diseases for prediction. This is also the mechanism of the GraphSAGE model. Although some drugs in the training/test set might have similar neighborhood topology structure, we don’t think they belong to the data leakage because these features are what the model depends on for predictions. Therefore, we believe that our data split strategy does not result in data leakage between the training set and test set.

In addition, inspired by the 2011 PREDICT paper, to demonstrate our model does not utilize the drug class information for prediction, we use MyChem API to access the FDA "Established Pharmacologic Class" (EPC) for each drug in the training set. Then for each disease in the test set, we utilize the KGML-xDTD model to predict top 100 drugs after excluding the known drug-disease pairs. We count the number of drug classes among these 100 predicted drugs that are not seen in the training set for each disease. Our analysis (see Figure 8 in the revised version) reveals that each disease has at least 70 different drug classes among the top 100 predicted drugs, indicating that our model does not rely on any "drug class" information shared between the training and test sets for predictions. This analysis has been added into a new section "Drug Class Analysis" in the revised version.

6) It's unclear to me that the 124k diseases are real (diagnosable) diseases that could be prescribed for. Inflating the number of possible (but implausible) diseases might augment the performance, but contribute nothing to medicine. Elaborate.

There are two reasons why the number of diseases in RTX-KG2 (the biomedical knowledge graph we used) is large. Firstly, this is due to permissive and detailed definitions of disease in the biomedical knowledge graph and multiple knowledge sources (e.g. UMLS, ChEMBL, DrugBank) it includes. Hence it includes disease entities such as "hypertension, mild", "hypertension, severe", "hypertension, renal" etc. Secondly, in Section "Notations" on page 2, we define the disease nodes to have categories of either "Disease", "PhenotypicFeature" (e.g. Astasia, A postural abnormality characterized by the inability to stand without external support despite having sufficient muscle strength.) or "DiseaseOrPhenotypicFeature" in RTX-KG2. Therefore, the 124k "diseases" include many variants of what is traditionally thought of as "a single disease" (as in the hypertension example before) as well as symptoms, all of which may be clinically relevant depending on the context.. However, the training data we collected from four data sources mainly consists of those diseases traditionally understood to be diagnosable.

These factors indicate that it becomes more difficult to correctly identify the true positive drug-disease pairs and their mechanism of action among many negative pairs, rather than easier to augment the performance. In addition, due to more permissive and detailed definitions of diseases in RTX-KG2, it is more useful to understand various types of diseases, highlighting the ability of our model in contributing to medicine.

7) Figures 5, 6 are difficult to read

We have re-generated these two figures in the revised version by using larger fonts and showing only the top 5 paths for each drug-disease pair rather than top 10 for better visualization.

8) It's nice to see the 2 use cases in the paper. However, the extracted subgraphs are quite different than the DrugMechDB MOA paths. So there's something to be said about the succinctness of the DrugMechDB MOA paths, which might prove to be a better training set for some explanation algorithm, rather that one that is independently generated.

Thanks for your comment. As mentioned in Question 2), the difference between extracted subgraphs and DrugMechDB MOA paths are due to the incompleteness of KGs, imperfect concept matching, and/or the length limit to 3. Note that DrugMechDB paths are derived from free-text description. Compared with text, the knowledge graph is a more abstract and compressed representation of knowledge. Therefore, it is quite difficult to make them exactly the same, hence would require further curation (on the order of the construction of DrugMechDB itself) in order to transform DrugMechDB MOA paths into a form that would be suitable for training our approach. Hence why we used DrugMechDB for comparison, not training.

Close
