# Supplementary material for: KGML-xDTD: a knowledge graph–based machine learning framework for drug treatment prediction and mechanism description
Source: Gigascience. 2023 Aug 21;12:giad057. doi: 10.1093/gigascience/giad057 (PMC10441000; doi:10.1093/gigascience/giad057)
Supplement: giad057_Response_to_Reviewer_Comments_Revision_2 [file giad057_response_to_reviewer_comments_revision_2.pdf]

Author's Response To Reviewer Comments

Close

We have resolved all minor issues.

Close
