# Supplementary material for: KGML-xDTD: a knowledge graph–based machine learning framework for drug treatment prediction and mechanism description
Source: Gigascience. 2023 Aug 21;12:giad057. doi: 10.1093/gigascience/giad057 (PMC10441000; doi:10.1093/gigascience/giad057)
Supplement: giad057_Reviewer_1_Report_Original_Submission — Yuansheng Liu -- 3/3/2023 Reviewed [file giad057_reviewer_1_report_original_submission.pdf]

Reviewer Report

Title: KGML-xDTD: A Knowledge Graph-based Machine Learning Framework for Drug Treatment Prediction and Mechanism Description

Version: Original Submission **Date:** 3/3/2023

Reviewer name: Yuansheng Liu

Reviewer Comments to Author:

The paper entitled "KGML-xDTD: A Knowledge Graph-based Machine Learning Framework for Drug Treatment Prediction and Mechanism Description" proposes KGML-xDTD, a two-module, knowledge graph-based machine learning framework . Author constructs a large knowledge graph for the training of the model. The model is divided into two modules, one for drug repurposing prediction and the other for Mechansim Of Action Prediction. Both modules have achieved good results compared with the existing baseline.
Here are my specific points:
(1) It is mentioned on page 6 that the data are classified into three categories, while other data are classified into two categories. How did you exclude the "unknown" category and adjusted result?
(2) Drug Repurposing Prediction model and Mechanism of Action Prediction model seems to be two separate training model. I can not find evidence of multitasking training from the content. If the model is trained separately, which model is the evaluation metrics according to? If training together, the model section should be written more clearly.
(3) The introduction part only mentioned about Drug Repurposing Prediction Model, but it didn't describe existing Mechanism Of Action Prediction model.
(4) Baseline seems to be Drug Repurposing Prediction SOTA model. But the best performance of the work is about Mechanism Of Action Prediction.
(5) The data set appears to selectively chose drug-disease pairs with intermediate paths. But if the drug or disease in the network do not connect, that how dose Drug Repurposing Prediction model perform?

Methods

Are the methods appropriate to the aims of the study, are they well described, and are necessary controls included? Choose an item.

Conclusions

Are the conclusions adequately supported by the data shown? Choose an item.

Reporting Standards

Does the manuscript adhere to the journal's guidelines on [minimum standards of reporting?]() Choose an item.

Choose an item.

Statistics

Are you able to assess all statistics in the manuscript, including the appropriateness of statistical tests used? Choose an item.

Quality of Written English

Please indicate the quality of language in the manuscript: Choose an item.

Declaration of Competing Interests

Please complete a declaration of competing interests, considering the following questions:

- Have you in the past five years received reimbursements, fees, funding, or salary from an organisation that may in any way gain or lose financially from the publication of this manuscript, either now or in the future?
- Do you hold any stocks or shares in an organisation that may in any way gain or lose financially from the publication of this manuscript, either now or in the future?
- Do you hold or are you currently applying for any patents relating to the content of the manuscript?
- Have you received reimbursements, fees, funding, or salary from an organization that holds or has applied for patents relating to the content of the manuscript?
- Do you have any other financial competing interests?
- Do you have any non-financial competing interests in relation to this paper?

If you can answer no to all of the above, write 'I declare that I have no competing interests' below. If your reply is yes to any, please give details below.

I declare that I have no competing interests

I agree to the open peer review policy of the journal. I understand that my name will be included on my report to the authors and, if the manuscript is accepted for publication, my named report including any attachments I upload will be posted on the website along with the authors' responses. I agree for my report to be made available under an Open Access Creative Commons CC-BY license (http://creativecommons.org/licenses/by/4.0/). I understand that any comments which I do not wish to be included in my named report can be included as confidential comments to the editors, which will not be published.

Choose an item.

To further support our reviewers, we have joined with Publons, where you can gain additional credit to further highlight your hard work (see: https://publons.com/journal/530/gigascience). On publication of this paper, your review will be automatically added to Publons, you can then choose whether or not to claim your Publons credit. I understand this statement.

Yes Choose an item.
