# Supplementary material for: KGML-xDTD: a knowledge graph–based machine learning framework for drug treatment prediction and mechanism description
Source: Gigascience. 2023 Aug 21;12:giad057. doi: 10.1093/gigascience/giad057 (PMC10441000; doi:10.1093/gigascience/giad057)
Supplement: giad057_Reviewer_2_Report_Original_Submission — Michel Dumontier -- 4/2/2023 Reviewed [file giad057_reviewer_2_report_original_submission.pdf]

Reviewer Report

Title: KGML-xDTD: A Knowledge Graph-based Machine Learning Framework for Drug Treatment Prediction and Mechanism Description

Version: Original Submission **Date:** 4/2/2023

Reviewer name: Michel Dumontier

Reviewer Comments to Author:

This paper describes KGML-xDTD, a knowledge graph-based ML framework to predict and explain potential applications of drugs. The main approach is the use of graph reinforcement learning to predict drug-disease pairs and provide a knowledge-based path as a potential mechanism of action. The method is evaluated against other approaches, various data partitioning strategies, comparison to a manually curated database of mechanisms of actions, and two use cases.
The paper is well written, easy to read, and makes a contribution to the scientific literature. Accurate prediction of drug uses remains an important and challenging problem in biomedical informatics. The novelty of the approach is to use graph reinforcement learning to achieve state of the art performance for the problem, and it also is able to generate plausible paths within a knowledge graph to serve as mechanistic explanations.
There are some limitation to the work that should be addressed. These include:
1) The baseline models (GAT & GraphSAGE+SVM) only use a small subset of drug-disease replacements. The authors indicate that the smaller subset is necessary owing to time performance constraints. However, there is no discussion as to the possible impact the reduced subset any aspects in relation to their method.
2) The approach only evaluate 3-hop KG paths, which is 1/7 of what is available in DrugMechDB. What is the quality/performance impact of choosing longer paths? Wouldn't the the number of biologically reasonable paths to explain a predict be substantially reduced? I worry that this is cherry picking the dataset to show good performance for the only case (3-hop) that it is capable of (While critizing other methods as not being performant)
3) The authors use RepoDB as one of their sources, and specifically use the "withdrawn" set as true negatives. However, most withdrawn tags are linked to reasons other than safety or efficacy of the clinical trial. As such it is not clear that this set is a good true negative set.
4) The authors use MyChem as a resource for drug indications/contraindications. However, MyChem is not an original source - it aggregates other resources. The authors should properly identify the source of "human curated annotations".
5) I commend the authors for their evaluation, which uses a number of different train/test strategies and against different methods. However, as far as i can see the train/test strategy does not adequately remove similar true drugs-disease pairs from the training/test set. That is to say there are many drugs that are approved for very similar conditions, and therefore it becomes somewhat trivial to predict these (this problem is highlighted in the 2011 PREDICT paper by assaf gottlieb). More work should be

done here to report an accuracy based on more stringent evaluation criteria.
6) It's unclear to me that the 124k diseases are real (diagnosable) diseases that could be prescribed for. Inflating the number of possible (but implausible) diseases might augment the performance, but contribute nothing to medicine. Elaborate.
7) Figures 5, 6 are difficult to read
8) It's nice to see the 2 use cases in the paper. However, the extracted subgraphs are quite different than the DrugMechDB MOA paths. So there's something to be said about the succinctness of the DrugMechDB MOA paths, which might prove to be a better training set for some explanation algorithm, rather that one that is independently generated.
Overall, this is a nice paper with an interesting approach.

Methods

Are the methods appropriate to the aims of the study, are they well described, and are necessary controls included? Choose an item.

Conclusions

Are the conclusions adequately supported by the data shown? Choose an item.

Reporting Standards

Does the manuscript adhere to the journal's guidelines on [minimum standards of reporting?] Choose an item.

Choose an item.

Statistics

Are you able to assess all statistics in the manuscript, including the appropriateness of statistical tests used? Choose an item.

Quality of Written English

Please indicate the quality of language in the manuscript: Choose an item.

Declaration of Competing Interests

Please complete a declaration of competing interests, considering the following questions:

- Have you in the past five years received reimbursements, fees, funding, or salary from an organisation that may in any way gain or lose financially from the publication of this manuscript, either now or in the future?

- Do you hold any stocks or shares in an organisation that may in any way gain or lose financially from the publication of this manuscript, either now or in the future?
- Do you hold or are you currently applying for any patents relating to the content of the manuscript?
- Have you received reimbursements, fees, funding, or salary from an organization that holds or has applied for patents relating to the content of the manuscript?
- Do you have any other financial competing interests?
- Do you have any non-financial competing interests in relation to this paper?

If you can answer no to all of the above, write 'I declare that I have no competing interests' below. If your reply is yes to any, please give details below.

I declare that I have no competing interests.

I agree to the open peer review policy of the journal. I understand that my name will be included on my report to the authors and, if the manuscript is accepted for publication, my named report including any attachments I upload will be posted on the website along with the authors' responses. I agree for my report to be made available under an Open Access Creative Commons CC-BY license (http://creativecommons.org/licenses/by/4.0/). I understand that any comments which I do not wish to be included in my named report can be included as confidential comments to the editors, which will not be published.

Choose an item.

To further support our reviewers, we have joined with Publons, where you can gain additional credit to further highlight your hard work (see: https://publons.com/journal/530/gigascience). On publication of this paper, your review will be automatically added to Publons, you can then choose whether or not to claim your Publons credit. I understand this statement.

Yes Choose an item.
